# Supplementary material for: Cu-catalyzed asymmetric regiodivergent electrosynthesis and its application in the enantioselective total synthesis of (-)-fumimycin
Source: Nat Commun. 2023 Oct 24;14:6749. doi: 10.1038/s41467-023-42603-w (PMC10598217; doi:10.1038/s41467-023-42603-w)
Supplement: Supplementary file 1 — Supplementary Information [file 41467_2023_42603_MOESM1_ESM.pdf]

Supplementary Information

Cu-catalyzed asymmetric regiodivergent electrosynthesis and its application in the enantioselective total synthesis of (-)-fumimycin

Tian Xie[1], Jianming Huang[2], Juan Li[2], Lingzi Peng[1], Jin Song[2], Chang Guo[1]*

[1]Hefei National Research Center for Physical Sciences at the Microscale and Department of Chemistry, University of Science and Technology of China, Hefei, 230026, China.

[2]Institutes of Physical Science and Information Technology, Key Laboratory of Environment-Friendly Polymeric Materials of Anhui Province, Anhui University, Hefei, 230601, China.

*Corresponding author. Email: guochang@ustc.edu.cn

CONTENTS:

1. Supplementary Note......2
2. Supplementary Methods......3
3. Supplementary Figure......46
4. Supplementary References......162

1. Supplementary Note

General Information

Unless otherwise noted, all reagents were purchased from commercial suppliers and used without further purification. Cyclic voltammograms were recorded on a CHI 760E potentiostat. ^{1}H NMR and ^{13}C NMR spectra were recorded at 25 ºC on Bruker Advance 500M NMR spectrometers and JEOL 600 MHz spectrometers. High-resolution mass spectral analysis (HRMS) was performed on Waters XEVO G2 Q-TOF. Optical rotations were determined at 589 nm (sodium D line) by using a Perkin-Elmer-343 polarimeter (1 dm path length cell). The measurement of enantiomeric excesses was performed on Waters-Alliance (2998, Photodiode Array Detector). Chiralpak IA, IF, IG, and AD-H columns were purchased from Daicel Chemical Industries, LTD.

2. Supplementary Methods

2.1 Synthesis and Characterization of Substrate

General procedure A: Synthesis of cyclic imino methyl esters

1a-1ap were prepared according to a modified literature procedure.[1-3] **1a**, **1ab**, and **1ad** were prepared according to method A. **1ac** and **1ae-1ak** were prepared according to method B. **1al** and **1ap** were prepared according to method C.

Method A:

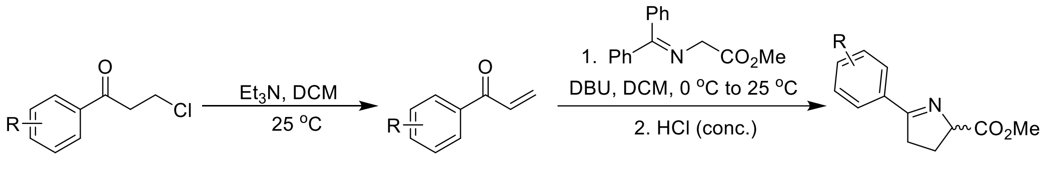

1a, **1ab**, **1ad**

To a round bottom flask was added 3-chloro-1-phenylpropan-1-one (30 mmol) and CH_2Cl_2 (60 mL), then Et_3N (33 mmol) was added dropwise for 10 min. After this the reaction was stirred at 25 °C for about 3 h, and the solvent was removed under reduced pressure. The residue was dissolved in THF, filtered and concentrated in vacuo to obtain the vinyl ketone.

To a solution of vinyl ketone (28 mmol) and (*N*,*N*-diphenylmethylgene)glycine methyl ester (25 mmol) in CH_2Cl_2 (25 mL) at 0 °C was added DBU (2.5 mmol). After stirring at 25 °C for 12 h, HCl (12 N, 7.5 mmol) was added and the reaction mixture was stirred at 25 °C overnight. Then, water was added and the mixture was extracted with CH_2Cl_2. The organic layers were combined, dried over $MgSO_4$, filtered and concentrated in vacuo. The crude residue was then purified by flash chromatography (petroleum ether/ethyl acetate, 8:1) over silica gel to obtain the products (**1a**, **1ab**, **1ad**).

Method B:

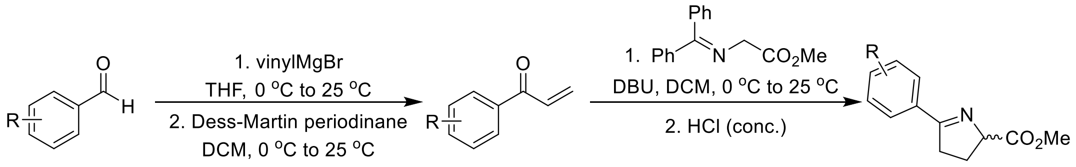

1ac, **1ae-1ak**

Under Ar atmosphere, the mixture of aldehyde (15 mmol) and dry THF (25 mL) was cooled to 0 °C, and vinylmagnesium bromide (2.0 M solution in THF, 18 mmol) was added dropwise. The mixture was warmed to 25 °C and stirred overnight. A saturated NH_4Cl solution (20 mL) was added to quench the reaction and the aqueous layer was extracted with EtOAc (30 mL × 3). The combined organic layers were washed with brine (30 mL), dried over anhydrous $MgSO_4$, filtered and concentrated in vacuo. The residue was dissolved in CH_2Cl_2 (50 mL) and Dess-Martin periodinane (20 mmol) was added. The mixture was stirred at 25 °C for 5 h. Saturated $NaHCO_3$ solution (20 mL) and saturated $Na_2S_2O_3$ solution (20 mL) were added to quench the reaction and the aqueous layer was extracted with CH_2Cl_2 (30 mL × 3). The combined organic layers were dried over anhydrous $MgSO_4$, filtered and concentrated in vacuo. The residue was chromatographed (petroleum ether/ethyl acetate, 50:1) on silica gel to get the vinyl ketone. Then, the following steps were the same as those shown in method A.

Method C:

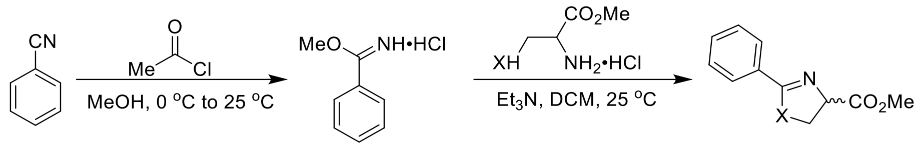

1al, X = S
1ap, X = O

Acetyl chloride (240 mmol) was added dropwise to a stirred solution of benzonitrile (30 mmol) in anhydrous methanol (360 mmol) at 0 °C. The mixture was stirred at 25 °C. After the complete consumption of benzonitrile, the solvent was removed under reduced pressure. The residue was dissolved in diethyl ether. Then, the precipitate was filtered, and washed with diethyl ether to afford methyl benzimidate hydrochloride.

To a mixture of the methyl benzimidate hydrochloride (10 mmol) and corresponding amino acid methyl ester hydrochloride (10 mmol) in CH_2Cl_2 (150 mL) was added Et_3N (10 mmol). The reaction solution was stirred for 24 h at 25 °C. The insoluble salts were filtered off and the filtrate was washed with saturated $NaHCO_3$ aqueous solution, and dried over $MgSO_4$. The organic layer was concentrated under vacuum, and the crude product was chromatographed (petroleum ether/ethyl acetate, 5:1) on silica gel to obtain the products.

General procedure B: Synthesis of cyclic imino naphthyl esters

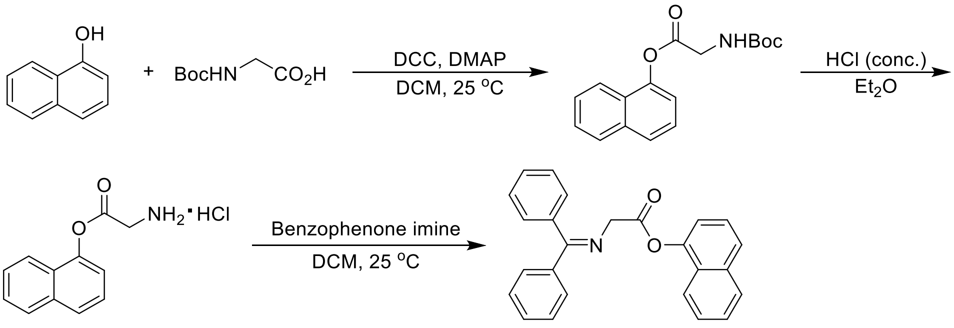

S1

To a round bottom flask was added 1-naphthalenol (30 mmol), BOC-*DL*-Glycine (30 mmol), DMAP (3 mmol), and DCM (120 mL), then DCC (33 mmol) was added in batches, the reaction was stirred overnight at 25 °C. The precipitated urea was filtered off, and the filtrate was washed with 5% NaOH (100 mL) and 5% HCl (100 mL). The organic phase was dried over $MgSO_4$, filtered and concentrated in vacuo. The residue was dissolved in diethyl ether (40 mL) and HCl (12 M, 48 mmol) was added. The reaction was stirred at 25 °C for 2 h. Then, the precipitate was filtered, and washed with diethyl ether to afford the HCl-salt of the glycine naphthyl ester. Under Ar atmosphere, a mixture of benzophenone imine (22 mmol), glycine naphthyl ester hydrochloride (22 mmol), and dry CH_2Cl_2 (60 mL) was stirred at 25 °C for 24 h. The solid was filtered off, and the filtrate was concentrated under reduced pressure. The residue was chromatographed (petroleum ether/ethyl acetate, 30:1) on silica gel to get **S1**.

1d-1dk were prepared according to a modified literature procedure.[4] **1d**, **1db**, and **1dd** were prepared according to method D. **1dc**, and **1de-1dj** were prepared according to method E. **1dk** was prepared according to method F.

Method D:

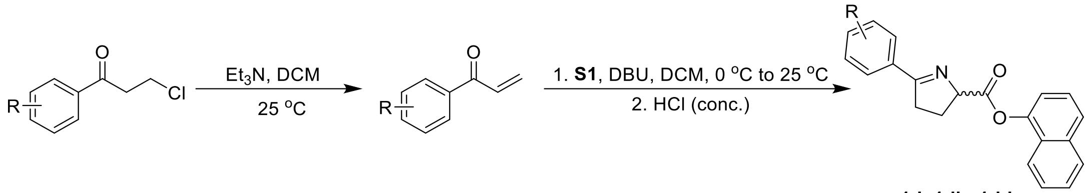

Method D was the same as method A, except for using **S1** instead of (*N*,*N*-diphenylmethylgene)glycine methyl ester.

Method E:

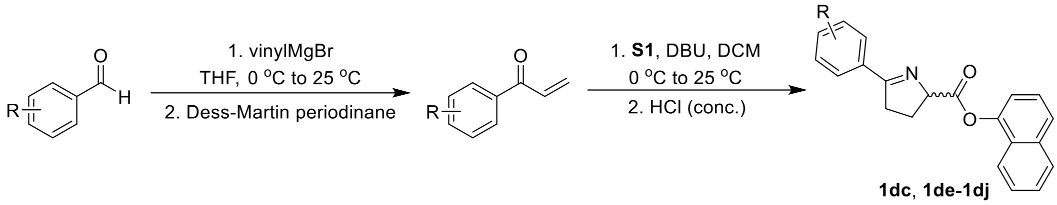

Method E was the same as method B, except for using **S1** instead of (*N*,*N*-diphenylmethylgene)glycine methyl ester.

Method F:

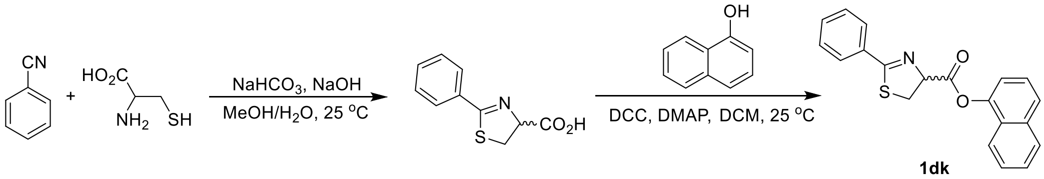

$NaHCO_3$ (15 mmol), NaOH (0.5 mmol), and *DL*-cysteine (15 mmol) were added to a stirred solution of benzonitrile (10 mmol) in methanol (26 mL) and H_2O (17 mL). The mixture was stirred at 25 °C until TLC analysis indicated the disappearance of the benzonitrile. Then, MeOH was removed from the mixture under reduced pressure. The residue was acidified with aq 2 M HCl to pH 2. The mixture was extracted with CH_2Cl_2 (30 mL × 3). The organic layers were combined, dried over $MgSO_4$, filtered and concentrated in vacuo to give 2-phenyl-4,5-dihydrothiazole-4-carboxylic acid. The crude residue was used directly for the next step without further purifications.

To a round bottom flask was added 1-naphthalenol (10 mmol), 2-phenyl-4,5-dihydrothiazole-4-carboxylic acid (10 mmol), DCC (11 mmol), DMAP (1 mmol), and CH_2Cl_2, the reaction was stirred overnight at 25 °C. The precipitated urea was filtered off, and the filtrate was concentrated in vacuo, and the crude product was chromatographed (petroleum ether/ethyl acetate, 5:1) on silica gel to obtain the product **1dk**.

General procedure C: Synthesis of aldimine Schiff bases

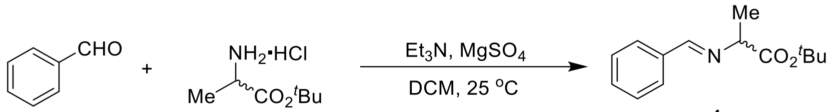

1e

1e was prepared according to a modified literature procedure.[5]

To a round bottom flask was added the *DL*-Alanine *tert*-butyl ester hydrochloride (12 mmol), benzaldehyde (10 mmol), $MgSO_4$ (16 mmol), and CH_2Cl_2 (30 mL), and the mixture was stirred at 0 °C. Then, Et_3N (14.0 mmol) was added dropwise. The reaction was allowed to warm to 25 °C and stirred for 12 h. Et_2O (40 mL) was then added and the solution was filtered. The filtrate was washed with water (2 × 30 mL), and brine (2 × 30 mL), dried over $MgSO_4$, filtered and concentrated in vacuo to give the product **1e**.

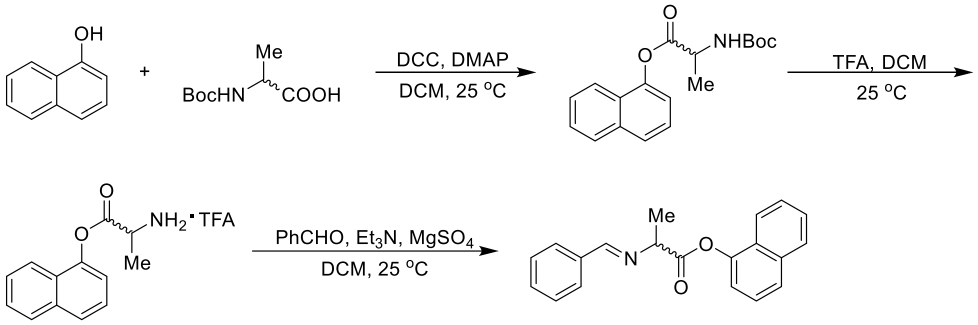

1f

To a round bottom flask was added 1-naphthalenol (30 mmol), BOC- *DL*-Alanine (30 mmol), DMAP (3 mmol), and CH_2Cl_2 (120 mL), then DCC (33 mmol) was added in batches, the reaction was stirred overnight at 25 °C. The precipitated urea was filtered off, and the filtrate was washed with 5% NaOH (100 mL) and 5% HCl (100 mL). The organic phase was dried over $MgSO_4$, filtered and concentrated in vacuo. The residue was dissolved in CH_2Cl_2 (40 mL) and TFA (240 mmol) was added. The reaction was stirred at 25 °C for 12 h. The solvent was removed under reduced pressure. The residue was dissolved in diethyl ether. Then, the precipitate was filtered, and washed with diethyl ether to afford the TFA-salt of the alanine naphthyl ester.

To a round bottom flask was added the TFA-salt of the alanine naphthyl ester (12 mmol), benzaldehyde (10 mmol), $MgSO_4$ (16 mmol), and CH_2Cl_2 (30 mL), and the mixture was stirred at 0 °C. Et_3N (14.0 mmol) was added dropwise. The reaction was allowed to warm to 25 °C and stirred for 12 h. Et_2O (40 mL) was then added and the solution was filtered. The filtrate was washed with water (2 × 30 mL), and brine (2 × 30 mL), dried over $MgSO_4$, filtered and concentrated in vacuo, and the crude product was chromatographed (petroleum ether/ethyl acetate, 20:1, + 1% Et_3N) on silica gel to obtain the products **1f**.

Hydroquinones and thiols

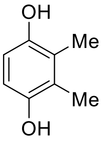
2a

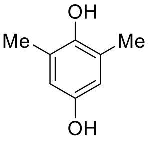
2b

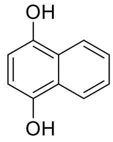
2c

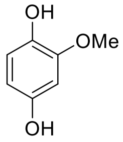
2d

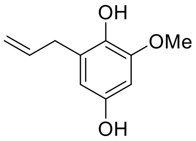
2e

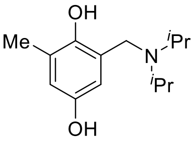
2f

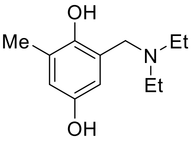
2g

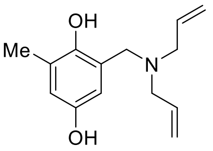
2h

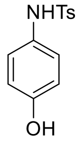
2i

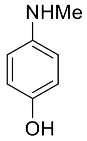
2j

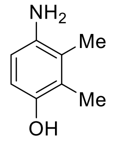
2k

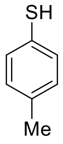
6a

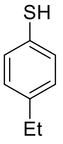
6b

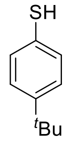
6c

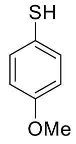
6d

2a-2d, **2i-2k** and **6a-6d** were purchased from commercial suppliers and used without further purification. **2e-2h** were prepared according to a literature procedure and the data was in agreement with those previously reported.[6,7]

Methyl 5-phenyl-3,4-dihydro-2H-pyrrole-2-carboxylate (1a)

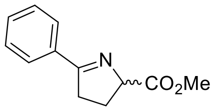

Pale yellow solid, 70% yield. **^{1}H NMR (500 MHz, CDCl$_3$)** δ 7.89 – 7.85 (m, 2H), 7.47 – 7.37 (m, 3H), 4.96 – 4.87 (m, 1H), 3.78 (s, 3H), 3.18 – 3.17 (m, 1H), 3.03 – 2.92 (m, 1H), 2.40 – 2.30 (m, 1H), 2.30 – 2.18 (m, 1H). **^{13}C NMR (151 MHz, CDCl$_3$)** δ 176.26, 173.51, 133.84, 131.11, 128.55, 128.15, 74.61, 52.41, 35.54, 26.44. **ESI-MS:** calculated [$C_{12}H_{13}NO_2$ + H]$^+$: 204.1019, found: 204.1018.

Methyl 5-(4-fluorophenyl)-3,4-dihydro-2H-pyrrole-2-carboxylate (1ab)

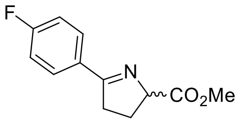

Yellow solid, 51% yield. **^{1}H NMR (500 MHz, CDCl$_3$)** δ 7.90 – 7.84 (m, 2H), 7.12 – 7.05 (m, 2H), 4.93 – 4.87 (m, 1H), 3.78 (s, 3H), 3.18 – 3.08 (m, 1H), 3.01 – 2.90 (m, 1H), 2.41 – 2.30 (m, 1H), 2.30 – 2.19 (m, 1H). **^{13}C NMR (126 MHz, CDCl$_3$)** δ 174.90, 173.34, 164.52 (d, J = 251.4 Hz), 130.18 (d, J = 8.8 Hz), 130.11, 115.51 (d, J = 21.7 Hz), 74.53, 52.32, 35.48, 26.46. **^{19}F NMR (471 MHz, CDCl$_3$)** δ -109.06. **ESI-MS:** calculated [$C_{12}H_{12}FNO_2$ + H]$^+$: 222.0925, found: 222.0923.

Methyl 5-(4-chlorophenyl)-3,4-dihydro-2H-pyrrole-2-carboxylate (1ac)

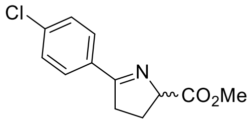

Yellow solid, 42% yield. **^{1}H NMR (500 MHz, CDCl$_3$)** δ 7.81 (d, *J* = 8.3 Hz, 2H), 7.38 (d, *J* = 8.3 Hz, 2H), 4.95 – 4.87 (m, 1H), 3.78 (s, 3H), 3.17 – 3.07 (m, 1H), 3.00 – 2.90 (m, 1H), 2.41 – 2.31 (m, 1H), 2.30 – 2.21 (m, 1H). **^{13}C NMR (126 MHz, CDCl$_3$)** δ 175.01, 173.23, 137.12, 132.27, 129.37, 128.73, 74.61, 52.34, 35.43, 26.41. **ESI-MS:** calculated [$C_{12}H_{12}ClNO_2$ + H]$^+$: 238.0629, found: 238.0627.

Methyl 5-(4-bromophenyl)-3,4-dihydro-2H-pyrrole-2-carboxylate (1ad)

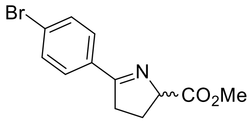

Pale yellow solid, 44% yield. **^{1}H NMR (500 MHz, CDCl$_3$)** δ 7.74 (d, *J* = 8.6 Hz, 2H), 7.55 (d, *J* = 8.5 Hz, 2H), 4.94 – 4.85 (m, 1H), 3.79 (s, 3H), 3.19 – 3.08 (m, 1H), 3.01 – 2.90 (m, 1H), 2.42 – 2.31 (m, 1H), 2.31 – 2.21 (m, 1H). **^{13}C NMR (126 MHz, CDCl$_3$)** δ 175.28, 173.32, 132.83, 131.85, 129.72, 125.76, 74.77, 52.49, 35.54, 26.53. **ESI-MS:** calculated [$C_{12}H_{12}BrNO_2$ + H]$^+$: 282.0124, found: 282.0124.

Methyl 5-(4-(trifluoromethyl)phenyl)-3,4-dihydro-2H-pyrrole-2-carboxylate (1ae)

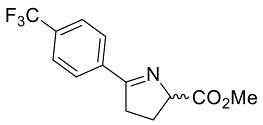

Pale yellow solid, 39% yield. **^{1}H NMR (500 MHz, CDCl$_3$)** δ 7.98 (d, *J* = 8.0 Hz, 2H), 7.66 (d, *J* = 8.0 Hz, 2H), 5.01 – 4.88 (m, 1H), 3.79 (s, 3H), 3.23 – 3.10 (m, 1H), 3.06 – 2.92 (m, 1H), 2.46 – 2.21 (m, 2H). **^{13}C NMR (126 MHz, CDCl$_3$)** δ 175.08, 173.14, 137.09, 132.70 (q, J = 32.4 Hz), 128.49, 123.99 (d, J = 272.2 Hz), 125.56 (q, J = 3.8 Hz), 74.88, 52.51, 35.67, 26.47. **ESI-MS:** calculated [$C_{13}H_{12}F_3NO_2$ + H]$^+$: 272.0893, found: 272.0891.

Methyl 5-(*p*-tolyl)-3,4-dihydro-2H-pyrrole-2-carboxylate (1af)

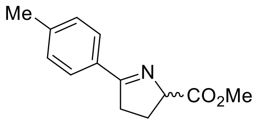

Yellow solid, 30% yield. **^{1}H NMR (500 MHz, CDCl$_3$)** δ 7.76 (d, *J* = 7.9 Hz, 2H), 7.20 (d, *J* = 7.9 Hz, 2H), 4.93 – 4.86 (m, 1H), 3.77 (s, 3H), 3.18 – 3.08 (m, 1H), 3.00 – 2.90 (m, 1H), 2.37 (s, 3H), 2.36 – 2.28 (m, 1H), 2.28 – 2.17 (m, 1H). **^{13}C NMR (126 MHz, CDCl$_3$)** δ 176.07, 173.62, 141.39, 131.23, 129.24, 128.12, 74.58, 52.35, 35.51, 26.46, 21.58. **ESI-MS:** calculated [$C_{13}H_{15}NO_2$ + H]$^+$: 218.1176, found: 218.1175.

Methyl 5-(4-methoxyphenyl)-3,4-dihydro-2H-pyrrole-2-carboxylate (1ag)

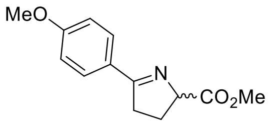

White solid, 42% yield.**^{1}H NMR (500 MHz, CDCl$_3$)** δ 7.83 (d, *J* = 8.8 Hz, 2H), 6.91 (d, *J* = 8.8 Hz, 2H), 4.91 – 4.85 (m, 1H), 3.84 (s, 3H), 3.77 (s, 3H), 3.18 – 3.07 (m, 1H), 3.00 – 2.89 (m, 1H), 2.39 – 2.28 (m, 1H), 2.28 – 2.17 (m, 1H). **^{13}C NMR (126 MHz, CDCl$_3$)** δ 175.51, 173.76, 161.97, 129.88, 126.76, 113.88, 74.51, 55.48, 52.38, 35.47, 26.57. **ESI-MS:** calculated [$C_{13}H_{15}NO_3$ + H]$^+$: 234.1125, found: 234.1123.

Methyl 5-(3-chlorophenyl)-3,4-dihydro-2H-pyrrole-2-carboxylate (1ah)

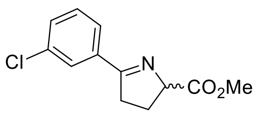

Brown oil, 44% yield. **^{1}H NMR (600 MHz, CDCl$_3$)** δ 7.89 (t, *J* = 1.9 Hz, 1H), 7.72 (dt, *J* = 7.9, 1.6 Hz, 1H), 7.42 (d, *J* = 8.1 Hz, 1H), 7.34 (tt, *J* = 8.0, 1.7 Hz, 1H), 4.95 – 4.89 (m, 1H), 3.78 (s, 3H), 3.17 – 3.09 (m, 1H), 3.00 – 2.91 (m, 1H), 2.41 – 2.32 (m, 1H), 2.31 – 2.20 (m, 1H). **^{13}C NMR (151 MHz, CDCl$_3$)** δ 175.11, 173.27,

135.58, 134.72, 131.09, 129.86, 128.19, 126.31, 74.69, 52.52, 35.57, 26.43. **ESI-MS:** calculated [$C_{12}H_{12}ClNO_2$ + H]$^+$: 238.0629, found: 238.0629.

Methyl 5-(2-chlorophenyl)-3,4-dihydro-2H-pyrrole-2-carboxylate (1ai)

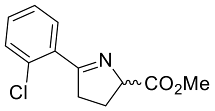

Brown oil, 39% yield. **^{1}H NMR (600 MHz, CDCl$_3$)** δ 7.64 – 7.60 (m, 1H), 7.39 – 7.35 (m, 1H), 7.34 – 7.29 (m, 1H), 7.28 – 7.24 (m, 1H), 4.91 – 4.82 (m, 1H), 3.77 (s, 3H), 3.26 – 3.16 (m, 1H), 3.11 – 3.02 (m, 1H), 2.42 – 2.31 (m, 1H), 2.29 – 2.18 (m, 1H). **^{13}C NMR (151 MHz, CDCl$_3$)** δ 177.32, 173.11, 134.43, 132.56, 130.97, 130.69, 130.24, 126.88, 73.98, 52.45, 38.87, 27.12. **ESI-MS:** calculated [$C_{12}H_{12}ClNO_2$ + H]$^+$: 238.0629, found: 238.0629.

Methyl 5-(naphthalen-2-yl)-3,4-dihydro-2H-pyrrole-2-carboxylate (1aj)

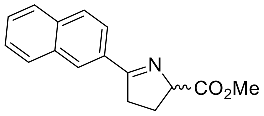

Yellow solid, 48% yield. **^{1}H NMR (500 MHz, CDCl$_3$)** δ 8.21 (d, *J* = 1.7 Hz, 1H), 8.11 (dd, *J* = 8.7, 1.8 Hz, 1H), 7.92 – 7.82 (m, 3H), 7.56 – 7.47 (m, 2H), 5.02 – 4.90 (m, 1H), 3.80 (s, 3H), 3.34 – 3.23 (m, 1H), 3.15 – 3.04 (m, 1H), 2.45 – 2.35 (m, 1H), 2.35 – 2.25 (m, 1H). **^{13}C NMR (126 MHz, CDCl$_3$)** δ 176.18, 173.52, 134.73, 132.97, 131.41, 128.90, 128.28, 127.87, 127.45, 126.57, 124.80, 74.77, 52.42, 35.57, 26.53. **ESI-MS:** calculated [$C_{16}H_{15}NO_2$ + H]$^+$: 254.1176, found: 254.1173.

Methyl 5-(thiophen-2-yl)-3,4-dihydro-2H-pyrrole-2-carboxylate (1ak)

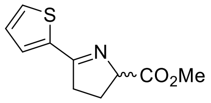

Pale yellow solid, 34% yield. **^{1}H NMR (500 MHz, CDCl$_3$)** δ 7.47 – 7.43 (m, 1H), 7.40 – 7.37 (m, 1H), 7.09 – 7.04 (m, 1H), 4.91 – 4.83 (m, 1H), 3.76 (s, 3H), 3.19 – 3.08 (m, 1H), 3.03 – 2.90 (m, 1H), 2.40 – 2.30 (m, 1H), 2.29 – 2.19 (m, 1H). **^{13}C NMR (126 MHz, CDCl$_3$)** δ 173.34, 170.57, 138.56, 130.35, 130.13, 127.62, 74.39, 52.39, 36.16, 26.88. **ESI-MS:** calculated [$C_{10}H_{11}NO_2S$ + H]$^+$: 210.0583, found: 210.0580.

Methyl 2-phenyl-4,5-dihydrothiazole-4-carboxylate (1al)

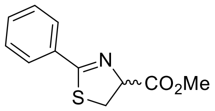

Colorless oil, 56% yield. **^{1}H NMR (500 MHz, CDCl$_3$)** δ 7.89 – 7.84 (m, 2H), 7.51 – 7.44 (m, 1H), 7.43 – 7.38 (m, 2H), 5.29 (t, *J* = 9.1 Hz, 1H), 3.83 (s, 3H), 3.72 (dd, *J* = 11.2, 8.9 Hz, 1H), 3.64 (dd, *J* = 11.2, 9.3 Hz, 1H). **^{13}C NMR (126 MHz, CDCl$_3$)** δ 171.37, 171.03, 132.68, 131.71, 128.64, 128.52, 78.50, 52.81, 35.38. **ESI-MS:** calculated [$C_{11}H_{11}NO_2S$ + H]$^+$: 222.0583, found: 222.0582.

Methyl 2-phenyl-4,5-dihydrooxazole-4-carboxylate (1ap)

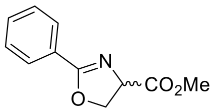

Colorless oil, 65% yield. **^{1}H NMR (500 MHz, CDCl$_3$)** δ 8.01 – 7.95 (m, 2H), 7.53 – 7.46 (m, 1H), 7.44 – 7.38 (m, 2H), 4.95 (dd, *J* = 10.6, 8.0 Hz, 1H), 4.69 (t, *J* = 8.3 Hz, 1H), 4.60 (dd, *J* = 10.6, 8.7 Hz, 1H), 3.82 (s, 3H). **^{13}C NMR (126 MHz, CDCl$_3$)** δ 171.79, 166.47, 132.02, 128.73, 128.50, 127.05, 69.69, 68.74, 52.87. **ESI-MS:** calculated [$C_{11}H_{11}NO_3$ + H]$^+$: 206.0812, found: 206.0817.

Naphthalen-1-yl 5-phenyl-3,4-dihydro-2H-pyrrole-2-carboxylate (1d)

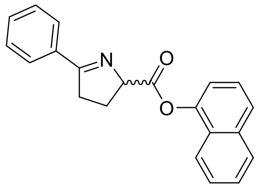

Pale yellow solid, 66% yield. **^{1}H NMR (500 MHz, $CDCl_3$)** δ 8.02 – 7.95 (m, 3H), 7.90 – 7.84 (m, 1H), 7.75 (d, *J* = 8.2 Hz, 1H), 7.53 – 7.43 (m, 6H), 7.32 (d, *J* = 7.5 Hz, 1H), 5.39 – 5.32 (m, 1H), 3.35 – 3.24 (m, 1H), 3.21 – 3.10 (m, 1H), 2.65 – 2.49 (m, 2H). **^{13}C NMR (126 MHz, $CDCl_3$)** δ 176.89, 171.51, 146.70, 134.77, 133.87, 131.30, 128.67, 128.27, 128.08, 126.89, 126.63, 126.56, 126.22, 125.48, 121.42, 118.15, 74.95, 35.82, 26.68. **ESI-MS:** calculated $[C_{21}H_{17}NO_2 + H]^+$: 316.1332, found: 316.1331.

Naphthalen-1-yl 5-(4-fluorophenyl)-3,4-dihydro-2H-pyrrole-2-carboxylate (1db)

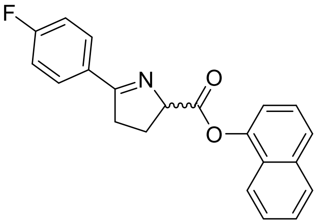

Yellow solid, 51% yield. **^{1}H NMR (500 MHz, $CDCl_3$)** δ 8.02 – 7.94 (m, 3H), 7.90 – 7.85 (m, 1H), 7.78 – 7.72 (m, 1H), 7.55 – 7.44 (m, 3H), 7.36 – 7.30 (m, 1H), 7.18 – 7.10 (m, 2H), 5.39 – 5.26 (m, 1H), 3.30 – 3.18 (m, 1H), 3.17 – 3.03 (m, 1H), 2.65 – 2.47 (m, 2H). **^{13}C NMR (126 MHz, $CDCl_3$)** δ 175.57, 171.45, 164.71 (d, *J* = 251.4 Hz), 146.63, 134.75, 130.37 (d, *J* = 8.7 Hz), 130.20 (d, *J* = 3.2 Hz), 128.10, 126.83, 126.59 (d, *J* = 6.2 Hz), 126.24, 125.47, 121.33, 118.12, 115.80, 115.63, 74.91, 35.79, 26.76. **^{19}F NMR (471 MHz, $CDCl_3$)** δ -108.71. **ESI-MS:** calculated $[C_{21}H_{16}FNO_2 + H]^+$: 334.1238, found: 334.1238.

Naphthalen-1-yl 5-(4-chlorophenyl)-3,4-dihydro-2H-pyrrole-2-carboxylate (1dc)

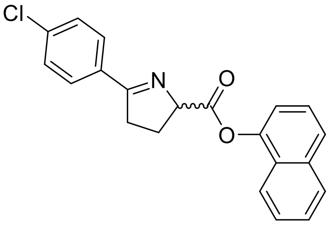

Yellow solid, 46% yield. **^{1}H NMR (500 MHz, $CDCl_3$)** δ 7.99 – 7.95 (m, 1H), 7.93 – 7.84 (m, 3H), 7.75 (d, *J* = 8.3 Hz, 1H), 7.54 – 7.39 (m, 5H), 7.38 – 7.30 (m, 1H), 5.38 – 5.30 (m, 1H), 3.30 – 3.20 (m, 1H), 3.16 – 3.04 (m, 1H), 2.65 – 2.49 (m, 2H). **^{13}C NMR (126 MHz, $CDCl_3$)** δ 175.72, 171.36, 146.64, 137.44, 134.78, 132.36, 129.58, 128.96, 128.13, 126.85, 126.65, 126.60, 126.28, 125.49, 121.34, 118.13, 75.01, 35.78, 26.74. **ESI-MS:** calculated $[C_{21}H_{16}ClNO_2 + H]^+$: 350.0942, found: 350.0941.

Naphthalen-1-yl 5-(4-bromophenyl)-3,4-dihydro-2H-pyrrole-2-carboxylate (1de)

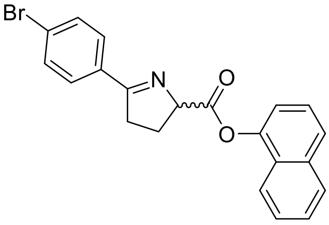

Pale yellow solid, 39% yield. **^{1}H NMR (500 MHz, $CDCl_3$)** δ 7.99 – 7.94 (m, 1H), 7.89 – 7.80 (m, 3H), 7.75 (d, *J* = 8.2 Hz, 1H), 7.62 – 7.56 (m, 2H), 7.53 – 7.44 (m, 3H), 7.34 – 7.29 (m, 1H), 5.36 – 5.28 (m, 1H), 3.30 – 3.20 (m, 1H), 3.16 – 3.05 (m, 1H), 2.65 – 2.49 (m, 2H). **^{13}C NMR (126 MHz, $CDCl_3$)** δ 175.72, 171.20, 146.53, 134.67, 132.67, 131.82, 129.67, 128.01, 126.73, 126.54, 126.49, 126.17, 125.82, 125.37, 121.22, 118.02, 74.93, 35.63, 26.62. **ESI-MS:** calculated $[C_{21}H_{16}BrNO_2 + H]^+$: 394.0437, found: 394.0432.

Naphthalen-1-yl 5-(*p*-tolyl)-3,4-dihydro-2H-pyrrole-2-carboxylate (1de)

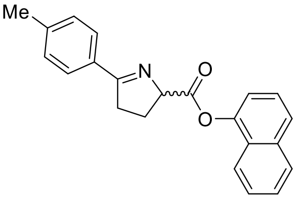

Brown solid, 28% yield. **^{1}H NMR (500 MHz, $CDCl_3$)** δ 8.02 – 7.96 (m, 1H), 7.89 – 7.83 (m, 3H), 7.73 (d, *J* = 8.2 Hz, 1H), 7.51 – 7.43 (m, 3H), 7.32 (d, *J* = 7.5 Hz, 1H), 7.25 (d, *J* = 7.7 Hz, 2H), 5.36 – 5.29 (m, 1H), 3.30 – 3.20 (m, 1H), 3.16 – 3.06 (m, 1H), 2.61 – 2.46 (m, 2H), 2.40 (s, 3H). **^{13}C NMR (126 MHz, $CDCl_3$)** δ 176.69, 171.61, 146.71, 141.61,

134.75, 131.21, 129.36, 128.24, 128.05, 126.89, 126.59, 126.53, 126.17, 125.47, 121.44, 118.14, 74.88, 35.75, 26.66, 21.63. **ESI-MS:** calculated [$C_{22}H_{19}NO_2$ + H]$^+$: 330.1489, found: 330.1504.

Naphthalen-1-yl 5-(4-methoxyphenyl)-3,4-dihydro-2H-pyrrole-2-carboxylate (1df)

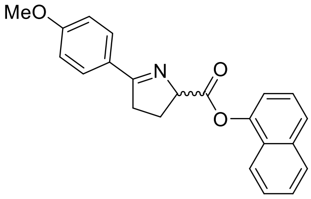

Brown solid, 41% yield. **^{1}H NMR (500 MHz, CDCl$_3$)** δ 8.02 – 7.97 (m, 1H), 7.93 (d, *J* = 8.4 Hz, 2H), 7.89 – 7.84 (m, 1H), 7.74 (d, *J* = 8.2 Hz, 1H), 7.53 – 7.43 (m, 3H), 7.32 (d, *J* = 7.5 Hz, 1H), 6.96 (d, *J* = 8.4 Hz, 2H), 5.35 – 5.29 (m, 1H), 3.86 (s, 3H), 3.30 – 3.20 (m, 1H), 3.16 – 3.06 (m, 1H), 2.62 – 2.47 (m, 2H). **^{13}C NMR (126 MHz, CDCl$_3$)** δ 176.10, 171.72, 162.09, 146.72, 134.75, 129.97, 128.06, 126.90, 126.71, 126.59, 126.54, 126.17, 125.48, 121.44, 118.15, 113.96, 74.78, 55.49, 35.68, 26.75. **ESI-MS:** calculated [$C_{22}H_{19}NO_3$ + H]$^+$: 346.1438, found: 346.1436.

Naphthalen-1-yl 5-(3-chlorophenyl)-3,4-dihydro-2H-pyrrole-2-carboxylate (1dg)

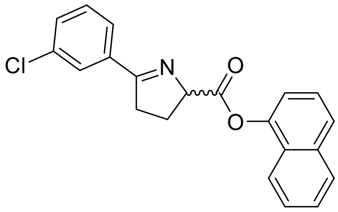

Brown solid, 36% yield. **^{1}H NMR (600 MHz, CDCl$_3$)** δ 8.00 – 7.96 (m, 2H), 7.89 – 7.86 (m, 1H), 7.83 (d, *J* = 7.8 Hz, 1H), 7.75 (d, *J* = 8.2 Hz, 1H), 7.54 – 7.44 (m, 4H), 7.41 – 7.37 (m, 1H), 7.34 – 7.30 (m, 1H), 5.40 – 5.31 (m, 1H), 3.30 – 3.21 (m, 1H), 3.16 – 3.05 (m, 1H), 2.65 – 2.50 (m, 2H). **^{13}C NMR (151 MHz, CDCl$_3$)** δ 175.80, 171.30, 146.59, 135.50, 134.81, 134.75, 131.29, 129.98, 128.34, 128.12, 126.81, 126.69, 126.62, 126.41, 126.31, 125.49, 121.32, 118.15, 74.91, 35.84, 26.65. **ESI-MS:** calculated [$C_{21}H_{16}ClNO_2$ + H]$^+$: 350.0942, found: 350.0941.

Naphthalen-1-yl 5-(*m*-tolyl)-3,4-dihydro-2H-pyrrole-2-carboxylate (1dh)

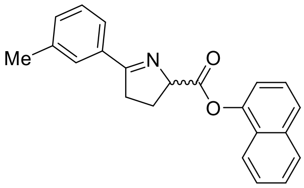

Brown oil, 32% yield. **^{1}H NMR (600 MHz, CDCl$_3$)** δ 8.01 – 7.97 (m, 1H), 7.89 – 7.83 (m, 2H), 7.77 – 7.70 (m, 2H), 7.52 – 7.44 (m, 3H), 7.36 – 7.29 (m, 3H), 5.39 – 5.31 (m, 1H), 3.33 – 3.24 (m, 1H), 3.20 – 3.08 (m, 1H), 2.67 – 2.49 (m, 2H), 2.41 (s, 3H). **^{13}C NMR (151 MHz, CDCl$_3$)** δ 177.20, 171.60, 146.63, 138.41, 134.72, 133.69, 132.14, 128.76, 128.55, 128.08, 126.83, 126.62, 126.57, 126.23, 125.53, 125.49, 121.40, 118.17, 74.80, 35.85, 26.69, 21.48. **ESI-MS:** calculated [$C_{22}H_{19}NO_2$ + H]$^+$: 330.1489, found: 330.1488.

Naphthalen-1-yl 5-(naphthalen-2-yl)-3,4-dihydro-2H-pyrrole-2-carboxylate (1di)

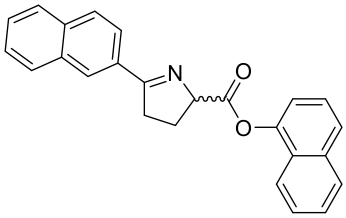

White solid, 50% yield. **^{1}H NMR (500 MHz, CDCl$_3$)** δ 8.32 – 8.22 (m, 2H), 8.07 – 8.02 (m, 1H), 7.96 – 7.85 (m, 4H), 7.76 (d, *J* = 8.2 Hz, 1H), 7.60 – 7.46 (m, 5H), 7.36 (d, *J* = 7.5 Hz, 1H), 5.46 – 5.35 (m, 1H), 3.47 – 3.33 (m, 1H), 3.29 – 3.19 (m, 1H), 2.70 – 2.53 (m, 2H). **^{13}C NMR (126 MHz, CDCl$_3$)** δ 176.77, 171.53, 146.69, 134.82, 134.75, 133.00, 131.40, 129.11, 128.95, 128.41, 128.07, 127.92, 127.56, 126.88, 126.65, 126.62, 126.56, 126.21, 125.48, 124.80, 121.41, 118.15, 75.07, 35.79, 26.72. **ESI-MS:** calculated [$C_{25}H_{19}NO_2$ + H]$^+$: 366.1489, found: 366.1488.

Naphthalen-1-yl 5-(thiophen-2-yl)-3,4-dihydro-2H-pyrrole-2-carboxylate (1dj)

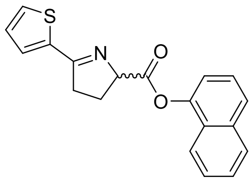

Brown oil, 32% yield. **^{1}H NMR (600 MHz, $CDCl_3$)** δ 8.01 – 7.97 (m, 1H), 7.88 – 7.84 (m, 1H), 7.77 – 7.72 (m, 1H), 7.54 – 7.43 (m, 5H), 7.33 – 7.29 (m, 1H), 7.13 – 7.09 (m, 1H), 5.34 – 5.29 (m, 1H), 3.32 – 3.23 (m, 1H), 3.18 – 3.10 (m, 1H), 2.60 – 2.53 (m, 2H). **^{13}C NMR (151 MHz, $CDCl_3$)** δ 171.34, 171.25, 146.62, 138.53, 134.73, 130.65, 130.47, 128.04, 127.75, 126.84, 126.66, 126.58, 126.23, 125.46, 121.46, 118.14, 74.71, 36.38, 26.99. **ESI-MS:** calculated $[C_{19}H_{15}NO_2S + H]^+$: 322.0896, found: 322.0895.

Naphthalen-1-yl 5-(naphthalen-2-yl)-3,4-dihydro-2H-pyrrole-2-carboxylate (1dk)

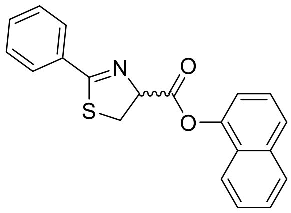

Brown solid, 28% yield. **^{1}H NMR (500 MHz, $CDCl_3$)** δ 8.01 – 7.96 (m, 3H), 7.91 – 7.85 (m, 1H), 7.77 (d, *J* = 8.2 Hz, 1H), 7.56 – 7.44 (m, 6H), 7.35 (d, *J* = 7.5 Hz, 1H), 5.73 (dd, *J* = 9.4, 8.2 Hz, 1H), 3.99 (dd, *J* = 11.2, 8.2 Hz, 1H), 3.85 (dd, *J* = 11.3, 9.4 Hz, 1H). **^{13}C NMR (126 MHz, $CDCl_3$)** δ 171.86, 169.60, 146.61, 134.79, 132.78, 132.01, 128.86, 128.75, 128.13, 126.77, 126.70, 126.67, 126.48, 125.46, 121.32, 118.08, 78.77, 35.62. **ESI-MS:** calculated $[C_{20}H_{15}NO_2S + H]^+$: 334.0896, found: 334.0897.

***tert*-butyl (*E*)-2-(benzylideneamino)propanoate (1e)**

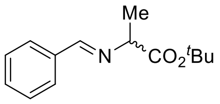

Colorless oil, 81% yield. **^{1}H NMR (600 MHz, $CDCl_3$)** δ 8.30 (s, 1H), 7.80 – 7.74 (m, 2H), 7.44 – 7.38 (m, 3H), 4.04 (q, *J* = 7.2 Hz, 1H), 1.49 (d, *J* = 7.1 Hz, 3H), 1.47 (s, 9H). **^{13}C NMR (126 MHz, $CDCl_3$)** δ 171.90, 162.64, 135.98, 131.03, 128.63, 128.52, 81.19, 68.60, 28.10, 19.52. **ESI-MS:** calculated $[C_{14}H_{19}NO_2 + H]^+$: 234.1489, found: 234.1488.

Naphthalen-1-yl (*E*)-2-(benzylideneamino)propanoate (1f)

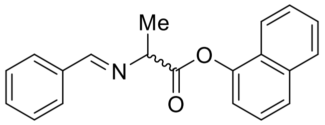

Colorless oil, 50% yield. **^{1}H NMR (500 MHz, CD_3COCD_3)** δ 8.67 (s, 1H), 8.02 – 7.92 (m, 4H), 7.85 – 7.81 (m, 1H), 7.56 – 7.48 (m, 6H), 7.33 (dd, *J* = 7.5, 1.1 Hz, 1H), 4.70 (q, *J* = 6.7 Hz, 1H), 1.69 (d, *J* = 6.7 Hz, 3H). **^{13}C NMR (151 MHz, CD_3COCD_3)** δ 170.85, 163.99, 146.99, 136.35, 134.83, 131.33, 128.92, 128.85, 128.54, 128.13, 127.74, 126.66, 126.09, 125.68, 121.38, 118.42, 67.77, 18.80. **ESI-MS:** calculated $[C_{20}H_{17}NO_2 + H]^+$: 304.1332, found: 304.1331.

2.2 Photographic Guide for Electrochemical Reactions

2.2.1 Overview of materials used

From left to right: 1) Electrochemical cell. 2) Carbon rod electrode (d = 6 mm) (Purchased from Tianjin AIDAhengsheng Science-Technology Development Co., Ltd.).

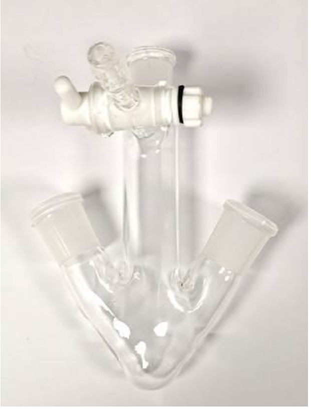

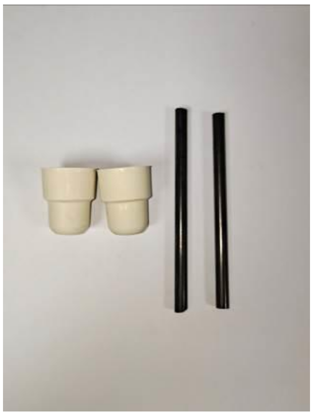

2.2.2 Assembling the cell

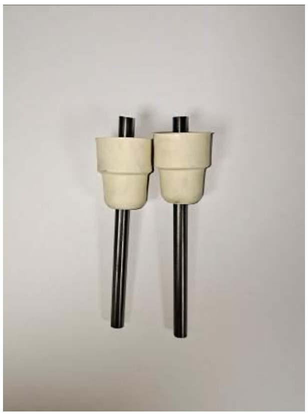

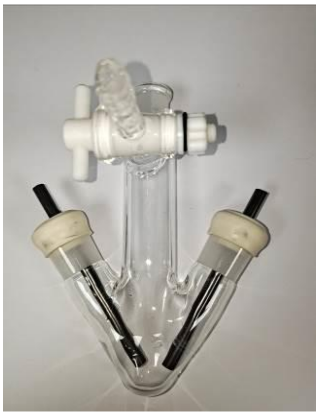

2.2.3 Electrolysis

The potentiostat was purchased from Shanghai Xin Rui Instrument Co., Ltd.

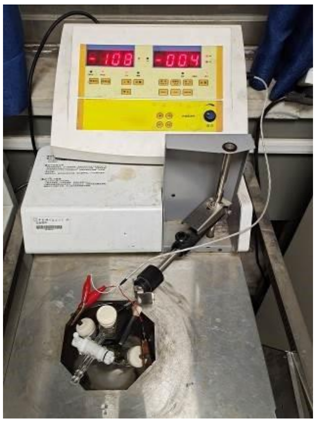

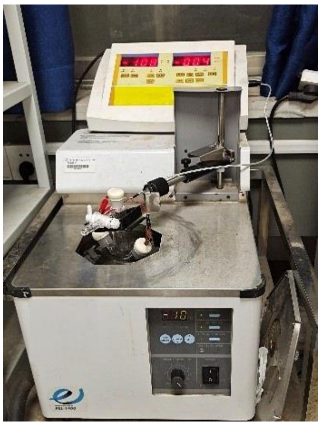

2.3 Synthesis and Characterization of Products

General procedure D: Synthesis of product 3

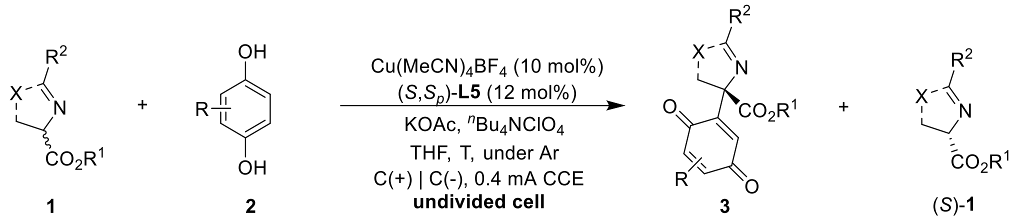

In a Schlenk tube equipped with two carbon electrodes, $Cu(MeCN)_4BF_4$ (0.015 mmol, 10 mol%) and (*S*, *S*$_p$)-**L5** (0.018 mmol 12 mol%) were stirred in 2 mL anhydrous THF under argon at 25 ^oC for 10 min. **1** (0.15 mmol), **2** (0.225 mmol), $^nBu_4NClO_4$ (0.28 mmol), KOAc (0.3 mmol), and THF (2 mL) were added successively under argon. The constant current (I = 0.4 mA) electrolysis was carried out at -10 °C to 5 ^oC until complete consumption of the substrate (monitored by TLC, 8-12 h). The solvent was removed under reduced pressure. The residue was purified by silica gel chromatography to afford the desired product **3**.

The kinetic resolution can be achieved at -30 ^oC to 10 ^oC to afford desired product **3** and (*S*)-**1**.

General procedure E: Synthesis of product 4

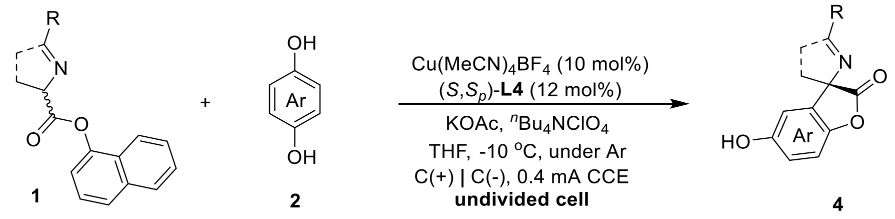

In a Schlenk tube equipped with two carbon electrodes, $Cu(MeCN)_4BF_4$ (0.015 mmol, 10 mol%) and (*S*,*S*$_p$)-**L4** (0.018 mmol, 12 mol%) were stirred in 2 mL anhydrous THF under argon at 25 ^oC for 10 min. **1** (0.15 mmol), **2** (0.225 mmol), $^nBu_4NClO_4$ (0.28 mmol), KOAc (0.3 mmol), and THF (2 mL) were added successively under argon. The constant current (I = 0.4 mA) electrolysis was carried out at -10 °C until complete consumption of the substrate (monitored by TLC, 6-8 h). The solvent was removed under reduced pressure. The residue was purified by silica gel chromatography to afford the desired product **4**.

General procedure F: Synthesis of product 5

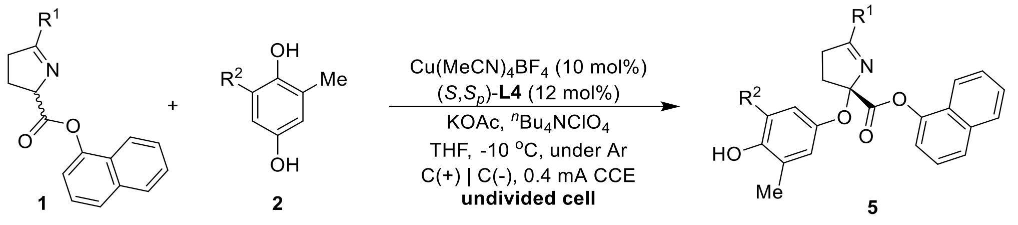

In a Schlenk tube equipped with two carbon electrodes, $Cu(MeCN)_4BF_4$ (0.015 mmol, 10 mol%) and (*S*,*S*$_p$)-**L4** (0.018 mmol, 12 mol%) were stirred in 2 mL anhydrous THF under argon at 25 ^oC for 10 min. **1** (0.15 mmol), **2** (0.225 mmol), $^nBu_4NClO_4$ (0.28 mmol), KOAc (0.3 mmol), and THF (2 mL) were added successively under argon. The constant current (I = 0.4 mA) electrolysis was carried out at -10 °C until complete consumption of the substrate (monitored by TLC, 8-12 h). The solvent was removed under reduced pressure. The residue was purified by silica gel chromatography to afford the desired product **5**.

General procedure G: Synthesis of product 7

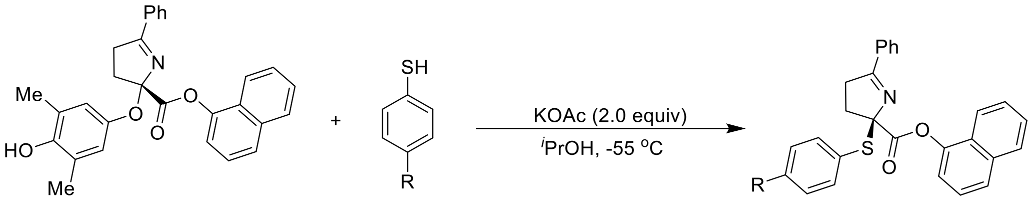

5a, 92% e.e. **6** **7**

To a flask was added **5a** (0.24 mmol), **6** (0.2 mmol), and KOAc (0.4 mmol) in iPrOH (4 mL), and the reaction was stirred overnight at -55 °C. The solvent was removed under reduced pressure. The residue was purified by silica gel chromatography to afford the desired product **7**.

Methyl (*S*)-2-(4,5-dimethyl-3,6-dioxocyclohexa-1,4-dien-1-yl)-5-phenyl-3,4-dihydro-2H-pyrrole-2-carboxylate (3a)

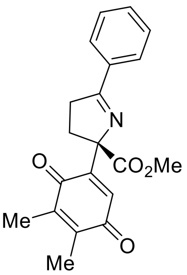

The title compound was prepared according to the general procedure D using **1a** (0.15 mmol) and **2a** (0.225 mmol) at 10 °C. The crude reaction mixture was purified by flash column chromatography (petroleum ether/ethyl acetate, 5:1) to afford the title compound as a yellow oil (71%).**^{1}H NMR (500 MHz, $CDCl_3$)** δ 7.94 – 7.90 (m, 2H), 7.51 – 7.46 (m, 1H), 7.44 – 7.40 (m, 2H), 6.78 (s, 1H), 3.69 (s, 3H), 3.39 – 3.30 (m, 1H), 3.25 – 3.18 (m, 1H), 3.12 – 3.03 (m, 1H), 2.04 (s, 3H), 2.03 (s, 3H), 1.88 – 1.80 (m, 1H). **^{13}C NMR (126 MHz, $CDCl_3$)** δ 187.78, 187.07, 178.21, 171.31, 149.90, 141.31, 140.99, 133.48, 131.67, 130.89, 128.65, 128.43, 82.95, 53.09, 36.56, 33.32, 12.32, 12.27. **ESI-MS:** calculated $[C_{20}H_{19}NO_4 + H]^+$: 338.1387, found: 338.1387. $[\alpha]^{20}_D$ = -342.4 (c = 2.16, CH_2Cl_2). The product was analyzed by HPLC to determine the enantiomeric excess: 92% e.e. (CHIRALPAK AD-H, hexane/*i*-PrOH = 80/20, detector: 254 nm, T = 25 °C, flow rate: 1 mL/min), t_1(minor) = 7.48 min, t_2(major) = 8.37 min.

Methyl (*S*)-5-phenyl-3,4-dihydro-2H-pyrrole-2-carboxylate (1a)

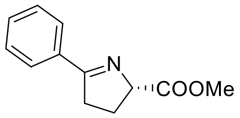

The title compound was prepared according to the general procedure D using **1a** (0.15 mmol) and **2a** (0.225 mmol) at -10 °C. The crude reaction mixture was purified by flash column chromatography (petroleum ether/ethyl acetate, 5:1) to afford **3a** (57%, 92% e.e.) and (*S*)-**1a** (30%, 98% e.e.). $[\alpha]^{20}_D$ = +56.5 (c = 0.59, CH_2Cl_2). The product was analyzed by HPLC to determine the enantiomeric excess: 98% e.e. (CHIRALPAK AD-H, hexane/*i*-PrOH = 80/20, detector: 254 nm, T = 25 °C, flow rate: 1 mL/min), t_1(minor) = 5.26 min, t_2(major) = 6.16 min.

Methyl (*S*)-2-(4,5-dimethyl-3,6-dioxocyclohexa-1,4-dien-1-yl)-5-(4-fluorophenyl)-3,4-dihydro-2H-pyrrole-2-carboxylate (3b)

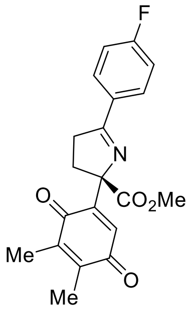

The title compound was prepared according to the general procedure D using **1ab** (0.15 mmol) and **2a** (0.225 mmol) at 0 °C. The crude reaction mixture was purified by flash column chromatography (petroleum ether/ethyl acetate, 5:1) to afford the title compound as a yellow oil (67%). **^{1}H NMR (600 MHz, $CDCl_3$)** δ 7.94 – 7.90 (m, 2H), 7.13 – 7.08 (m, 2H), 6.76 (s, 1H), 3.69 (s, 3H), 3.35 – 3.28 (m, 1H), 3.24 – 3.18 (m, 1H), 3.09 – 3.02 (m, 1H), 2.04 (s, 3H), 2.03 (s, 3H), 1.88 – 1.79 (m, 1H). **^{13}C NMR (151 MHz, $CDCl_3$)** δ 187.79, 187.05, 176.99, 171.29, 164.95 (d, *J* = 252.2 Hz), 149.78, 141.35, 141.02, 130.84, 130.64 (d, *J* = 8.7 Hz), 129.76 (d, *J* = 2.8 Hz), 115.76 (d, *J* = 21.8 Hz), 82.92, 53.16, 36.57, 33.40, 12.35, 12.29. **^{19}F NMR (565 MHz, $CDCl_3$)** δ -107.77. **ESI-MS:** calculated $[C_{20}H_{18}FNO_4 + H]^+$: 356.1293, found: 356.1295. $[\alpha]^{20}_D$ = -284.0 (c = 0.60, CH_2Cl_2). The product was analyzed by HPLC to determine the enantiomeric excess: 93% e.e. (CHIRALPAK AD-H, hexane/*i*-PrOH = 80/20, detector: 254 nm, T = 25 °C, flow rate: 1 mL/min), t_1(minor) = 6.61 min, t_2(major) = 9.38 min.

Methyl (*S*)-5-(4-chlorophenyl)-2-(4,5-dimethyl-3,6-dioxocyclohexa-1,4-dien-1-yl)-3,4-dihydro-2H-pyrrole-2-carboxylate (3c)

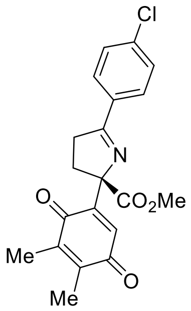

The title compound was prepared according to the general procedure D using **1ac** (0.15 mmol) and **2a** (0.225 mmol) at -10 °C. The crude reaction mixture was purified by flash column chromatography (petroleum ether/ethyl acetate, 5:1) to afford the title compound as a yellow oil (67%). **^{1}H NMR (600 MHz, $CDCl_3$)** δ 7.87 – 7.81 (m, 2H), 7.43 – 7.36 (m, 2H), 6.74 (s, 1H), 3.69 (s, 3H), 3.35 – 3.27 (m, 1H), 3.25 – 3.18 (m, 1H), 3.08 – 3.01 (m, 1H), 2.04 (s, 3H), 2.03 (s, 3H), 1.88 – 1.81 (m, 1H). **^{13}C NMR (151 MHz, $CDCl_3$)** δ 187.76, 187.01, 177.15, 171.20, 149.68, 141.37, 141.02, 137.87, 131.87, 130.84, 129.74, 128.94, 83.00, 53.19, 36.53, 33.36, 12.35, 12.30. **ESI-MS:** calculated $[C_{20}H_{18}ClNO_4 + H]^+$: 372.0997, found: 372.0997. $[\alpha]^{20}_D$ = -306.1 (c = 1.16, CH_2Cl_2). The product was analyzed by HPLC to determine the enantiomeric excess: 92% e.e. (CHIRALPAK AD-H, hexane/*i*-PrOH = 80/20, detector: 254 nm, T = 25 °C, flow rate: 1 mL/min), t_1(minor) = 6.89 min, t_2(major) = 10.05 min.

Methyl (*S*)-5-(4-chlorophenyl)-3,4-dihydro-2H-pyrrole-2-carboxylate (1ac)

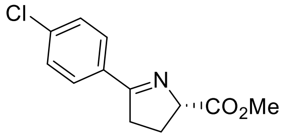

The title compound was prepared according to the general procedure D using **1ac** (0.15 mmol) and **2a** (0.225 mmol) at -25 °C. The crude reaction mixture was purified by flash column chromatography (petroleum ether/ethyl acetate, 5:1) to afford **3c** (51%, 93% e.e.) and (*S*)-**1ac** (33%, 99% e.e.). $[\alpha]^{20}_D$ = +8.5 (c = 0.24, CH_2Cl_2). The product was analyzed by HPLC to determine the enantiomeric excess: 99% e.e. (CHIRALPAK AD-H, hexane/*i*-PrOH = 80/20, detector: 254 nm, T = 25 °C, flow rate: 1 mL/min), t_1(minor) = 5.94 min, t_2(major) = 6.90 min.

Methyl (*S*)-5-(4-bromophenyl)-2-(4,5-dimethyl-3,6-dioxocyclohexa-1,4-dien-1-yl)-3,4-dihydro-2H-pyrrole-2-carboxylate (3d)

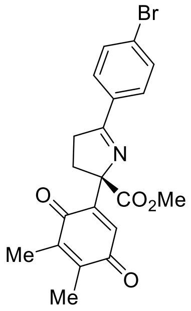

The title compound was prepared according to the general procedure D using **1ad** (0.15 mmol) and **2a** (0.225 mmol) at 0 °C. The crude reaction mixture was purified by flash column chromatography (petroleum ether/ethyl acetate, 5:1) to afford the title compound as a yellow oil (78%). **^{1}H NMR (600 MHz, $CDCl_3$)** δ 7.79 – 7.77 (m, 2H), 7.58 – 7.55 (m, 2H), 6.74 (s, 1H), 3.69 (s, 3H), 3.34 – 3.27 (m, 1H), 3.24 – 3.19 (m, 1H), 3.08 – 3.01 (m, 1H), 2.04 (s, 3H), 2.03 (s, 3H), 1.88 – 1.81 (m, 1H). **^{13}C NMR (151 MHz, $CDCl_3$)** δ 187.76, 187.01, 177.28, 171.17, 149.65, 141.38, 141.03, 132.29, 131.92, 130.85, 129.93, 126.42, 83.04, 53.21, 36.51, 33.36, 12.36, 12.31. **ESI-MS:** calculated $[C_{20}H_{18}BrNO_4 + H]^+$: 416.0492, found: 416.0489. $[\alpha]^{20}_{D}$ = -292.6 (c = 0.26, CH_2Cl_2). The product was analyzed by HPLC to determine the enantiomeric excess: 92% e.e. (CHIRALPAK AD-H, hexane/*i*-PrOH = 80/20, detector: 254 nm, T = 25 °C, flow rate: 1 mL/min), t_1(minor) = 7.32 min, t_2(major) = 10.77 min.

Methyl (*S*)-5-(4-bromophenyl)-3,4-dihydro-2H-pyrrole-2-carboxylate (1ad)

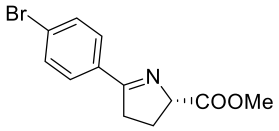

The title compound was prepared according to the general procedure D using **1ad** (0.15 mmol) and **2a** (0.225 mmol) at -30 °C. The crude reaction mixture was purified by flash column chromatography (petroleum ether/ethyl acetate, 5:1) to afford **3d** (49%, 95% e.e.) and (*S*)-**1ad** (43%, 82% e.e.). $[\alpha]^{20}_{D}$ = +52.3 (c = 0.35, CH_2Cl_2). The product was analyzed by HPLC to determine the enantiomeric excess: 82% e.e. (CHIRALPAK AD-H, hexane/*i*-PrOH = 80/20, detector: 254 nm, T = 25 °C, flow rate: 1 mL/min), t_1(minor) = 6.22 min, t_2(major) = 7.90 min.

Methyl (*S*)-2-(4,5-dimethyl-3,6-dioxocyclohexa-1,4-dien-1-yl)-5-(4-(trifluoromethyl)phenyl)-3,4-dihydro-2H-pyrrole-2-carboxylate (3e)

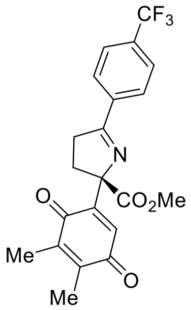

The title compound was prepared according to the general procedure D using **1ae** (0.15 mmol) and **2a** (0.225 mmol) at -10 °C. The crude reaction mixture was purified by flash column chromatography (petroleum ether/ethyl acetate, 5:1) to afford the title compound as a yellow oil (74%). **^{1}H NMR (600 MHz, $CDCl_3$)** δ 8.02 (d, *J* = 8.0 Hz, 2H), 7.68 (d, *J* = 7.9 Hz, 2H), 6.74 (s, 1H), 3.69 (s, 3H), 3.40 – 3.31 (m, 1H), 3.27 – 3.20 (m, 1H), 3.14 – 3.06 (m, 1H), 2.03 (s, 3H), 2.02 (s, 3H), 1.91 – 1.83 (m, 1H). **^{13}C NMR (151 MHz, $CDCl_3$)** δ 187.66, 186.92, 177.12, 171.02, 149.47, 141.39, 141.01, 136.51, 133.14 (q, *J* = 32.6 Hz), 130.82, 128.74, 125.62 (q, *J* = 3.7 Hz), 123.86 (q, *J* = 272.4 Hz), 83.18, 53.22, 36.64, 33.31, 12.31, 12.26. **^{19}F NMR (565 MHz, $CDCl_3$)** δ -62.82. **ESI-MS:** calculated $[C_{21}H_{18}F_3NO_4 + Na]^+$: 428.1080, found: 428.1078. $[\alpha]^{20}_{D}$ = -268.2 (c = 0.61, CH_2Cl_2). The product was analyzed by HPLC to determine the enantiomeric excess: 92% e.e. (CHIRALPAK AD-H, hexane/*i*-PrOH = 80/20, detector: 254 nm, T = 25 °C, flow rate: 1 mL/min), t_1(minor) = 5.81 min, t_2(major) = 8.81 min.

Methyl (*S*)-5-(4-(trifluoromethyl)phenyl)-3,4-dihydro-2H-pyrrole-2-carboxylate (1ae)

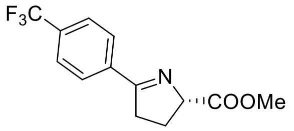

The title compound was prepared according to the general procedure D using **1ae** (0.15 mmol) and **2a** (0.225 mmol) at -25 °C. The crude reaction mixture was purified by flash column chromatography (petroleum ether/ethyl acetate, 5:1) to afford **3e** (53%, 93% e.e.) and (*S*)-**1ae** (30%, 93% e.e.). $[\alpha]^{20}_{D}$ = -41.0 (c = 0.81, CH_2Cl_2). The product was analyzed by HPLC to determine the enantiomeric excess: 93% e.e. (CHIRALPAK AD-H, hexane/*i*-PrOH = 80/20, detector: 254 nm, T = 25 °C, flow rate: 1 mL/min), t_1(minor) = 5.48 min, t_2(major) = 6.64 min.

Methyl (*S*)-2-(4,5-dimethyl-3,6-dioxocyclohexa-1,4-dien-1-yl)-5-(p-tolyl)-3,4-dihydro-2H-pyrrole-2-carboxylate (3f)

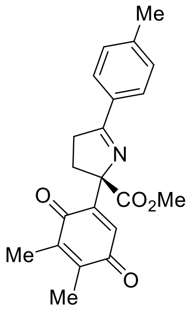

The title compound was prepared according to the general procedure D using **1af** (0.15 mmol) and **2a** (0.225 mmol) at 10 °C. The crude reaction mixture was purified by flash column chromatography (petroleum ether/ethyl acetate, 5:1) to afford the title compound as a yellow oil (65%). **^{1}H NMR (600 MHz, CDCl$_3$)** δ 7.83 – 7.77 (m, 2H), 7.24 – 7.19 (m, 2H), 6.78 (s, 1H), 3.68 (s, 3H), 3.36 – 3.27 (m, 1H), 3.22 – 3.16 (m, 1H), 3.09 – 3.02 (m, 1H), 2.39 (s, 3H), 2.04 (s, 3H), 2.03 (s, 3H), 1.86 – 1.78 (m, 1H). **^{13}C NMR (151 MHz, CDCl$_3$)** δ 187.85, 187.13, 178.12, 171.44, 149.99, 142.15, 141.31, 140.99, 130.86, 130.77, 129.35, 128.42, 82.81, 53.11, 36.52, 33.27, 21.69, 12.34, 12.29. **ESI-MS:** calculated $[C_{21}H_{21}NO_4 + H]^+$: 352.1543, found: 352.1547. $[\alpha]^{20}_{D}$ = -322.8 (c = 0.36, CH_2Cl_2). The product was analyzed by HPLC to determine the enantiomeric excess: 86% e.e. (CHIRALPAK AD-H, hexane/*i*-PrOH = 80/20, detector: 254 nm, T = 25 °C, flow rate: 1 mL/min), t_1(minor) = 6.33 min, t_2(major) = 7.66 min.

Methyl (*S*)-2-(4,5-dimethyl-3,6-dioxocyclohexa-1,4-dien-1-yl)-5-(4-methoxyphenyl)-3,4-dihydro-2H-pyrrole-2-carboxylate (3g)

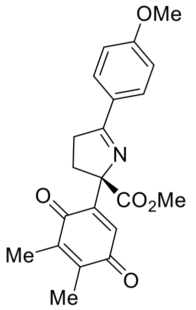

The title compound was prepared according to the general procedure D using **1ag** (0.15 mmol) and **2a** (0.225 mmol) at 5 °C. The crude reaction mixture was purified by flash column chromatography (petroleum ether/ethyl acetate, 4:1) to afford the title compound as a yellow oil (63%). **^{1}H NMR (500 MHz, CDCl$_3$)** δ 7.89 – 7.80 (m, 2H), 6.94 – 6.87 (m, 2H), 6.78 (s, 1H), 3.85 (s, 3H), 3.68 (s, 3H), 3.34 – 3.25 (m, 1H), 3.22 – 3.14 (m, 1H), 3.08 – 2.98 (m, 1H), 2.03 (s, 3H), 2.02 (s, 3H), 1.86 – 1.76 (m, 1H). **^{13}C NMR (126 MHz, CDCl$_3$)** δ 187.85, 187.14, 177.39, 171.51, 162.38, 150.11, 141.27, 140.98, 130.85, 130.20, 126.28, 113.92, 82.72, 55.53, 53.06, 36.42, 33.35, 12.33, 12.27. **ESI-MS:** calculated $[C_{21}H_{21}NO_5 + H]^+$: 368.1492, found: 368.1490. $[\alpha]^{20}_{D}$ = -327.3 (c = 0.56, CH_2Cl_2). The product was analyzed by HPLC to determine the enantiomeric excess: 92% e.e. (CHIRALPAK AD-H, hexane/*i*-PrOH = 80/20, detector: 254 nm, T = 25 °C, flow rate: 1 mL/min), t_1(minor) = 8.91 min, t_2(major) = 12.87 min.

Methyl (*S*)-5-(4-methoxyphenyl)-3,4-dihydro-2H-pyrrole-2-carboxylate (1ag)

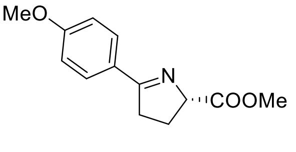

The title compound was prepared according to the general procedure D using **1ag** (0.15 mmol) and **2a** (0.225 mmol) at -10 °C. The crude reaction mixture was purified by flash column chromatography (petroleum ether/ethyl acetate, 4:1) to afford **3g** (49%, 92% e.e.) and (*S*)-**1ag** (31%, 94% e.e.). $[\alpha]^{20}_{D}$ = +10.7 (c = 0.25, CH_2Cl_2). The product was analyzed by HPLC to determine the enantiomeric excess: 94% e.e. (CHIRALPAK AD-H, hexane/*i*-PrOH = 80/20, detector: 254 nm, T = 25 °C, flow rate: 1 mL/min), t_1(minor) = 7.37 min, t_2(major) = 10.04 min.

Methyl (*S*)-5-(3-chlorophenyl)-2-(4,5-dimethyl-3,6-dioxocyclohexa-1,4-dien-1-yl)-3,4-dihydro-2H-pyrrole-2-carboxylate (3h)

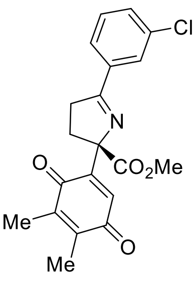

The title compound was prepared according to the general procedure D using **1ah** (0.15 mmol) and **2a** (0.225 mmol) at -10 °C. The crude reaction mixture was purified by flash column chromatography (petroleum ether/ethyl acetate, 5:1) to afford the title compound as a yellow oil (68%). **^{1}H NMR (600 MHz, $CDCl_3$)** δ 7.96 – 7.93 (m, 1H), 7.78 – 7.74 (m, 1H), 7.48 – 7.44 (m, 1H), 7.39 – 7.34 (m, 1H), 6.75 (s, 1H), 3.70 (s, 3H), 3.36 – 3.28 (m, 1H), 3.26 – 3.19 (m, 1H), 3.09 – 3.01 (m, 1H), 2.04 (s, 3H), 2.03 (s, 3H), 1.89 – 1.81 (m, 1H). **^{13}C NMR (151 MHz, $CDCl_3$)** δ 187.74, 186.99, 177.10, 171.12, 149.61, 141.40, 141.03, 135.13, 134.86, 131.66, 130.88, 129.97, 128.39, 126.60, 83.05, 53.23, 36.59, 33.33, 12.36, 12.31. **ESI-MS:** calculated $[C_{20}H_{18}ClNO_4 + H]^+$: 372.0997, found: 372.0996. $[\alpha]^{20}_{D}$ = -293.0 (c = 0.93, CH_2Cl_2). The product was analyzed by HPLC to determine the enantiomeric excess: 94% e.e. (CHIRALPAK AD-H, hexane/*i*-PrOH = 80/20, detector: 254 nm, T = 25 °C, flow rate: 1 mL/min), t_1(minor) = 7.18 min, t_2(major) = 10.41 min.

Methyl (*S*)-5-(3-chlorophenyl)-3,4-dihydro-2H-pyrrole-2-carboxylate (1ah)

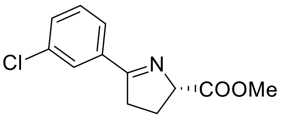

The title compound was prepared according to the general procedure D using **1ah** (0.15 mmol) and **2a** (0.225 mmol) at -25 °C. The crude reaction mixture was purified by flash column chromatography (petroleum ether/ethyl acetate, 5:1) to afford **3h** (49%, 95% e.e.) and (*S*)-**1ah** (31%, 94% e.e.). $[\alpha]^{20}_{D}$ = +13.1 (c = 0.33, CH_2Cl_2). The product was analyzed by HPLC to determine the enantiomeric excess: 94% e.e. (CHIRALPAK AD-H, hexane/*i*-PrOH = 80/20, detector: 254 nm, T = 25 °C, flow rate: 1 mL/min), t_1(minor) = 4.88 min, t_2(major) = 5.73 min.

Methyl (*S*)-5-(2-chlorophenyl)-2-(4,5-dimethyl-3,6-dioxocyclohexa-1,4-dien-1-yl)-3,4-dihydro-2H-pyrrole-2-carboxylate (3i)

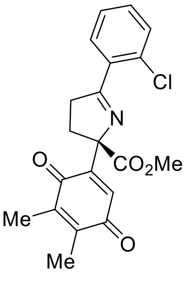

The title compound was prepared according to the general procedure D using **1ai** (0.15 mmol) and **2a** (0.225 mmol) at -10 °C. The crude reaction mixture was purified by flash column chromatography (petroleum ether/ethyl acetate, 5:1) to afford the title compound as a yellow oil (80%). **^{1}H NMR (500 MHz, $CDCl_3$)** δ 7.68 – 7.63 (m, 1H), 7.44 – 7.40 (m, 1H), 7.38 – 7.34 (m, 1H), 7.32 – 7.28 (m, 1H), 6.83 (s, 1H), 3.70 (s, 3H), 3.50 – 3.41 (m, 1H), 3.26 – 3.19 (m, 1H), 3.18 – 3.10 (m, 1H), 2.04 (s, 6H), 1.93 – 1.84 (m, 1H). **^{13}C NMR (151 MHz, $CDCl_3$)** δ 187.71, 187.00, 179.65, 170.96, 149.33, 141.42, 140.98, 133.90, 132.67, 131.40, 130.96, 130.57, 127.00, 82.70, 53.21, 40.16, 33.74, 12.34, 12.32. **ESI-MS:** calculated $[C_{20}H_{18}ClNO_4 + H]^+$: 372.0997, found: 372.0998. $[\alpha]^{20}_{D}$ = -

198.3 (c = 0.43, CH_2Cl_2). The product was analyzed by HPLC to determine the enantiomeric excess: 93% e.e. (CHIRALPAK AD-H, hexane/*i*-PrOH = 80/20, detector: 254 nm, T = 25 °C, flow rate: 1 mL/min), t_1(minor) = 6.37 min, t_2(major) = 7.37 min.

Methyl (*S*)-2-(4,5-dimethyl-3,6-dioxocyclohexa-1,4-dien-1-yl)-5-(naphthalen-2-yl)-3,4-dihydro-2H-pyrrole-2-carboxylate (3j)

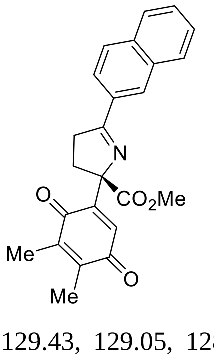

The title compound was prepared according to the general procedure D using **1aj** (0.15 mmol) and **2a** (0.225 mmol) at 0 °C. The crude reaction mixture was purified by flash column chromatography (petroleum ether/ethyl acetate, 5:1) to afford the title compound as a yellow solid (81%) . **^{1}H NMR (600 MHz, $CDCl_3$)** δ 8.27 – 8.25 (m, 1H), 8.16 – 8.13 (m, 1H), 7.92 – 7.90 (m, 1H), 7.89 – 7.85 (m, 2H), 7.58 – 7.50 (m, 2H), 6.85 (s, 1H), 3.72 (s, 3H), 3.52 – 3.44 (m, 1H), 3.31 – 3.18 (m, 2H), 2.06 (s, 3H), 2.04 (s, 3H), 1.93 – 1.87 (m, 1H). **^{13}C NMR (151 MHz, $CDCl_3$)** δ 187.86, 187.13, 178.27, 171.38, 149.93, 141.37, 141.04, 134.99, 132.92, 130.96, 130.93, 129.43, 129.05, 128.43, 127.96, 127.80, 126.77, 124.83, 83.05, 53.20, 36.63, 33.42, 12.39, 12.33. **ESI-MS:** calculated $[C_{24}H_{21}NO_4 + H]^+$: 388.1543, found: 388.1544. $[\alpha]^{20}_D$ = -347.3 (c = 0.91, CH_2Cl_2). The product was analyzed by HPLC to determine the enantiomeric excess: 93% e.e. (CHIRALPAK AD-H, hexane/*i*-PrOH = 80/20, detector: 254 nm, T = 25 °C, flow rate: 1 mL/min), t_1(minor) = 11.06 min, t_2(major) = 14.30 min.

Methyl (*S*)-5-(naphthalen-2-yl)-3,4-dihydro-2H-pyrrole-2-carboxylate (1aj)

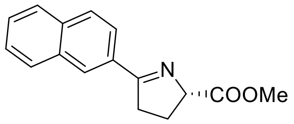

The title compound was prepared according to the general procedure D using **1aj** (0.15 mmol) and **2a** (0.225 mmol) at -10 °C. The crude reaction mixture was purified by flash column chromatography (petroleum ether/ethyl acetate, 5:1) to afford **3j** (48%, 94% e.e.) and (*S*)-**1aj** (25%, 96% e.e.). $[\alpha]^{20}_D$ = +37.0 (c = 0.78, CH_2Cl_2). The product was analyzed by HPLC to determine the enantiomeric excess: 96% e.e. (CHIRALPAK AD-H, hexane/*i*-PrOH = 80/20, detector: 254 nm, T = 25 °C, flow rate: 1 mL/min), t_1(minor) = 7.18 min, t_2(major) = 10.79 min.

Methyl (*S*)-2-(4,5-dimethyl-3,6-dioxocyclohexa-1,4-dien-1-yl)-5-(thiophen-2-yl)-3,4-dihydro-2H-pyrrole-2-carboxylate (3k)

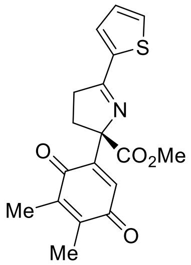

The title compound was prepared according to the general procedure D using **1ak** (0.15 mmol) and **2a** (0.225 mmol) at 10 °C. The crude reaction mixture was purified by flash column chromatography (petroleum ether/ethyl acetate, 5:1) to afford the title compound as a yellow oil (61%). **^{1}H NMR (500 MHz, $CDCl_3$)** δ 7.52 – 7.48 (m, 1H), 7.46 – 7.42 (m, 1H), 7.12 – 7.06 (m, 1H), 6.79 (s, 1H), 3.68 (s, 3H), 3.38 – 3.27 (m, 1H), 3.25 – 3.17 (m, 1H), 3.12 – 3.02 (m, 1H), 2.03 (s, 6H), 1.89 – 1.79 (m, 1H). **^{13}C NMR (126 MHz, $CDCl_3$)** δ 187.76, 187.08, 172.29, 171.21, 149.70, 141.35, 140.97, 138.07, 131.19, 131.02, 130.97, 127.76, 82.77, 53.11, 37.16, 33.67, 12.32, 12.28. **ESI-MS:** calculated $[C_{18}H_{17}NO_4S + H]^+$: 344.0951, found: 344.0952. $[\alpha]^{20}_D$ = -269.3 (c = 0.32, CH_2Cl_2). The product was analyzed by HPLC to determine the enantiomeric excess: 88% e.e. (CHIRALPAK AD-H, hexane/*i*-PrOH = 80/20, detector: 254 nm, T = 25 °C, flow rate: 1 mL/min), t_1(minor) = 9.91 min, t_2(major) = 10.86 min.

Methyl (*S*)-4-(4,5-dimethyl-3,6-dioxocyclohexa-1,4-dien-1-yl)-2-phenyl-4,5-dihydrothiazole-4-carboxylate (3l)

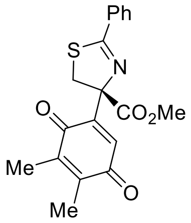

The title compound was prepared according to the general procedure D using **1al** (0.15 mmol) and **2a** (0.225 mmol) at 0 °C. The crude reaction mixture was purified by flash column chromatography (petroleum ether/ethyl acetate, 6:1) to afford the title compound as a yellow oil (68%). **^{1}H NMR (500 MHz, CDCl$_3$)** δ 7.92 – 7.86 (m, 2H), 7.54 – 7.49 (m, 1H), 7.45 – 7.40 (m, 2H), 6.72 (s, 1H), 4.58 (d, *J* = 12.0 Hz, 1H), 3.77 (s, 3H), 3.29 (d, *J* = 12.0 Hz, 1H), 2.04 (s, 3H), 2.04 (s, 3H). **^{13}C NMR (126 MHz, CDCl$_3$)** δ 187.36, 186.98, 173.77, 169.52, 146.94, 141.78, 141.14, 132.35, 132.31, 128.90, 128.74, 87.03, 53.62, 40.34, 12.34. **ESI-MS:** calculated [$C_{19}H_{17}NO_4S$ + H]$^+$: 356.0951, found: 356.0949. $[\alpha]^{20}_D$ = -435.0 (c = 0.25, CH_2Cl_2). The product was analyzed by HPLC to determine the enantiomeric excess: 92% e.e. (CHIRALPAK AD-H, hexane/*i*-PrOH = 80/20, detector: 254 nm, T = 25 °C, flow rate: 1 mL/min), t_1(minor) = 7.67 min, t_2(major) = 8.76 min.

***tert*-butyl (S)-2-amino-2-(4,5-dimethyl-3,6-dioxocyclohexa-1,4-dien-1-yl)propanoate (3m)**

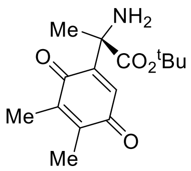

The title compound was prepared according to the general procedure D using **1e** (0.15 mmol) and **2a** (0.225 mmol) at 10 °C. After the reaction was complete, aqueous HCl (4 mL, 1 mol/L) was carefully added to the reaction mixture at 25 °C and the mixture was stirred for 3 h. The mixture was then neutralized with K_2CO_3 and extracted with DCM. The combined extracts were concentrated in vacuo. The crude reaction mixture was purified by flash column chromatography (petroleum ether/ethyl acetate, 2:1) to afford the title compound as a yellow oil (51%). **^{1}H NMR (600 MHz, CDCl$_3$)** δ 6.72 (s, 1H), 2.02 (s, 3H), 2.01 (s, 3H), 1.98 – 1.75 (br, 2H), 1.47 (s, 3H), 1.39 (s, 9H). **^{13}C NMR (151 MHz, CDCl$_3$)** δ 188.06, 187.43, 174.70, 149.97, 141.30, 141.00, 130.60, 81.88, 58.29, 27.87, 24.19, 12.39, 12.20. **ESI-MS:** calculated [$C_{15}H_{21}NO_4$ + H]$^+$: 280.1543, found: 280.1540. $[\alpha]^{20}_D$ = +15.3 (c = 0.20, CH_2Cl_2). The product was analyzed by HPLC to determine the enantiomeric excess: 90% e.e. (CHIRALPAK IA, hexane/*i*-PrOH = 90/10, detector: 254 nm, T = 25 °C, flow rate: 1 mL/min), t_1(minor) = 6.99 min, t_2(major) = 10.57 min.

Methyl (*S*)-2-(4-methoxy-3,6-dioxocyclohexa-1,4-dien-1-yl)-5-phenyl-3,4-dihydro-2H-pyrrole-2-carboxylate (3n)

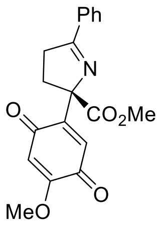

The title compound was prepared according to the general procedure D using **1a** (0.15 mmol) and **2d** (0.225 mmol) at 10 °C. The crude reaction mixture was purified by flash column chromatography (petroleum ether/ethyl acetate, 3:1) to afford the title compound as a yellow oil (90%). **^{1}H NMR (500 MHz, CDCl$_3$)** δ 7.93 – 7.89 (m, 2H), 7.51 – 7.47 (m, 1H), 7.45 – 7.40 (m, 2H), 6.79 (s, 1H), 5.95 (s, 1H), 3.84 (s, 3H), 3.70 (s, 3H), 3.40 – 3.31 (m, 1H), 3.25 – 3.18 (m, 1H), 3.13 – 3.05 (m, 1H), 1.92 – 1.83 (m, 1H). **^{13}C NMR (126 MHz, CDCl$_3$)** δ 186.99, 182.38, 178.50, 171.12, 158.94, 151.18, 133.36, 131.76, 129.16, 128.68, 128.41, 107.80, 82.86, 56.49, 53.16, 36.64, 33.45. **ESI-MS:** calculated [$C_{19}H_{17}NO_5$ + H]$^+$: 340.1179, found: 340.1179. $[\alpha]^{20}_D$ = -292.6 (c = 0.47, CH_2Cl_2). The product was analyzed by HPLC to determine the enantiomeric excess: 89% e.e. (CHIRALPAK AD-H, hexane/*i*-PrOH = 80/20, detector: 254 nm, T = 25 °C, flow rate: 1 mL/min), t_1(major) = 12.77 min, t_2(minor) = 14.90 min.

Methyl (*S*)-2-(1,4-dioxo-1,4-dihydronaphthalen-2-yl)-5-phenyl-3,4-dihydro-2H-pyrrole-2-carboxylate (3o)

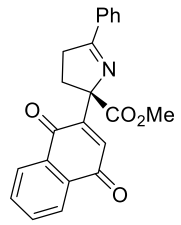

The title compound was prepared according to the general procedure D using **1a** (0.15 mmol) and **2c** (0.225 mmol) at 10 °C. The crude reaction mixture was purified by flash column chromatography (petroleum ether/ethyl acetate, 5:1) to afford the title compound as a yellow oil (80%). **^{1}H NMR (600 MHz, CDCl$_3$)** δ 8.12 – 8.06 (m, 2H), 7.98 – 7.94 (m, 2H), 7.78 – 7.74 (m, 2H), 7.52 – 7.49 (m, 1H), 7.47 – 7.43 (m, 2H), 7.09 (s, 1H), 3.71 (s, 3H), 3.44 – 3.37 (m, 1H), 3.36 – 3.30 (m, 1H), 3.17 – 3.10 (m, 1H), 1.98 – 1.91 (m, 1H). **^{13}C NMR (151 MHz, CDCl$_3$)** δ 185.40, 184.85, 178.48, 171.27, 152.48, 134.22, 133.95, 133.44, 133.41, 132.19, 131.77, 128.70, 128.48, 126.75, 126.41, 83.19, 53.21, 36.68, 33.39. **ESI-MS:** calculated [$C_{22}H_{17}NO_4$ + H]$^+$: 360.1230, found: 360.1230. $[\alpha]^{20}_D$ = -270.3 (c = 0.42, CH_2Cl_2). The product was analyzed by HPLC to determine the enantiomeric excess: 94% e.e. (CHIRALPAK IG, hexane/*i*-PrOH = 80/20, detector: 254 nm, T = 25 ℃, flow rate: 1 mL/min), t_1(minor) = 18.58 min, t_2(major) = 21.24 min.

Methyl (*R*)-4-(4,5-dimethyl-3,6-dioxocyclohexa-1,4-dien-1-yl)-2-phenyl-4,5-dihydrooxazole-4-carboxylate (3p)

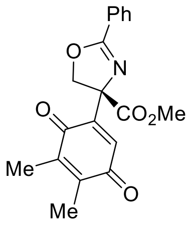

The title compound was prepared according to the general procedure D using **1ap** (0.15 mmol) and **2a** (0.225 mmol) at 10 °C. The crude reaction mixture was purified by flash column chromatography (petroleum ether/ethyl acetate, 5:1) to afford the title compound as a yellow oil (40%). **^{1}H NMR (500 MHz, CDCl$_3$)** δ 8.04 – 7.97 (m, 2H), 7.56 – 7.50 (m, 1H), 7.46 – 7.40 (m, 2H), 6.94 (s, 1H), 5.44 (d, *J* = 9.4 Hz, 1H), 4.15 (d, *J* = 9.4 Hz, 1H), 3.74 (s, 3H), 2.04 (s, 3H), 2.04 (s, 3H). **^{13}C NMR (126 MHz, CDCl$_3$)** δ 187.43, 187.33, 170.15, 167.34, 148.31, 142.01, 140.73, 132.49, 128.98, 128.59, 126.64, 77.21, 75.83, 53.60, 12.39, 12.23. **ESI-MS:** calculated [$C_{19}H_{17}NO_5$ + Na]$^+$: 362.0999, found: 362.0996. $[\alpha]^{20}_D$ = -243.3 (c = 0.26, CH_2Cl_2). The product was analyzed by HPLC to determine the enantiomeric excess: 90% e.e. (CHIRALPAK IA, hexane/i-PrOH = 95/5, detector: 254 nm, T = 25 ℃, flow rate: 1 mL/min), t_1(minor) = 12.79 min, t_2(major) = 13.43 min.

Methyl (*S*)-2-phenyl-4,5-dihydrooxazole-4-carboxylate (1ap)

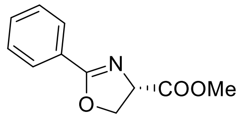

The title compound was prepared according to the general procedure D using **1ap** (0.15 mmol) and **2a** (0.225 mmol) at 10 °C. The crude reaction mixture was purified by flash column chromatography (petroleum ether/ethyl acetate, 5:1) to afford **3p** (40%, 90% e.e.) and (*S*)-**1ap** (48%, 83% e.e.). $[\alpha]^{20}_D$ = -0.6 (c = 0.98, CH_2Cl_2). The product was analyzed by HPLC to determine the enantiomeric excess: 83% e.e. (CHIRALPAK AD-H, hexane/*i*-PrOH = 80/20, detector: 254 nm, T = 25 ℃, flow rate: 1 mL/min), t_1(minor) = 5.35 min, t_2(major) = 6.34 min.

(*S*)-5-hydroxy-6,7-dimethyl-5'-phenyl-3',4'-dihydro-2H-spiro[benzofuran-3,2'-pyrrol]-2-one (4a)

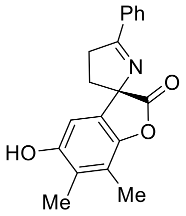

The title compound was prepared according to the general procedure E using **1d** (0.15 mmol) and **2a** (0.225 mmol). The crude reaction mixture was purified by flash column chromatography (petroleum ether/ethyl acetate, 3:1) to afford the title compound as a colorless oil (80%). **^{1}H NMR (600 MHz, $CDCl_3$)** δ 8.67 (s, 1H), 7.92 – 7.88 (m, 2H), 7.58 – 7.53 (m, 1H), 7.51 – 7.46 (m, 2H), 6.00 (s, 1H), 3.42 – 3.34 (m, 1H), 3.24 – 3.16 (m, 1H), 2.63 – 2.55 (m, 1H), 2.18 – 2.13 (m, 1H), 2.16 (s, 3H), 1.98 (s, 3H). **^{13}C NMR (151 MHz, $CDCl_3$)** δ 179.20, 176.95, 151.01, 145.53, 132.45, 132.31, 129.12, 128.64, 127.64, 124.17, 120.37, 109.82, 80.54, 36.53, 33.53, 12.45, 11.99. **ESI-MS:** calculated $[C_{19}H_{17}NO_3 + H]^+$: 308.1281, found: 308.1291. $[\alpha]^{20}_D$ = -236.6 (c = 0.45, CH_2Cl_2). The product was analyzed by HPLC to determine the enantiomeric excess: 95% e.e. (CHIRALPAK IA, hexane/*i*-PrOH = 90/10, detector: 254 nm, T = 25 °C, flow rate: 1 mL/min), t_1(minor) = 12.85 min, t_2(major) = 16.66 min.

(*S*)-5'-(4-fluorophenyl)-5-hydroxy-6,7-dimethyl-3',4'-dihydro-2H-spiro[benzofuran-3,2'-pyrrol]-2-one (4b)

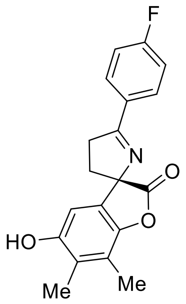

The title compound was prepared according to the general procedure E using **1db** (0.15 mmol) and **2a** (0.225 mmol). The crude reaction mixture was purified by flash column chromatography (petroleum ether/ethyl acetate, 3:1) to afford the title compound as a colorless oil (78%). **^{1}H NMR (500 MHz, $CDCl_3$)** δ 7.96 – 7.89 (m, 2H), 7.19 – 7.12 (m, 2H), 6.09 (s, 1H), 3.47 – 3.37 (m, 1H), 3.32 – 3.23 (m, 1H), 2.67 – 2.59 (m, 1H), 2.23 – 2.13 (m, 1H), 2.17 (s, 3H), 2.00 (s, 3H). **^{13}C NMR (126 MHz, $CDCl_3$)** δ 178.04, 176.90, 165.23 (d, *J* = 254.1 Hz), 150.94, 145.77, 130.93 (d, *J* = 9.0 Hz), 128.86 (d, *J* = 3.2 Hz), 127.54, 124.53, 120.66, 116.25 (d, *J* = 21.8 Hz), 109.64, 80.67, 36.64, 33.82, 12.46, 12.04. **^{19}F NMR (471 MHz, $CDCl_3$)** δ -106.22. **ESI-MS:** calculated $[C_{19}H_{16}FNO_3 + H]^+$: 326.1187, found: 326.1189. $[\alpha]^{20}_D$ = -212.1 (c = 0.40, CH_2Cl_2). The product was analyzed by HPLC to determine the enantiomeric excess: 94% e.e. (CHIRALPAK IA, hexane/*i*-PrOH = 80/20, detector: 254 nm, T = 25 °C, flow rate: 1 mL/min), t_1(minor) = 6.80 min, t_2(major) = 8.06 min.

(*S*)-5'-(4-chlorophenyl)-5-hydroxy-6,7-dimethyl-3',4'-dihydro-2H-spiro[benzofuran-3,2'-pyrrol]-2-one (4c)

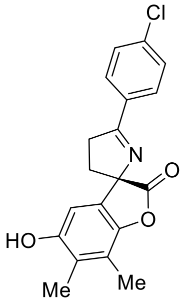

The title compound was prepared according to the general procedure E using **1dc** (0.15 mmol) and **2a** (0.225 mmol). The crude reaction mixture was purified by flash column chromatography (petroleum ether/ethyl acetate, 3:1) to afford the title compound as a colorless oil (80%). **^{1}H NMR (500 MHz, $CDCl_3$)** δ 7.87 – 7.81 (m, 2H), 7.46 – 7.40 (m, 2H), 6.12 (s, 1H), 3.46 – 3.37 (m, 1H), 3.34 – 3.24 (m, 1H), 2.69 – 2.60 (m, 1H), 2.24 – 2.19 (m, 1H), 2.18 (s, 3H), 2.02 (s, 3H). **^{13}C NMR (151 MHz, $CDCl_3$)** δ 178.36, 176.76, 150.91, 145.76, 138.69, 130.86, 129.91, 129.39, 127.78, 124.23, 120.61, 109.79, 80.70, 36.59, 33.71, 12.47, 12.04. **ESI-MS:** calculated $[C_{19}H_{16}ClNO_3 + H]^+$: 342.0891, found: 342.0890. $[\alpha]^{20}_D$ = -298.3 (c = 0.39, CH_2Cl_2). The product was analyzed by HPLC to determine the enantiomeric excess: 95% e.e. (CHIRALPAK IA, hexane/*i*-PrOH = 80/20, detector: 254 nm, T = 25 °C, flow rate: 1 mL/min), t_1(minor) = 7.35 min, t_2(major) = 8.21 min.

(*S*)-5'-(4-bromophenyl)-5-hydroxy-6,7-dimethyl-3',4'-dihydro-2H-spiro[benzofuran-3,2'-pyrrol]-2-one (4d)

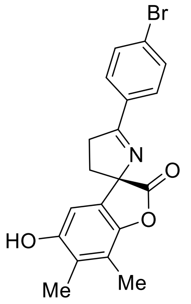

The title compound was prepared according to the general procedure E using **1dd** (0.15 mmol) and **2a** (0.225 mmol). The crude reaction mixture was purified by flash column chromatography (petroleum ether/ethyl acetate, 3:1) to afford the title compound as a colorless oil (83%). **^{1}H NMR (600 MHz, $CDCl_3$)** δ 8.27 (s, 1H), 7.78 – 7.75 (m, 2H), 7.62 – 7.58 (m, 2H), 6.04 (s, 1H), 3.45 – 3.36 (m, 1H), 3.31 – 3.23 (m, 1H), 2.66 – 2.59 (m, 1H), 2.22 – 2.17 (m, 1H), 2.16 (s, 3H), 1.98 (s, 3H). **^{13}C NMR (151 MHz, $CDCl_3$)** δ 178.49, 176.71, 150.93, 145.73, 132.37, 131.28, 130.04, 127.77, 127.23, 124.21, 120.60, 109.75, 80.73, 36.54, 33.70, 12.46, 12.03. **ESI-MS:** calculated $[C_{19}H_{16}BrNO_3 + H]^+$: 386.0386, found: 386.0382. $[\alpha]^{20}_{D}$ = -288.1 (c = 0.39, CH_2Cl_2). The product was analyzed by HPLC to determine the enantiomeric excess: 98% e.e. (CHIRALPAK AD-H, hexane/*i*-PrOH = 80/20, detector: 254 nm, T = 25 °C, flow rate: 1 mL/min), t_1(major) = 9.21 min, t_2(minor) = 10.07 min.

(*S*)-5-hydroxy-6,7-dimethyl-5'-(p-tolyl)-3',4'-dihydro-2H-spiro[benzofuran-3,2'-pyrrol]-2-one (4e)

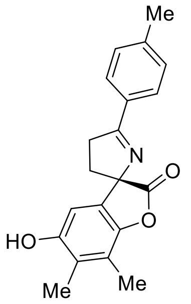

The title compound was prepared according to the general procedure E using **1de** (0.15 mmol) and **2a** (0.225 mmol). The crude reaction mixture was purified by flash column chromatography (petroleum ether/ethyl acetate, 4:1) to afford the title compound as a colorless oil (85%). **^{1}H NMR (500 MHz, $CDCl_3$)** δ 8.80 (s, 1H), 7.80 (d, J = 8.1 Hz, 2H), 7.28 (d, J = 8.0 Hz, 2H), 5.99 (s, 1H), 3.42 – 3.31 (m, 1H), 3.23 – 3.14 (m, 1H), 2.62 – 2.53 (m, 1H), 2.42 (s, 3H), 2.15 (s, 3H), 2.17 – 2.10 (m, 1H), 1.97 (s, 3H). **^{13}C NMR (151 MHz, $CDCl_3$)** δ 179.05, 177.10, 151.04, 145.50, 143.13, 129.80, 129.62, 128.67, 127.60, 124.19, 120.26, 109.92, 80.41, 36.50, 33.48, 21.78, 12.44, 11.99. **ESI-MS:** calculated $[C_{20}H_{19}NO_3 + Na]^+$: 344.1257, found: 344.1258. $[\alpha]^{20}_{D}$ = -321.7 (c = 0.26, CH_2Cl_2). The product was analyzed by HPLC to determine the enantiomeric excess: 93% e.e. (CHIRALPAK IA, hexane/*i*-PrOH = 80/20, detector: 254 nm, T = 25 °C, flow rate: 1 mL/min), t_1(minor) = 7.31 min, t_2(major) = 8.73 min.

(*S*)-5-hydroxy-5'-(4-methoxyphenyl)-6,7-dimethyl-3',4'-dihydro-2H-spiro[benzofuran-3,2'-pyrrol]-2-one (4f)

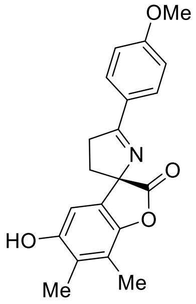

The title compound was prepared according to the general procedure E using **1df** (0.15 mmol) and **2a** (0.225 mmol). The crude reaction mixture was purified by flash column chromatography (petroleum ether/ethyl acetate, 2:1) to afford the title compound as a colorless oil (82%). **^{1}H NMR (600 MHz, $CDCl_3$)** δ 8.75 (s, 1H), 7.90 – 7.84 (m, 2H), 7.00 – 6.94 (m, 2H), 6.03 (s, 1H), 3.87 (s, 3H), 3.42 – 3.33 (m, 1H), 3.25 – 3.16 (m, 1H), 2.62 – 2.54 (m, 1H), 2.15 (s, 3H), 2.17 – 2.12 (m, 1H), 1.98 (s, 3H). **^{13}C NMR (151 MHz, $CDCl_3$)** δ 178.33, 177.27, 162.89, 151.04, 145.55, 130.57, 127.48, 125.08, 124.45, 120.31, 114.36, 109.89, 80.32, 55.64, 36.45, 33.62, 12.45, 12.01. **ESI-MS:** calculated $[C_{20}H_{19}NO_4 + Na]^+$: 360.1206, found: 360.1213. $[\alpha]^{20}_{D}$ = -83.1 (c = 0.30, CH_2Cl_2). The product was analyzed by HPLC to determine the enantiomeric excess: 96% e.e. (CHIRALPAK IA, hexane/*i*-PrOH = 80/20, detector: 254 nm, T = 25 °C, flow rate: 1 mL/min), t_1(minor) = 9.49 min, t_2(major) = 12.83 min.

(*S*)-5'-(3-chlorophenyl)-5-hydroxy-6,7-dimethyl-3',4'-dihydro-2H-spiro[benzofuran-3,2'-pyrrol]-2-one (4g)

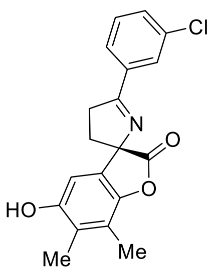

The title compound was prepared according to the general procedure E using **1dg** (0.15 mmol) and **2a** (0.225 mmol). The crude reaction mixture was purified by flash column chromatography (petroleum ether/ethyl acetate, 3:1) to afford the title compound as a colorless oil (89%). **^{1}H NMR (600 MHz, $CDCl_3$)** δ 7.85 – 7.79 (m, 2H), 7.56 (s, 1H), 7.52 – 7.48 (m, 1H), 7.43 – 7.38 (m, 1H), 6.08 (s, 1H), 3.43 – 3.35 (m, 1H), 3.27 – 3.19 (m, 1H), 2.67 – 2.60 (m, 1H), 2.24 – 2.19 (m, 1H), 2.17 (s, 3H), 2.01 (s, 3H). **^{13}C NMR (151 MHz, $CDCl_3$)** δ 177.95, 176.81, 150.93, 145.72, 135.09, 134.29, 132.19, 130.38, 128.68, 127.29, 126.56, 124.63, 120.79, 109.27, 80.82, 36.60, 33.70, 12.48, 12.03. **ESI-MS:** calculated $[C_{19}H_{16}ClNO_3 + H]^+$: 342.0891, found: 342.0891. $[\alpha]^{20}_D$ = -188.7 (c = 0.46, CH_2Cl_2). The product was analyzed by HPLC to determine the enantiomeric excess: 94% e.e. (CHIRALPAK IG, hexane/*i*-PrOH = 80/20, detector: 264 nm, T = 25 °C, flow rate: 1 mL/min), t_1(major) = 6.54 min, t_2(minor) = 11.05 min.

(*S*)-5-hydroxy-6,7-dimethyl-5'-(m-tolyl)-3',4'-dihydro-2H-spiro[benzofuran-3,2'-pyrrol]-2-one (4h)

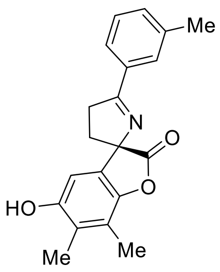

The title compound was prepared according to the general procedure E using **1dh** (0.15 mmol) and **2a** (0.225 mmol). The crude reaction mixture was purified by flash column chromatography (petroleum ether/ethyl acetate, 3:1) to afford the title compound as a colorless oil (91%). **^{1}H NMR (600 MHz, $CDCl_3$)** δ 8.46 (s, 1H), 7.73 – 7.69 (m, 2H), 7.38 – 7.34 (m, 2H), 6.02 (s, 1H), 3.41 – 3.32 (m, 1H), 3.22 – 3.14 (m, 1H), 2.63 – 2.55 (m, 1H), 2.41 (s, 3H), 2.16 (s, 3H), 2.17 – 2.13 (m, 1H), 1.99 (s, 3H). **^{13}C NMR (151 MHz, $CDCl_3$)** δ 179.21, 177.06, 151.04, 145.50, 138.82, 133.14, 132.34, 129.26, 128.97, 127.36, 125.74, 124.43, 120.40, 109.59, 80.52, 36.57, 33.51, 21.57, 12.45, 11.99. **ESI-MS:** calculated $[C_{20}H_{19}NO_3 + Na]^+$: 344.1257, found: 344.1259. $[\alpha]^{20}_D$ = -223.9 (c = 0.42, CH_2Cl_2). The product was analyzed by HPLC to determine the enantiomeric excess: 94% e.e. (CHIRALPAK IG, hexane/*i*-PrOH = 80/20, detector: 254 nm, T = 25 °C, flow rate: 1 mL/min), t_1(major) = 6.86 min, t_2(minor) = 10.36 min.

(*S*)-5-hydroxy-6,7-dimethyl-5'-(naphthalen-2-yl)-3',4'-dihydro-2H-spiro[benzofuran-3,2'-pyrrol]-2-one (4i)

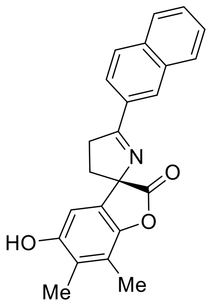

The title compound was prepared according to the general procedure E using **1di** (0.15 mmol) and **2a** (0.225 mmol). The crude reaction mixture was purified by flash column chromatography (petroleum ether/ethyl acetate, 3:1) to afford the title compound as a colorless oil (89%). **^{1}H NMR (600 MHz, $CDCl_3$)** δ 8.56 (s, 1H), 8.14 (s, 1H), 8.10 – 8.05 (m, 1H), 7.92 – 7.84 (m, 3H), 7.64 – 7.53 (m, 2H), 6.05 (s, 1H), 3.47 – 3.37 (m, 1H), 3.23 – 3.15 (m, 1H), 2.63 – 2.56 (m, 1H), 2.17 (s, 3H), 2.18 – 2.13 (m, 1H), 2.00 (s, 3H). **^{13}C NMR (151 MHz, $CDCl_3$)** δ 179.01, 177.07, 151.08, 145.53, 135.10, 132.79, 130.29, 129.78, 129.12, 128.99, 128.41, 128.04, 127.50, 127.16, 124.35, 124.23, 120.46, 109.67, 80.64, 36.42, 33.56, 12.46, 12.01. **ESI-MS:** calculated $[C_{23}H_{19}NO_3 + H]^+$: 358.1438, found: 358.1438. $[\alpha]^{20}_D$ = -321.7 (c = 0.30, CH_2Cl_2). The product was analyzed by HPLC to determine the enantiomeric excess: 94% e.e. (CHIRALPAK IG, hexane/*i*-PrOH = 80/20, detector: 254 nm, T = 25 °C, flow rate: 1 mL/min), t_1(major) = 10.39 min, t_2(minor) = 18.65 min.

(*S*)-5-hydroxy-6,7-dimethyl-5'-(thiophen-2-yl)-3',4'-dihydro-2H-spiro[benzofuran-3,2'-pyrrol]-2-one (4j)

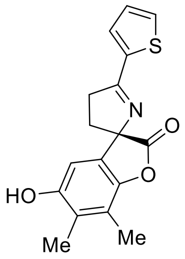

The title compound was prepared according to the general procedure E using **1dj** (0.15 mmol) and **2a** (0.225 mmol). The crude reaction mixture was purified by flash column chromatography (petroleum ether/ethyl acetate, 2:1) to afford the title compound as a colorless oil (87%). **^{1}H NMR (600 MHz, $CDCl_3$)** δ 8.30 (s, 1H), 7.59 – 7.52 (m, 2H), 7.19 – 7.13 (m, 1H), 6.13 (s, 1H), 3.51 – 3.42 (m, 1H), 3.30 – 3.22 (m, 1H), 2.66 – 2.59 (m, 1H), 2.24 – 2.16 (m, 1H), 2.15 (s, 3H), 1.97 (s, 3H). **^{13}C NMR (151 MHz, $CDCl_3$)** δ 176.96, 173.12, 151.08, 145.60, 136.93, 132.27, 131.81, 128.26, 127.50, 124.10, 120.30, 109.79, 80.27, 37.08, 34.09, 12.43, 12.00. **ESI-MS:** calculated $[C_{17}H_{15}NO_3S + H]^+$: 314.0845, found: 314.0847. $[\alpha]^{20}_{D}$ = -332.0 (c = 0.47, CH_2Cl_2). The product was analyzed by HPLC to determine the enantiomeric excess: 92% e.e. (CHIRALPAK AD-H, hexane/*i*-PrOH = 80/20, detector: 254 nm, T = 25 °C, flow rate: 1 mL/min), t_1(minor) = 9.43 min, t_2(major) = 10.94 min.

(*S*)-5-hydroxy-6,7-dimethyl-2'-phenyl-2H,5'H-spiro[benzofuran-3,4'-thiazol]-2-one (4k)

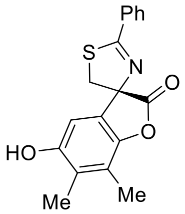

The title compound was prepared according to the general procedure E using **1dk** (0.15 mmol) and **2a** (0.225 mmol). The crude reaction mixture was purified by flash column chromatography (petroleum ether/ethyl acetate, 4:1) to afford the title compound as a yellow oil (46%). **^{1}H NMR (600 MHz, $CDCl_3$)** δ 7.86 – 7.82 (m, 2H), 7.58 – 7.52 (m, 1H), 7.48 – 7.43 (m, 2H), 6.39 (s, 1H), 3.95 (d, *J* = 11.4 Hz, 1H), 3.53 (d, *J* = 11.4 Hz, 1H), 2.21 (s, 3H), 2.07 (s, 3H). **^{13}C NMR (151 MHz, $CDCl_3$)** δ 175.52, 174.24, 150.83, 144.85, 132.69, 131.77, 128.94, 127.16, 124.15, 121.29, 108.32, 84.08, 41.26, 12.53, 12.05. **ESI-MS:** calculated $[C_{18}H_{15}NO_3S + H]^+$: 326.0845, found: 326.0844. $[\alpha]^{20}_{D}$ = -3.5 (c = 0.31, CH_2Cl_2). The product was analyzed by HPLC to determine the enantiomeric excess: 81% e.e. (CHIRALPAK IG, hexane/*i*-PrOH = 80/20, detector: 254 nm, T = 25 °C, flow rate: 1 mL/min), t_1(major) = 7.10 min, t_2(minor) = 9.72 min.

(*S*)-5-hydroxy-5'-phenyl-3',4'-dihydro-2H-spiro[naphtho[1,2-b]furan-3,2'-pyrrol]-2-one (4l)

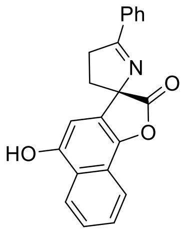

The title compound was prepared according to the general procedure E using **1d** (0.15 mmol) and **2c** (0.225 mmol). The crude reaction mixture was purified by flash column chromatography (petroleum ether/ethyl acetate, 2:1) to afford the title compound as a purple oil (70%). **^{1}H NMR (600 MHz, CD_3COCD_3)** δ 9.12 (s, 1H), 8.31 (d, *J* = 8.5 Hz, 1H), 8.02 – 7.96 (m, 3H), 7.70 – 7.65 (m, 1H), 7.62 – 7.52 (m, 2H), 7.52 – 7.44 (m, 2H), 6.85 (s, 1H), 3.59 – 3.46 (m, 2H), 2.78 – 2.70 (m, 1H), 2.56 – 2.48 (m, 1H). **^{13}C NMR (151 MHz, CD_3COCD_3)** δ 178.73, 178.12, 151.56, 142.35, 134.55, 132.28, 129.45, 129.19, 128.26, 126.72, 126.46, 125.86, 123.99, 121.46, 121.40, 103.45, 82.45, 37.13, 34.11. **ESI-MS:** calculated $[C_{21}H_{15}NO_3 + H]^+$: 330.1125, found: 330.1123. $[\alpha]^{20}_{D}$ = -203.2 (c = 0.12, CH_2Cl_2). The product was analyzed by HPLC to determine the enantiomeric excess: 81% e.e. (CHIRALPAK AD-H, hexane/*i*-PrOH = 80/20, detector: 254 nm, T = 25 °C, flow rate: 1 mL/min), t_1(minor) = 6.84 min, t_2(major) = 8.39 min.

(*S*)-5-hydroxy-6-methoxy-5'-phenyl-3',4'-dihydro-2H-spiro[benzofuran-3,2'-pyrrol]-2-one (4m)

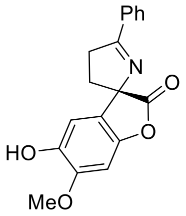

The title compound was prepared according to the general procedure E using **1d** (0.15 mmol) and **2d** (0.225 mmol). The crude reaction mixture was purified by flash column chromatography (petroleum ether/ethyl acetate, 2:1) to afford the title compound as a white solid (79%). **^{1}H NMR (600 MHz, $CDCl_3$)** δ 7.92 – 7.88 (m, 2H), 7.51 – 7.47 (m, 1H), 7.45 – 7.41 (m, 2H), 6.73 (s, 1H), 6.73 (s, 1H), 5.61 (s, 1H), 3.92 (s, 3H), 3.49 – 3.34 (m, 2H), 2.72 – 2.64 (m, 1H), 2.34 – 2.25 (m, 1H). **^{13}C NMR (151 MHz, $CDCl_3$)** δ 178.23, 177.54, 147.98, 146.73, 143.20, 133.37, 131.72, 128.72, 128.51, 121.52, 109.93, 95.55, 80.59, 56.58, 36.59, 34.15. **ESI-MS:** calculated $[C_{18}H_{15}NO_4 + H]^+$: 310.1074, found: 310.1073. $[\alpha]^{20}_D$ = -33.5 (c = 0.33, CH_2Cl_2). The product was analyzed by HPLC to determine the enantiomeric excess: 93% e.e. (CHIRALPAK IG, hexane/*i*-PrOH = 80/20, detector: 254 nm, T = 25 °C, flow rate: 1 mL/min), t_1(major) = 24.66 min, t_2(minor) = 28.46 min.

(*S*)-4-allyl-5-hydroxy-6-methoxy-5'-phenyl-3',4'-dihydro-2H-spiro[benzofuran-3,2'-pyrrol]-2-one (4n)

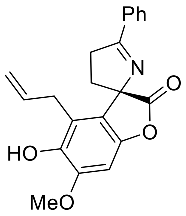

The title compound was prepared according to the general procedure E using **1d** (0.15 mmol) and **2e** (0.225 mmol). The crude reaction mixture was purified by flash column chromatography (petroleum ether/ethyl acetate, 3:1) to afford the title compound as a blue oil (79%). **^{1}H NMR (600 MHz, $CDCl_3$)** δ 7.94 – 7.90 (m, 2H), 7.53 – 7.48 (m, 1H), 7.47 – 7.42 (m, 2H), 6.63 (s, 1H), 5.98 – 5.88 (m, 1H), 5.80 (s, 1H), 4.96 (d, *J* = 10.1 Hz, 1H), 4.86 (d, *J* = 17.1 Hz, 1H), 3.89 (s, 3H), 3.54 – 3.46 (m, 1H), 3.40 – 3.26 (m, 2H), 3.13 – 3.06 (m, 1H), 2.66 – 2.59 (m, 1H), 2.49 – 2.41 (m, 1H). **^{13}C NMR (151 MHz, $CDCl_3$)** δ 178.34, 177.67, 147.67, 146.57, 141.10, 135.57, 133.37, 131.69, 128.73, 128.52, 123.50, 119.74, 115.47, 93.73, 81.52, 56.47, 36.68, 33.17, 30.26. **ESI-MS:** calculated $[C_{21}H_{19}NO_4 + H]^+$: 350.1387, found: 350.1387. $[\alpha]^{20}_D$ = -99.1 (c = 0.48, CH_2Cl_2). The product was analyzed by HPLC to determine the enantiomeric excess: 80% e.e. (CHIRALPAK AD-H, hexane/*i*-PrOH = 80/20, detector: 254 nm, T = 25 °C, flow rate: 1 mL/min), t_1(major) = 7.56 min, t_2(minor) = 10.16 min.

(*S,E*)-3-(benzylideneamino)-5-hydroxy-6-methoxy-3-methylbenzofuran-2(3H)-one (4o)

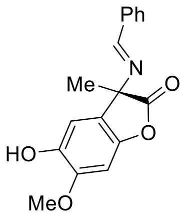

The title compound was prepared according to the general procedure E using **1f** (0.15 mmol) and **2d** (0.225 mmol). The crude reaction mixture was purified by flash column chromatography (petroleum ether/ethyl acetate, 3:1) to afford the title compound as a yellow oil (60%). **^{1}H NMR (600 MHz, CD_3CN)** δ 8.37 (s, 1H), 7.77 – 7.72 (m, 2H), 7.50 – 7.39 (m, 3H), 6.90 (s, 1H), 6.88 (s, 1H), 3.87 (s, 3H), 1.73 (s, 3H). **^{13}C NMR (151 MHz, CD_3CN)** δ 177.48, 162.03, 149.24, 146.47, 144.21, 136.59, 132.28, 129.59, 129.18, 121.39, 111.45, 96.87, 68.90, 56.98, 24.45. **ESI-MS:** calculated $[C_{17}H_{15}NO_4 + H]^+$: 298.1074, found: 298.1068. $[\alpha]^{20}_D$ = -2.0 (c = 0.22, CH_2Cl_2). The product was analyzed by HPLC to determine the enantiomeric excess: 93% e.e. (CHIRALPAK AD-H, hexane/*i*-PrOH = 90/10, detector: 254 nm, T = 25 °C, flow rate: 1 mL/min), t_1(major) = 14.60 min, t_2(minor) = 19.44 min.

Naphthalen-1-yl (*S*)-2-(4-hydroxy-3,5-dimethylphenoxy)-5-phenyl-3,4-dihydro-2H-pyrrole-2-carboxylate (5a)

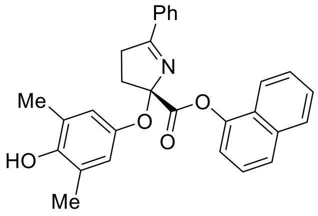

The title compound was prepared according to the general procedure F using **1d** (0.15 mmol) and **2b** (0.225 mmol). The crude reaction mixture was purified by flash column chromatography (petroleum ether/ethyl acetate, 4:1) to afford the title compound as a colorless oil (78%). **^{1}H NMR (600 MHz, CD_2Cl_2)** δ 8.09 – 8.04 (m, 2H), 7.90 – 7.85 (m, 1H), 7.79 – 7.74 (m, 1H), 7.59 – 7.54 (m, 2H), 7.53 – 7.48 (m, 3H), 7.48 – 7.44 (m, 1H), 7.43 – 7.39 (m, 1H), 7.15 – 7.10 (m, 1H), 7.04 (s, 2H), 4.65 (s, 1H), 3.32 – 3.20 (m, 2H), 3.05 – 2.97 (m, 1H), 2.69 – 2.62 (m, 1H), 2.24 (s, 6H). **^{13}C NMR (151 MHz, CD_2Cl_2)** δ 179.29, 170.08, 148.94, 148.32, 146.91, 134.92, 133.73, 132.25, 128.99, 128.87, 128.20, 127.02, 126.94, 126.88, 126.61, 125.67, 124.43, 121.53, 119.64, 118.08, 108.61, 35.25, 34.18, 16.42. **ESI-MS:** calculated $[C_{29}H_{25}NO_4 + H]^+$: 452.1856, found: 452.1860. $[\alpha]^{20}_{D}$ = +32.4 (c = 1.53, CH_2Cl_2). The product was analyzed by HPLC to determine the enantiomeric excess: 92% e.e. (CHIRALPAK AD-H, hexane/*i*-PrOH = 70/30, detector: 254 nm, T = 25 °C, flow rate: 1 mL/min), t_1(major) = 8.36 min, t_2(minor) = 10.14 min.

Naphthalen-1-yl (*S*)-5-(4-fluorophenyl)-2-(4-hydroxy-3,5-dimethylphenoxy)-3,4-dihydro-2H-pyrrole-2-carboxylate (5b)

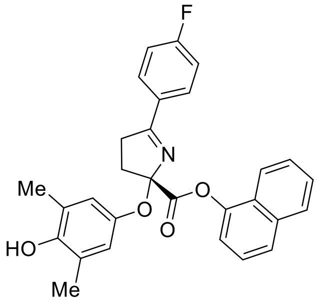

The title compound was prepared according to the general procedure F using **1db** (0.15 mmol) and **2b** (0.225 mmol). The crude reaction mixture was purified by flash column chromatography (petroleum ether/ethyl acetate, 4:1) to afford the title compound as a colorless oil (84%). **^{1}H NMR (600 MHz, CD_3CN)** δ 8.09 – 8.03 (m, 2H), 7.89 (d, *J* = 8.3 Hz, 1H), 7.78 (d, *J* = 8.3 Hz, 1H), 7.53 – 7.44 (m, 3H), 7.43 – 7.38 (m, 1H), 7.24 – 7.18 (m, 2H), 7.14 (d, *J* = 7.6 Hz, 1H), 7.02 (s, 2H), 5.94 (s, 1H), 3.26 – 3.13 (m, 2H), 3.04 – 2.96 (m, 1H), 2.64 – 2.56 (m, 1H), 2.21 (s, 6H). **^{13}C NMR (151 MHz, CD_3CN)** δ 178.93, 170.73, 165.85 (d, *J* = 250.4 Hz), 149.46 (d, *J* = 18.8 Hz), 147.37, 135.45, 131.84 (d, *J* = 9.1 Hz), 130.79 (d, *J* = 3.1 Hz), 128.85, 127.64, 127.58, 127.43, 127.26, 126.48, 125.86, 121.83, 119.89, 118.83, 116.63, 116.48, 109.06, 35.39, 35.01, 16.90. **^{19}F NMR (565 MHz, CD_3CN)** δ -109.36. **ESI-MS:** calculated $[C_{29}H_{24}FNO_4 + Na]^+$: 492.1582, found: 492.1591. $[\alpha]^{20}_{D}$ = +41.0 (c = 1.08, CH_2Cl_2). The product was analyzed by HPLC to determine the enantiomeric excess: 93% e.e. (CHIRALPAK AD-H, hexane/*i*-PrOH = 70/30, detector: 254 nm, T = 25 °C, flow rate: 1 mL/min), t_1(major) = 8.14 min, t_2(minor) = 10.70 min.

Naphthalen-1-yl (*S*)-5-(4-chlorophenyl)-2-(4-hydroxy-3,5-dimethylphenoxy)-3,4-dihydro-2H-pyrrole-2-carboxylate (5c)

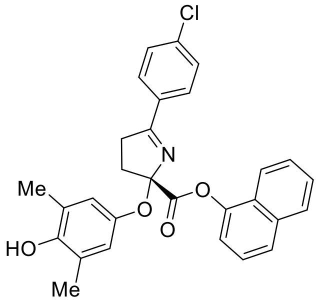

The title compound was prepared according to the general procedure F using **1dc** (0.15 mmol) and **2b** (0.225 mmol). The crude reaction mixture was purified by flash column chromatography (petroleum ether/ethyl acetate, 4:1) to afford the title compound as a colorless oil (84%). **^{1}H NMR (600 MHz, $CDCl_3$)** δ 8.01 – 7.96 (m, 2H), 7.84 – 7.80 (m, 1H), 7.73 – 7.69 (m, 1H), 7.58 – 7.54 (m, 1H), 7.47 – 7.44 (m, 3H), 7.43 – 7.39 (m, 1H), 7.37 – 7.33 (m, 1H), 7.14 – 7.09 (m, 1H), 7.01 (s, 2H), 4.51 (s, 1H), 3.28 – 3.15 (m, 2H), 3.09 – 3.01 (m, 1H), 2.67 – 2.59 (m,

1H), 2.23 (s, 6H). **^{13}C NMR (151 MHz, CDCl$_3$)** δ 177.73, 169.68, 148.53, 147.96, 146.62, 138.21, 134.68, 131.83, 130.04, 129.00, 127.91, 126.77, 126.58, 126.49, 126.37, 125.35, 124.13, 121.42, 119.26, 117.77, 108.46, 35.21, 33.51, 16.39. **ESI-MS:** calculated $[C_{29}H_{24}ClNO_4 + H]^+$: 486.1467, found: 486.1470. $[\alpha]^{20}_D$ = +44.6 (c = 1.75, CH_2Cl_2). The product was analyzed by HPLC to determine the enantiomeric excess: 95% e.e. (CHIRALPAK AD-H, hexane/*i*-PrOH = 70/30, detector: 254 nm, T = 25 °C, flow rate: 1 mL/min), t_1(major) = 8.51 min, t_2(minor) = 11.75 min.

Naphthalen-1-yl (*S*)-5-(4-bromophenyl)-2-(4-hydroxy-3,5-dimethylphenoxy)-3,4-dihydro-2H-pyrrole-2-carboxylate (5d)

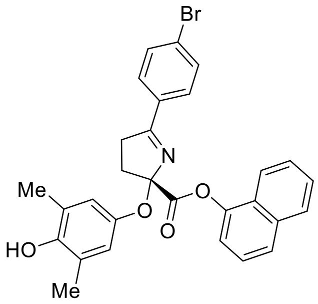

The title compound was prepared according to the general procedure F using **1dd** (0.15 mmol) and **2b** (0.225 mmol). The crude reaction mixture was purified by flash column chromatography (petroleum ether/ethyl acetate, 4:1) to afford the title compound as a colorless oil (86%). **^{1}H NMR (600 MHz, CDCl$_3$)** δ 7.93 – 7.89 (m, 2H), 7.82 (d, *J* = 8.3 Hz, 1H), 7.71 (d, *J* = 8.2 Hz, 1H), 7.63 – 7.59 (m, 2H), 7.55 (d, *J* = 8.4 Hz, 1H), 7.48 – 7.44 (m, 1H), 7.43 – 7.40 (m, 1H), 7.36 – 7.32 (m, 1H), 7.11 (d, *J* = 7.6 Hz, 1H), 7.00 (s, 2H), 4.46 (s, 1H), 3.28 – 3.15 (m, 2H), 3.09 – 3.01 (m, 1H), 2.67 – 2.59 (m, 1H), 2.23 (s, 6H). **^{13}C NMR (151 MHz, CDCl$_3$)** δ 177.85, 169.64, 148.54, 147.96, 146.63, 134.69, 132.26, 131.99, 130.22, 127.92, 126.80, 126.78, 126.59, 126.50, 126.38, 125.36, 124.10, 121.42, 119.28, 117.77, 108.49, 35.20, 33.51, 16.40. **ESI-MS:** calculated $[C_{29}H_{24}BrNO_4 + H]^+$: 530.0961, found: 530.0966. $[\alpha]^{20}_D$ = +38.4 (c = 1.98, CH_2Cl_2). The product was analyzed by HPLC to determine the enantiomeric excess: 96% e.e. (CHIRALPAK AD-H, hexane/*i*-PrOH = 80/20, detector: 254 nm, T = 25 °C, flow rate: 1 mL/min), t_1(major) = 14.55 min, t_2(minor) = 21.50 min.

Naphthalen-1-yl (*S*)-2-(4-hydroxy-3,5-dimethylphenoxy)-5-(p-tolyl)-3,4-dihydro-2H-pyrrole-2-carboxylate (5e)

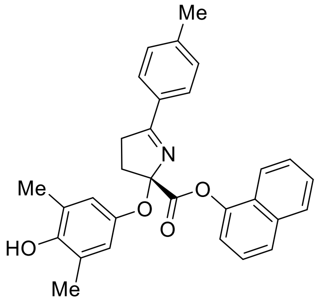

The title compound was prepared according to the general procedure F using **1de** (0.15 mmol) and **2b** (0.225 mmol). The crude reaction mixture was purified by flash column chromatography (petroleum ether/ethyl acetate, 4:1) to afford the title compound as a colorless oil (78%). **^{1}H NMR (600 MHz, CDCl$_3$)** δ 7.96 – 7.93 (m, 2H), 7.83 – 7.80 (m, 1H), 7.72 – 7.69 (m, 1H), 7.62 – 7.59 (m, 1H), 7.47 – 7.43 (m, 1H), 7.43 – 7.39 (m, 1H), 7.36 – 7.32 (m, 1H), 7.29 – 7.26 (m, 2H), 7.13 – 7.10 (m, 1H), 7.00 (s, 2H), 4.43 (s, 1H), 3.30 – 3.17 (m, 2H), 3.09 – 3.01 (m, 1H), 2.63 – 2.55 (m, 1H), 2.43 (s, 3H), 2.23 (s, 6H). **^{13}C NMR (151 MHz, CDCl$_3$)** δ 178.71, 169.87, 148.61, 147.85, 146.75, 142.52, 134.69, 130.76, 129.42, 128.77, 127.87, 126.86, 126.55, 126.47, 126.29, 125.36, 124.04, 121.60, 119.37, 117.84, 108.63, 35.34, 33.22, 21.77, 16.40. **ESI-MS:** calculated $[C_{30}H_{27}NO_4 + H]^+$: 466.2103, found: 466.2105. $[\alpha]^{20}_D$ = +34.0 (c = 1.39, CH_2Cl_2). The product was analyzed by HPLC to determine the enantiomeric excess: 95% e.e. (CHIRALPAK AD-H, hexane/*i*-PrOH = 80/20, detector: 254 nm, T = 25 °C, flow rate: 1 mL/min), t_1(major) = 10.60 min, t_2(minor) = 12.36 min.

Naphthalen-1-yl (*S*)-5-(3-chlorophenyl)-2-(4-hydroxy-3,5-dimethylphenoxy)-3,4-dihydro-2H-pyrrole-2-carboxylate (5f)

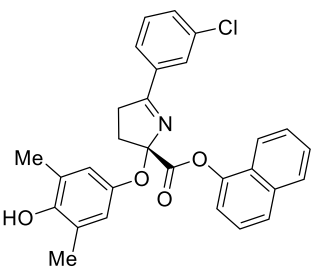

The title compound was prepared according to the general procedure F using **1dg** (0.15 mmol) and **2b** (0.225 mmol). The crude reaction mixture was purified by flash column chromatography (petroleum ether/ethyl acetate, 4:1) to afford the title compound as a colorless oil (87%). **^{1}H NMR (600 MHz, $CDCl_3$)** δ 8.09 – 8.05 (m, 1H), 7.92 – 7.87 (m, 1H), 7.84 – 7.80 (m, 1H), 7.74 – 7.69 (m, 1H), 7.59 – 7.54 (m, 1H), 7.51 – 7.49 (m, 1H), 7.48 – 7.44 (m, 1H), 7.43 – 7.39 (m, 2H), 7.38 – 7.34 (m, 1H), 7.14 – 7.09 (m, 1H), 7.01 (s, 2H), 4.46 (s, 1H), 3.29 – 3.16 (m, 2H), 3.09 – 3.01 (m, 1H), 2.68 – 2.60 (m, 1H), 2.24 (s, 6H). **^{13}C NMR (151 MHz, $CDCl_3$)** δ 177.67, 169.58, 148.54, 147.99, 146.62, 135.12, 134.86, 134.69, 131.96, 130.02, 128.73, 127.92, 126.86, 126.78, 126.60, 126.52, 126.39, 125.36, 124.12, 121.44, 119.28, 117.79, 108.39, 35.27, 33.54, 16.40. **ESI-MS:** calculated $[C_{29}H_{24}ClNO_4 + H]^+$: 486.1467, found: 486.1467. $[\alpha]^{20}_{D}$ = +23.8 (c = 2.08, CH_2Cl_2). The product was analyzed by HPLC to determine the enantiomeric excess: 95% e.e. (CHIRALPAK AD-H, hexane/*i*-PrOH = 70/30, detector: 254 nm, T = 25 °C, flow rate: 1 mL/min), t_1(major) = 10.34 min, t_2(minor) = 15.85 min.

Naphthalen-1-yl (*S*)-2-(3-((diisopropylamino)methyl)-4-hydroxy-5-methylphenoxy)-5-phenyl-3,4-dihydro-2H-pyrrole-2-carboxylate (5g)

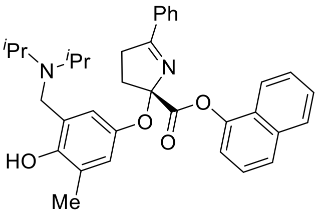

The title compound was prepared according to the general procedure F using **1d** (0.15 mmol) and **2f** (0.225 mmol). The crude reaction mixture was purified by flash column chromatography (petroleum ether/ethyl acetate, 4:1) to afford the title compound as a colorless oil (78%). **^{1}H NMR (500 MHz, CD_3CN)** δ 8.07 – 8.01 (m, 2H), 7.90 (d, J = 8.3 Hz, 1H), 7.79 (d, J = 8.3 Hz, 1H), 7.59 – 7.37 (m, 7H), 7.15 – 7.10 (m, 1H), 7.06 (d, *J* = 2.9 Hz, 1H), 6.92 (d, *J* = 3.0 Hz, 1H), 3.78 (s, 2H), 3.25 – 3.19 (m, 2H), 3.08 – 2.96 (m, 3H), 2.65 – 2.54 (m, 1H), 2.15 (s, 3H), 1.01 (d, *J* = 6.7 Hz, 12H). **^{13}C NMR (151 MHz, CD_3CN)** δ 180.03, 170.73, 153.31, 148.44, 147.32, 135.44, 134.26, 132.83, 129.63, 129.33, 128.81, 127.62, 127.40, 127.19, 126.44, 125.77, 123.66, 121.85, 120.97, 118.83, 118.01, 109.13, 49.11, 48.46, 35.31, 34.79, 19.90, 15.96. **ESI-MS:** calculated $[C_{35}H_{38}N_2O_4 + H]^+$: 551.2904, found: 551.2906**.** $[\alpha]^{20}_{D}$ = +15.9 (c = 1.14, CH_2Cl_2). The product was analyzed by HPLC to determine the enantiomeric excess: 93% e.e. (CHIRALPAK AD-H, hexane/*i*-PrOH = 90/10, detector: 272 nm, T = 25 °C, flow rate: 1 mL/min), t_1(minor) = 8.45 min, t_2(major) = 9.77 min.

Naphthalen-1-yl (*S*)-2-(3-((diethylamino)methyl)-4-hydroxy-5-methylphenoxy)-5-phenyl-3,4-dihydro-2H-pyrrole-2-carboxylate (5h)

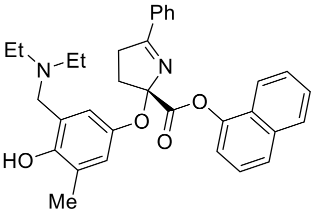

The title compound was prepared according to the general procedure F using **1d** (0.15 mmol) and **2g** (0.225 mmol). The crude reaction mixture was purified by flash column chromatography (petroleum ether/ethyl acetate, 2:1) to afford the title compound as a colorless oil (69%). **^{1}H NMR (500 MHz, CD_3CN)** δ 8.06 – 8.01 (m, 2H), 7.90 (d, *J* = 8.4 Hz, 1H), 7.79 (d, *J* = 8.3 Hz, 1H), 7.58 – 7.37 (m, 7H), 7.14 – 7.11 (m, 1H), 7.10 (d, *J* = 2.9 Hz, 1H), 6.91 (d, *J* = 3.0 Hz, 1H), 3.72 – 3.63 (m, 2H), 3.24 – 3.18 (m, 2H), 3.04 – 2.95 (m, 1H), 2.65 – 2.56 (m, 1H), 2.50 (q, *J* = 7.2 Hz, 4H), 2.16 (s, 3H), 1.00 (t, *J* = 7.2 Hz, 6H). **^{13}C NMR**

(151 MHz, CD_3CN) δ 180.05, 170.72, 153.00, 148.46, 147.31, 135.42, 134.21, 132.82, 129.61, 129.31, 128.81, 127.62, 127.39, 127.20, 126.44, 125.94, 123.14, 121.81, 121.26, 118.82, 118.16, 109.14, 57.34, 46.82, 35.28, 34.79, 16.02, 11.46. **ESI-MS:** calculated $[C_{33}H_{34}N_2O_4 + H]^+$: 523.2591, found: 523.2590. $[\alpha]^{20}_D$ = +21.3 (c = 1.15, CH_2Cl_2). The product was analyzed by HPLC to determine the enantiomeric excess: 92% e.e. (CHIRALPAK IG, hexane/*i*-PrOH = 80/20, detector: 255 nm, T = 25 °C, flow rate: 1 mL/min), t_1(minor) = 14.29 min, t_2(major) = 16.09 min.

Naphthalen-1-yl (*S*)-2-(3-((diallylamino)methyl)-4-hydroxy-5-methylphenoxy)-5-phenyl-3,4-dihydro-2H-pyrrole-2-carboxylate (5i)

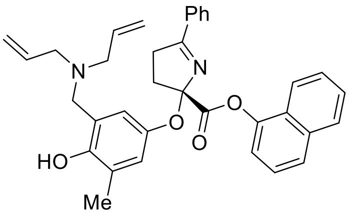

The title compound was prepared according to the general procedure F using **1d** (0.15 mmol) and **2h** (0.225 mmol). The crude reaction mixture was purified by flash column chromatography (petroleum ether/ethyl acetate, 2:1) to afford the title compound as a colorless oil (61%). **^{1}H NMR (500 MHz, CD_3CN)** δ 8.06 – 8.01 (m, 2H), 7.90 (d, *J* = 8.3 Hz, 1H), 7.79 (d, *J* = 8.3 Hz, 1H), 7.59 – 7.36 (m, 7H), 7.14 – 7.12 (m, 1H), 7.11 (d, *J* = 3.0 Hz, 1H), 6.92 (d, *J* = 3.0 Hz, 1H), 5.92 – 5.80 (m, 2H), 5.21 – 5.08 (m, 4H), 3.73 – 3.62 (m, 2H), 3.25 – 3.18 (m, 2H), 3.11 – 3.03 (m, 4H), 3.02 – 2.96 (m, 1H), 2.67 – 2.56 (m, 1H), 2.17 (s, 3H). **^{13}C NMR (126 MHz, CD_3CN)** δ 180.14, 170.70, 152.75, 148.70, 147.38, 135.49, 134.58, 134.30, 132.88, 129.67, 129.37, 128.86, 127.69, 127.67, 127.45, 127.24, 126.48, 126.17, 122.83, 121.84, 121.66, 119.78, 118.87, 118.55, 109.27, 57.06, 56.13, 35.36, 34.81, 16.05. **ESI-MS:** calculated $[C_{35}H_{34}N_2O_4 + Na]^+$: 569.2411, found: 569.2415. $[\alpha]^{20}_D$ = +8.1 (c = 0.37, CH_2Cl_2). The product was analyzed by HPLC to determine the enantiomeric excess: 93% e.e. (CHIRALPAK IG, hexane/*i*-PrOH = 80/20, detector: 254 nm, T = 25 °C, flow rate: 1 mL/min), t_1(minor) = 14.72 min, t_2(major) = 15.97 min.

Naphthalen-1-yl (*R*)-5-phenyl-2-(p-tolylthio)-3,4-dihydro-2H-pyrrole-2-carboxylate (7a)

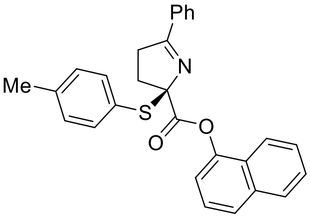

The title compound was prepared according to the general procedure G using **5a** (0.24 mmol) and **6a** (0.20 mmol). The crude reaction mixture was purified by flash column chromatography (petroleum ether/ethyl acetate, 6:1) to afford the title compound as a colorless oil (79%). **^{1}H NMR (600 MHz, $CDCl_3$)** δ 8.06 – 8.03 (m, 1H), 8.01 – 7.98 (m, 2H), 7.88 – 7.85 (m, 1H), 7.75 (d, *J* = 8.3 Hz, 1H), 7.61 – 7.57 (m, 2H), 7.54 – 7.44 (m, 6H), 7.28 – 7.26 (m, 1H), 7.13 – 7.10 (m, 2H), 3.15 – 3.07 (m, 1H), 3.01 – 2.93 (m, 1H), 2.80 – 2.72 (m, 1H), 2.66 – 2.58 (m, 1H), 2.35 (s, 3H). **^{13}C NMR (151 MHz, $CDCl_3$)** δ 176.33, 169.63, 146.82, 139.75, 136.67, 134.70, 133.64, 131.55, 129.76, 128.62, 128.50, 127.91, 127.37, 126.91, 126.51, 126.16, 125.39, 121.70, 117.95, 89.68, 35.42, 33.50, 21.40. **ESI-MS:** calculated $[C_{28}H_{23}NO_2S + Na]^+$: 460.1342, found: 460.1344. $[\alpha]^{20}_D$ = -8.8 (c = 1.13, CH_2Cl_2). The product was analyzed by HPLC to determine the enantiomeric excess: 92% e.e. (CHIRALPAK IA, hexane/*i*-PrOH = 80/20, detector: 254 nm, T = 25 °C, flow rate: 1 mL/min), t_1(major) = 8.77 min, t_2(minor) = 9.34 min.

Naphthalen-1-yl (*R*)-2-((4-ethylphenyl)thio)-5-phenyl-3,4-dihydro-2H-pyrrole-2-carboxylate (7b)

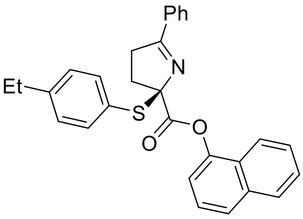

The title compound was prepared according to the general procedure G using **5a** (0.24 mmol) and **6b** (0.20 mmol). The crude reaction mixture was purified by flash column chromatography (petroleum ether/ethyl acetate, 6:1) to afford the title compound as a colorless oil (83%). **^{1}H NMR (600 MHz, CDCl$_3$)** δ 8.07 – 8.04 (m, 1H), 8.00 – 7.97 (m, 2H), 7.88 – 7.85 (m, 1H), 7.75 (d, *J* = 8.3 Hz, 1H), 7.64 – 7.60 (m, 2H), 7.54 – 7.44 (m, 6H), 7.25 – 7.22 (m, 1H), 7.16 – 7.12 (m, 2H), 3.14 – 3.06 (m, 1H), 3.02 – 2.93 (m, 1H), 2.77 – 2.69 (m, 1H), 2.69 – 2.59 (m, 3H), 1.22 (t, *J* = 7.6 Hz, 3H). **^{13}C NMR (151 MHz, CDCl$_3$)** δ 176.31, 169.67, 146.82, 146.02, 136.76, 134.70, 133.64, 131.55, 128.62, 128.56, 128.49, 127.92, 127.61, 126.92, 126.52, 126.17, 125.39, 121.69, 117.95, 89.73, 35.39, 33.60, 28.70, 15.51. **ESI-MS:** calculated [$C_{29}H_{25}NO_2S$ + Na]$^+$: 474.1498, found: 474.1501. $[\alpha]^{20}_D$ = -2.6 (c = 1.21, CH_2Cl_2). The product was analyzed by HPLC to determine the enantiomeric excess: 92% e.e. (CHIRALPAK IF, hexane/*i*-PrOH = 80/20, detector: 254 nm, T = 25 °C, flow rate: 1 mL/min), t_1(major) = 8.08 min, t_2(minor) = 8.77 min.

Naphthalen-1-yl (*R*)-2-((4-(*tert*-butyl)phenyl)thio)-5-phenyl-3,4-dihydro-2H-pyrrole-2-carboxylate (7c)

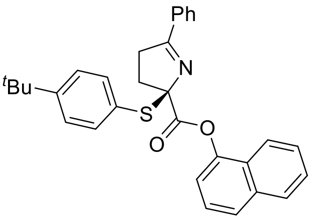

The title compound was prepared according to the general procedure G using **5a** (0.24 mmol) and **6c** (0.20 mmol). The crude reaction mixture was purified by flash column chromatography (petroleum ether/ethyl acetate, 6:1) to afford the title compound as a colorless oil (88%). **^{1}H NMR (600 MHz, CDCl$_3$)** δ 8.07 – 8.03 (m, 1H), 7.98 – 7.94 (m, 2H), 7.87 – 7.83 (m, 1H), 7.74 (d, *J* = 8.3 Hz, 1H), 7.64 – 7.59 (m, 2H), 7.53 – 7.41 (m, 6H), 7.33 – 7.30 (m, 2H), 7.19 – 7.17 (m, 1H), 3.12 – 3.05 (m, 1H), 3.01 – 2.92 (m, 1H), 2.74 – 2.60 (m, 2H), 1.29 (s, 9H). **^{13}C NMR (151 MHz, CDCl$_3$)** δ 176.30, 169.74, 152.87, 146.88, 136.45, 134.75, 133.72, 131.57, 128.63, 128.52, 127.95, 127.57, 126.97, 126.57, 126.54, 126.19, 126.04, 125.41, 121.73, 117.96, 89.79, 35.41, 34.83, 33.82, 31.35. **ESI-MS:** calculated [$C_{31}H_{29}NO_2S$ + H]$^+$: 480.1992, found: 480.1996. $[\alpha]^{20}_D$ = -5.1 (c = 1.67, CH_2Cl_2). The product was analyzed by HPLC to determine the enantiomeric excess: 92% e.e. (CHIRALPAK IF, hexane/*i*-PrOH = 80/20, detector: 256 nm, T = 25 °C, flow rate: 1 mL/min), t_1(major) = 7.65 min, t_2(minor) = 8.35 min.

Naphthalen-1-yl (*R*)-2-((4-methoxyphenyl)thio)-5-phenyl-3,4-dihydro-2H-pyrrole-2-carboxylate (7d)

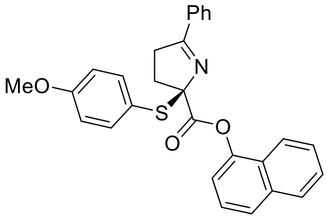

The title compound was prepared according to the general procedure G using **5a** (0.24 mmol) and **6d** (0.20 mmol). The crude reaction mixture was purified by flash column chromatography (petroleum ether/ethyl acetate, 6:1) to afford the title compound as a colorless oil (96%). **^{1}H NMR (500 MHz, CDCl$_3$)** δ 8.09 – 8.04 (m, 1H), 8.00 – 7.95 (m, 2H), 7.89 – 7.84 (m, 1H), 7.75 (d, *J* = 8.3 Hz, 1H), 7.62 – 7.58 (m, 2H), 7.54 – 7.44 (m, 6H), 7.30 (dd, *J* = 7.5, 1.0 Hz, 1H), 6.85 – 6.79 (m, 2H), 3.76 (s, 3H), 3.15 – 3.04 (m, 1H), 3.00 – 2.89 (m, 1H), 2.74 – 2.58 (m, 2H). **^{13}C NMR (126 MHz, CDCl$_3$)** δ 176.22, 169.62, 160.99, 146.78, 138.67, 134.69, 133.60, 131.52, 128.61, 128.45, 127.91, 126.90, 126.52, 126.15, 125.38, 121.67, 121.35, 117.95, 114.48, 89.82, 55.37, 35.39, 33.32. **ESI-MS:** calculated [$C_{28}H_{23}NO_3S$ + Na]$^+$: 476.1291, found: 476.1295. $[\alpha]^{20}_{D}$ = -11.9 (c = 1.93, CH_2Cl_2). The product was analyzed by HPLC to determine the enantiomeric excess: 92% e.e. (CHIRALPAK AD-H, hexane/*i*-PrOH = 80/20, detector: 266 nm, T = 25 °C, flow rate: 1 mL/min), t_1(minor) = 11.48 min, t_2(major) = 12.72 min.

2.4 Synthesis and Characterization of (-) Fumimycin

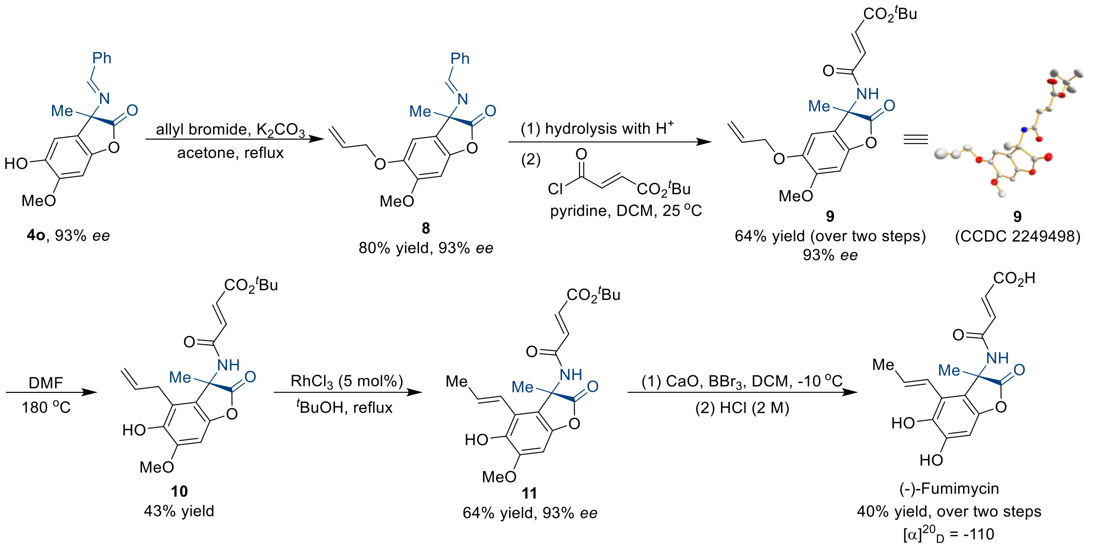

To a round-bottom flask was added **4o** (1.0 mmol), allyl bromide (1.3 mmol), K_2CO_3 (1.3 mmol), and acetone (15 mL), the mixture was heated to reflux in an oil bath for 5 h. The reaction mixture was allowed to cool to 25 ºC. K_2CO_3 was filtered off, and the filtrate was concentrated in vacuo. The residue was purified by silica gel chromatography (petroleum ether/ethyl acetate, 6:1) to afford the desired product **8**.

To a round-bottom flask was added **8** (0.8 mmol) and CH_2Cl_2 (10 mL), then 2M HCl (3ml) was added. The reaction mixture was stirred overnight at 25 ºC. The solvent was removed under reduced pressure. The residue was dissolved in CH_2Cl_2 (10 mL) at 0 ºC. Pyridine (8 mmol) and *tert*-butyl (*E*)-4-chloro-4-oxobut-2-enoate (1.1 mmol) were added. The reaction was allowed to warm to 25 ºC and stirred for 4 h. The solvent was removed under reduced pressure. The residue was diluted with water (10 mL). The aqueous layer was extracted with EtOAc (3×15 mL). The combined organic extracts were washed with brine (20 mL), dried over $MgSO_4$ and concentrated under reduced pressure. The residue was purified by silica gel chromatography (petroleum ether/ethyl acetate, 2:1) to afford the desired product **9**.

To a round-bottom flask was added **9** (0.6 mmol) in DMF (3 mL), the mixture was heated to reflux in an oil bath for 1 h. The reaction mixture was allowed to cool to 25 ºC. The solvent was removed under reduced pressure. The residue was purified by silica gel chromatography (petroleum ether/ethyl acetate, 2:1) to afford the desired product **10** and substrate **9**. Then, repeat these steps 2-3 times until substrate **9** was exhausted. The combined product **10** was used for the next step.

A mixture of **10** (0.3 mmol) and $RhCl_3·3H_2O$ (0.015 mmol) in *tert*-butanol (3 mL) was heated to reflux in an oil bath for 24 h under argon. The reaction mixture was allowed to cool to 25 ºC. The solvent was removed under reduced pressure. The residue was purified by silica gel chromatography (petroleum ether/ethyl acetate, 1:1) to afford the desired product **11**.

To a Schlenk tube was added **11** (0.15 mmol), CaO (1.5 mmol), and anhydrous CH_2Cl_2 (2 mL), then BBr_3 (1M in CH_2Cl_2, 1.5 mmol) was added drop wise slowly, the reaction was stirred at -10 ºC for 4 h under argon. Saturated $NaHCO_3$ solution was added to quench the reaction and the phases were separated. The aqueous layer was acidified with 2 M HCl (pH 2−3) and extracted with EtOAc (3 × 10 mL). The combined organic extracts were dried over $MgSO_4$ and concentrated under reduced pressure. The residue was purified by silica gel chromatography (DCM/MeOH, 4:1, +1% acetic acid) to afford the desired product (−)-**Fumimycin**.

(*S*,*E*)-5-(allyloxy)-3-(benzylideneamino)-6-methoxy-3-methylbenzofuran-2(3H)-one (8)

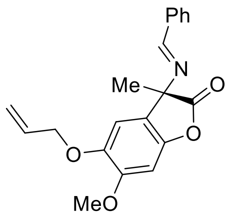

Yellow oil (80%). **^{1}H NMR (600 MHz, CD$_3$COCD$_3$)** δ 8.45 (s, 1H), 7.84 – 7.79 (m, 2H), 7.51 – 7.41 (m, 3H), 7.17 (s, 1H), 6.96 (s, 1H), 6.12 – 6.03 (m, 1H), 5.42 (d, *J* = 16.7 Hz, 1H), 5.23 (d, *J* = 10.4 Hz, 1H), 4.60 – 4.56 (m, 2H), 3.89 (s, 3H), 1.79 (s, 3H). **^{13}C NMR (151 MHz, CD$_3$COCD$_3$)** δ 176.96, 161.71, 152.45, 147.72, 146.44, 136.80, 134.84, 132.21, 129.48, 129.31, 120.96, 117.58, 111.74, 97.40, 71.20, 68.91, 56.61, 24.66. **ESI-MS:** calculated [$C_{20}H_{19}NO_4$ + Na]$^+$: 360.1206, found: 360.1215. $[\alpha]^{20}_{D}$ = -12.9 (c = 0.36, CH_2Cl_2). The product was analyzed by HPLC to determine the enantiomeric excess: 93% e.e. (CHIRALPAK AD-H, hexane/*i*-PrOH = 90/10, detector: 254 nm, T = 25 °C, flow rate: 1 mL/min), t_1(major) = 6.80 min, t_2(minor) = 7.30 min.

***tert*-butyl (*S*,*E*)-4-((5-(allyloxy)-6-methoxy-3-methyl-2-oxo-2,3-dihydrobenzofuran-3-yl)amino)-4-oxobut-2-enoate (9)**

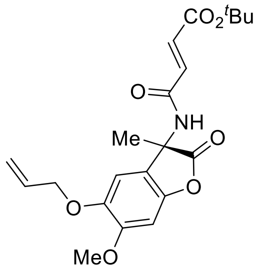

Brown oil (64%). **^{1}H NMR (600 MHz, CD$_3$COCD$_3$)** δ 8.64 (s, 1H), 7.00 (s, 1H), 6.92 (d, *J* = 15.2 Hz, 1H), 6.87 (s, 1H), 6.50 (d, *J* = 15.5 Hz, 1H), 6.09 – 5.99 (m, 1H), 5.38 (d, *J* = 17.3 Hz, 1H), 5.20 (d, *J* = 9.6 Hz, 1H), 4.51 – 4.48 (m, 2H), 3.87 (s, 3H), 1.65 (s, 3H), 1.47 (s, 9H). **^{13}C NMR (151 MHz, CD$_3$COCD$_3$)** δ 176.56, 164.87, 163.38, 152.28, 148.62, 146.28, 135.00, 134.92, 133.15, 121.01, 117.40, 110.19, 97.28, 81.84, 71.38, 58.16, 56.57, 28.05, 24.28. **ESI-MS:** calculated [$C_{21}H_{25}NO_7$ + Na]$^+$: 426.1523, found: 426.1526. $[\alpha]^{20}_{D}$ = +1.2 (c = 0.38, CH_2Cl_2). The product was analyzed by HPLC to determine the enantiomeric excess: 93% e.e. (CHIRALPAK AD-H, hexane/*i*-PrOH = 70/30, detector: 254 nm, T = 25 °C, flow rate: 1 mL/min), t_1(minor) = 3.94 min, t_2(major) = 4.46 min.

***tert*-butyl (*E*)-4-(((*S*)-5-hydroxy-6-methoxy-3-methyl-2-oxo-4-((*E*)-prop-1-en-1-yl)-2,3-dihydrobenzofuran-3-yl)amino)-4-oxobut-2-enoate (11)**

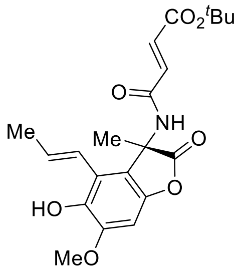

White solid (64%). **^{1}H NMR (600 MHz, CD$_3$COCD$_3$)** δ 8.83 (s, 1H), 7.58 (s, 1H), 6.94 (d, *J* = 15.3 Hz, 1H), 6.84 – 6.77 (m, 1H), 6.75 (s, 1H), 6.57 (d, *J* = 15.8 Hz, 1H), 6.52 (d, *J* = 15.1 Hz, 1H), 3.90 (s, 3H), 1.86 (d, *J* = 6.7 Hz, 3H), 1.71 (s, 3H), 1.47 (s, 9H). **^{13}C NMR (151 MHz, CD$_3$COCD$_3$)** δ 176.55, 164.91, 163.24, 148.78, 146.83, 142.34, 134.95, 133.91, 133.21, 123.05, 121.15, 118.07, 94.66, 81.87, 58.88, 56.86, 28.06, 23.62, 19.84. **ESI-MS:** calculated [$C_{21}H_{25}NO_7$ + Na]$^+$: 426.1523, found: 426.1528. $[\alpha]^{20}_{D}$ = -12.8 (c = 0.18, CH_2Cl_2). The product was analyzed by HPLC to determine the enantiomeric excess: 93% e.e. (CHIRALPAK IA, hexane/*i*-PrOH = 70/30, detector: 316 nm, T = 25 °C, flow rate: 1 mL/min), t_1(minor) = 5.23 min, t_2(major) = 6.34 min.

(*E*)-4-(((*S*)-5,6-dihydroxy-3-methyl-2-oxo-4-((*E*)-prop-1-en-1-yl)-2,3-dihydrobenzofuran-3-yl)amino)-4-oxobut-2-enoic acid ((−)-Fumimycin)

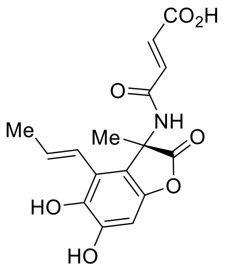

Brown oil (40%). **^{1}H NMR (600 MHz, CD_3OD)** δ 6.81 (d, *J* = 15.5 Hz, 1H), 6.69 – 6.62 (m, 2H), 6.50 (s, 1H), 6.41 (dq, *J* = 15.8, 1.7 Hz, 1H), 1.91 (dd, *J* = 6.7, 1.7 Hz, 3H), 1.65 (s, 3H). **^{13}C NMR (151 MHz, CD_3OD)** δ 178.44, 172.63, 166.19, 147.30, 147.26, 141.79, 137.64, 134.23, 132.26, 123.28, 122.78, 117.35, 97.70, 59.45, 23.50, 19.69. **ESI-MS:** calculated $[C_{16}H_{15}NO_7 + Na]^+$: 356.0741, found: 356.0736. $[\alpha]^{20}_D$ = -110 (c = 0.17, MeOH). The title compound proved to be too polar for the determination of the enantiomeric excess. The absolute configuration of (-)-fumimycin was determined by comparison of the optical rotation with that reported in the literature, and is also consistent with the X-ray diffraction analysis of **9**.

2.5 Mechanistic Studies

2.5.1 Racemization studies

(*S*)-**1a** was prepared according to the general procedure D using (*rac*)-**1a** (0.15 mmol) and **2a** (0.225 mmol) at -10 °C, and the crude reaction mixture was purified by flash column chromatography to afford (*S*)-**1a**.

(*S*)-**1d** was prepared according to the general procedure E using (*rac*)-**1d** (0.15 mmol) and **2a** (0.225 mmol) at -40 °C, and the crude reaction mixture was purified by flash column chromatography to afford (*S*)-**1a**.

In a Schlenk tube, $Cu(MeCN)_4BF_4$ (0.015 mmol, 10 mol%) and (*S*,*S*$_p$)-**L4** (0.018 mmol, 12 mol%) were stirred in 2 mL anhydrous THF under argon at 25 °C for 10 min. The Schlenk tube was placed at -10 °C or 10 °C. (*S*)-**1** (0.1 mmol), **2b** (0.1 mmol), and KOAc (0.2 mmol) were then added successively. The enantiomeric excess of **1** was measured at various times.

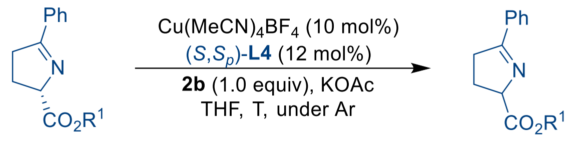

1a, R[1] = Me
1d, R[1] = 1-Nap

1

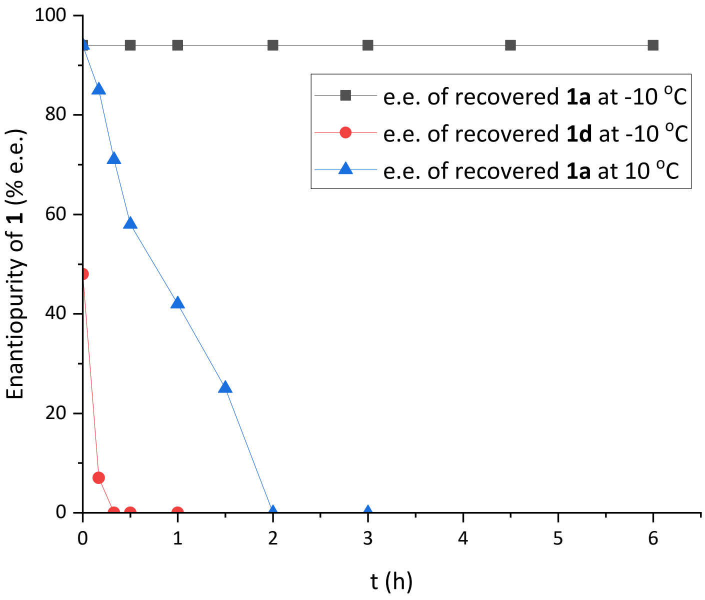

Time (h)	e.e. of recovered **1a** at -10 °C (%)	e.e. of recovered **1d** at -10 °C (%)	e.e. of recovered **1a** at 10 °C (%)
0	94	48	94
0.17	-	7	85
0.33	-	0	71
0.5	94	0	58
1	94	0	42
1.5			25
2	94	-	0
3	94	-	0
4.5	94	-	-
6	94	-	-

Supplementary Figure 1. Racemization profile of (*S*)-**1a** and (*S*)-**1d**.

2.5.2 Kinetic studies

We used [1]**H NMR** spectroscopy to monitor the reaction progress to determine the possible interconversion of **1d**, **2a**, **4a**, and **2a'**. Under the standard conditions, all compound was prepared according to the general procedure E and ended the reaction at different times. The crude reaction mixture was purified by flash column chromatography to afford all compounds. The yield of all compounds was determined by ^{1}**H NMR** analysis using 1,3,5-trimethoxybenzene.

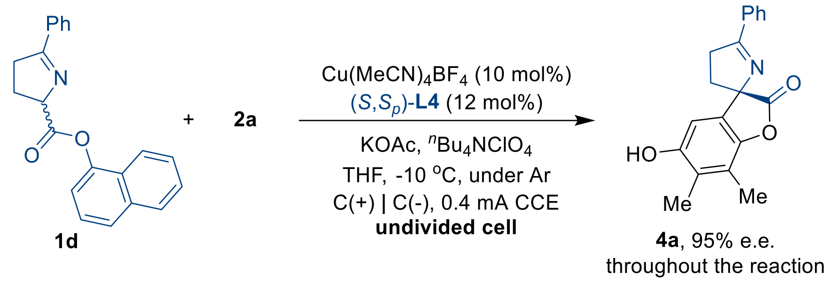

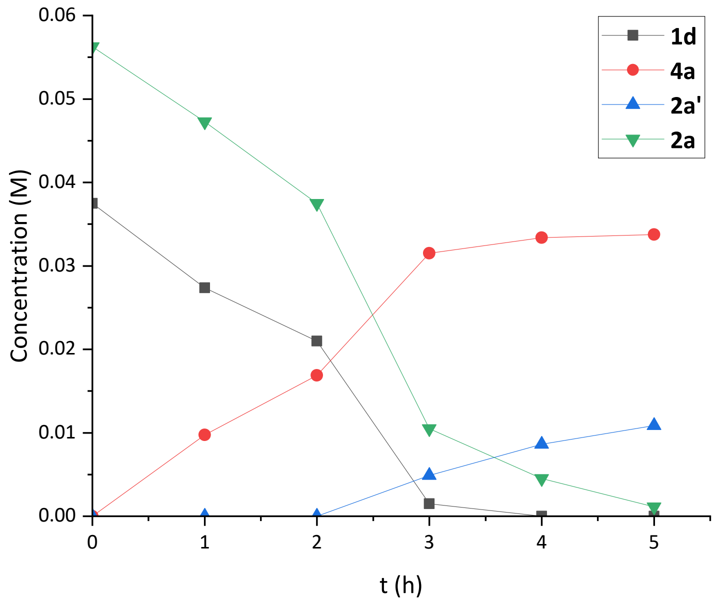

t(h)	**1d** (M)	**4a** (M)	**2a'** (M)	**2a** (M)
0	0.0375	0	0	0.0563
1	0.0274	0.0098	0	0.0473
2	0.021	0.0169	0	0.0375
3	0.0015	0.0315	0.0049	0.0105
4	0	0.0334	0.0086	0.0045
5	0	0.0338	0.0109	0.0011

Supplementary Figure 2. Kinetic profile of the reaction.

In a 5 mL Schlenk tube, $Cu(MeCN)_4BF_4$ (0.015 mmol, 10 mol%) and (*S*, S_p)-**L4** (0.018 mmol 12 mol%) were stirred in 2 mL anhydrous THF under argon at 25 °C for 10 min.

1d (0.15 mmol) and KOAc (0.3 mmol) were then added successively. The Schlenk tube was carried out at -10 °C. **2a’** in 2 mL THF was injected into the Schlenk tube through a syringe pump at various times. The solvent was removed under reduced pressure. The residue was purified by silica gel chromatography to afford the desired product **4a**.

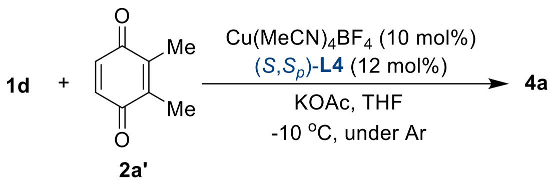

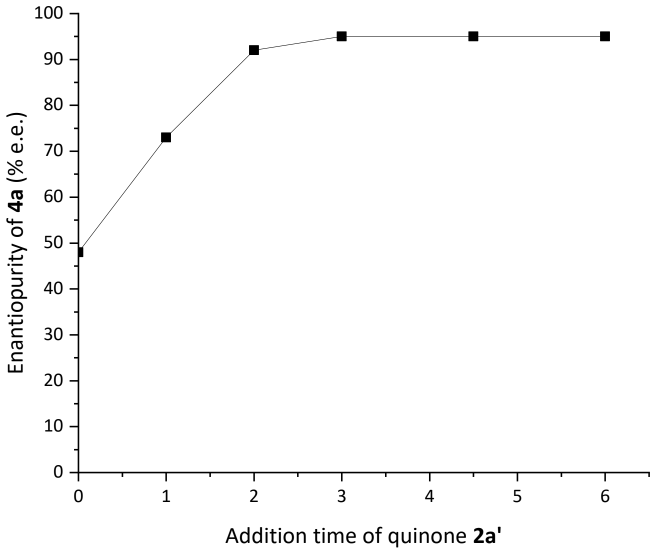

t (h)	e.e. of **4a** (%)
0	48
1	73
2	92
3	95
4.5	95
6	95

Supplementary Figure 3. Effect of addition time of quinone **2a’** on the e.e. values of **4a**.

2.5.3 Control experiments with 4-aminophenols as substrates

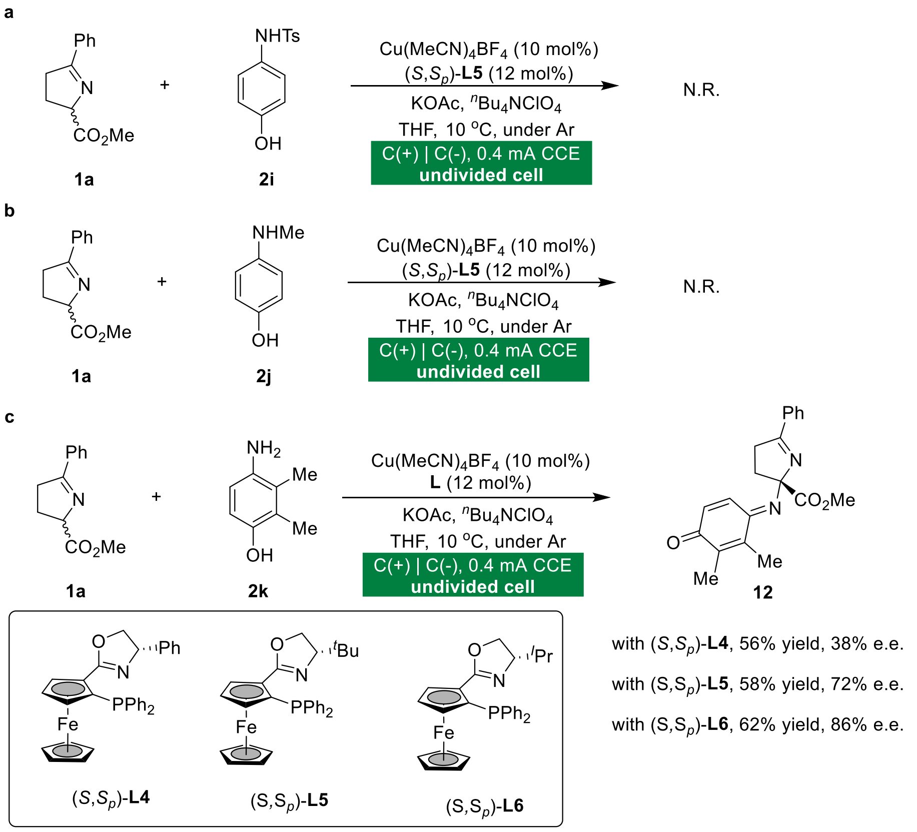

Supplementary Figure 4. Evaluation of 4-aminophenols as the reaction partner. The utilization of different 4-aminophenol partners exhibited a significant impact on reactivity (a-c). No reaction was observed when employing **2i** and **2j** as substrates. Encouragingly, **2k** was found to be an appropriate reaction partner. The desired product **12** with a yield of 56% and an enantiomeric excess (e.e.) of 38% was obtained while utilizing (*S*,*S*$_p$)-**L4** as the chiral ligand. Several chiral PHOX ligands were evaluated to promote the reaction, and the desired product **12** could be obtained in good yield and enantioselectivity (62% yield, 86% e.e.) with (*S*,*S*$_p$)-**L6**.

Methyl (*S*,*E*)-2-((2,3-dimethyl-4-oxocyclohexa-2,5-dien-1-ylidene)amino)-5-phenyl-3,4-dihydro-2H-pyrrole-2-carboxylate (12)

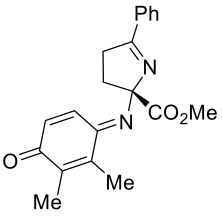

The title compound was prepared according to the general procedure D using **1a** (0.15 mmol), **2k** (0.225 mmol), and (*S*,*S*$_p$)-**L6** as ligand at 10 °C. The crude reaction mixture was purified by flash column chromatography (petroleum ether/ethyl acetate, 6:1) to afford the title compound as a yellow oil (62%). **^{1}H NMR (600 MHz, CDCl$_3$)** δ 7.93 – 7.89 (m, 2H), 7.68 (d, J = 10.3 Hz, 1H), 7.50 – 7.45 (m, 1H), 7.45 – 7.39 (m, 2H), 6.45 (d, J = 10.3 Hz, 1H), 3.75 (s, 3H), 3.25 – 3.12 (m, 2H), 3.04 – 2.96 (m, 1H), 2.55 – 2.47 (m, 1H), 2.19 (s, 3H), 2.01 (s, 3H). **^{13}C NMR (151 MHz, CDCl$_3$)** δ 187.38, 176.24, 172.22, 160.17, 145.41, 136.75, 133.55, 131.65, 131.10, 130.86, 128.65, 128.53, 98.23, 53.20, 37.52, 35.03, 14.40, 12.26. **ESI-MS**: calculated [$C_{20}H_{20}N_2O_3$ + H]$^+$: 337.1547, found: 337.1551. $[\alpha]^{20}_D$ = -198.5 (c = 0.18, CH_2Cl_2). The product was analyzed by HPLC to determine the enantiomeric excess: 86% e.e. (CHIRALPAK AD-H, hexane/*i*-PrOH = 70/30, detector: 268 nm, T = 25 °C, flow rate: 1 mL/min), t$_1$(major minor) = 5.10 min, t$_2$(minor) = 6.06 min.

2.6 Cyclic Voltammetry Studies

Unless otherwise noted, the cyclic voltammograms were recorded on a CHI 760E instrument using a glassy carbon disk working electrode (diameter, 3 mm), a Pt wire auxiliary electrode, an SCE reference electrode, and a scan rate of 100 mV/s.

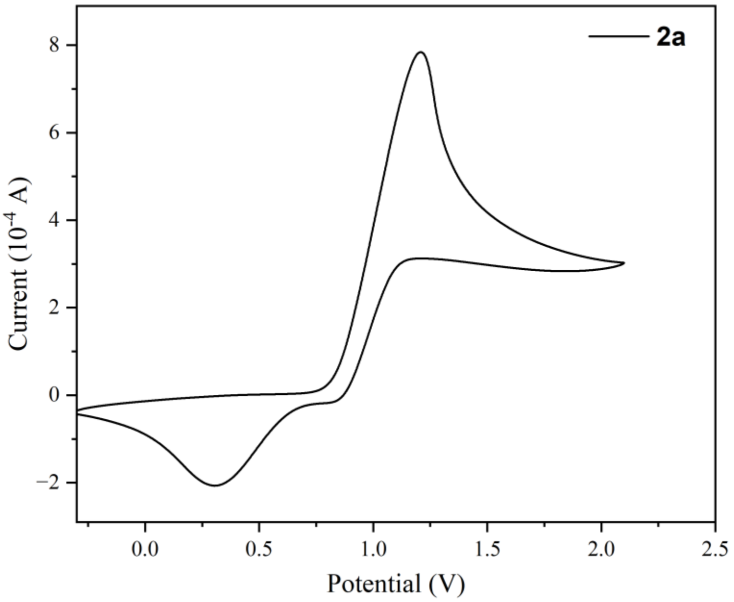

Supplementary Figure 5. Cyclic voltammogram of **2a** (20 mM) in an electrolyte of $^{n}Bu_4NClO_4$ (0.07 M) in MeCN. The oxidation peak of **2a** was observed at 1.21 V (vs SCE).

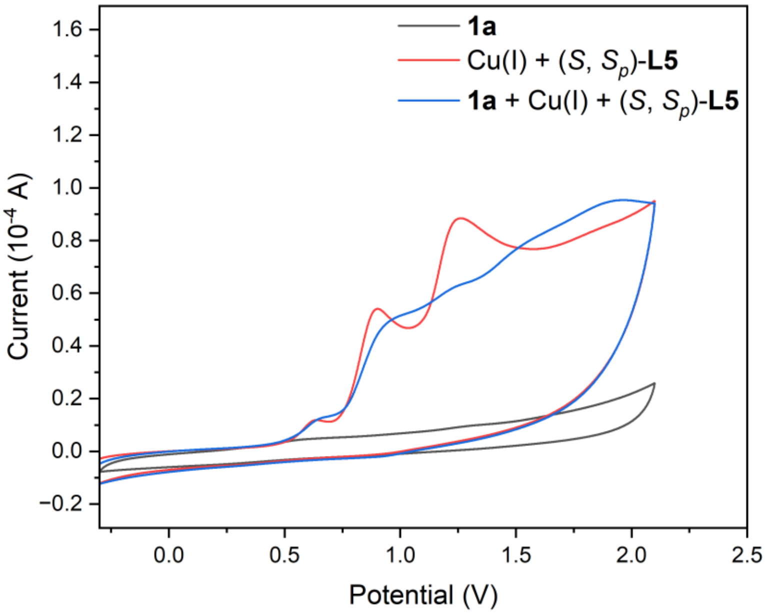

Supplementary Figure 6. Cyclic voltammogram of **1a** (20 mM), $Cu(CH_3CN)_4BF_4$ (10 mM), and (*S*,*S*$_p$)-**L5** (12 mM) in an electrolyte of $^{n}Bu_4NClO_4$ (0.07 M) in MeCN. The oxidation peak of **1a** was not observed. The new oxidation peak of **1a** was not observed when $Cu(CH_3CN)_4BF_4$ and (*S*,*S*$_p$)-**L5** were present.

2.7 Cyclic Voltammetry Studies

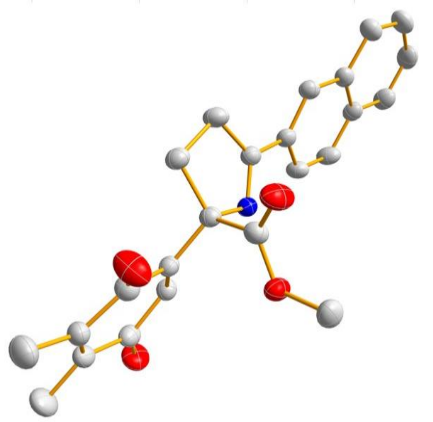

3j
(CCDC 2249495)

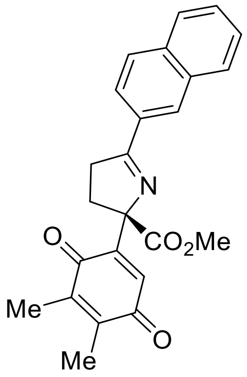

3j

Chemical formula	$C_{24}H_{21}NO_4$
Formula weight	387.42
Space group	P 1 21 1
Z	4
α, Å	17.535(4)
b, Å	6.0643(12)
c, Å	20.300(3)
α, °	90.000
β, °	109.003(10)
γ, °	90.000
V, $Å^3$	2040.9(7)

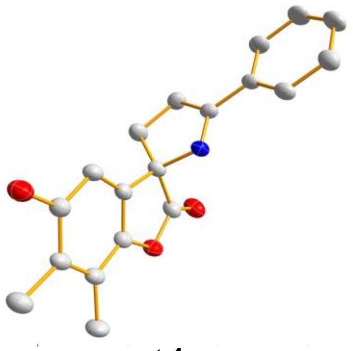

ent-**4a**
(CCDC 2249496)

≡

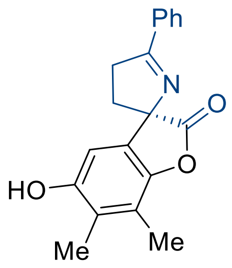

ent-**4a**

Chemical formula	$C_{19}H_{17}NO_3$
Formula weight	316.34
Space group	C 2 2 21
Z	8
α, Å	7.1845(2)
b, Å	26.5672(8)
c, Å	17.0552(5)
α, °	90.000
β, °	90.000
γ, °	90.000
V, $Å^3$	3255.36(16)

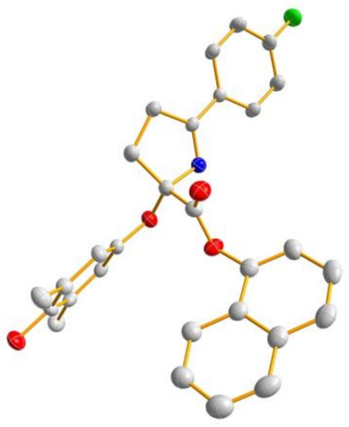

5b
(CCDC 2249497)

≡

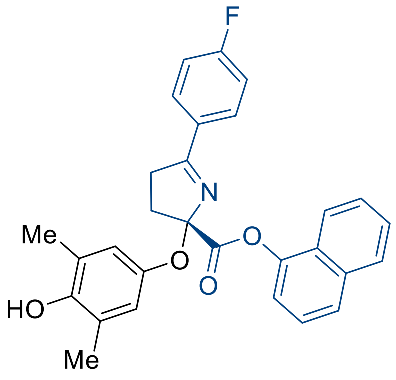

5b

Chemical formula	$C_{29}H_{24}FNO_4$
Formula weight	469.49
Space group	P 1 21 1
Z	4
α, Å	8.1851(5)
b, Å	9.5931(6)
c, Å	29.6470(16)
α, °	90.000
β, °	94.912(4)
γ, °	90.000
V, $Å^3$	2319.3(2)

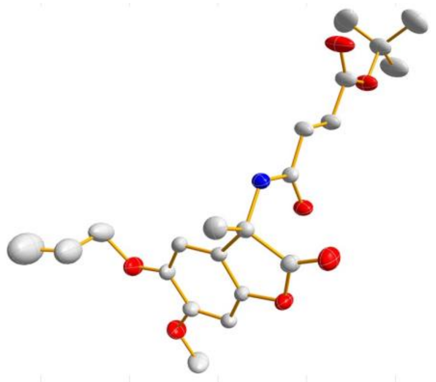

9
(CCDC 2249498)

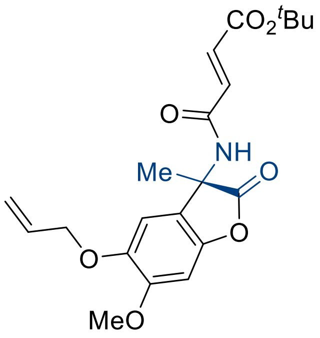

9

Chemical formula	$C_{21}H_{25}NO_7$
Formula weight	403.42
Space group	P 21 21 21
Z	8
α, Å	11.785(8)
b, Å	18.436(13)
c, Å	20.766(15)
α, o	90.000
β, o	90.000
γ, o	90.000
V, Å^3	4512(5)

3. Supplementary Figure

3.1 NMR spectra

^{1}H NMR of 3a

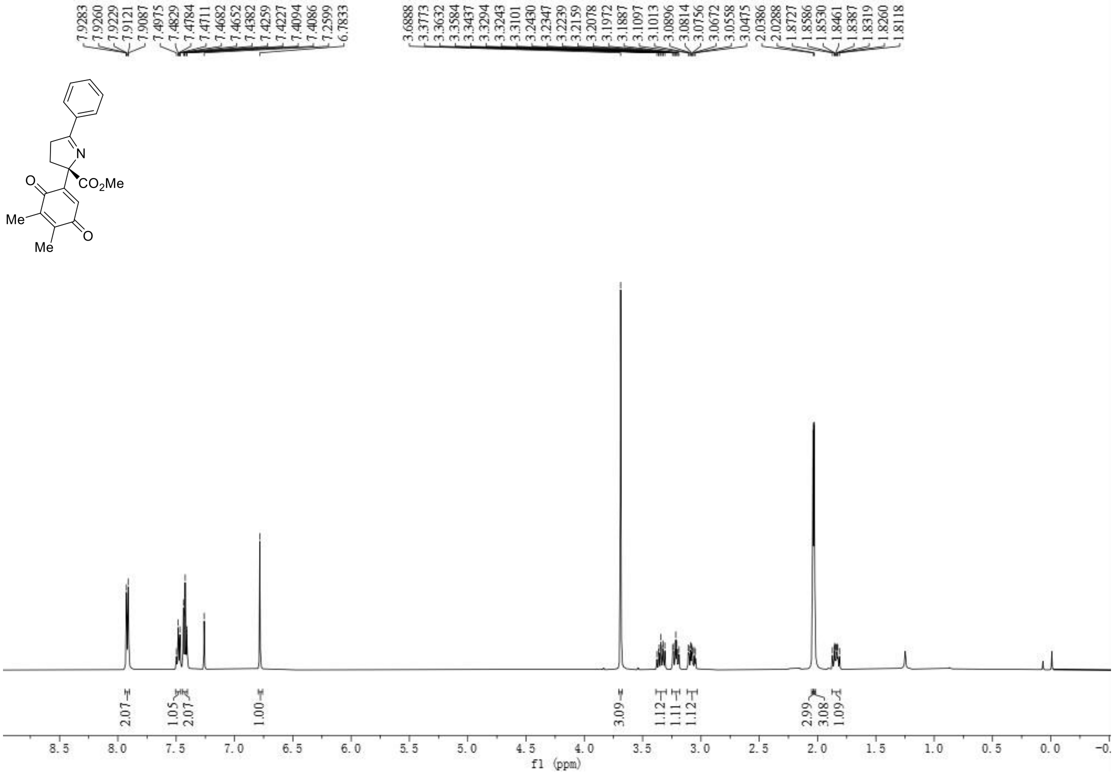

^{13}C NMR of 3a

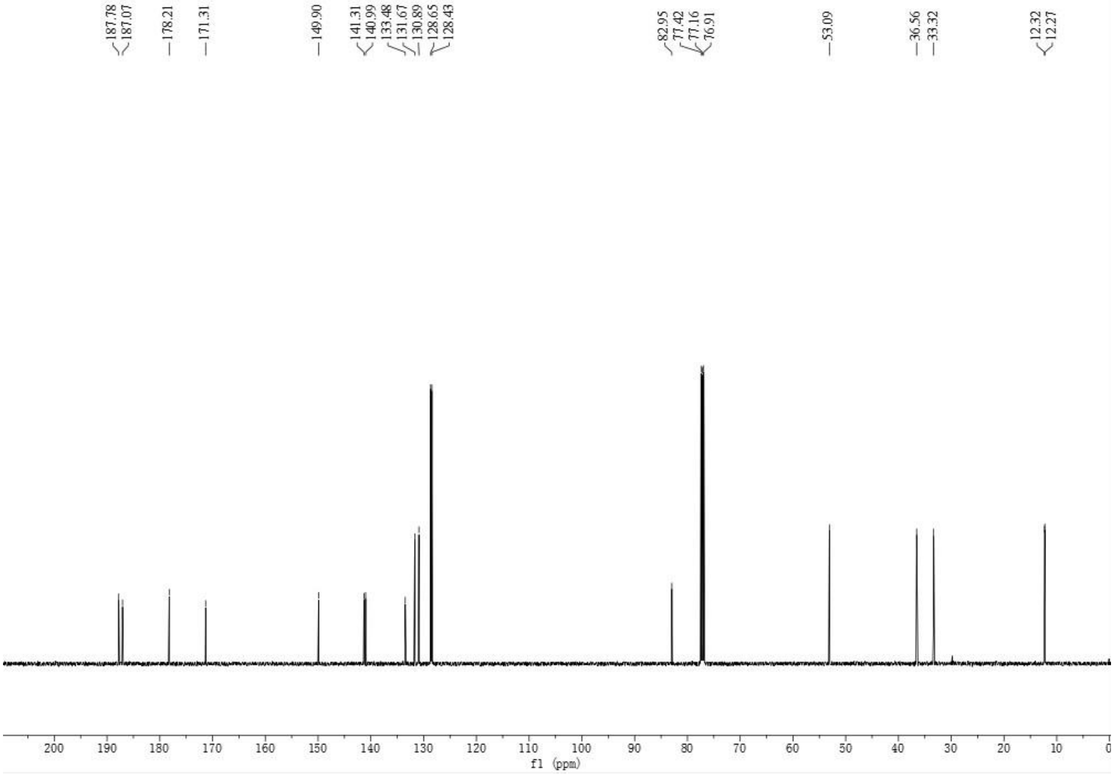

Supplementary Figure 7. ^{1}H NMR, and ^{13}C NMR spectra of compound **3a**

^{1}H NMR of 3b

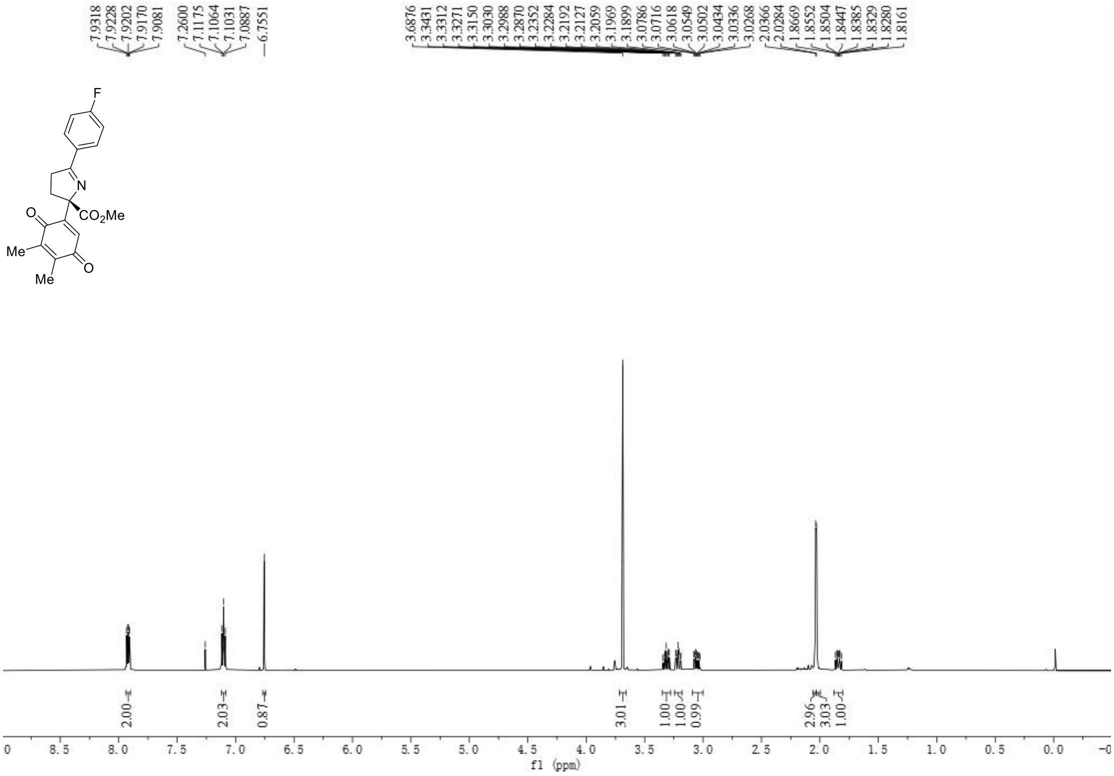

^{19}F NMR of 3b

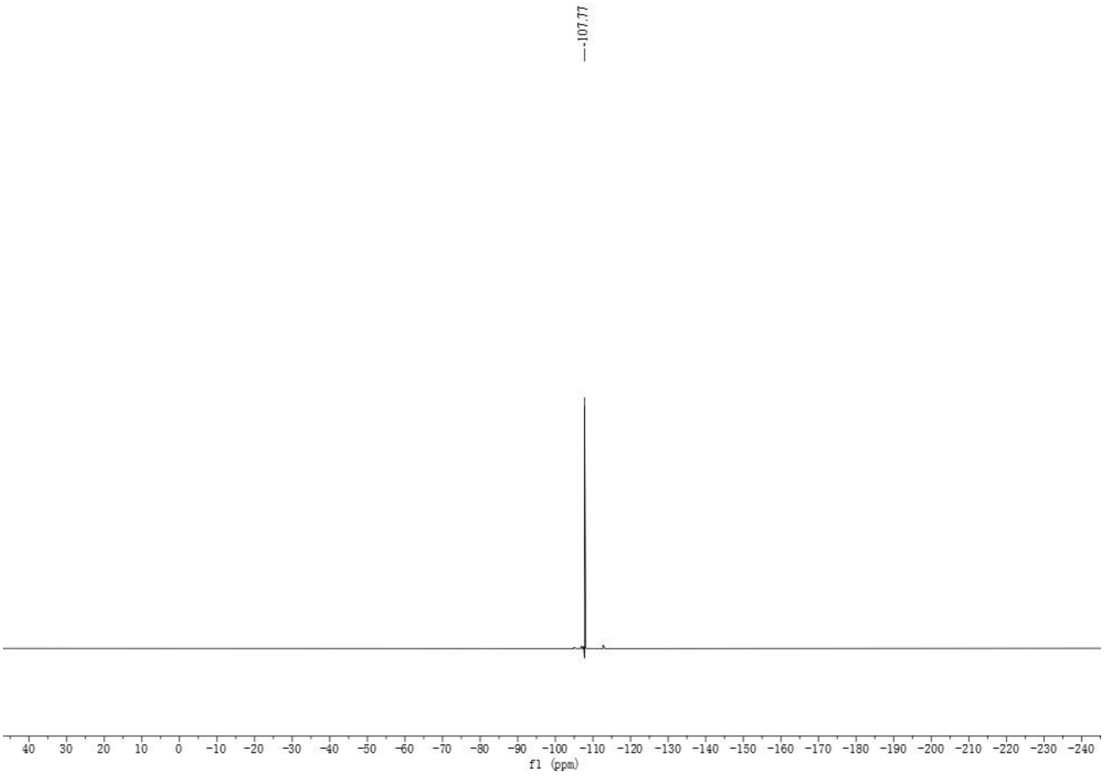

^{13}C NMR of 3b

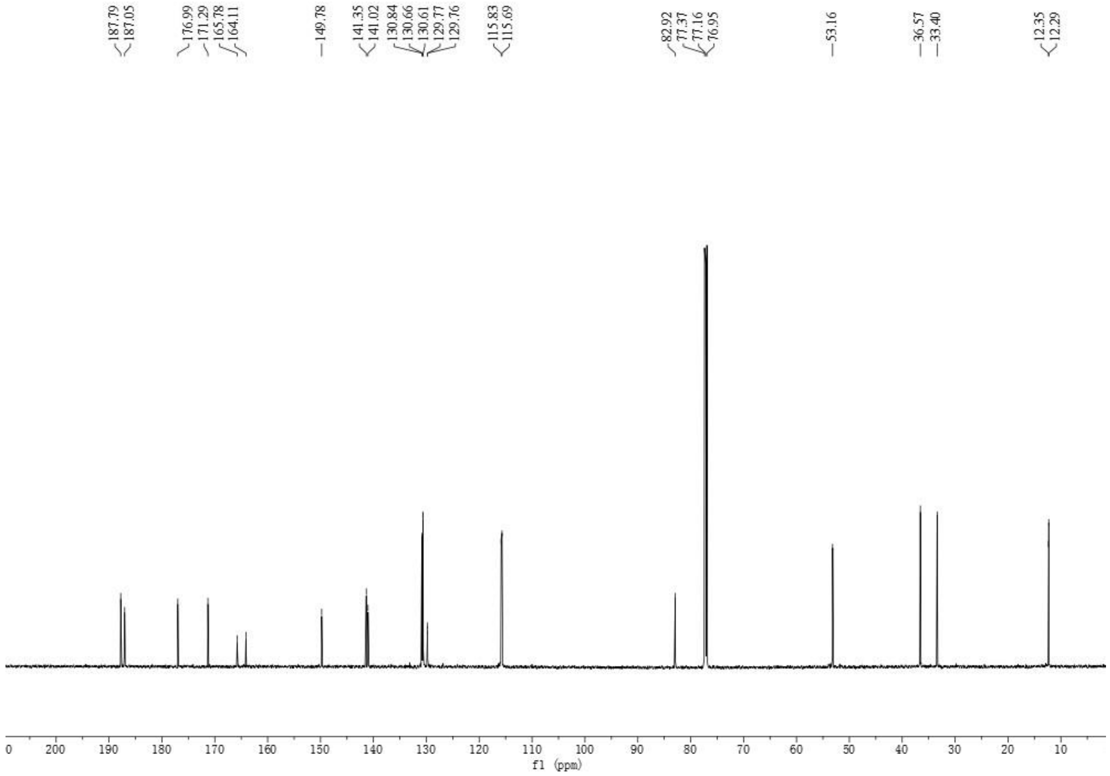

Supplementary Figure 8. ^{1}H NMR, ^{13}C NMR, and ^{19}F NMR spectra of compound **3b**

^{1}H NMR of 3c

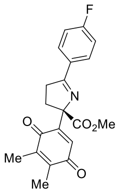

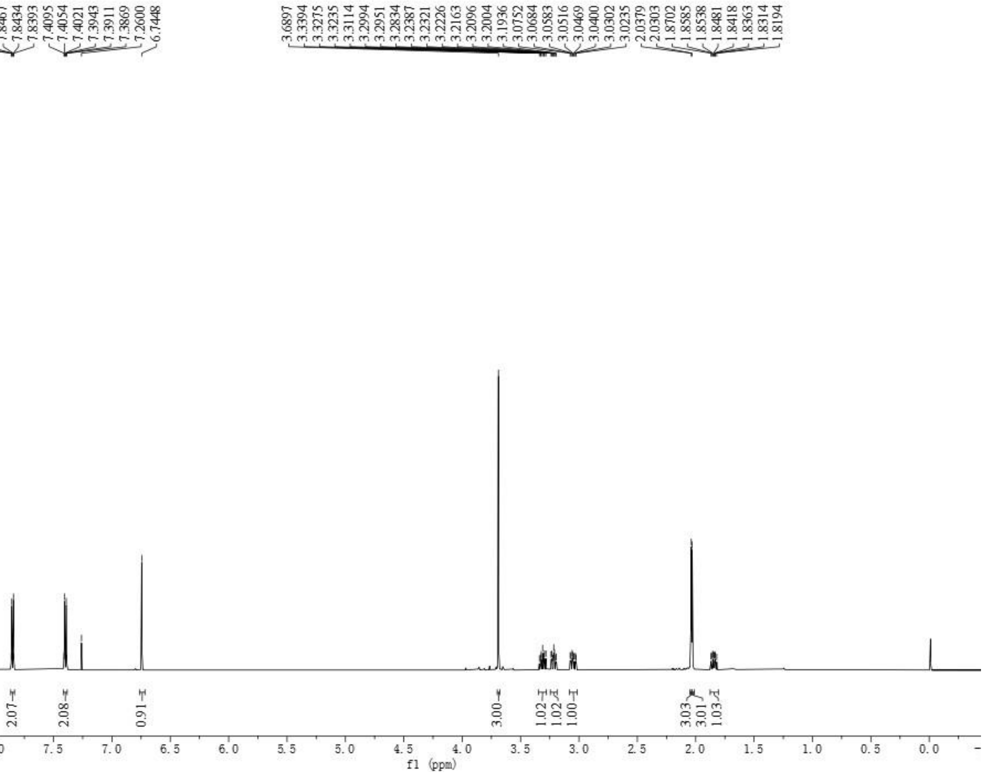

^{13}C NMR of 3c

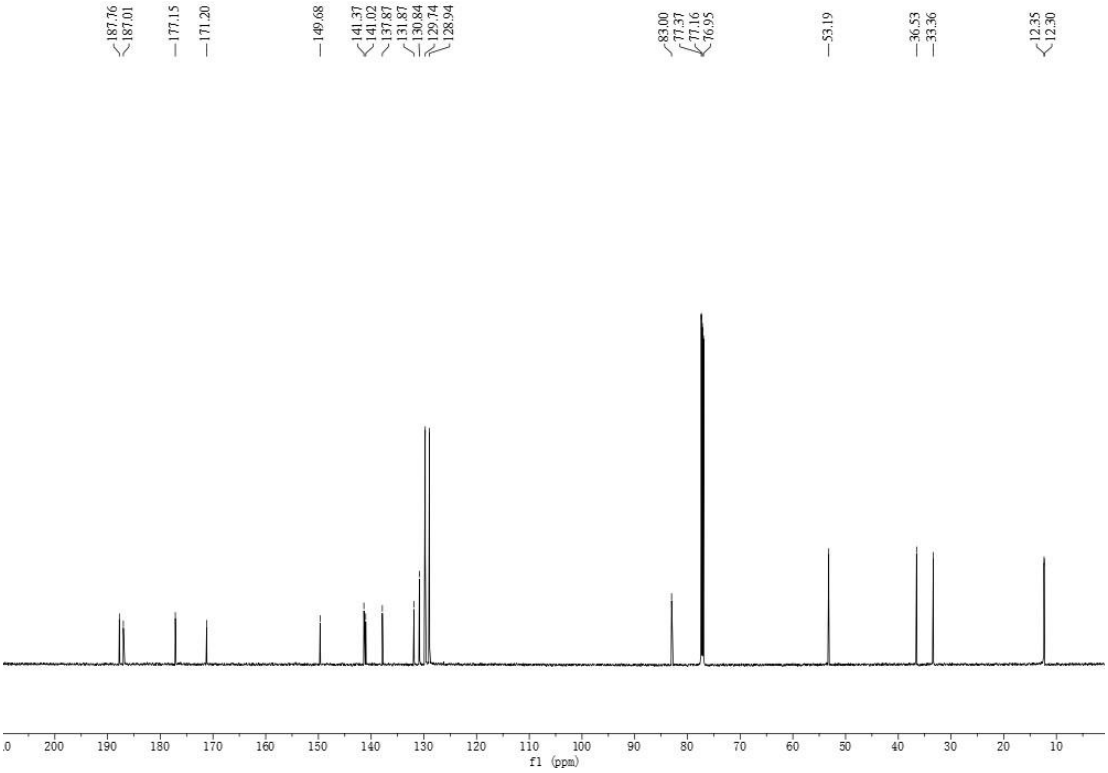

Supplementary Figure 9. ^{1}H NMR, and ^{13}C NMR spectra of compound **3c**

^{1}H NMR of 3d

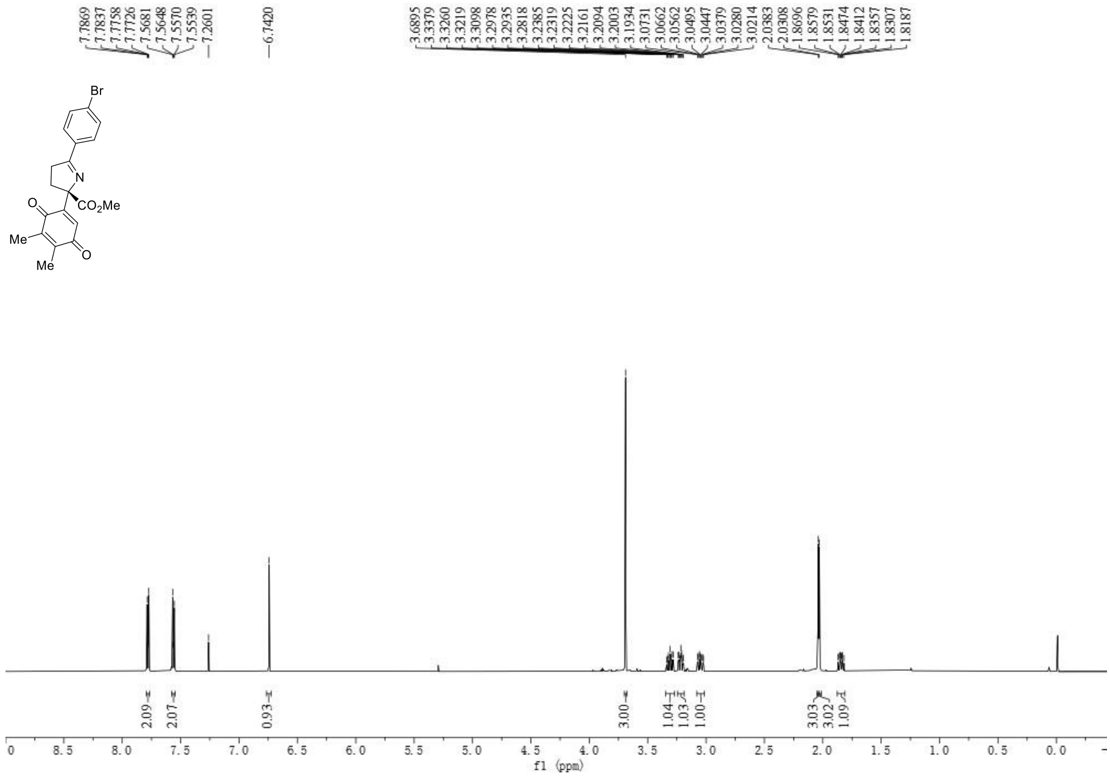

^{13}C NMR of 3d

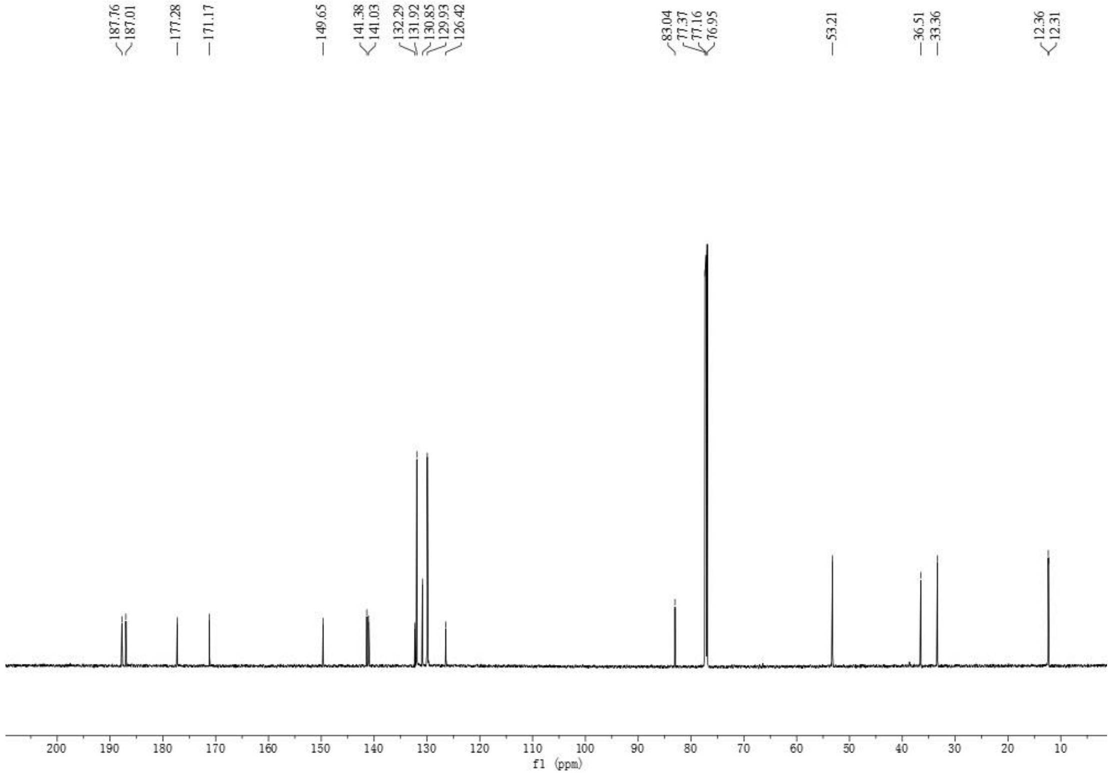

Supplementary Figure 10. ^{1}H NMR, and ^{13}C NMR spectra of compound **3d**

^{1}H NMR of 3e

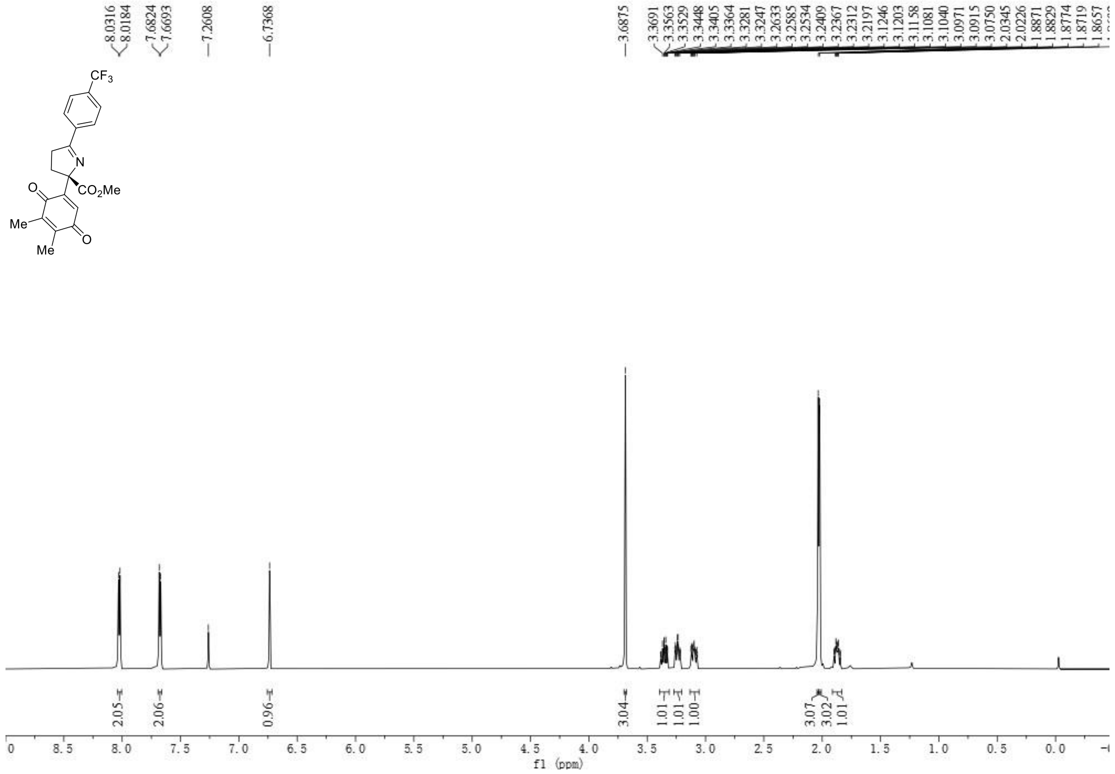

^{19}F NMR of 3e

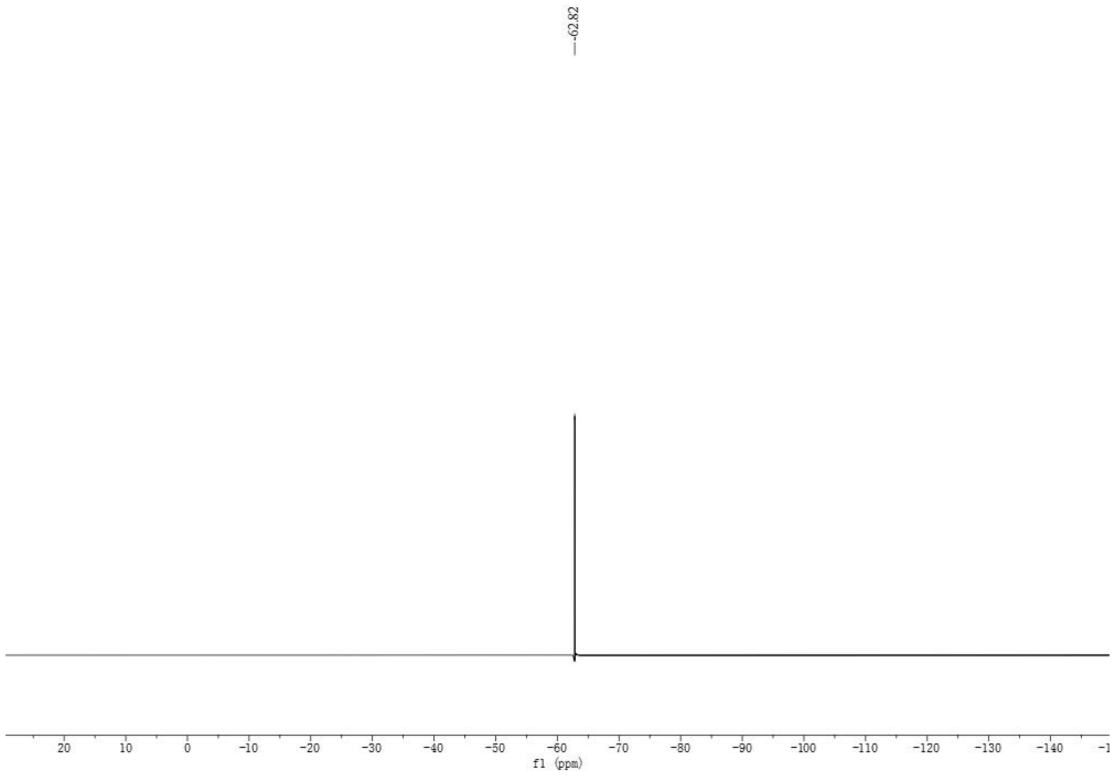

^{13}C NMR of 3e

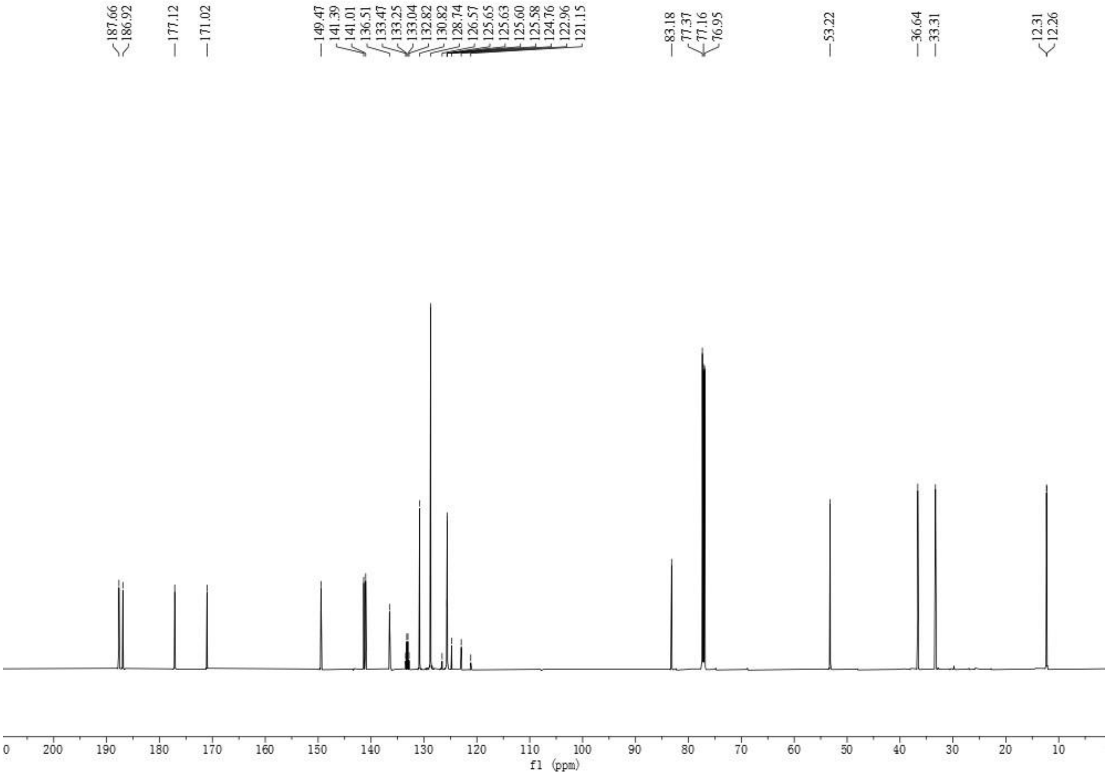

Supplementary Figure 11. ^{1}H NMR, ^{13}C NMR, and ^{19}F NMR spectra of compound **3e**

1H NMR of 3f

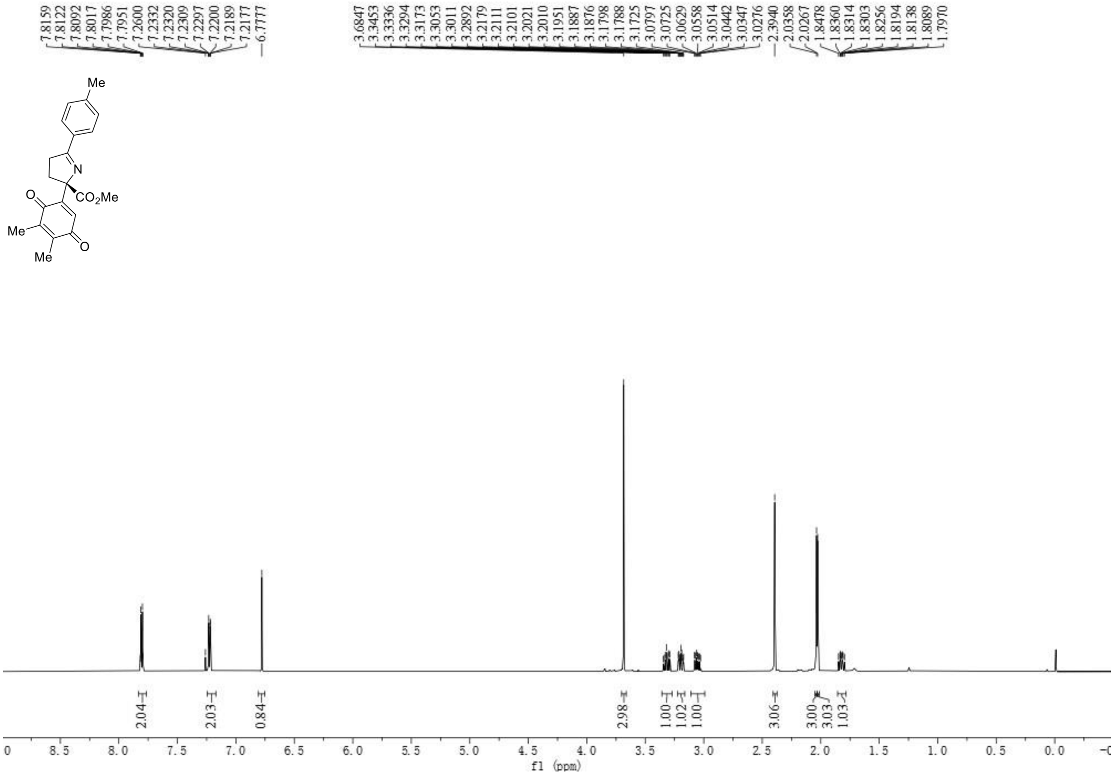

13C NMR of 3f

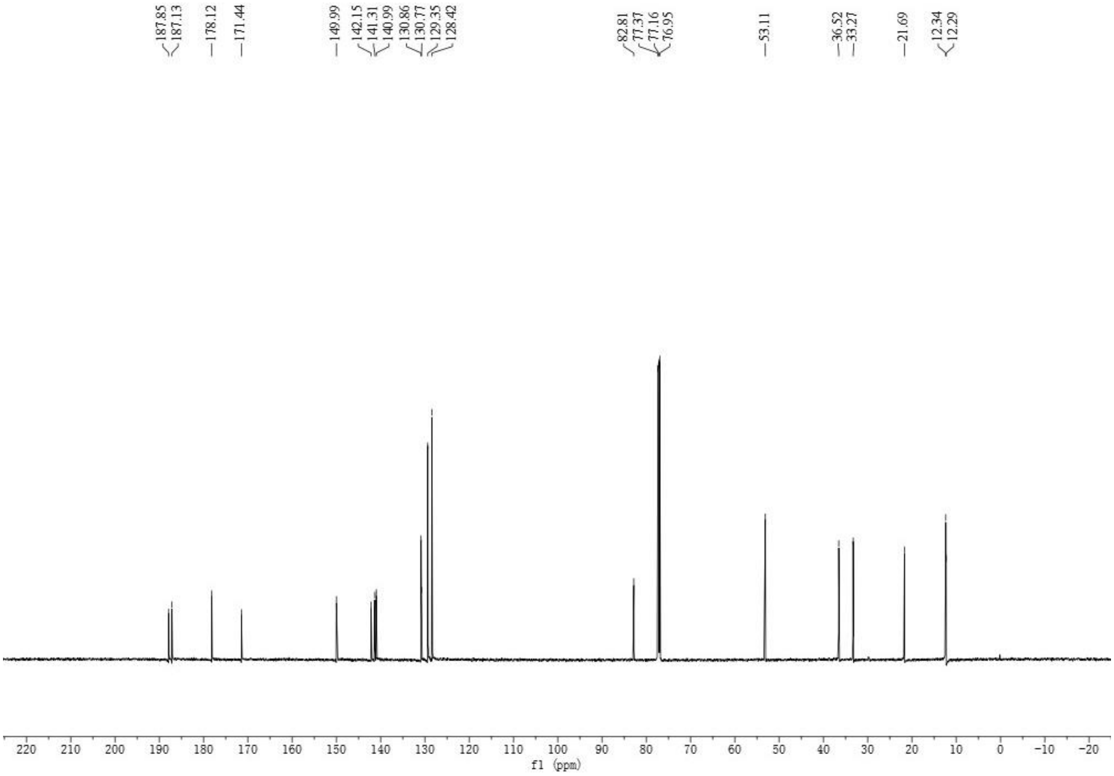

Supplementary Figure 12. ^{1}H NMR, and ^{13}C NMR spectra of compound **3f**

^{1}H NMR of 3g

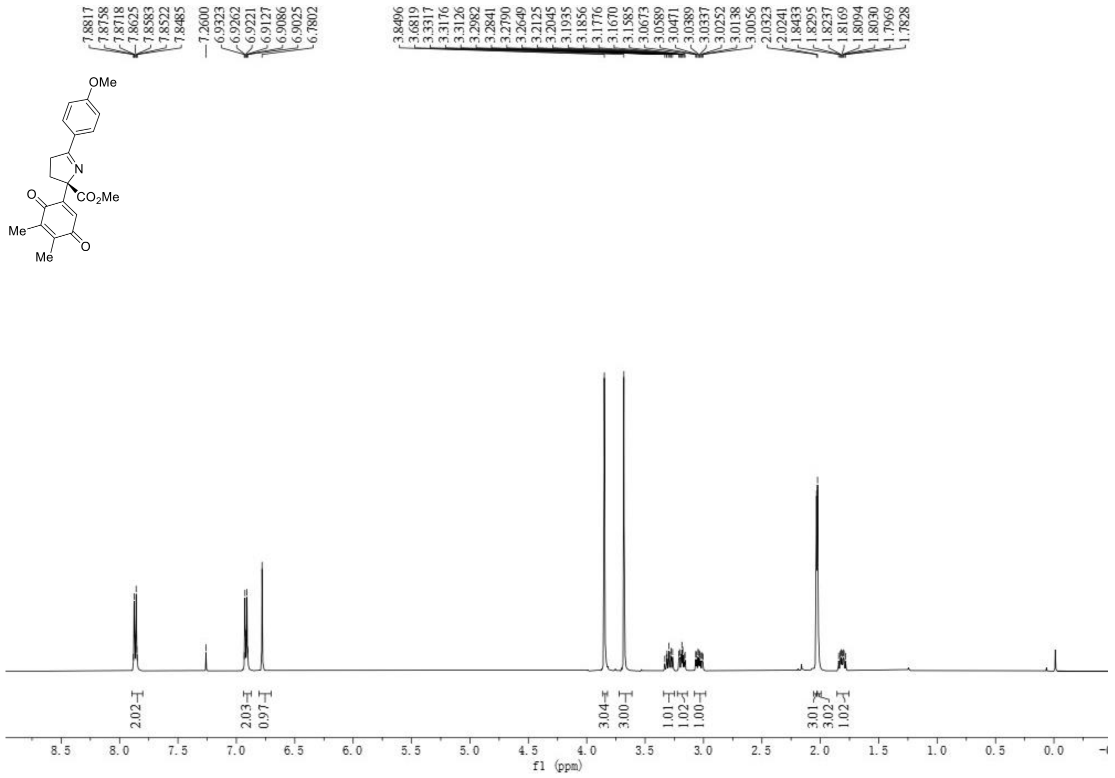

^{13}C NMR of 3g

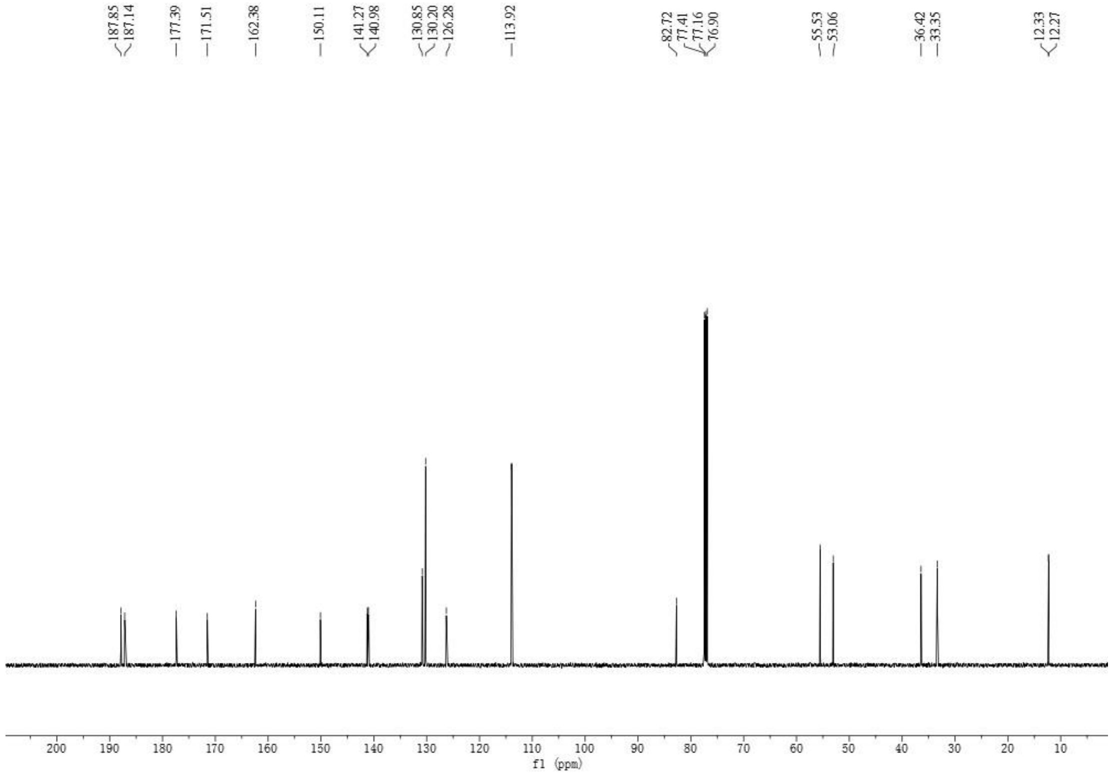

Supplementary Figure 13. ^{1}H NMR, and ^{13}C NMR spectra of compound **3g**

^{1}H NMR of 3h

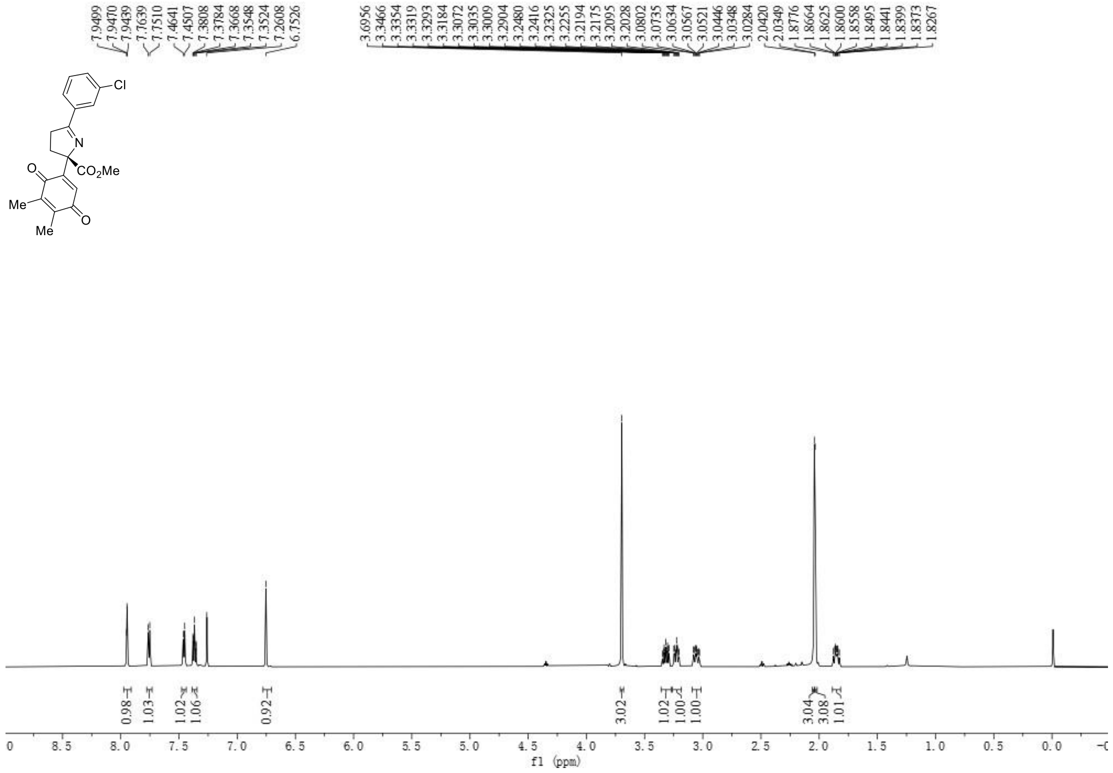

^{13}C NMR of 3h

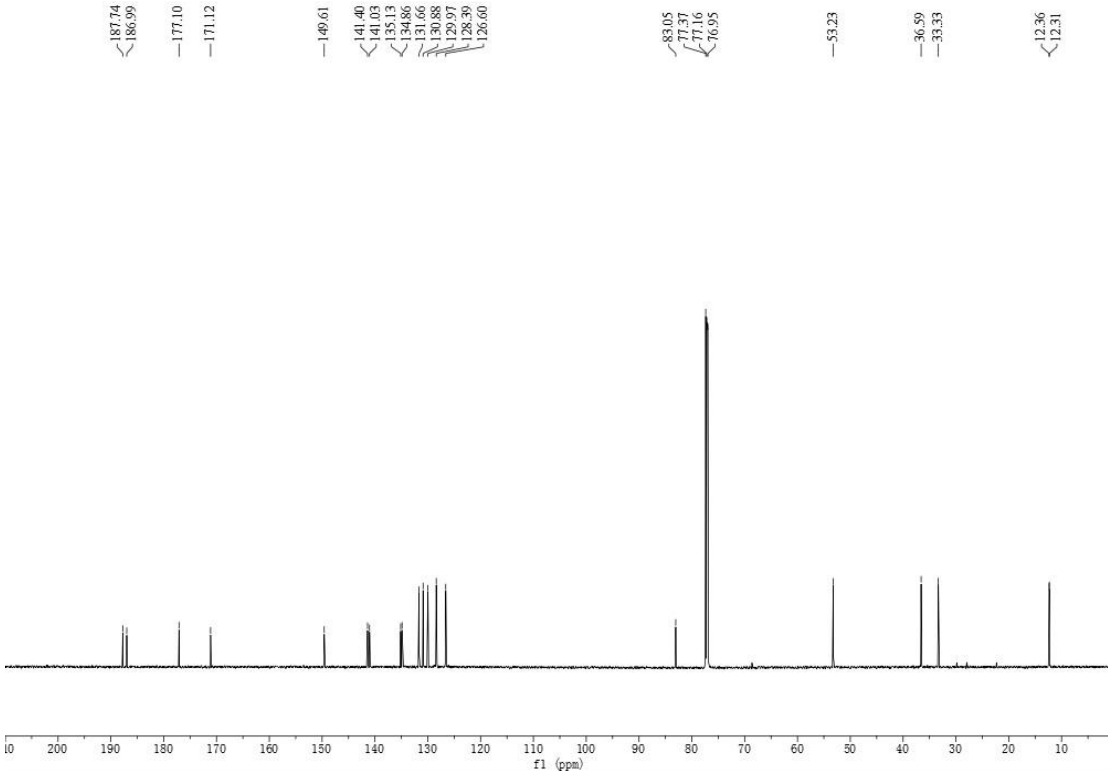

Supplementary Figure 14. ^{1}H NMR, and ^{13}C NMR spectra of compound **3h**

^{1}H NMR of 3i

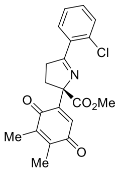

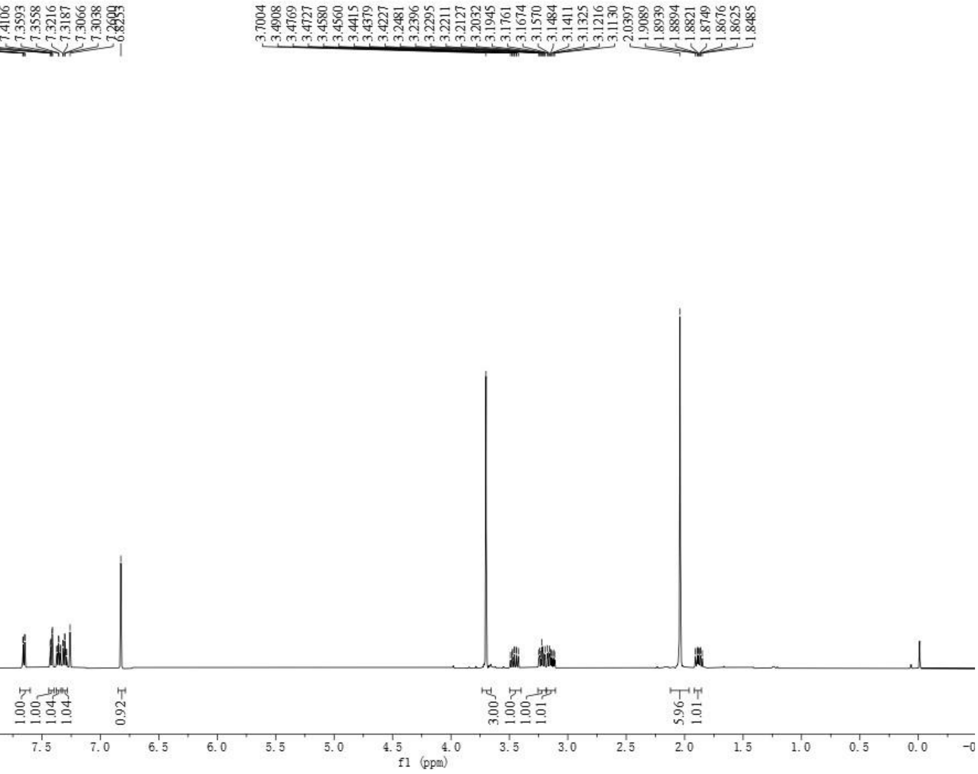

^{13}C NMR of 3i

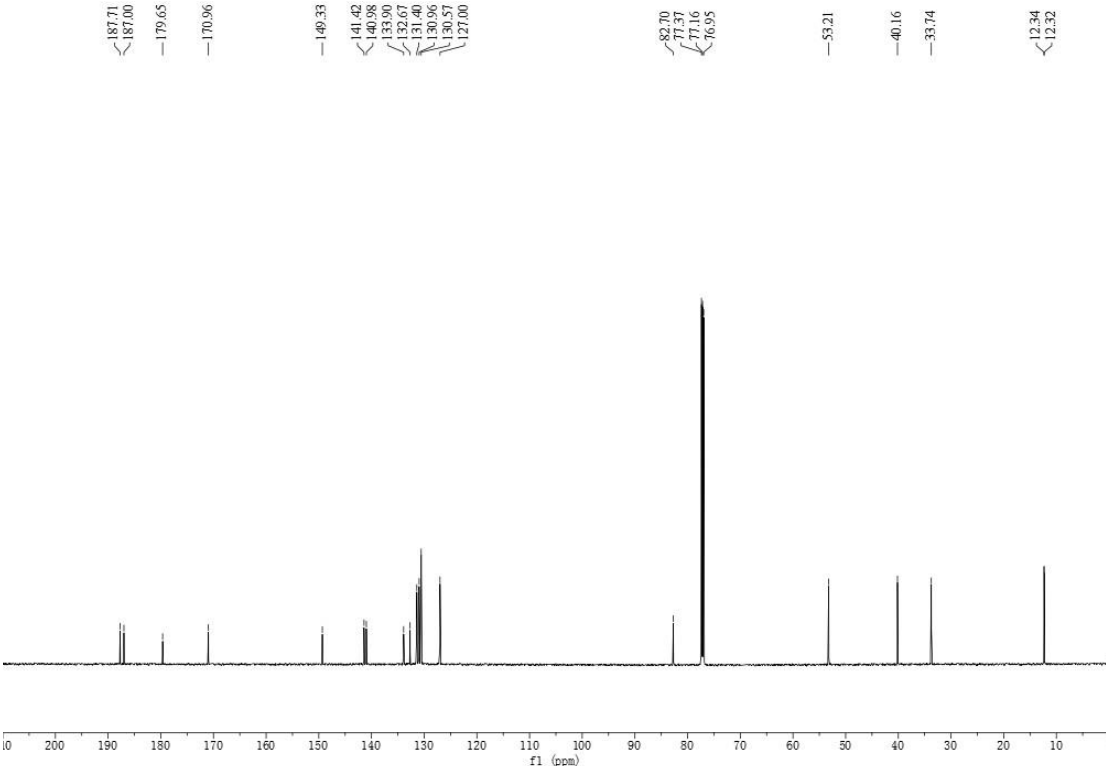

Supplementary Figure 15. ^{1}H NMR, and ^{13}C NMR spectra of compound **3i**

^{1}H NMR of 3j

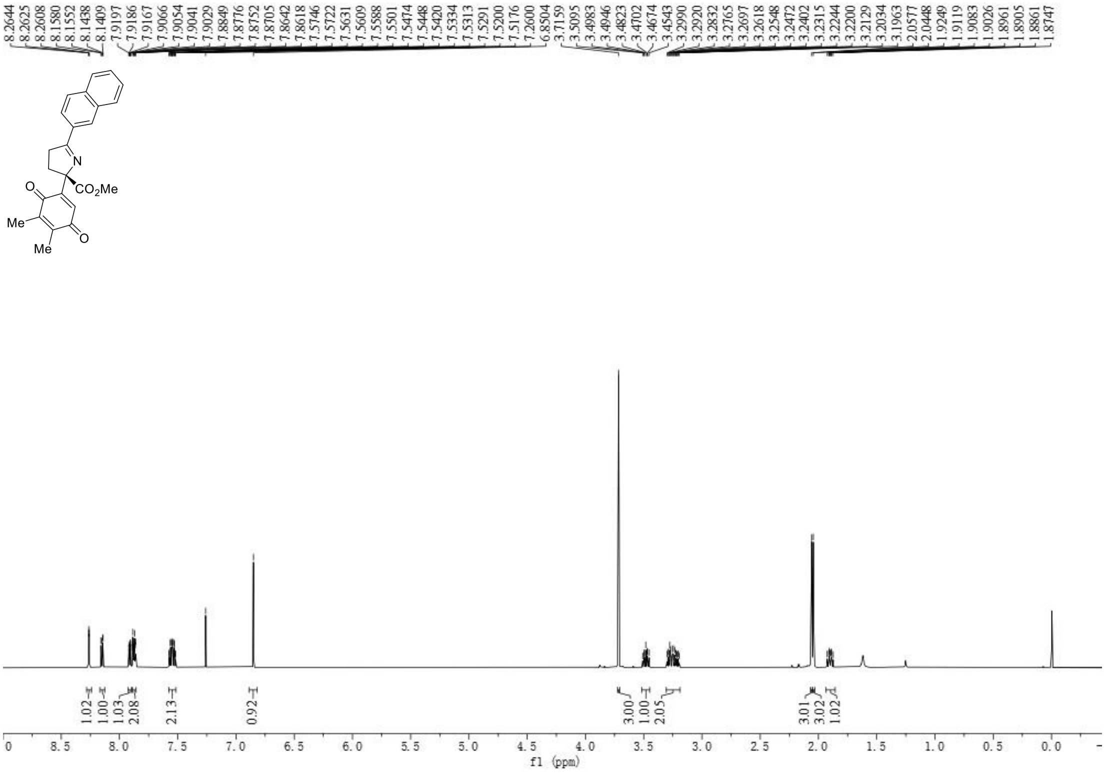

^{13}C NMR of 3j

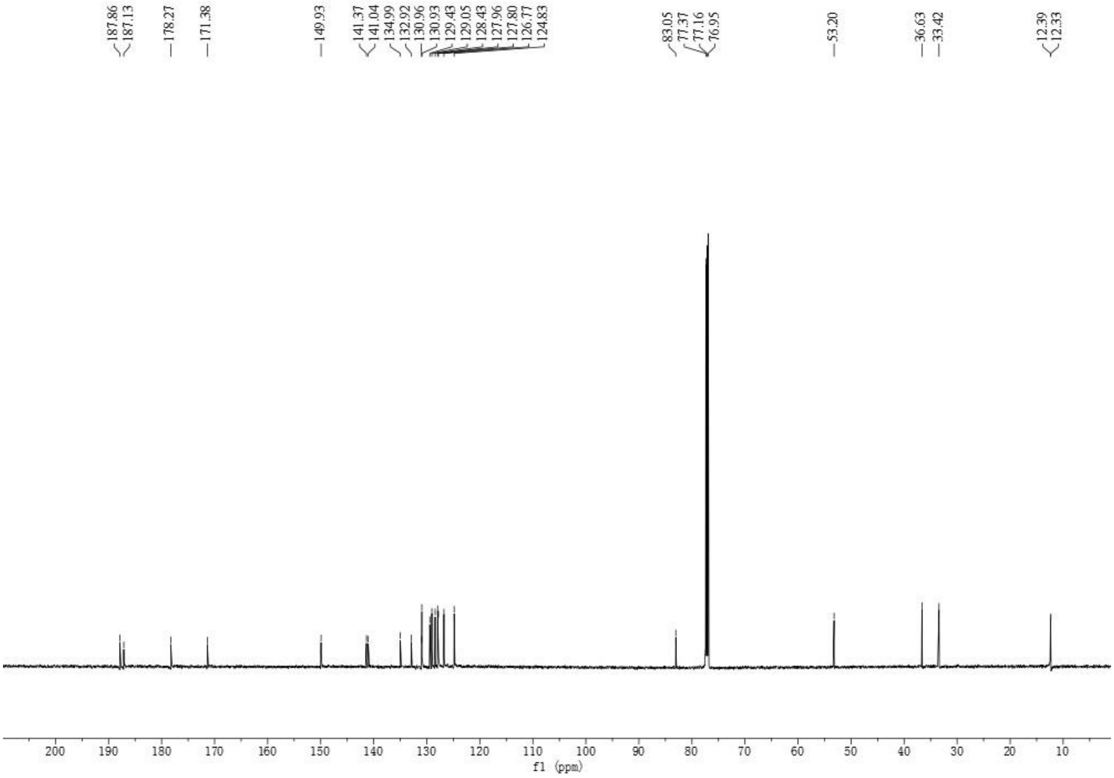

Supplementary Figure 16. ^{1}H NMR, and ^{13}C NMR spectra of compound **3j**

^{1}H NMR of 3k

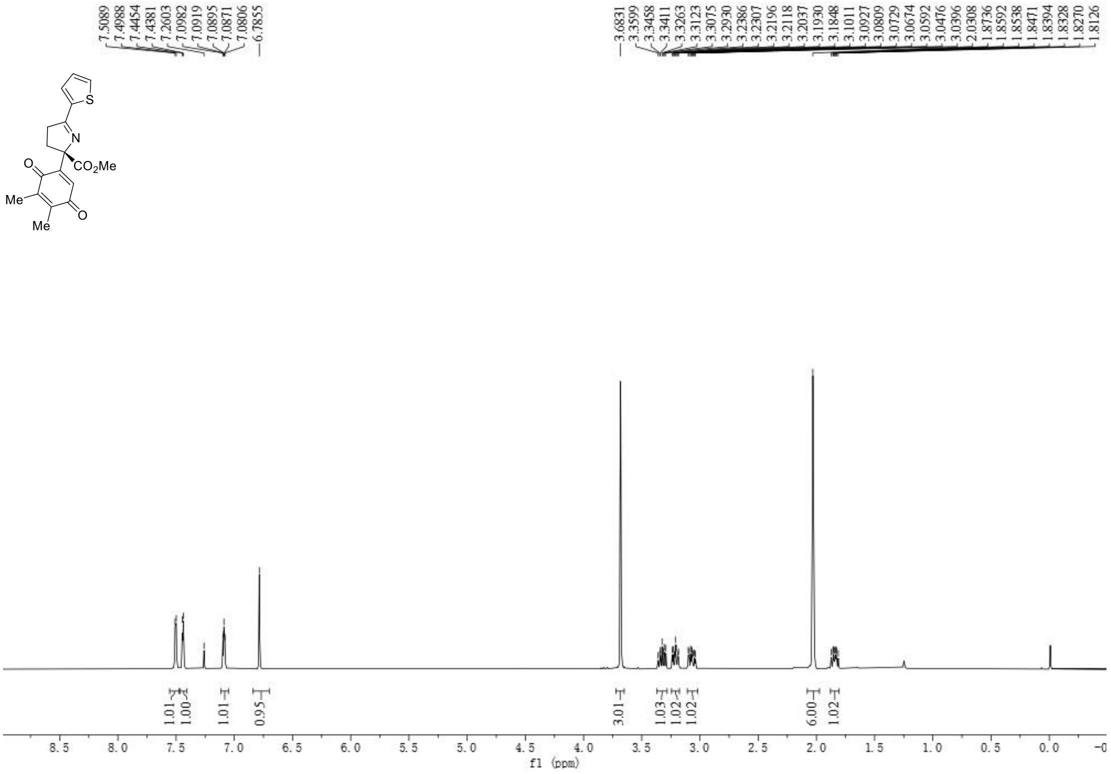

^{13}C NMR of 3k

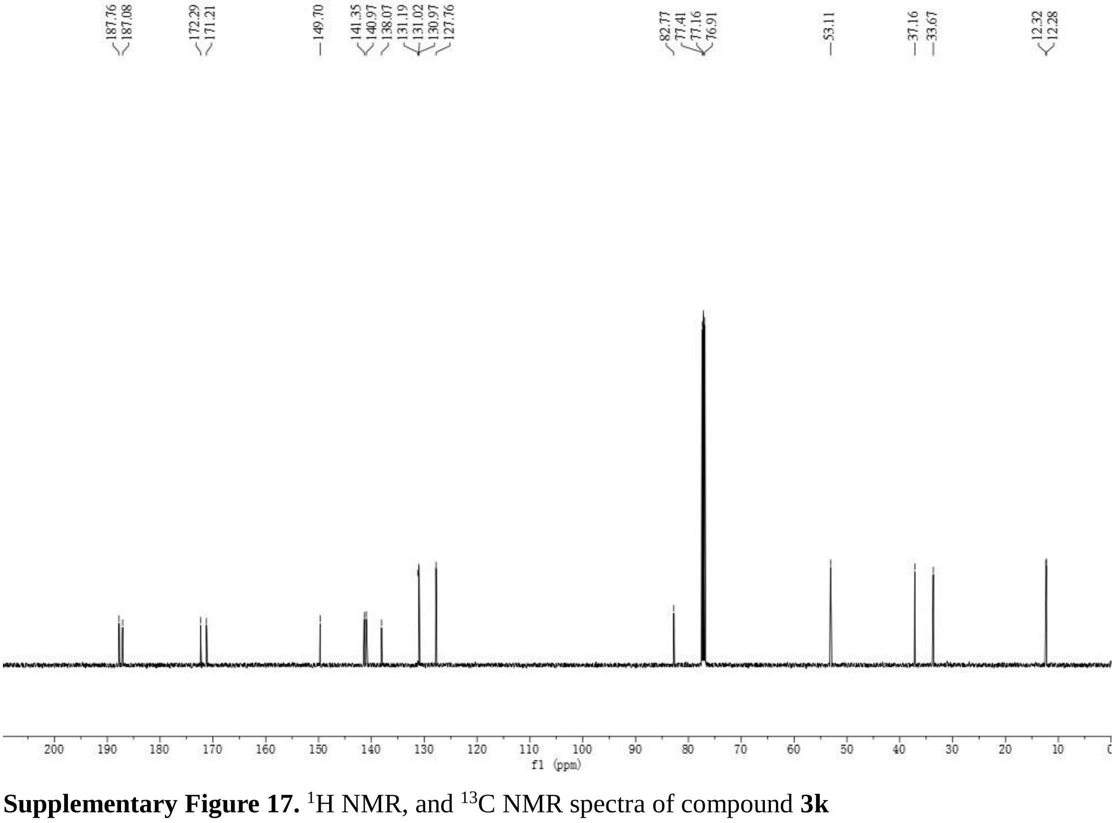

Supplementary Figure 17. ^{1}H NMR, and ^{13}C NMR spectra of compound **3k**

^{1}H NMR of 3l

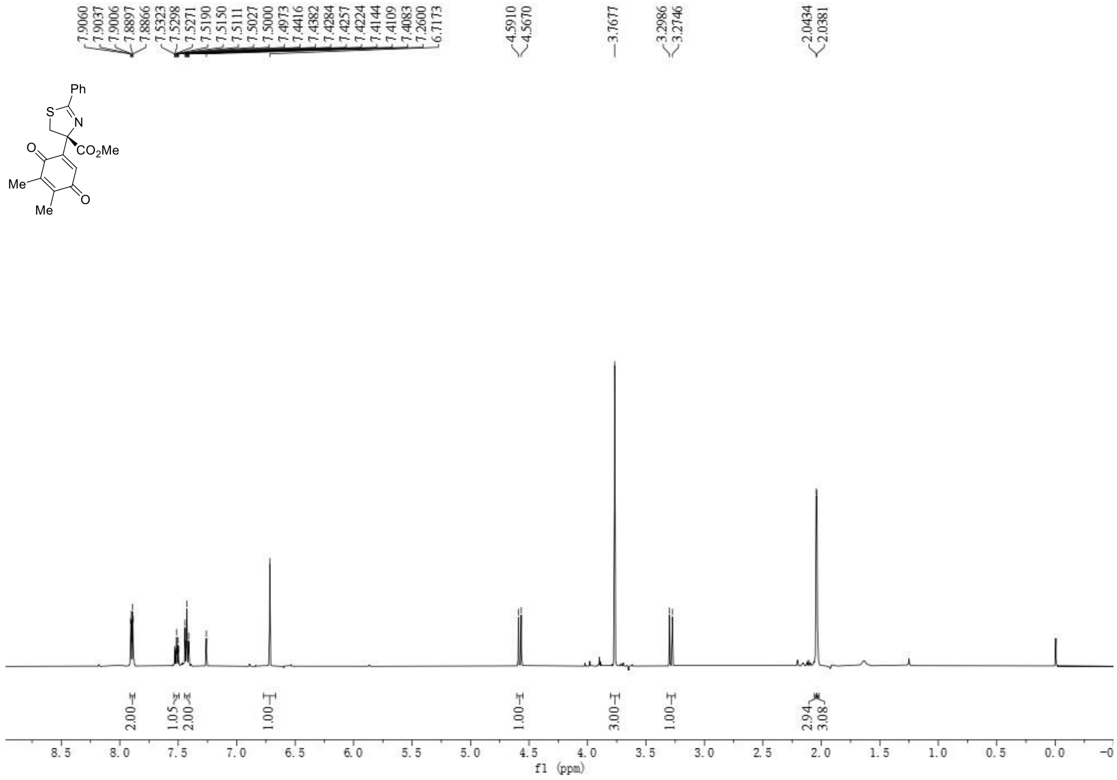

^{13}C NMR of 3l

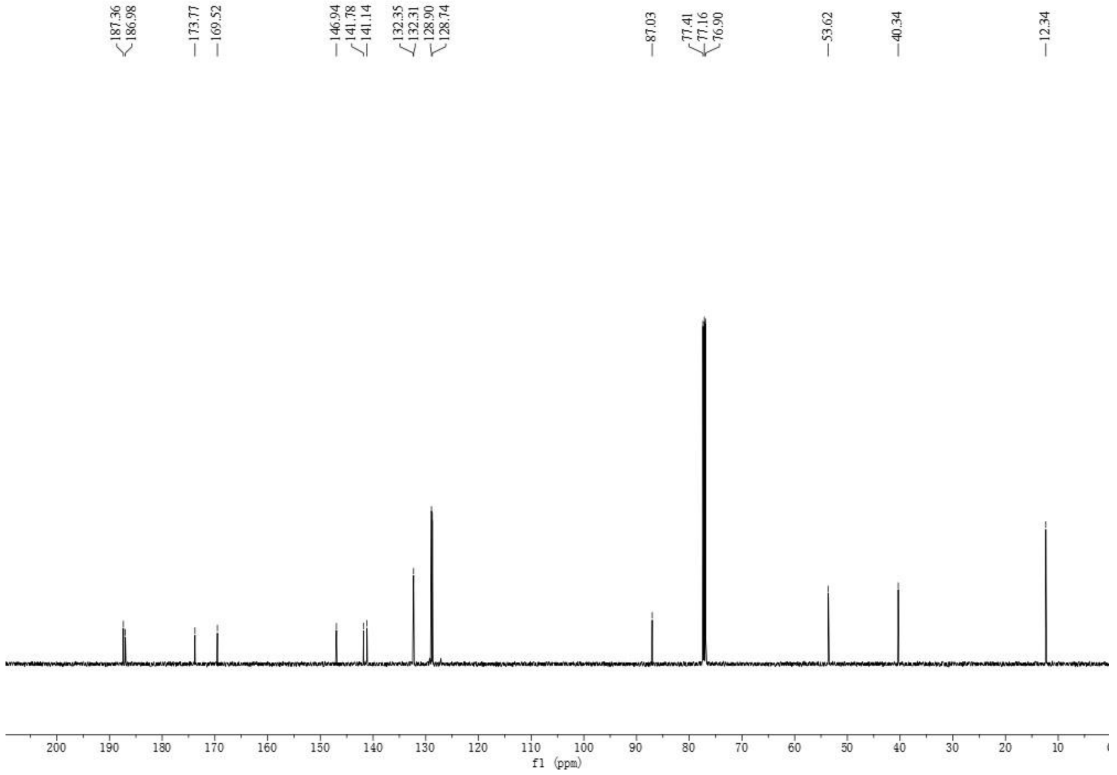

Supplementary Figure 18. ^{1}H NMR, and ^{13}C NMR spectra of compound **3l**

^{1}H NMR of 3m

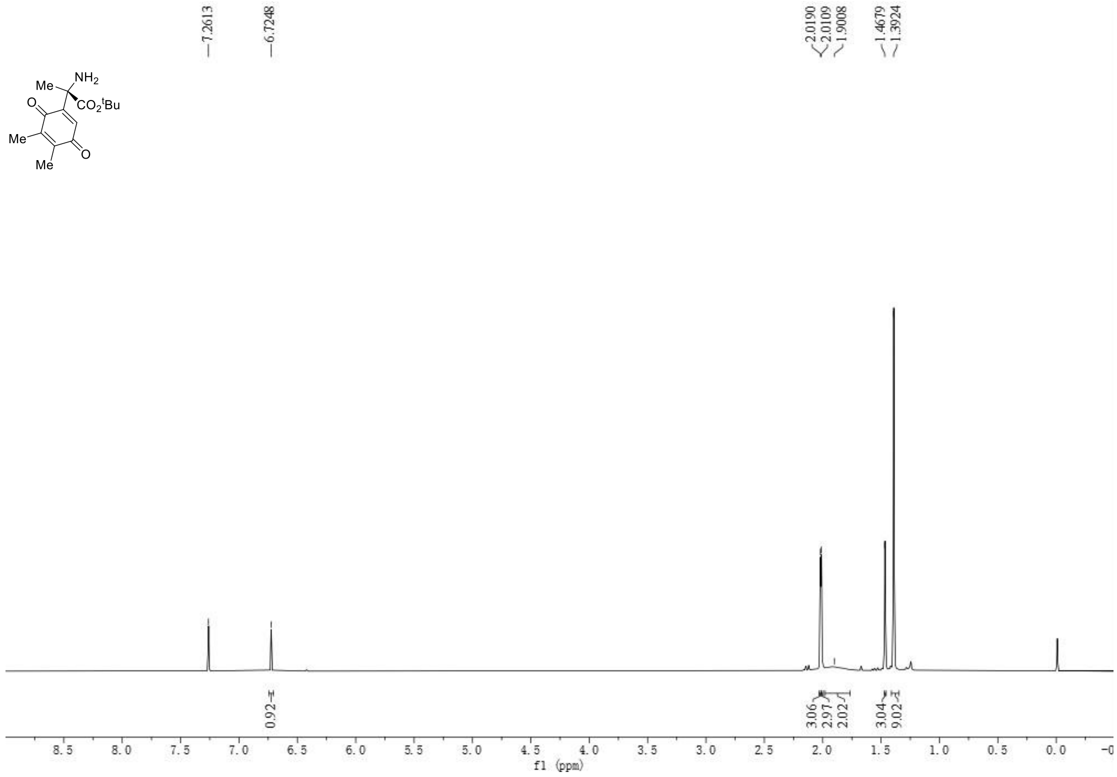

^{13}C NMR of 3m

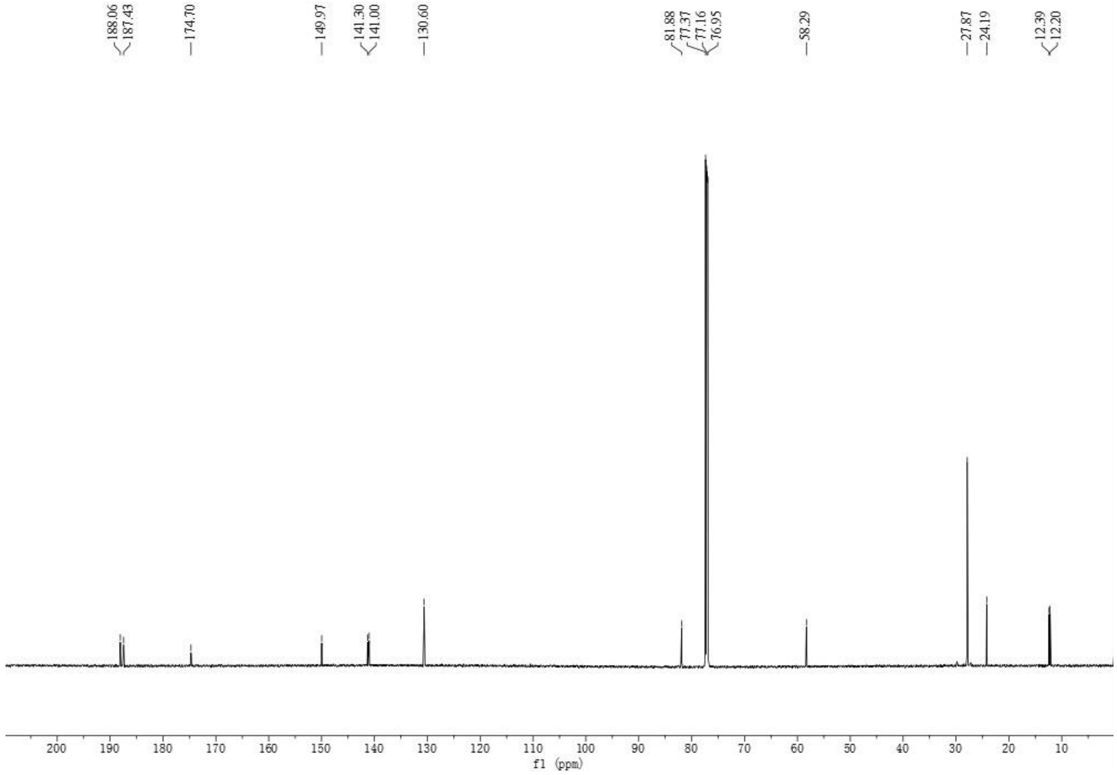

Supplementary Figure 19. ^{1}H NMR, and ^{13}C NMR spectra of compound **3m**

^{1}H NMR of 3n

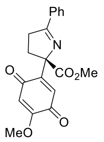

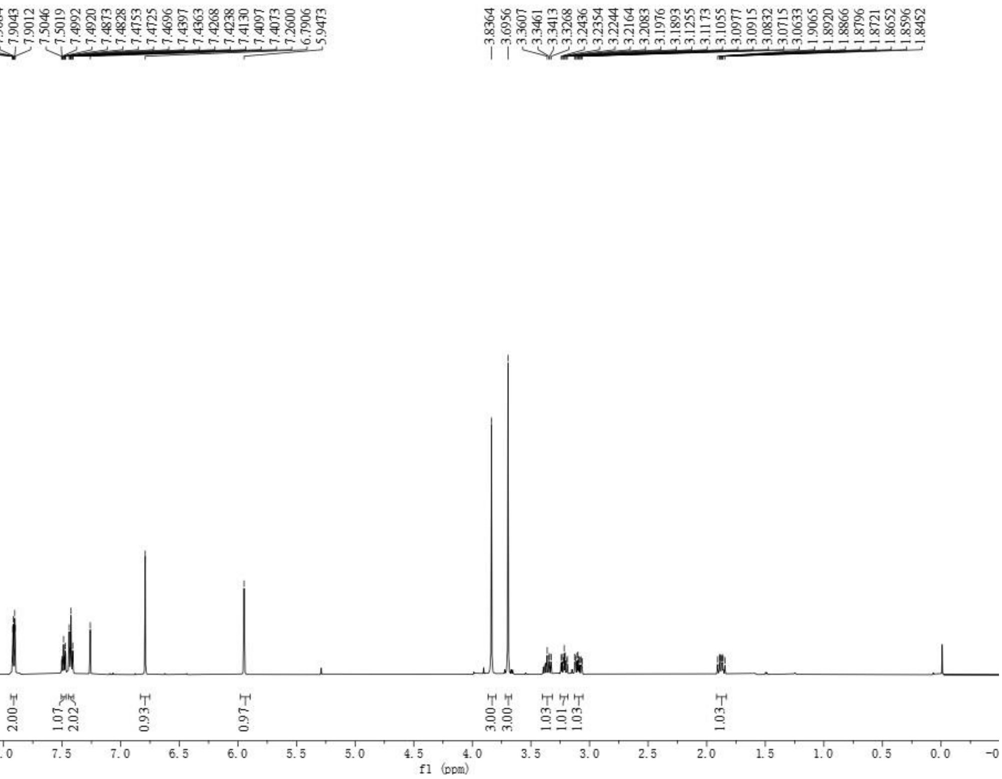

^{13}C NMR of 3n

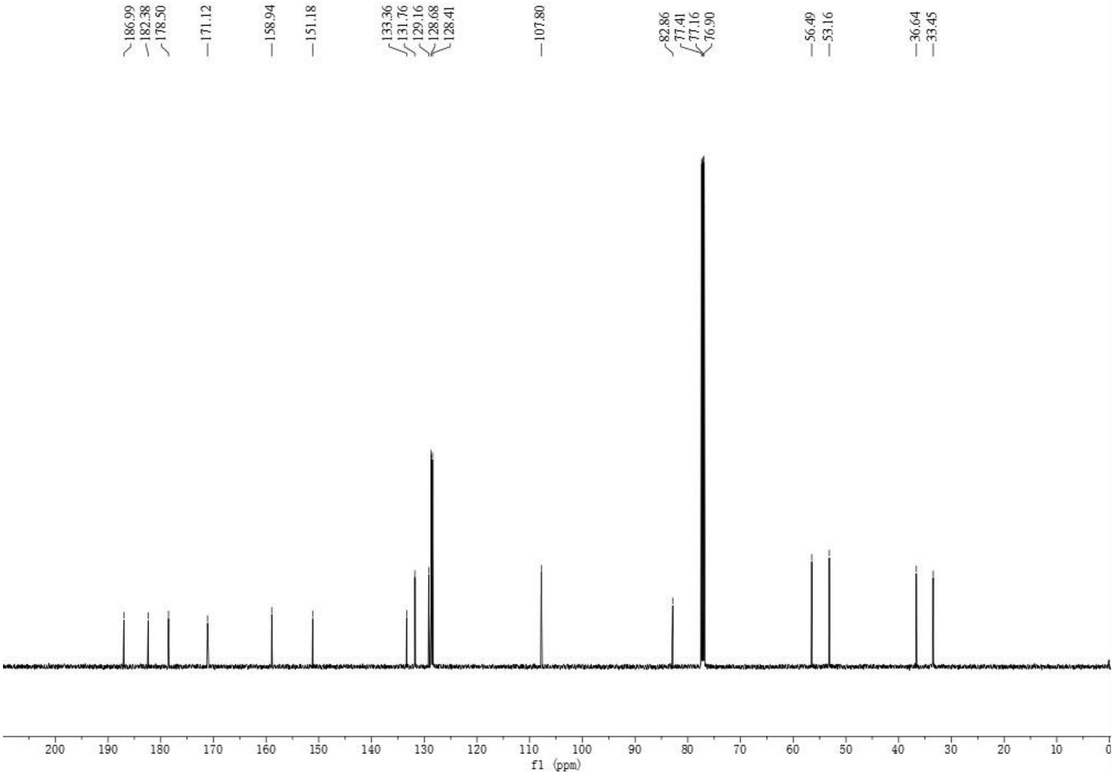

Supplementary Figure 20. ^{1}H NMR, and ^{13}C NMR spectra of compound **3n**

^{1}H NMR of 3o

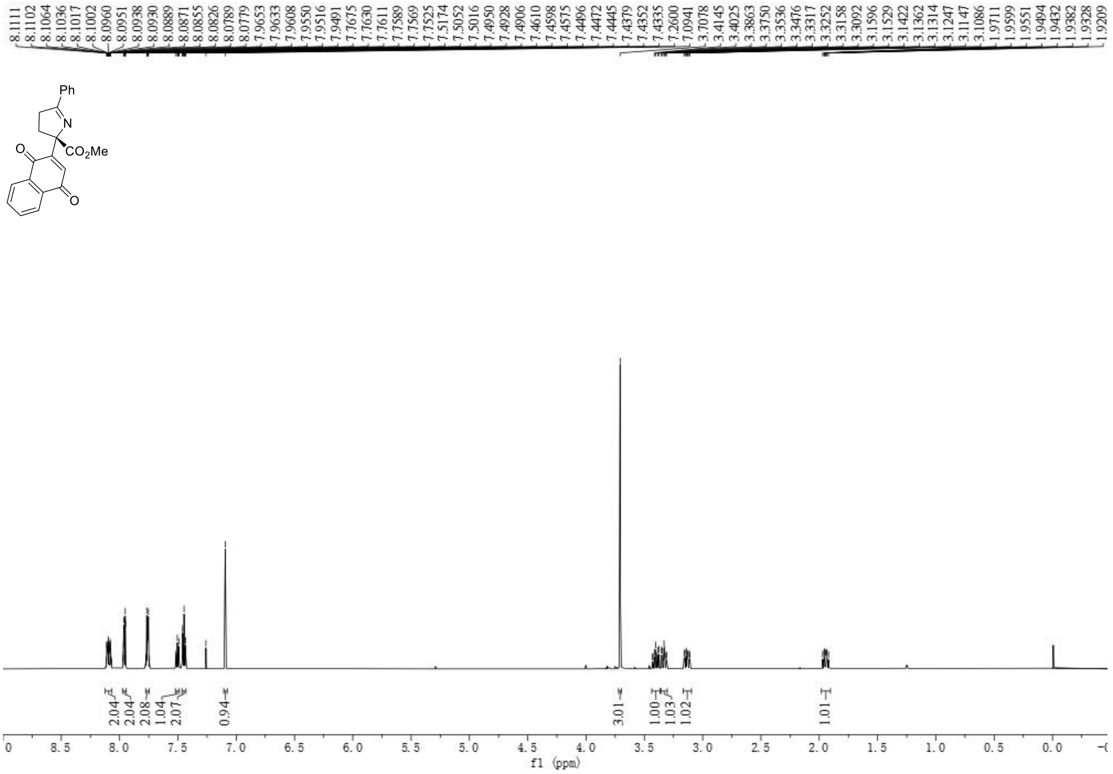

^{13}C NMR of 3o

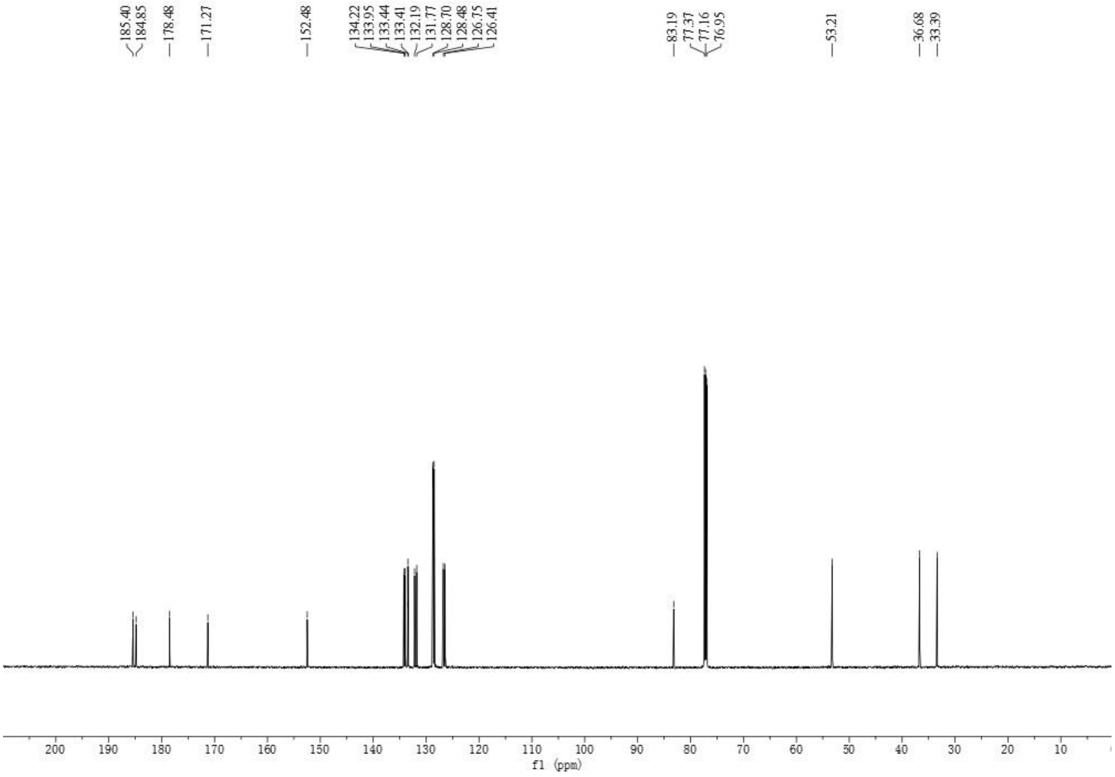

Supplementary Figure 21. ^{1}H NMR, and ^{13}C NMR spectra of compound **3o**

^{1}H NMR of 3p

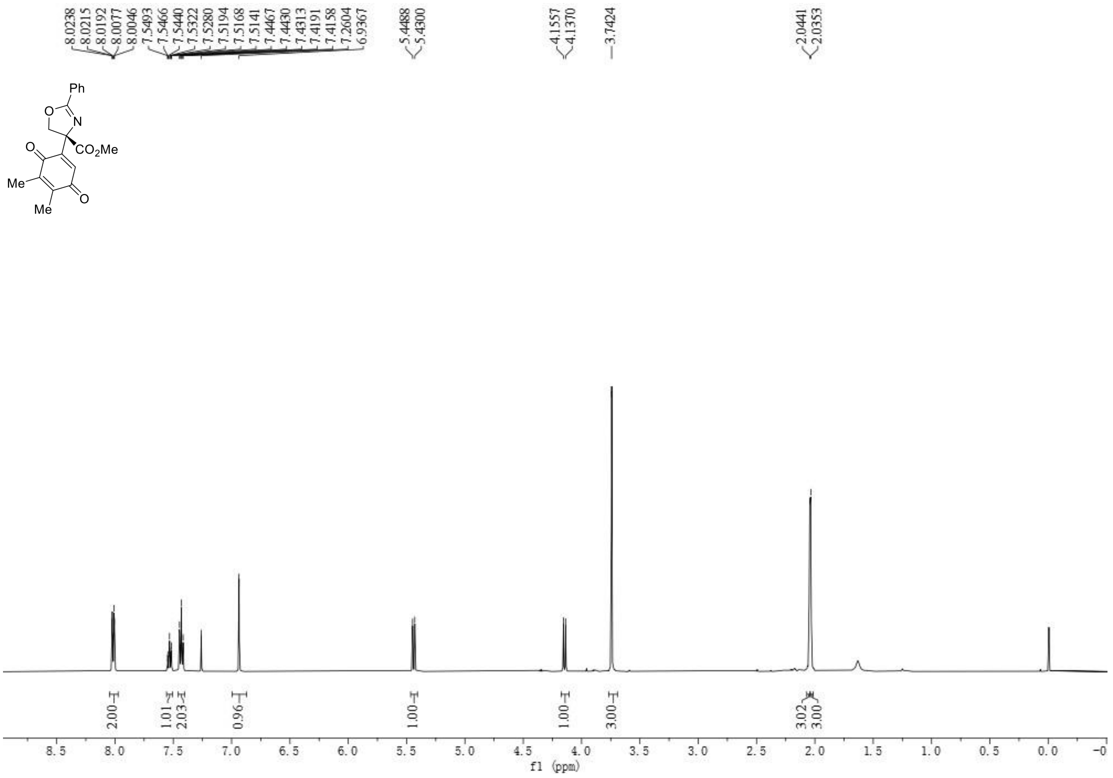

^{13}C NMR of 3p

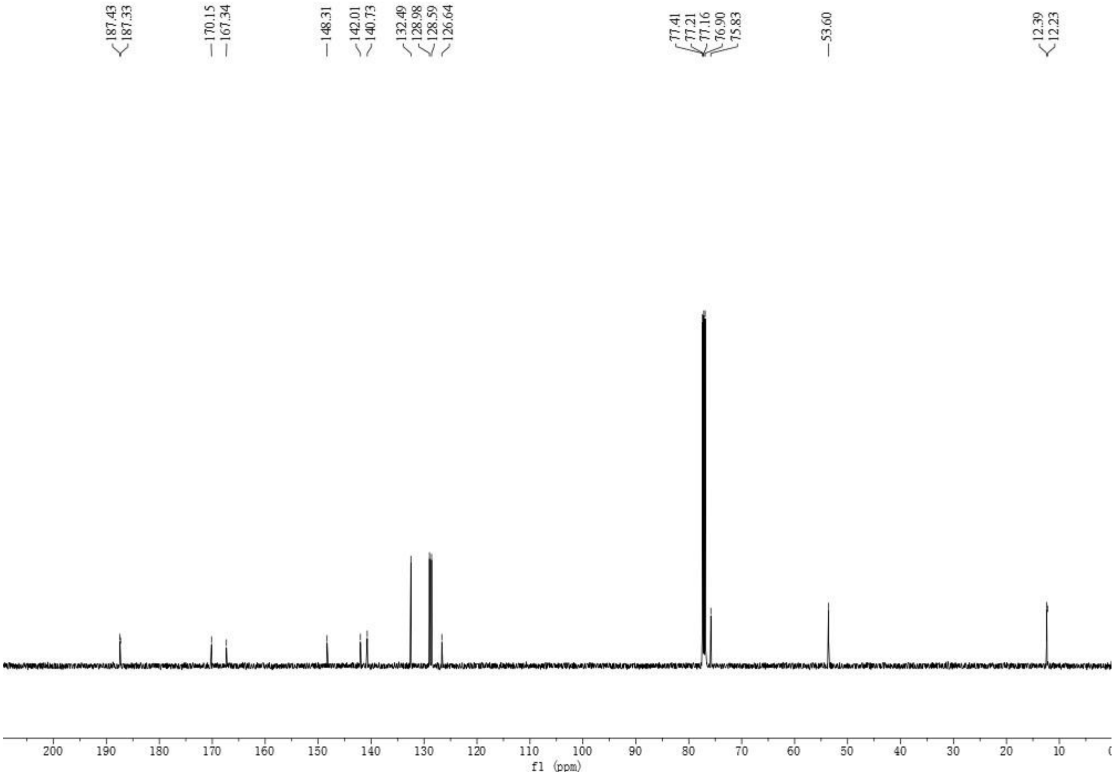

Supplementary Figure 22. ^{1}H NMR, and ^{13}C NMR spectra of compound **3p**

^{1}H NMR of 4a

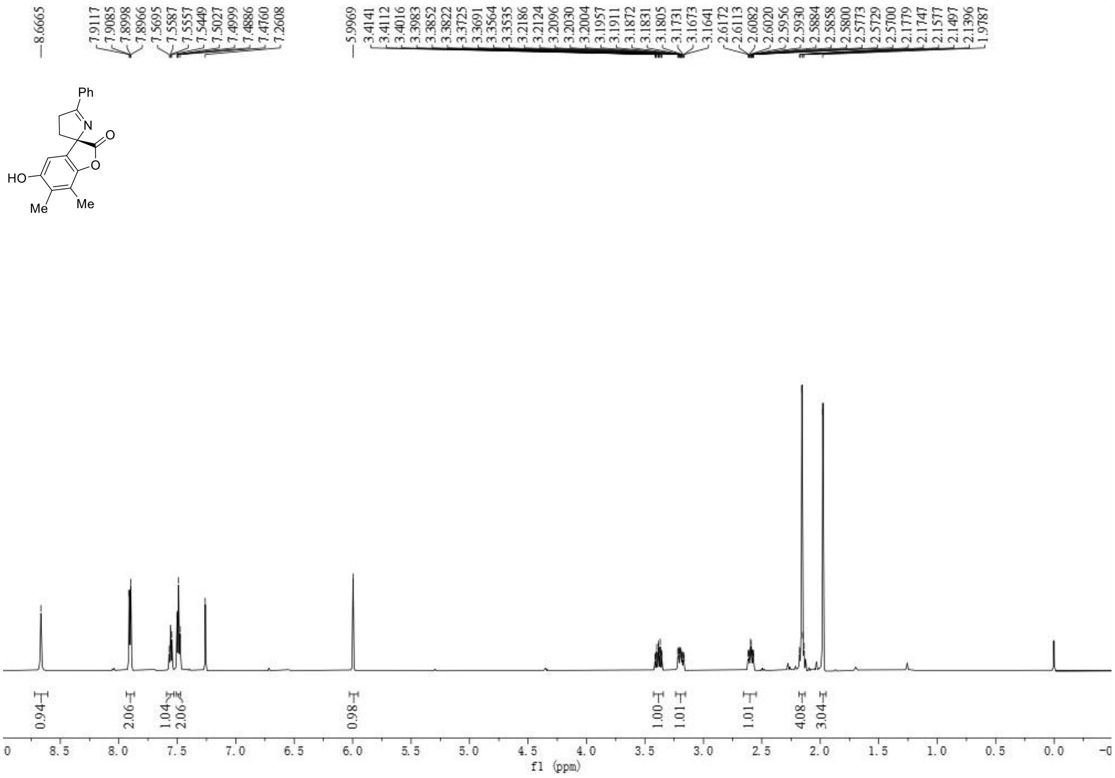

^{13}C NMR of 4a

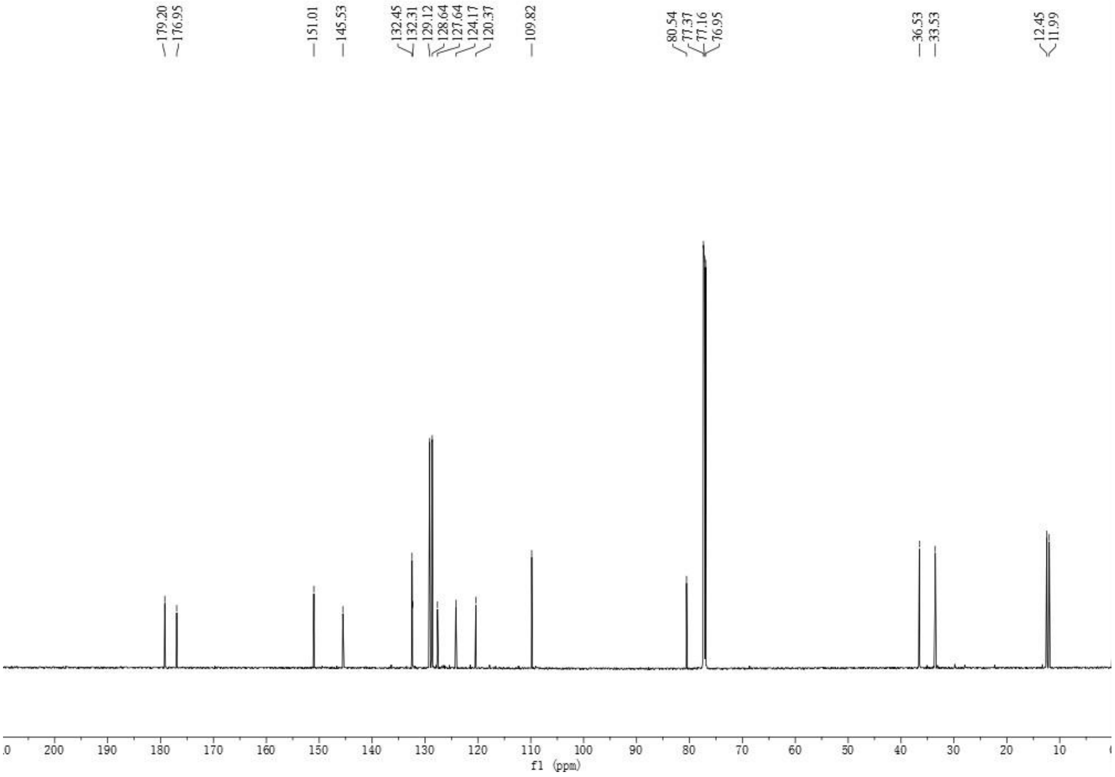

Supplementary Figure 23. ^{1}H NMR, and ^{13}C NMR spectra of compound **4a**

^{1}H NMR of 4b

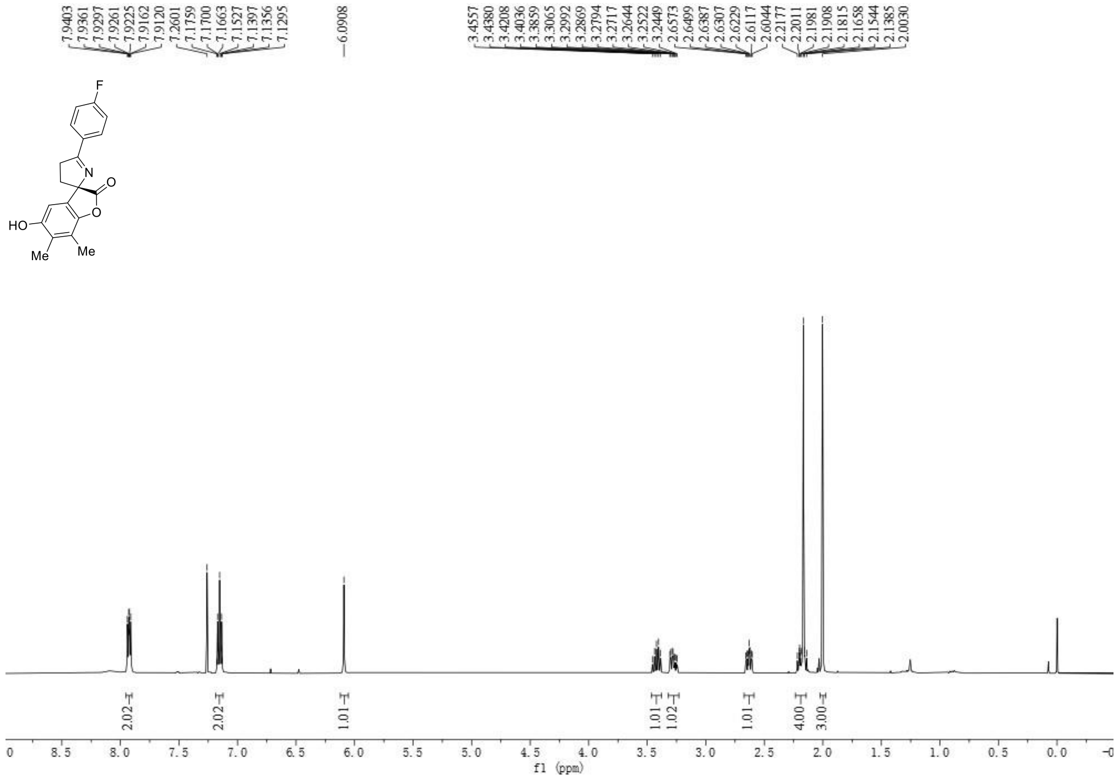

^{19}F NMR of 4b

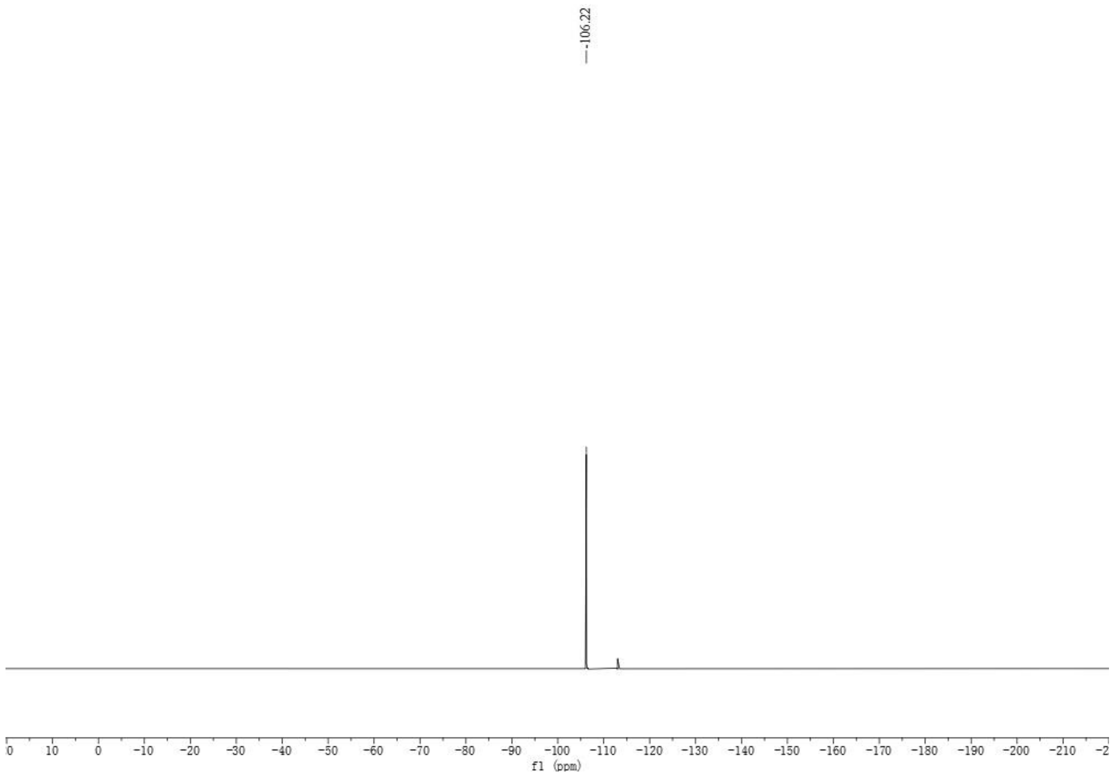

^{13}C NMR of 4b

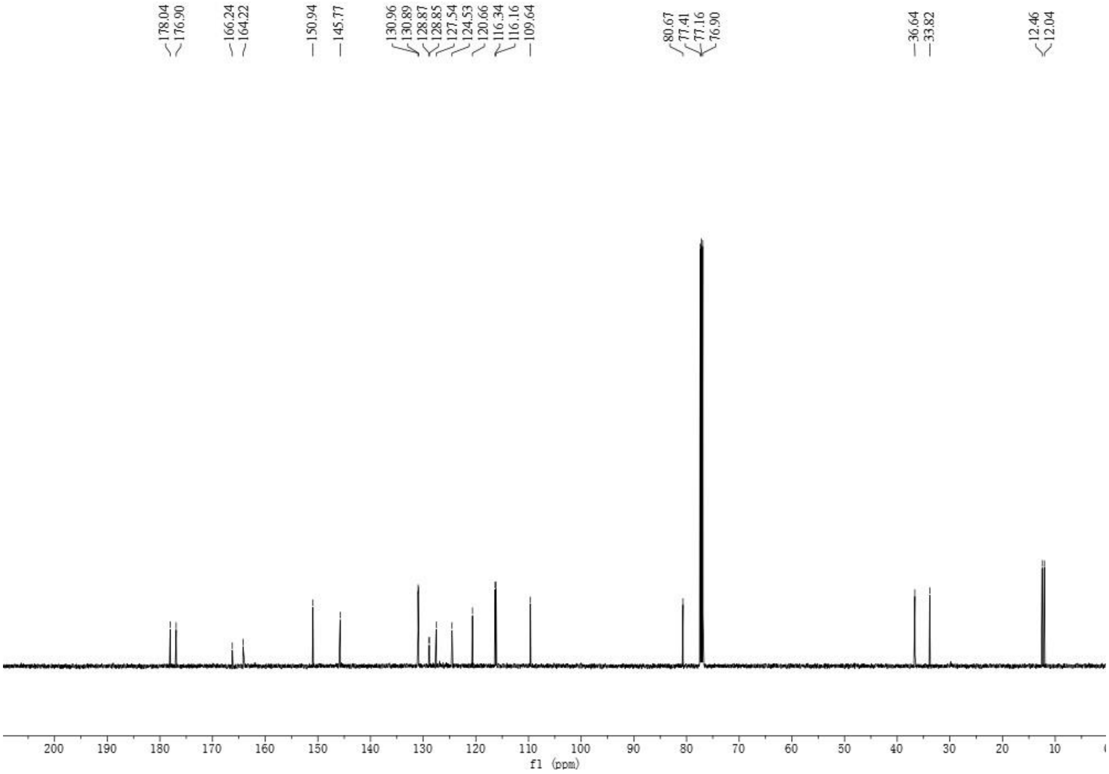

Supplementary Figure 24. ^{1}H NMR, ^{13}C NMR, and ^{19}F NMR spectra of compound **4b**

^{1}H NMR of 4c

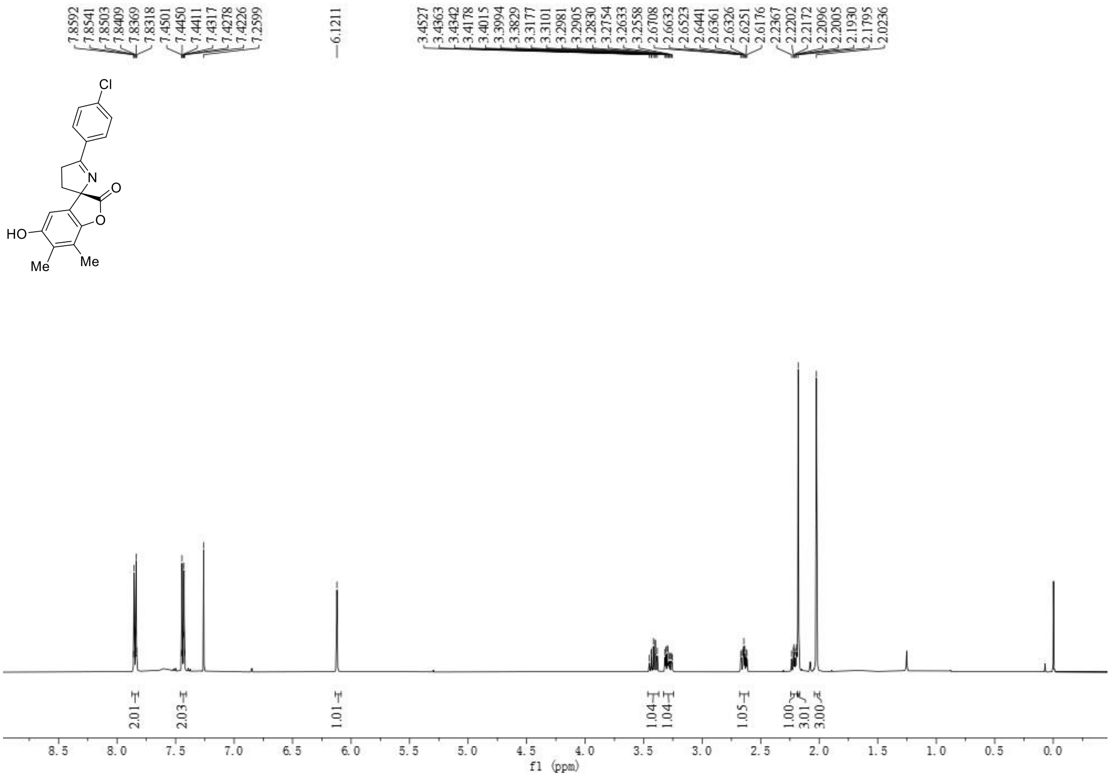

^{13}C NMR of 4c

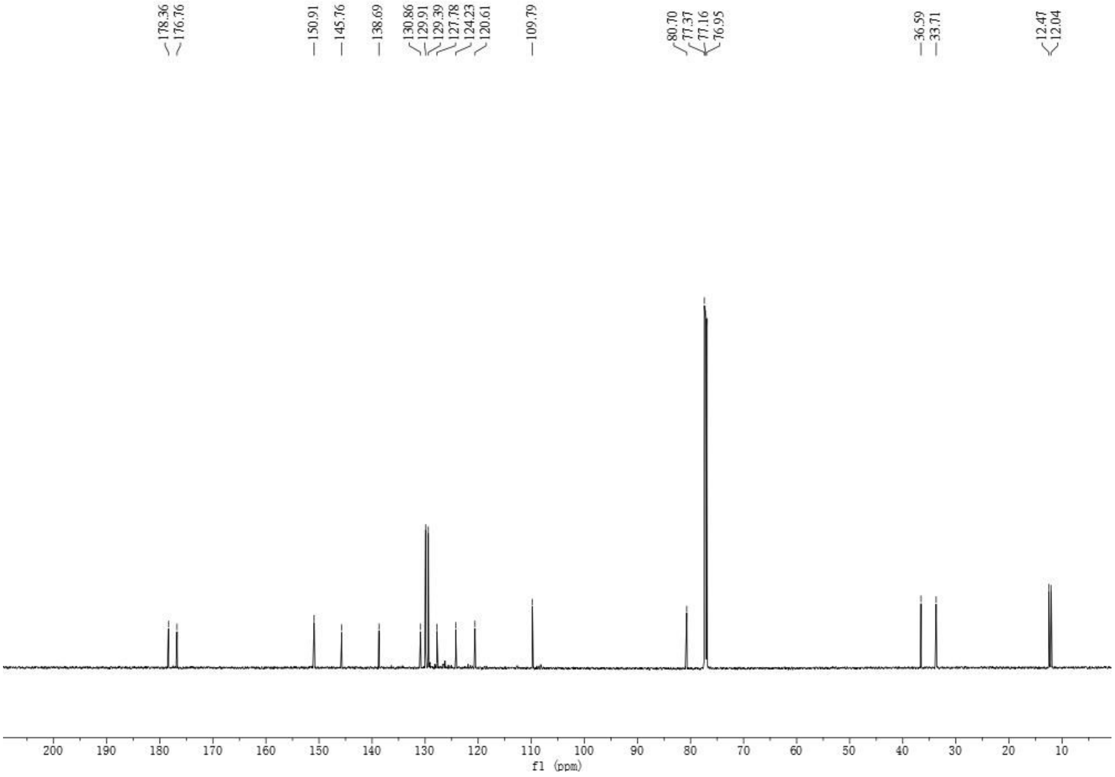

Supplementary Figure 25. ^{1}H NMR, and ^{13}C NMR spectra of compound **4c**

^{1}H NMR of 4d

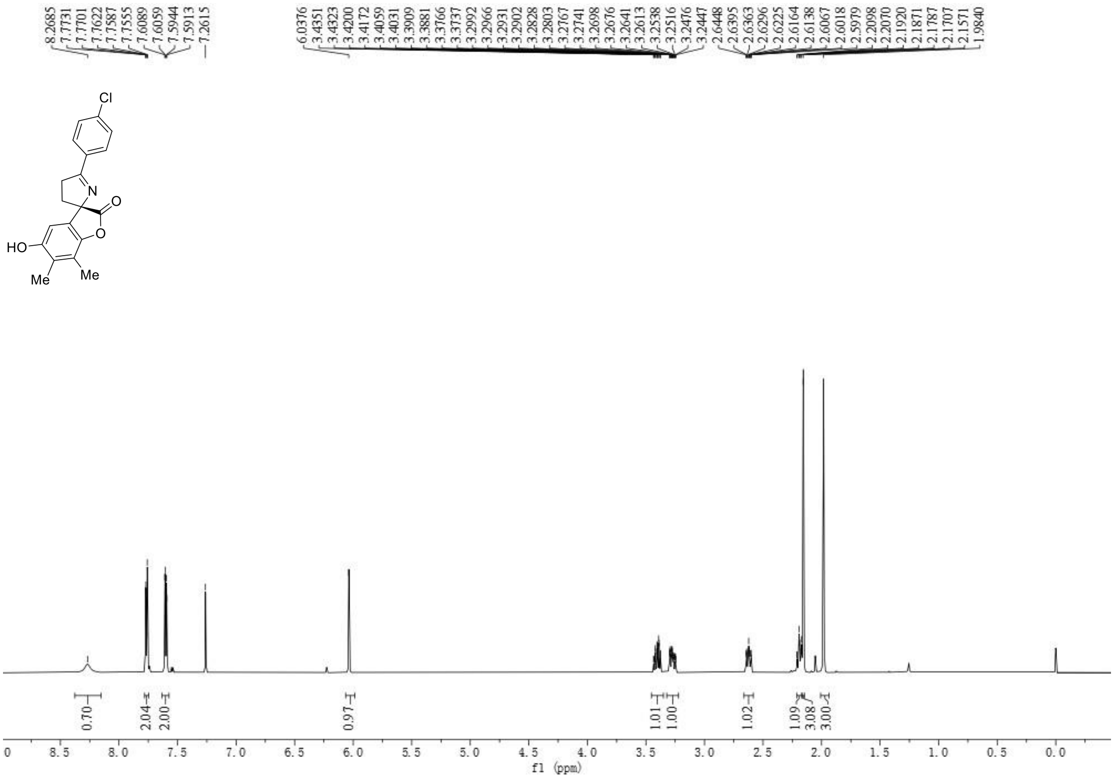

^{13}C NMR of 4d

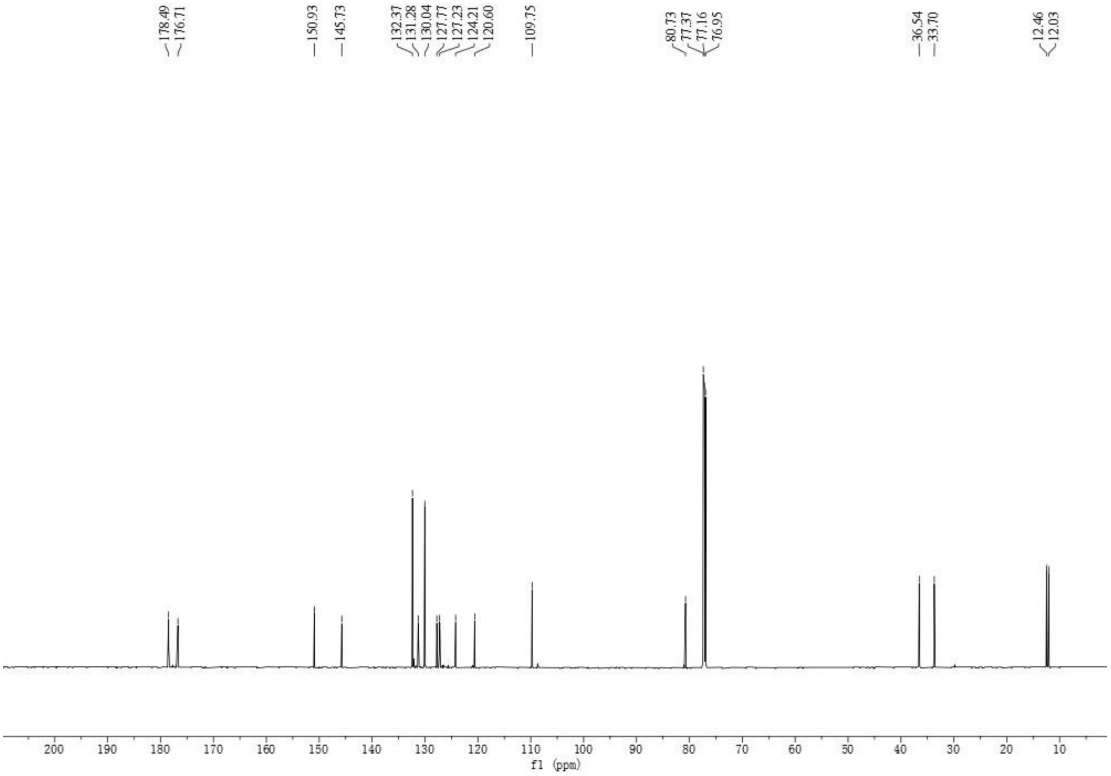

Supplementary Figure 26. ^{1}H NMR, and ^{13}C NMR spectra of compound **4d**

^{1}H NMR of 4e

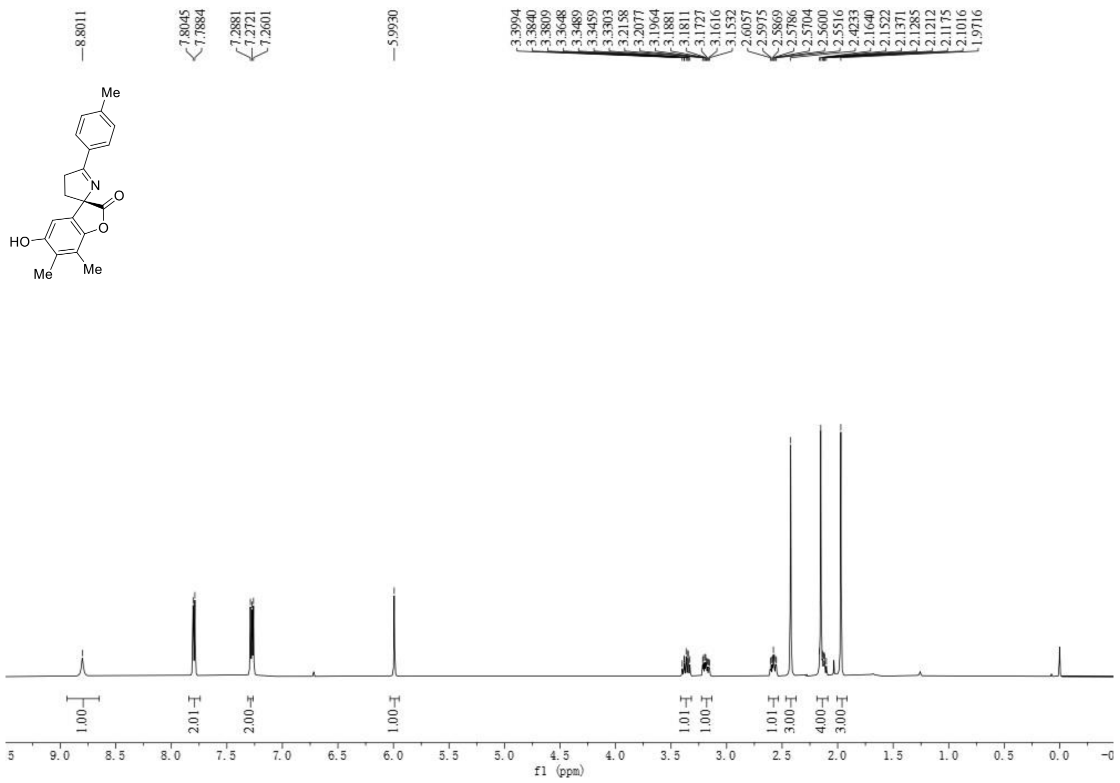

^{13}C NMR of 4e

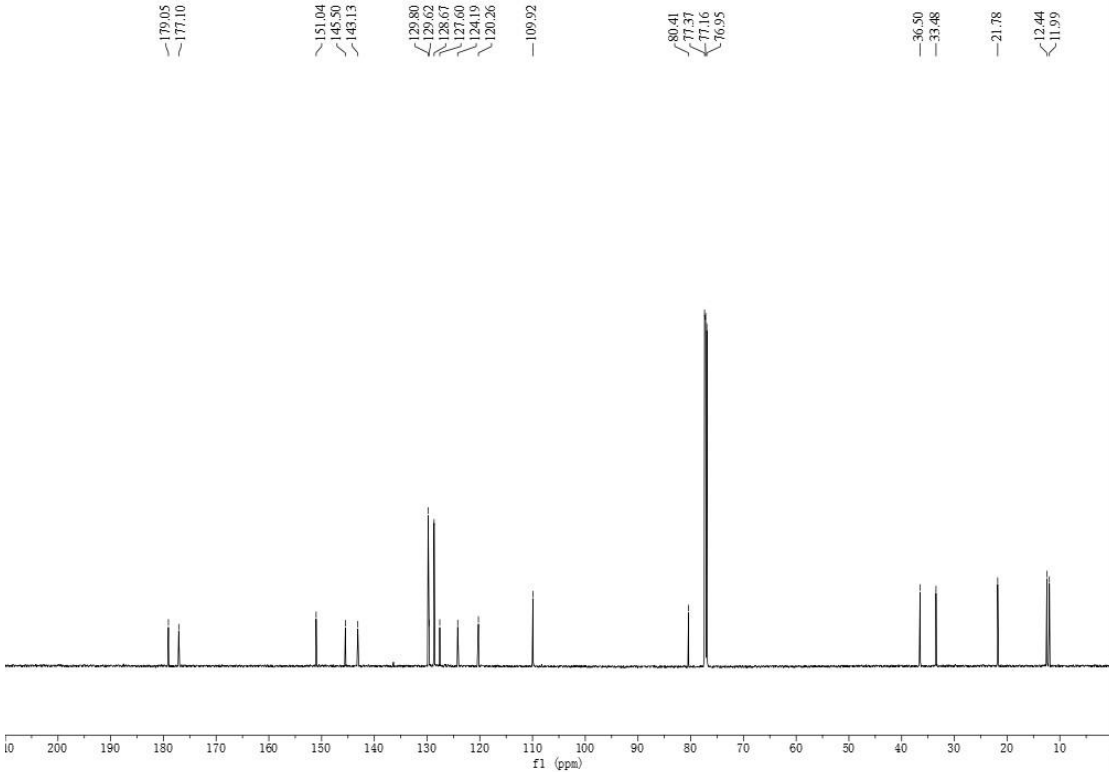

Supplementary Figure 27. ^{1}H NMR, and ^{13}C NMR spectra of compound **4e**

¹H NMR of 4f

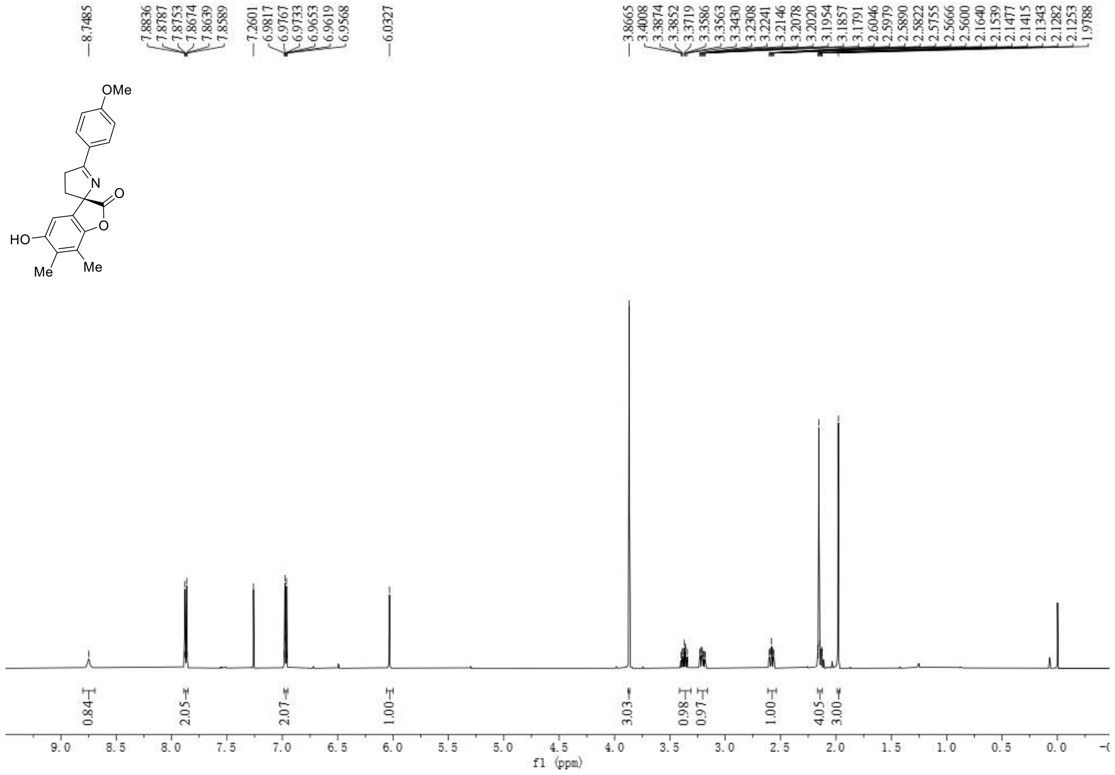

¹³C NMR of 4f

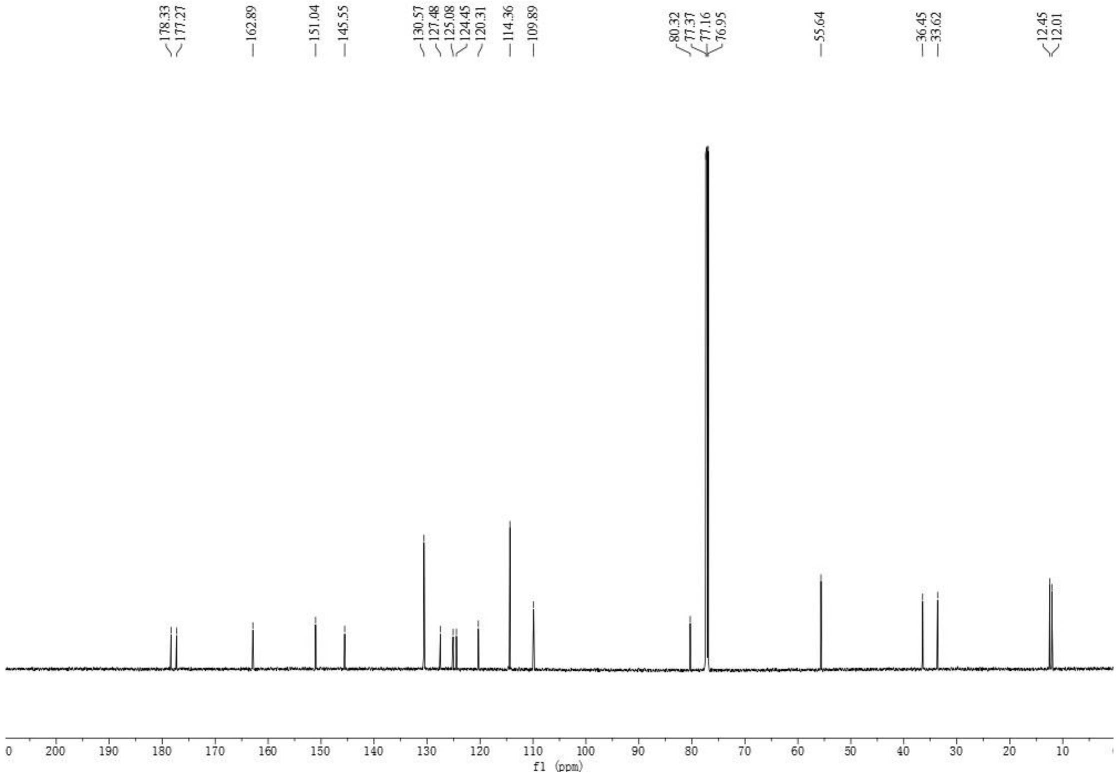

Supplementary Figure 28. ¹H NMR, and ¹³C NMR spectra of compound **4f**

^{1}H NMR of 4g

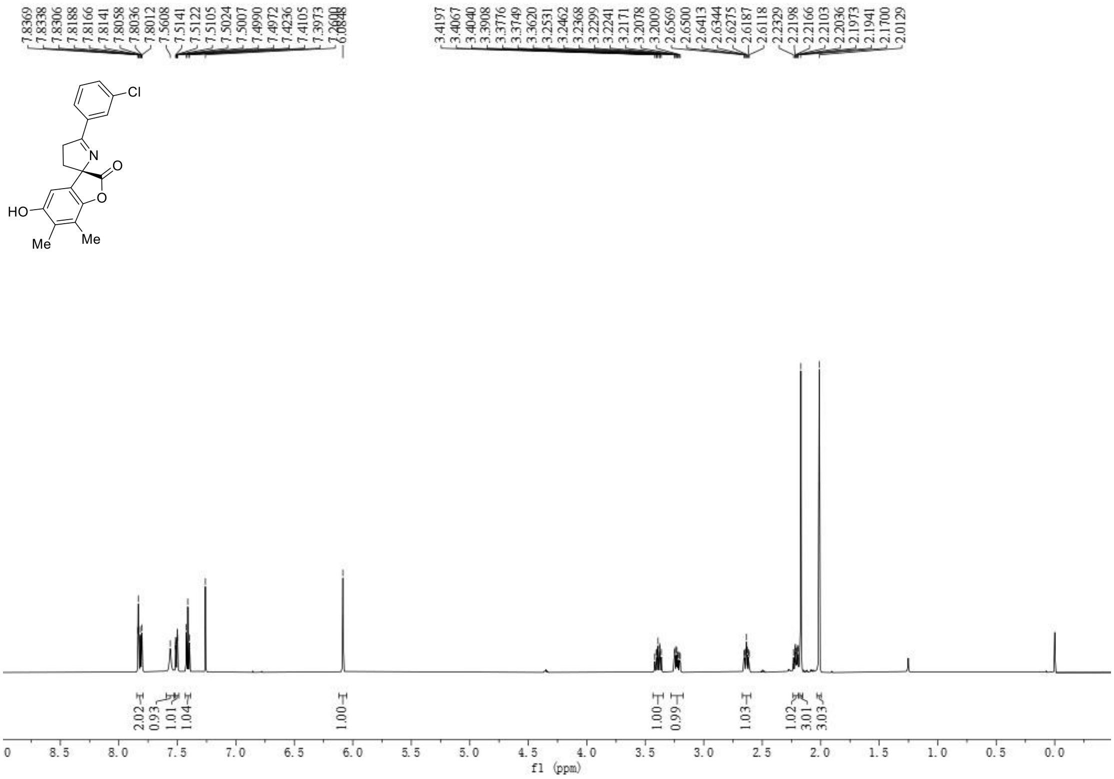

^{13}C NMR of 4g

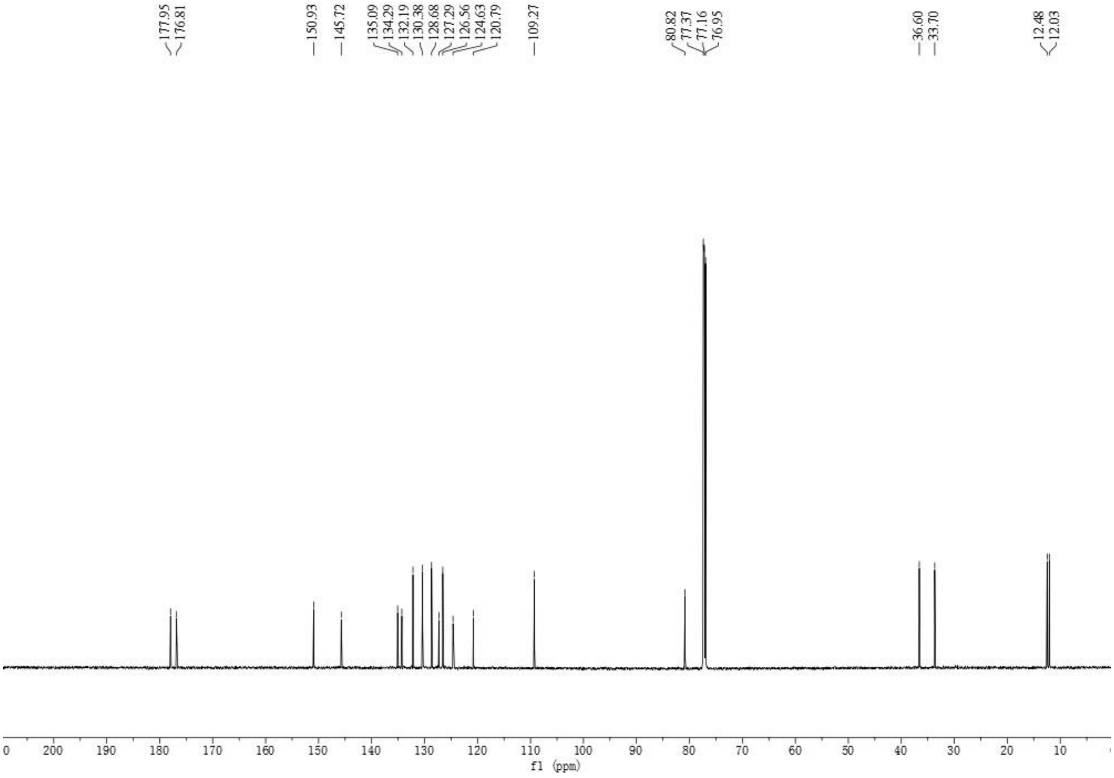

Supplementary Figure 29. ^{1}H NMR, and ^{13}C NMR spectra of compound **4g**

^{1}H NMR of 4h

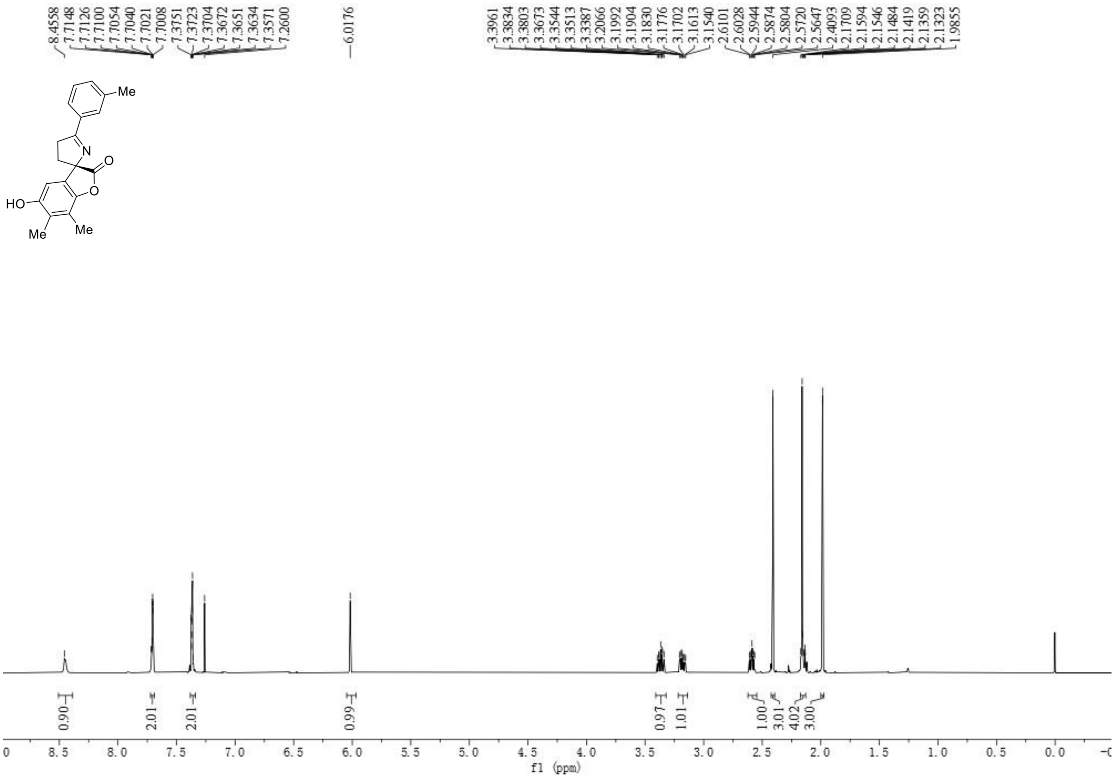

^{13}C NMR of 4h

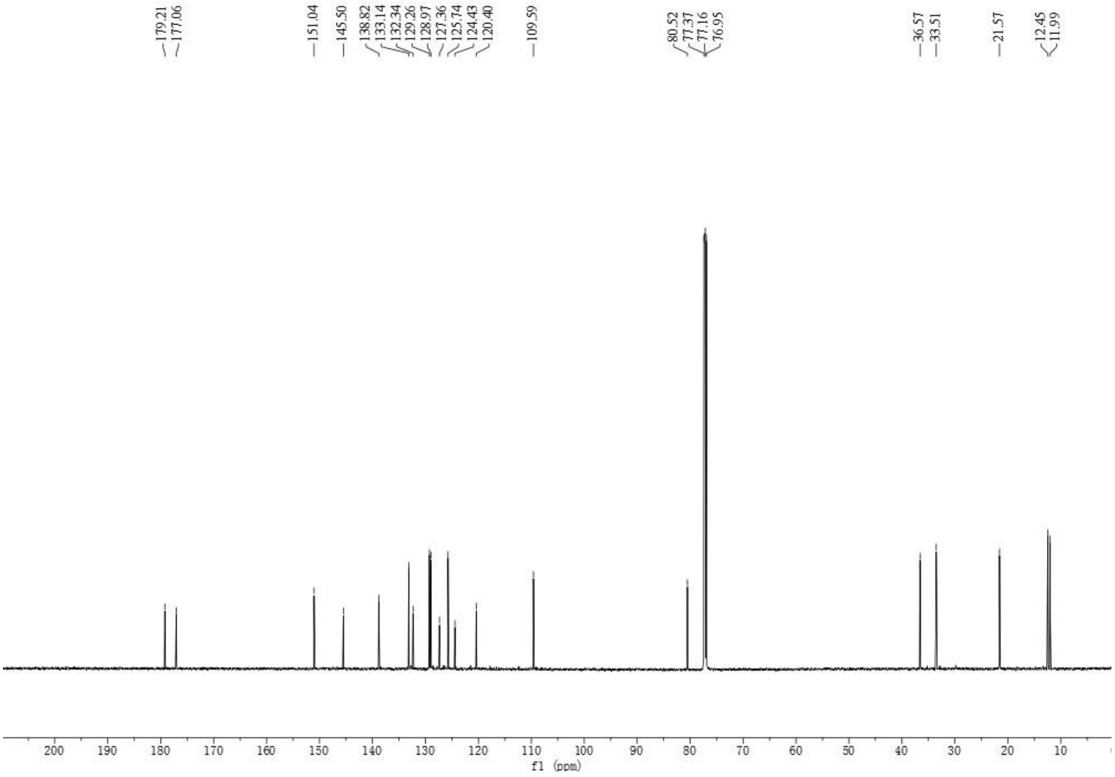

Supplementary Figure 30. ^{1}H NMR, and ^{13}C NMR spectra of compound **4h**

^{1}H NMR of 4i

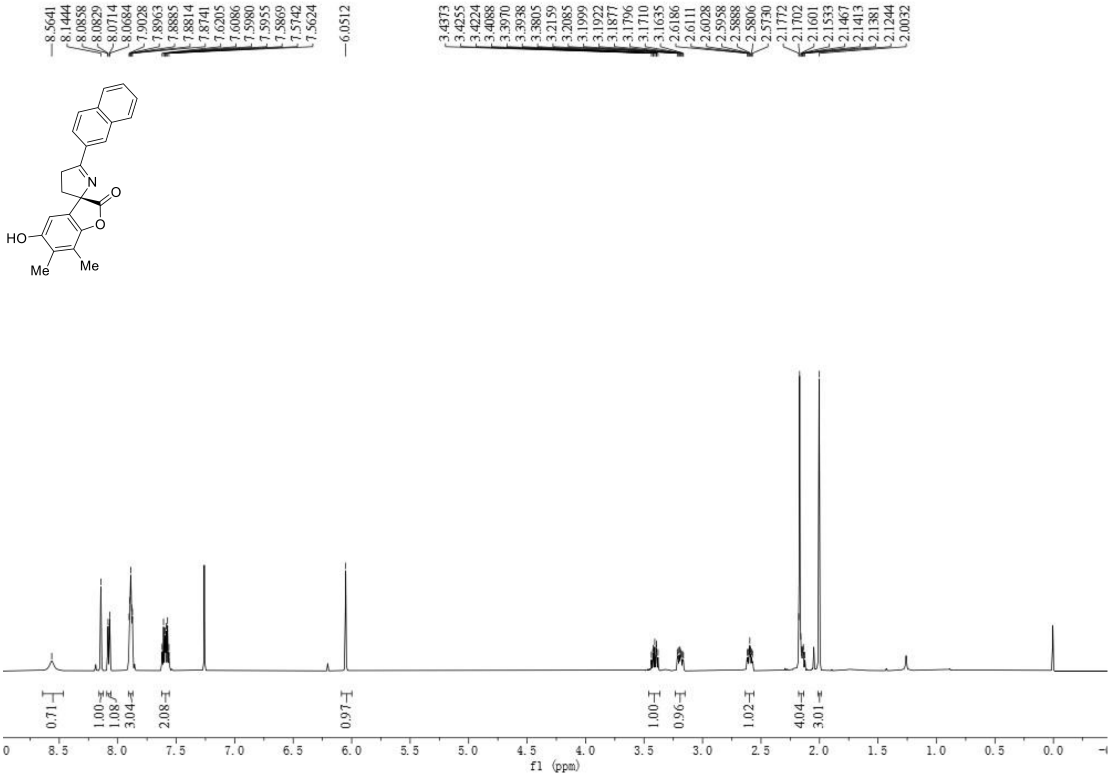

^{13}C NMR of 4i

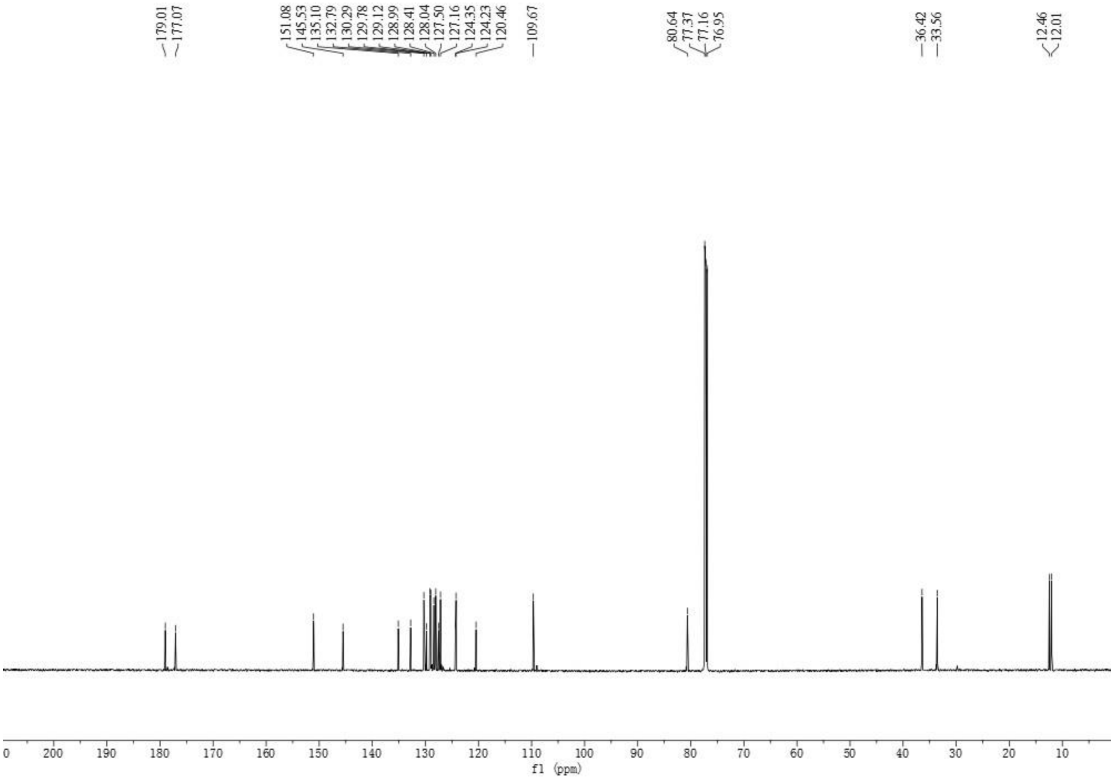

Supplementary Figure 31. ^{1}H NMR, and ^{13}C NMR spectra of compound **4i**

^{1}H NMR of 4j

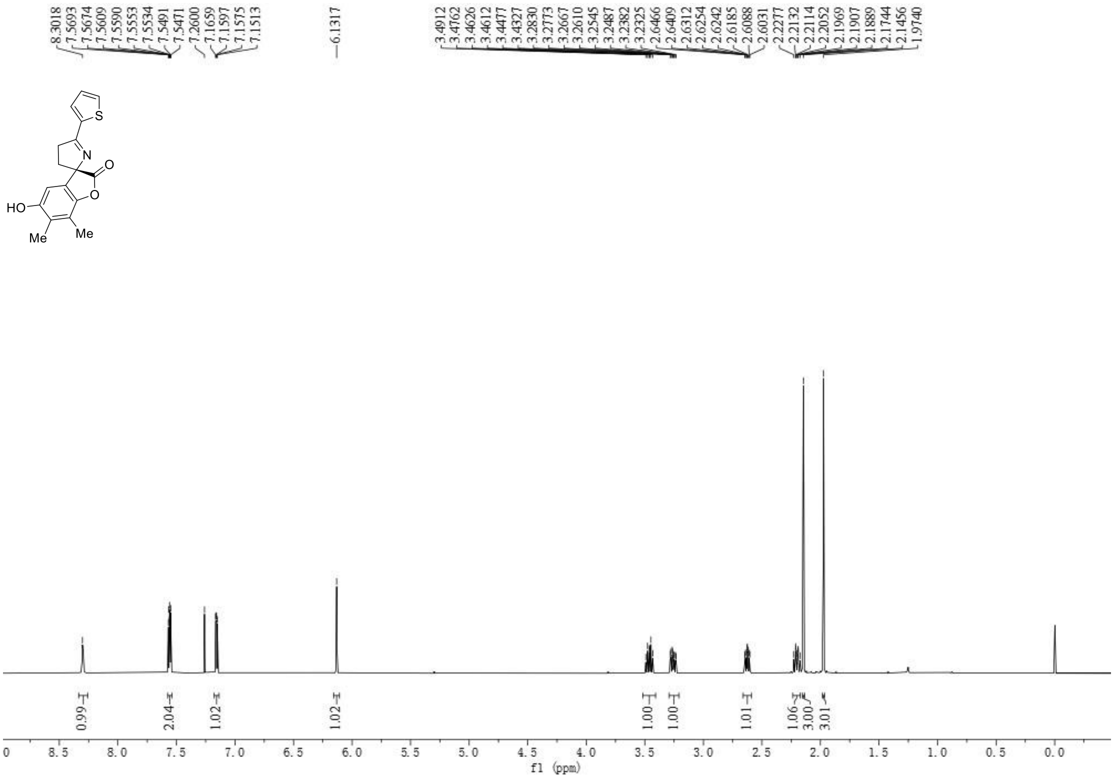

^{13}C NMR of 4j

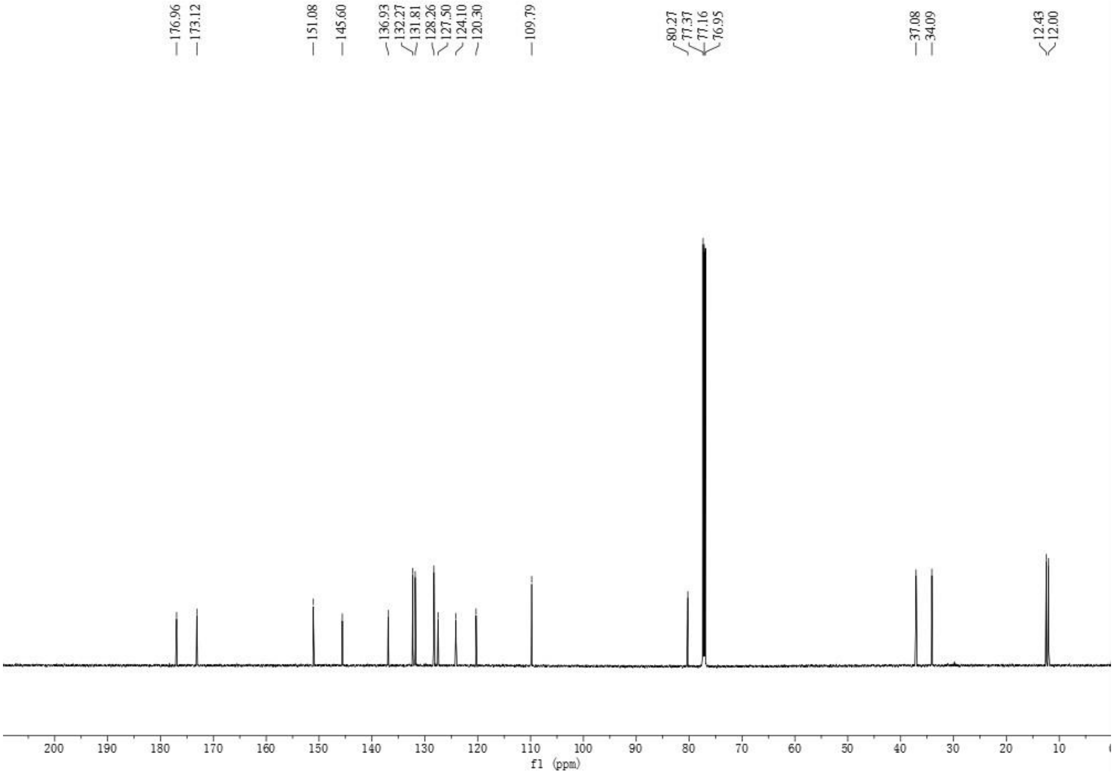

Supplementary Figure 32. ^{1}H NMR, and ^{13}C NMR spectra of compound **4j**

^{1}H NMR of 4k

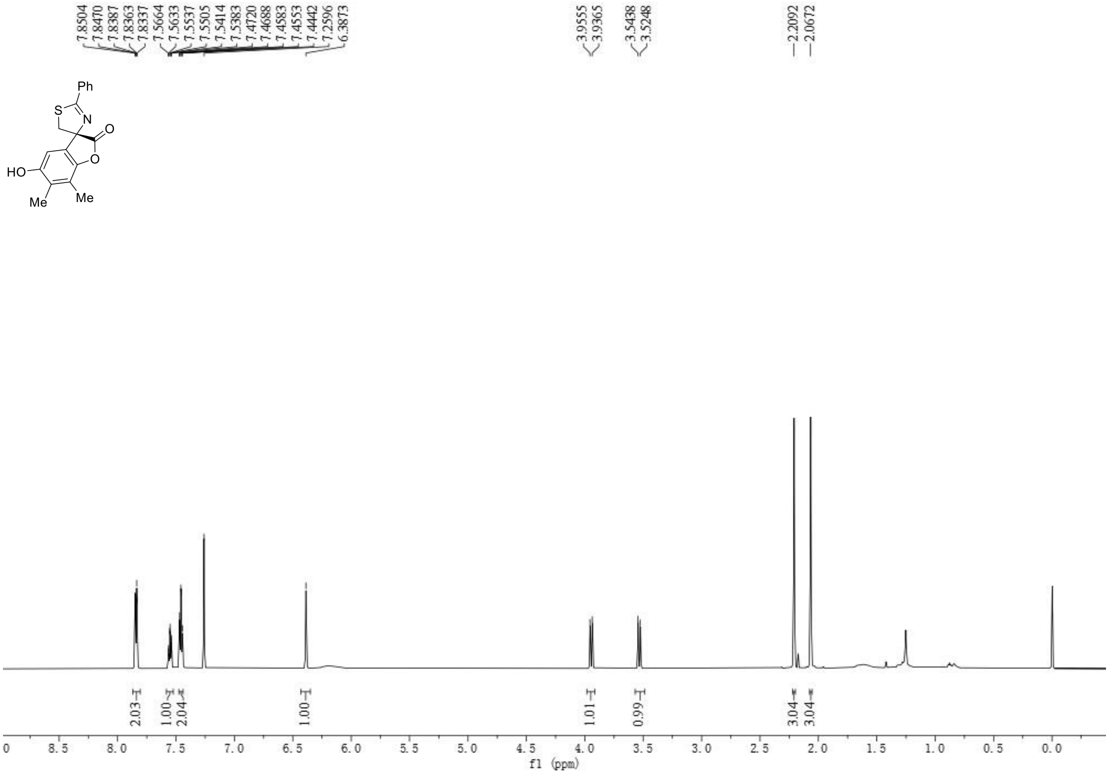

^{13}C NMR of 4k

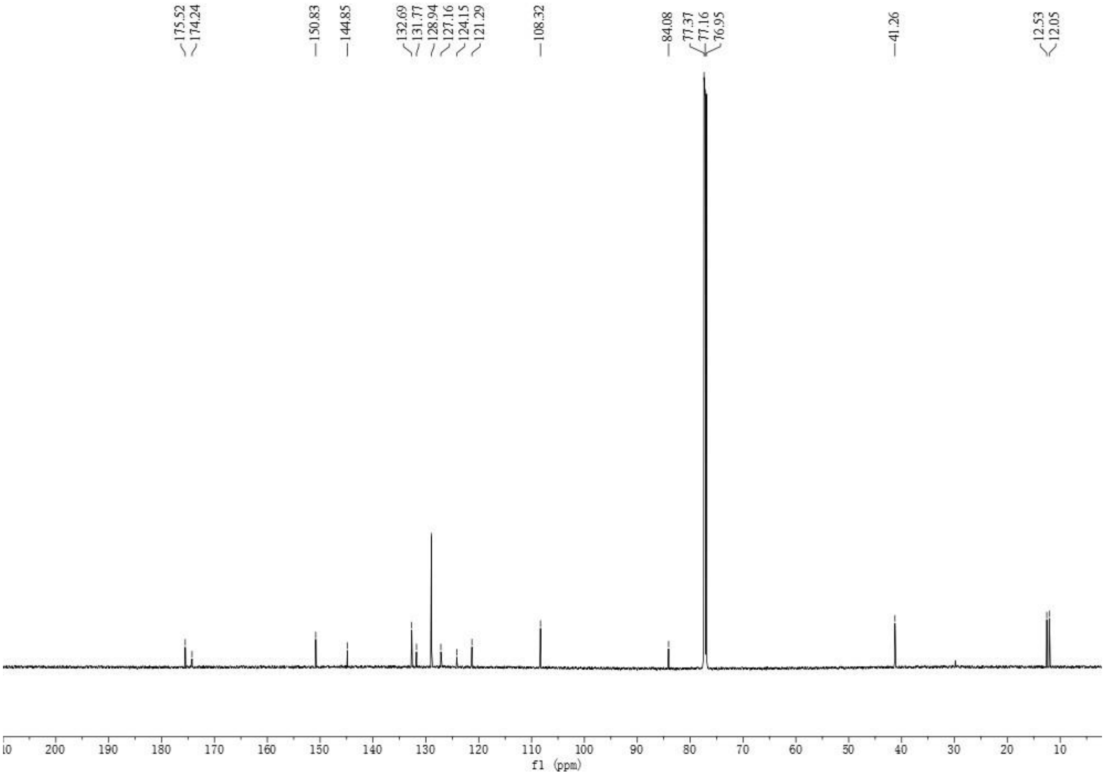

Supplementary Figure 33. ^{1}H NMR, and ^{13}C NMR spectra of compound **4k**

^{1}H NMR of 4l

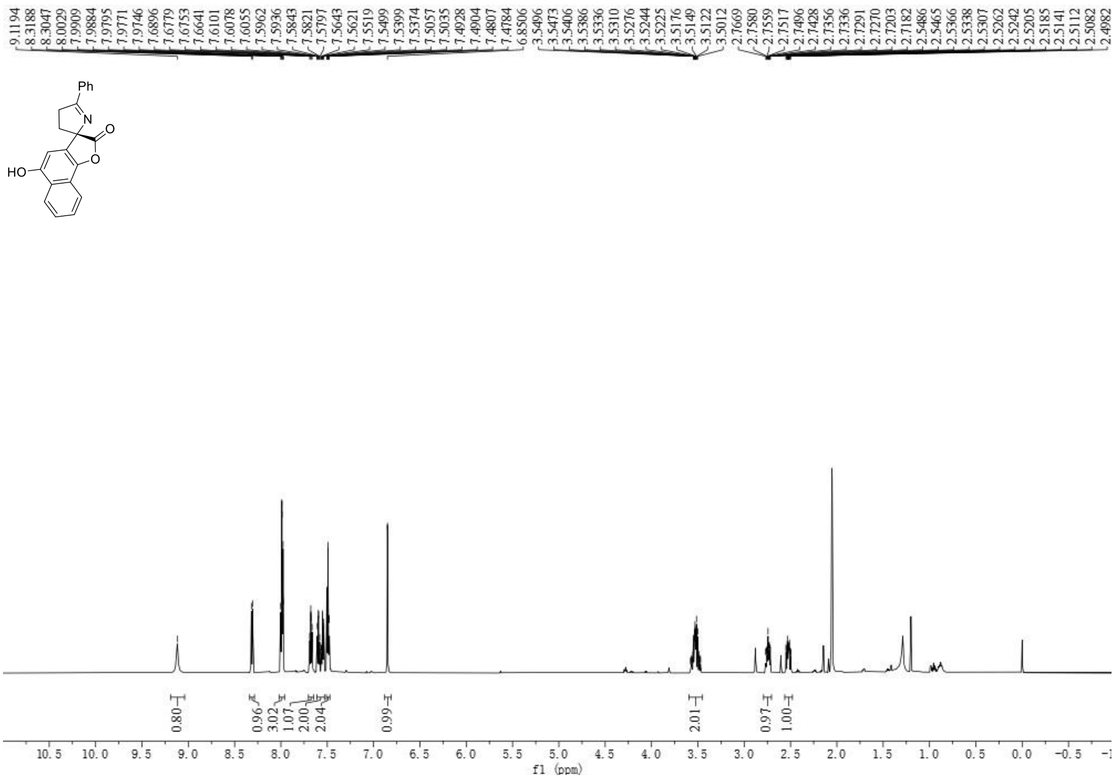

^{13}C NMR of 4l

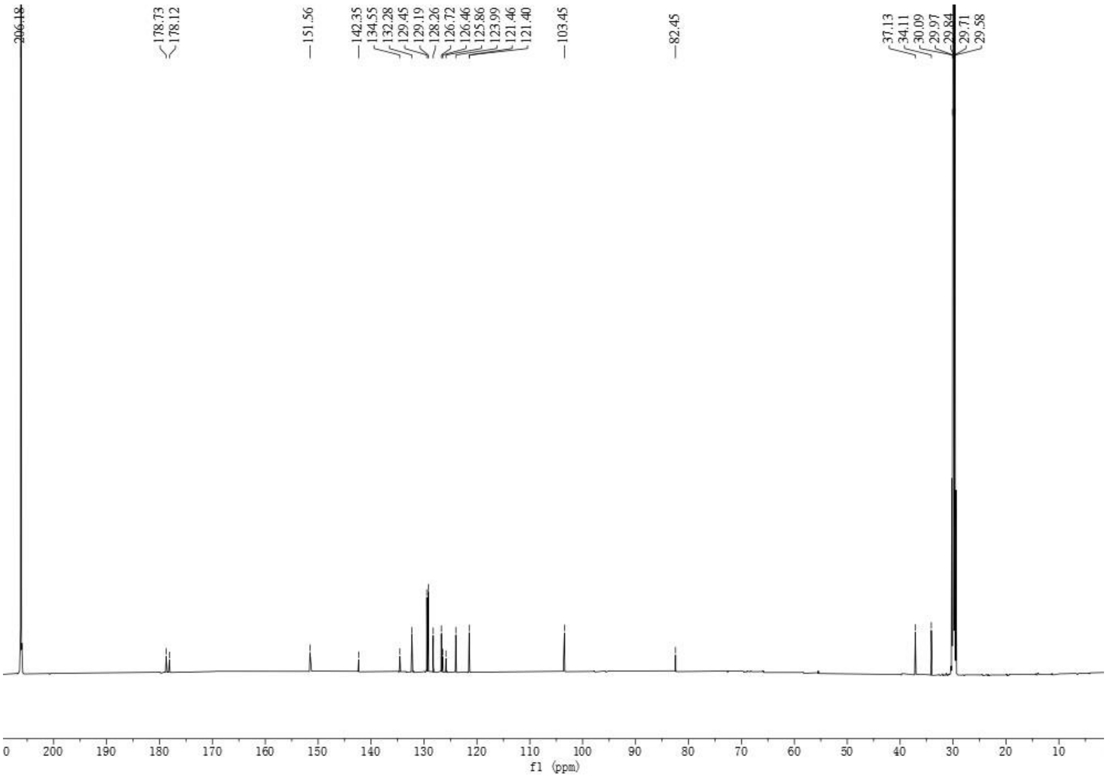

Supplementary Figure 34. ^{1}H NMR, and ^{13}C NMR spectra of compound **4l**

^{1}H NMR of 4m

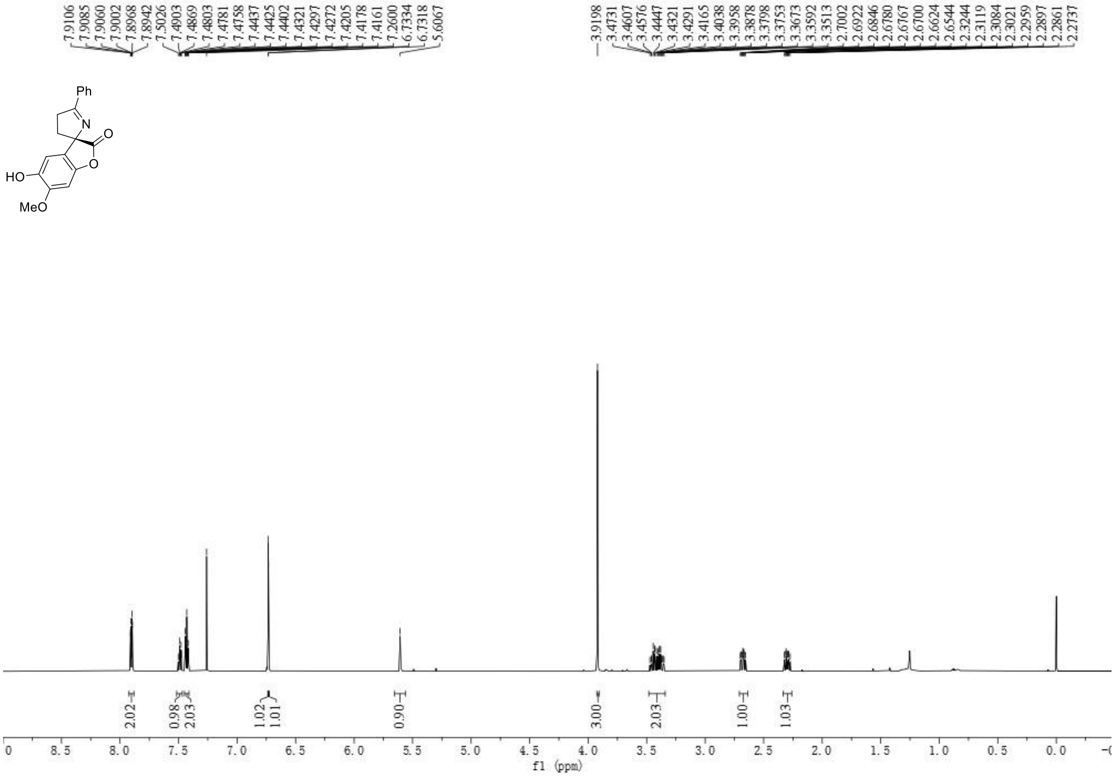

^{13}C NMR of 4m

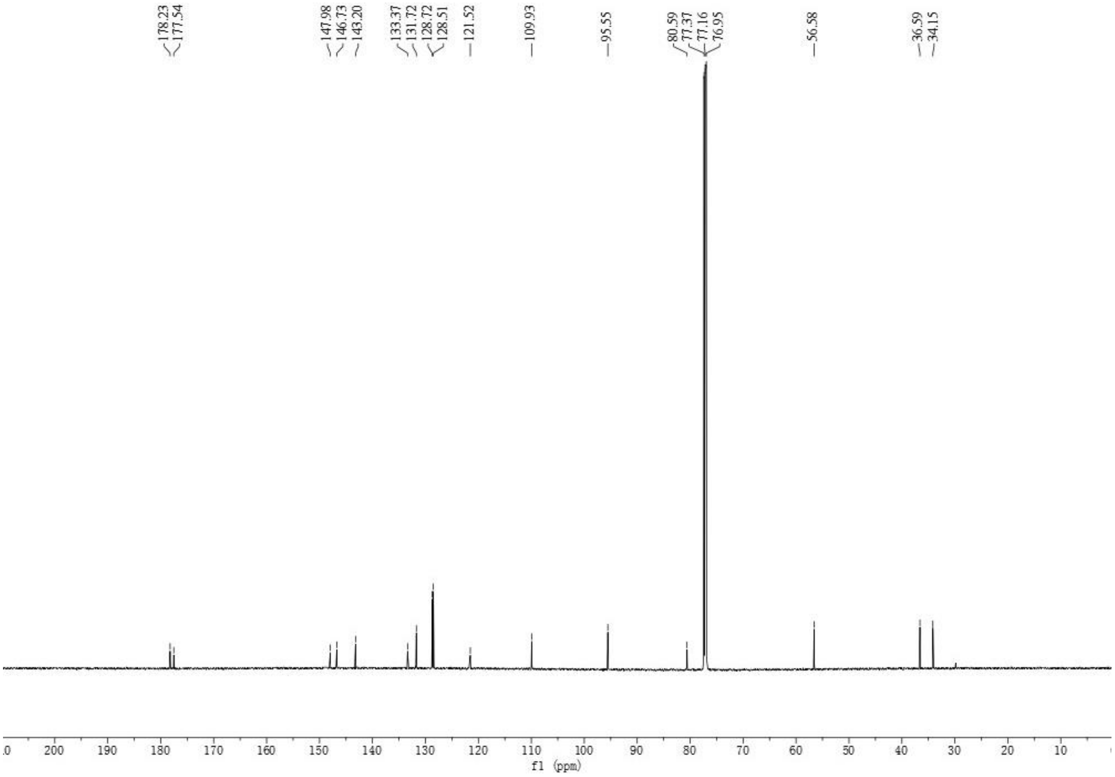

Supplementary Figure 35. ^{1}H NMR, and ^{13}C NMR spectra of compound **4m**

^{1}H NMR of 4n

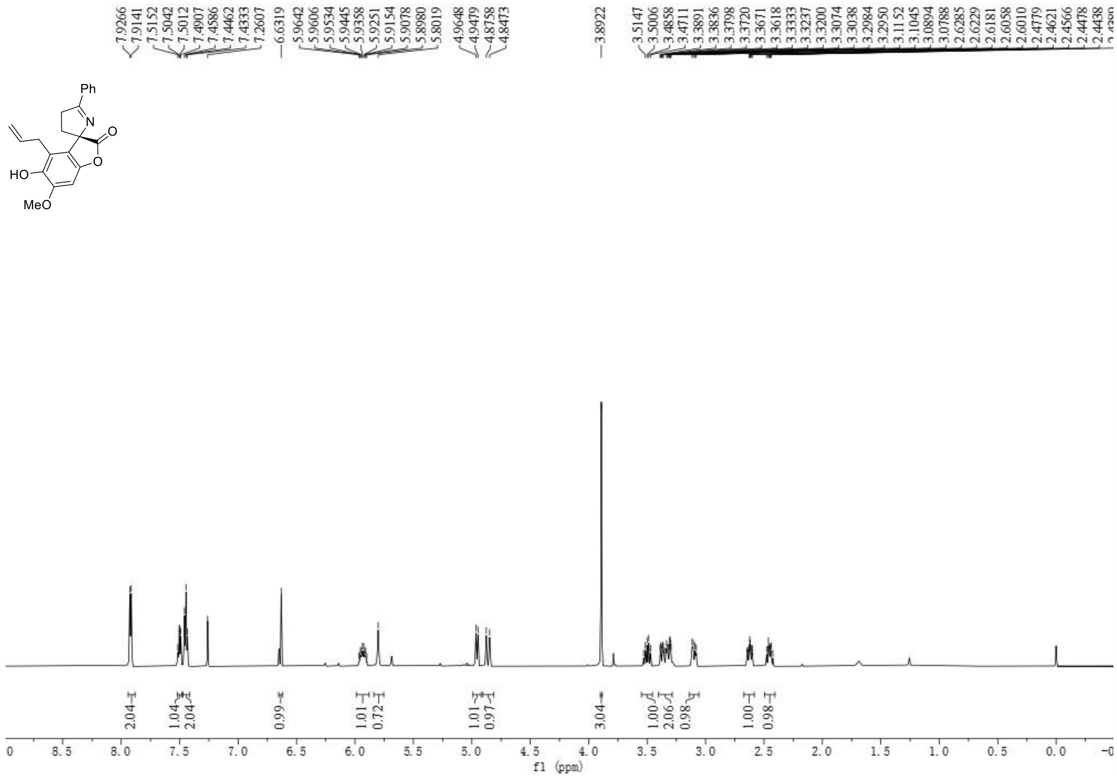

^{13}C NMR of 4n

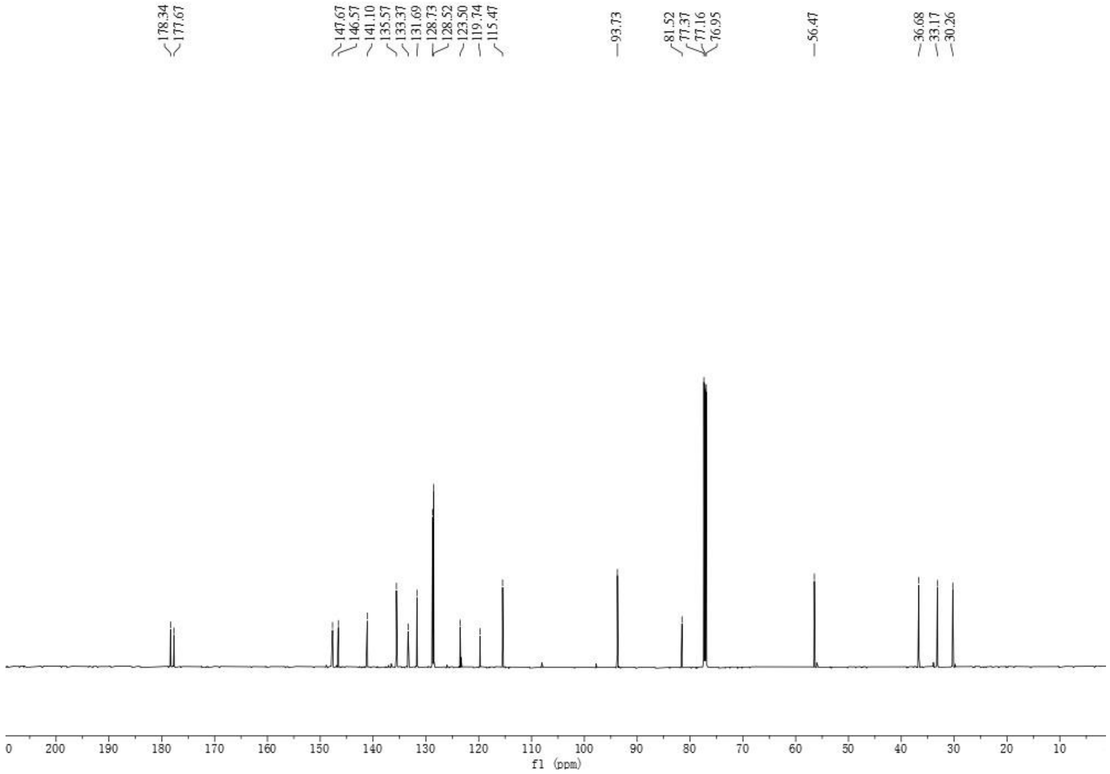

Supplementary Figure 36. ^{1}H NMR, and ^{13}C NMR spectra of compound **4n**

^{1}H NMR of 4o

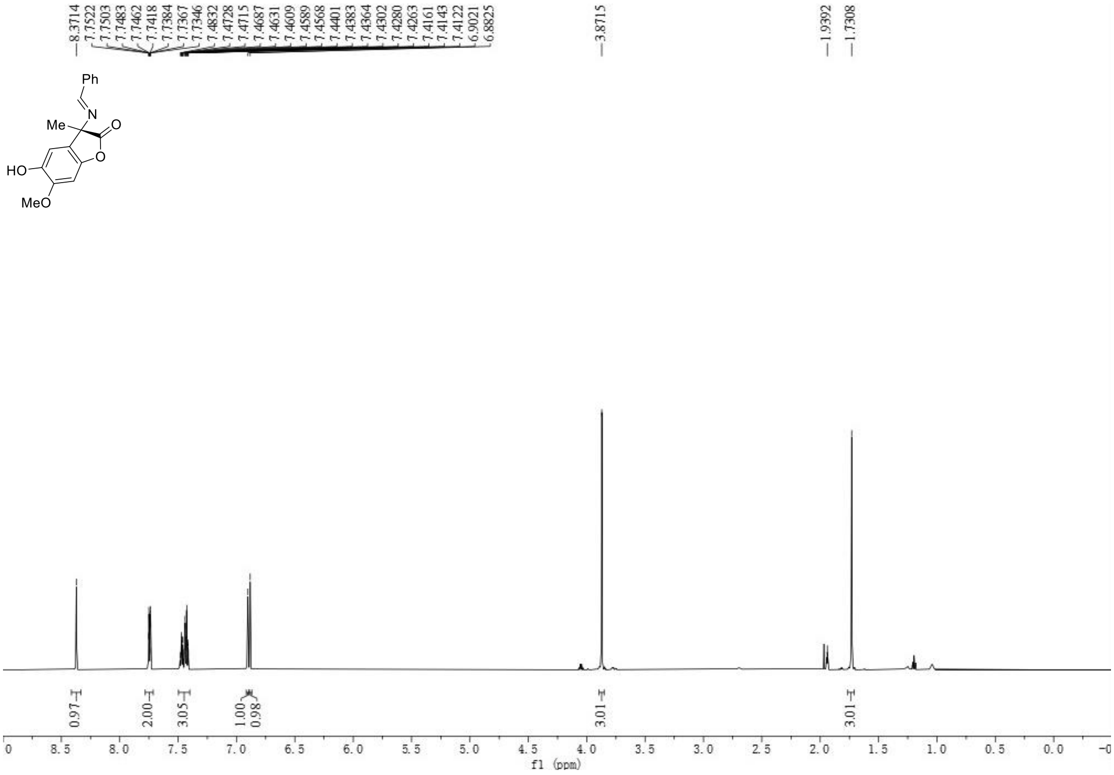

^{13}C NMR of 4o

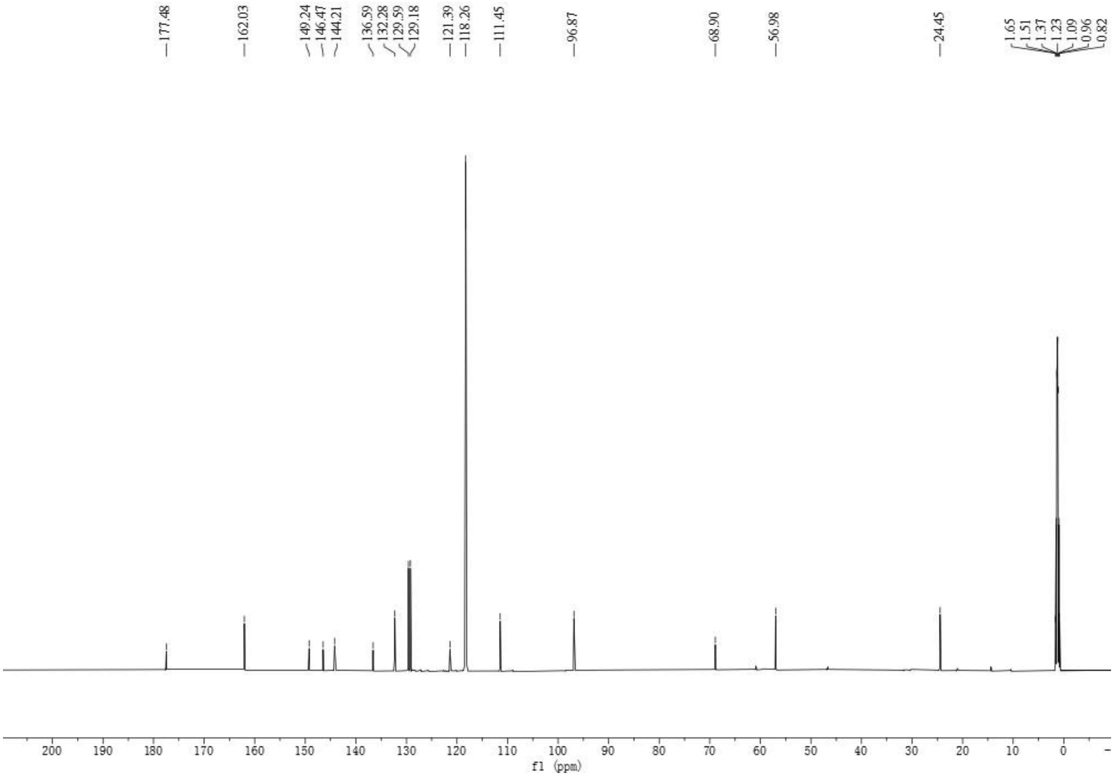

Supplementary Figure 37. ^{1}H NMR, and ^{13}C NMR spectra of compound **4o**

^{1}H NMR of 5a

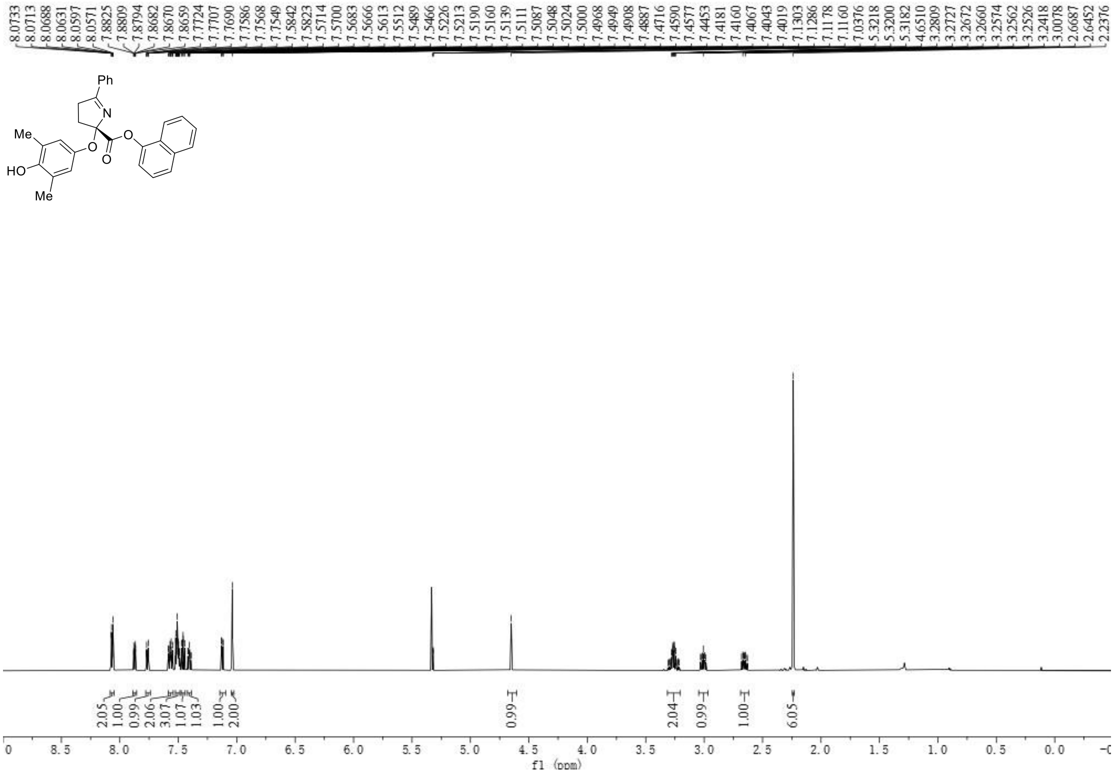

^{13}C NMR of 5a

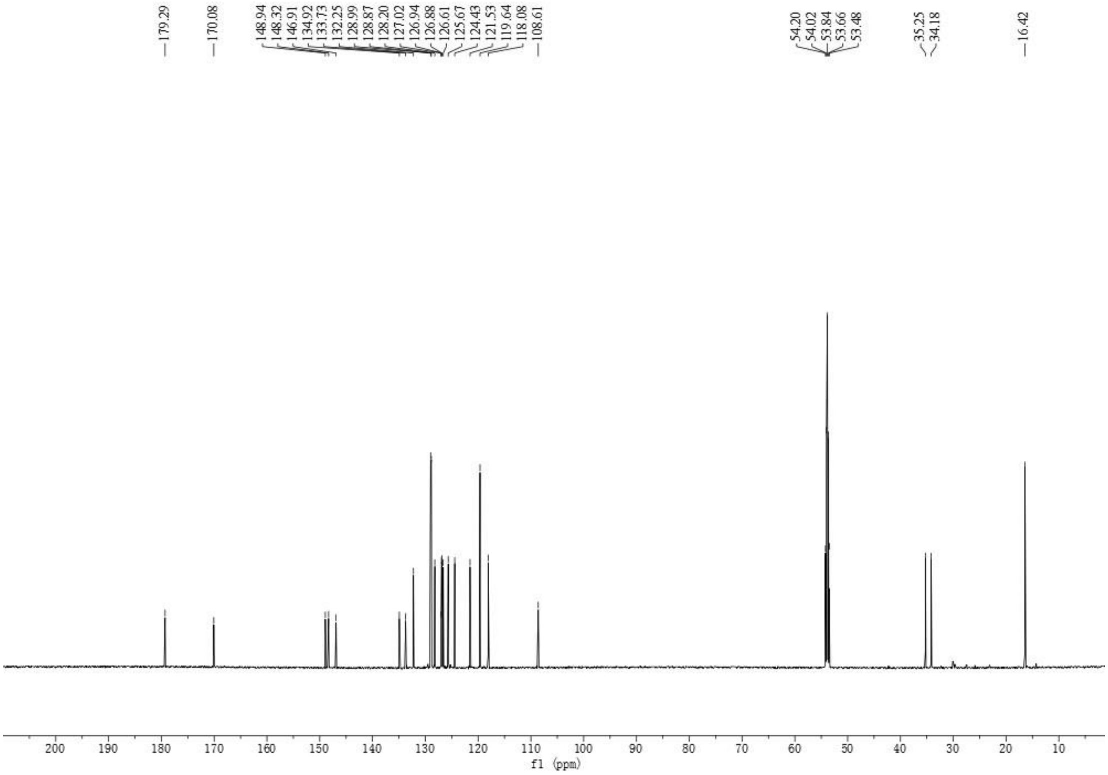

Supplementary Figure 38. ^{1}H NMR, and ^{13}C NMR spectra of compound **5a**

^{1}H NMR of 5b

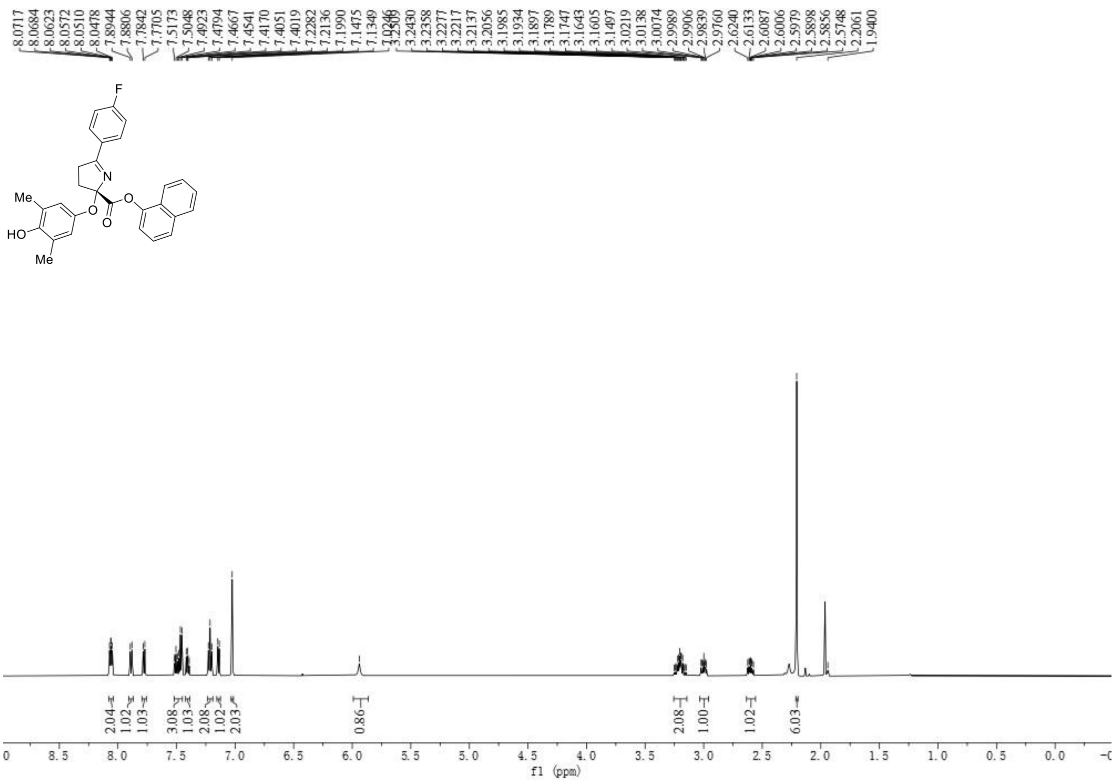

^{19}F NMR of 5b

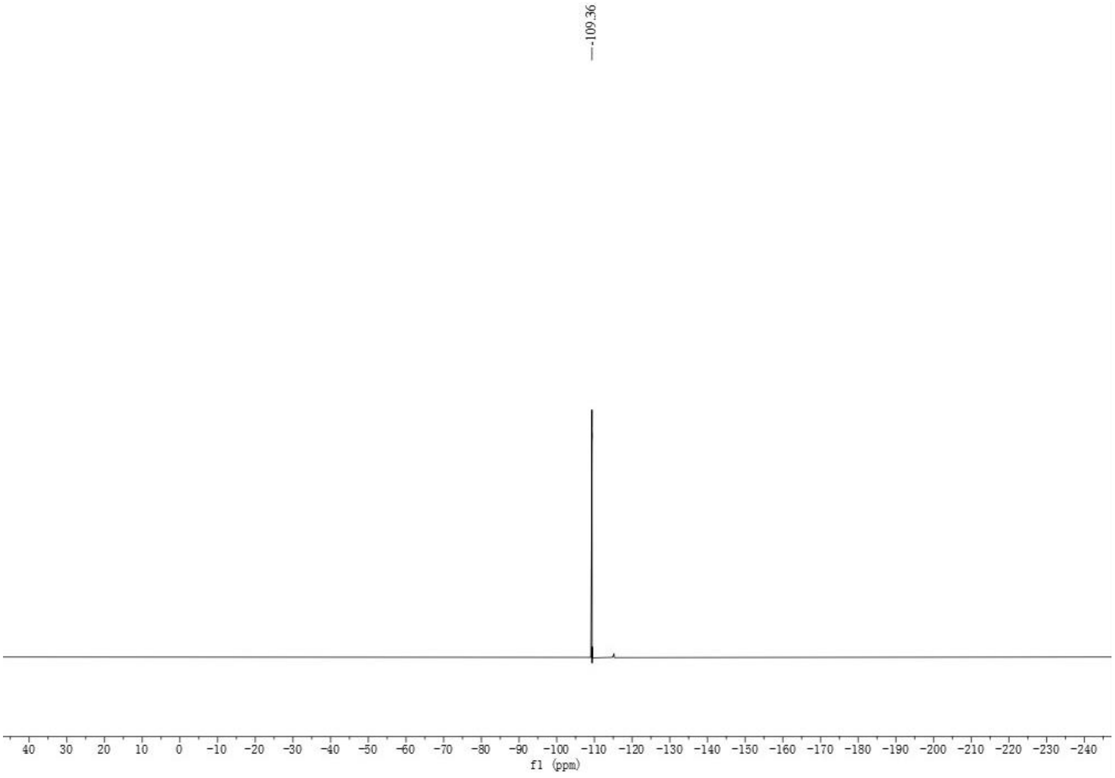

^{13}C NMR of 5b

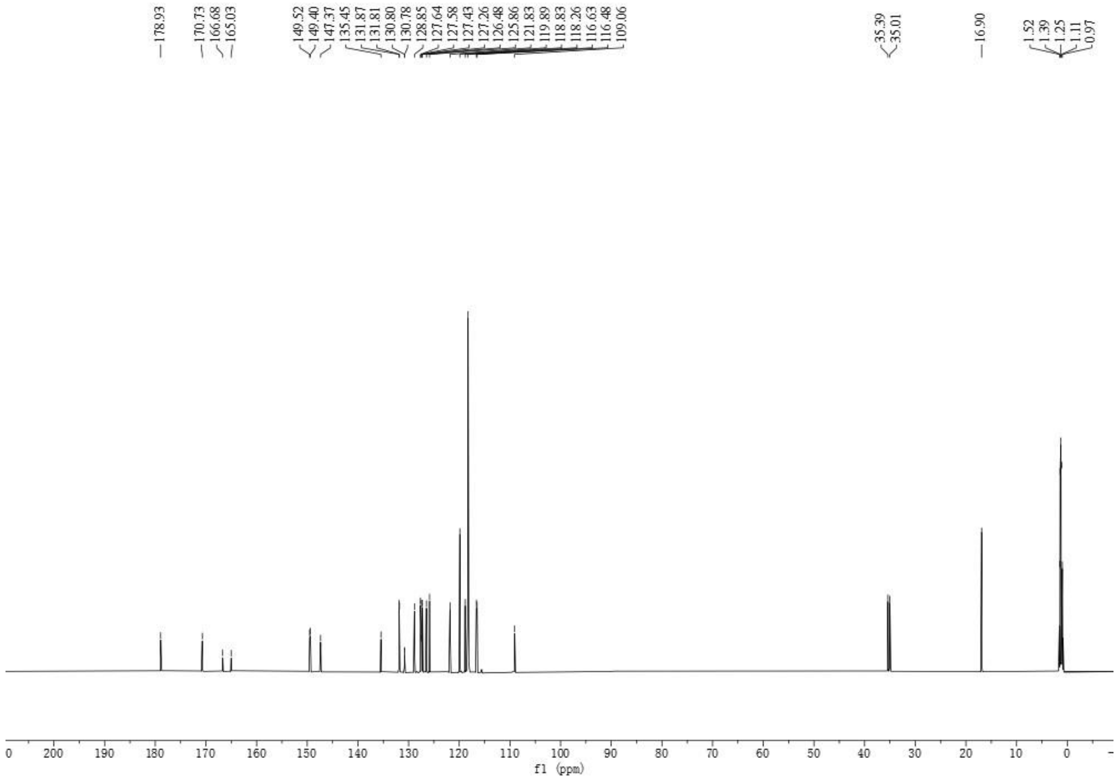

Supplementary Figure 39. ^{1}H NMR, ^{13}C NMR, and ^{19}F NMR spectra of compound **5b**

^{1}H NMR of 5c

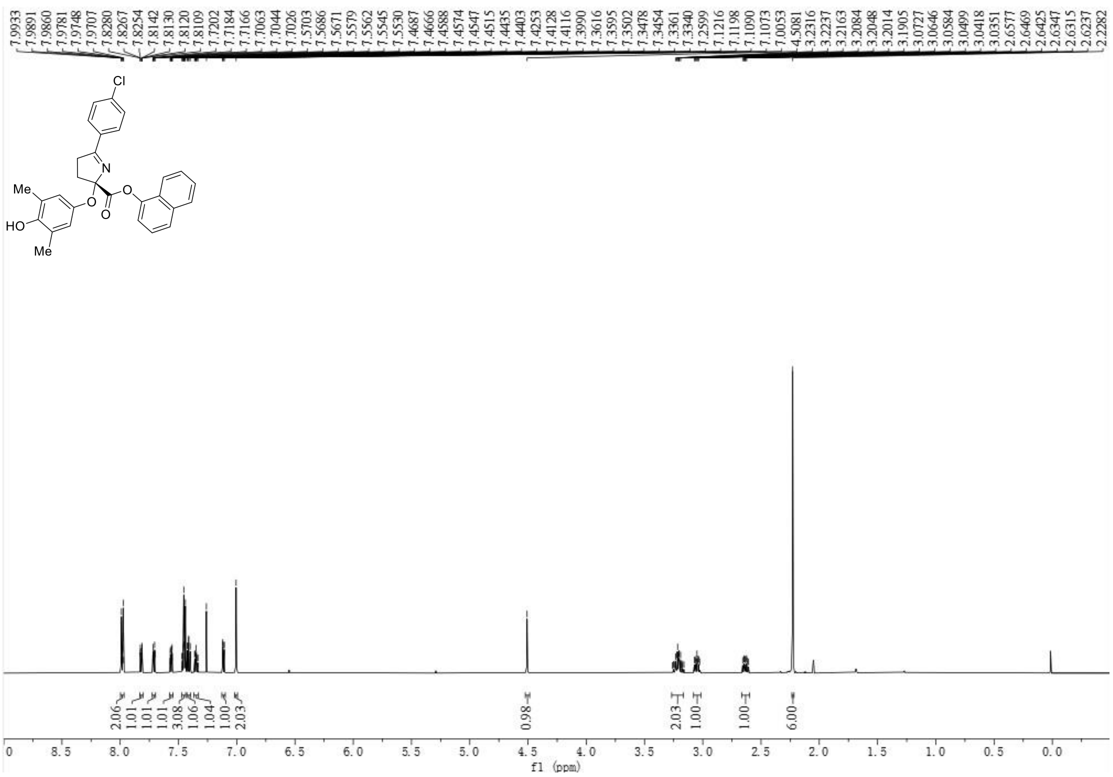

^{13}C NMR of 5c

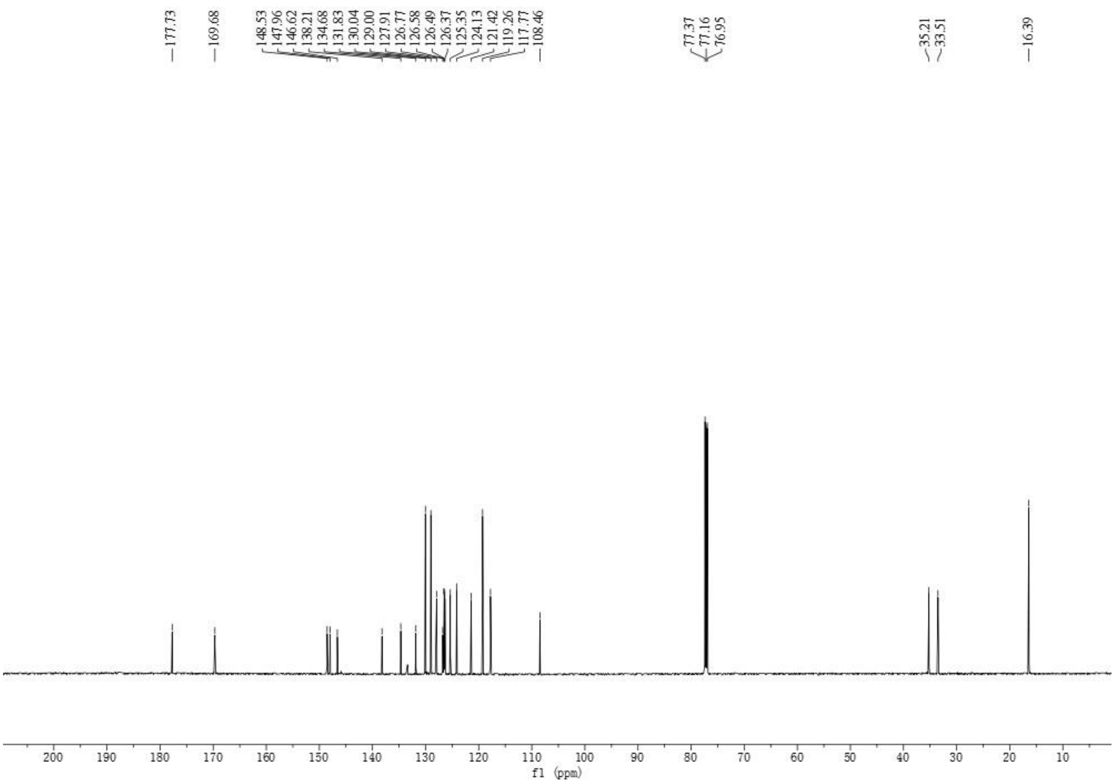

Supplementary Figure 40. ^{1}H NMR, and ^{13}C NMR spectra of compound **5c**

^{1}H NMR of 5d

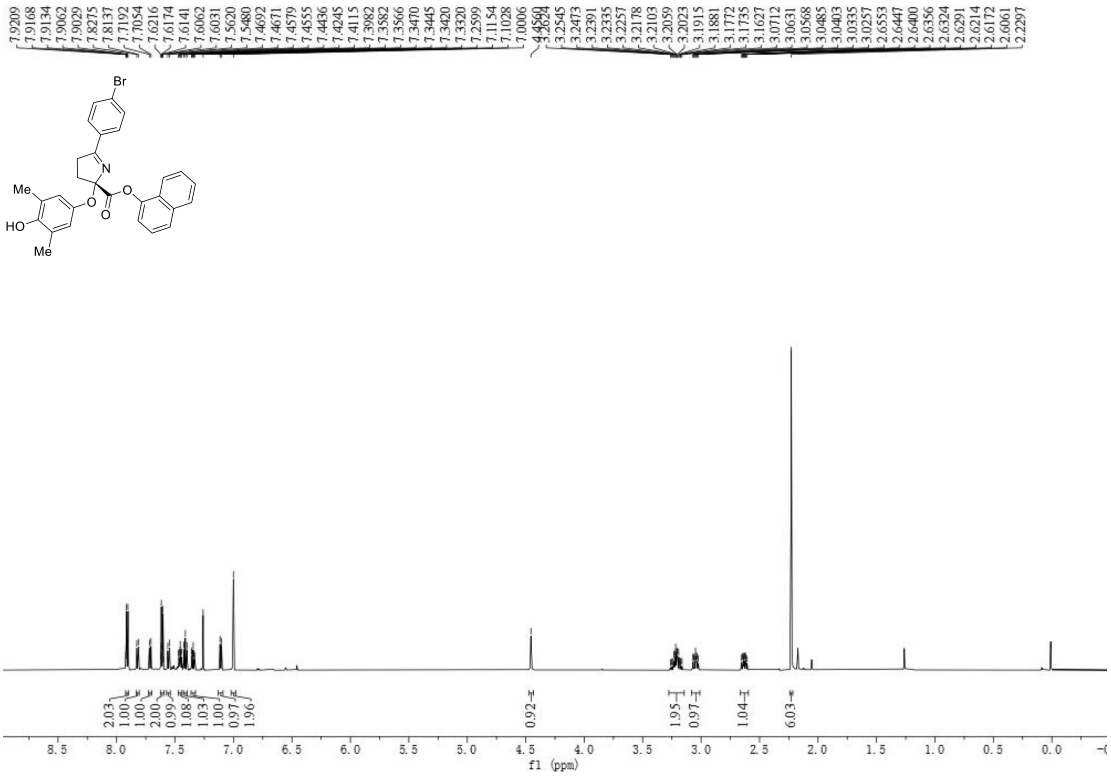

^{13}C NMR of 5d

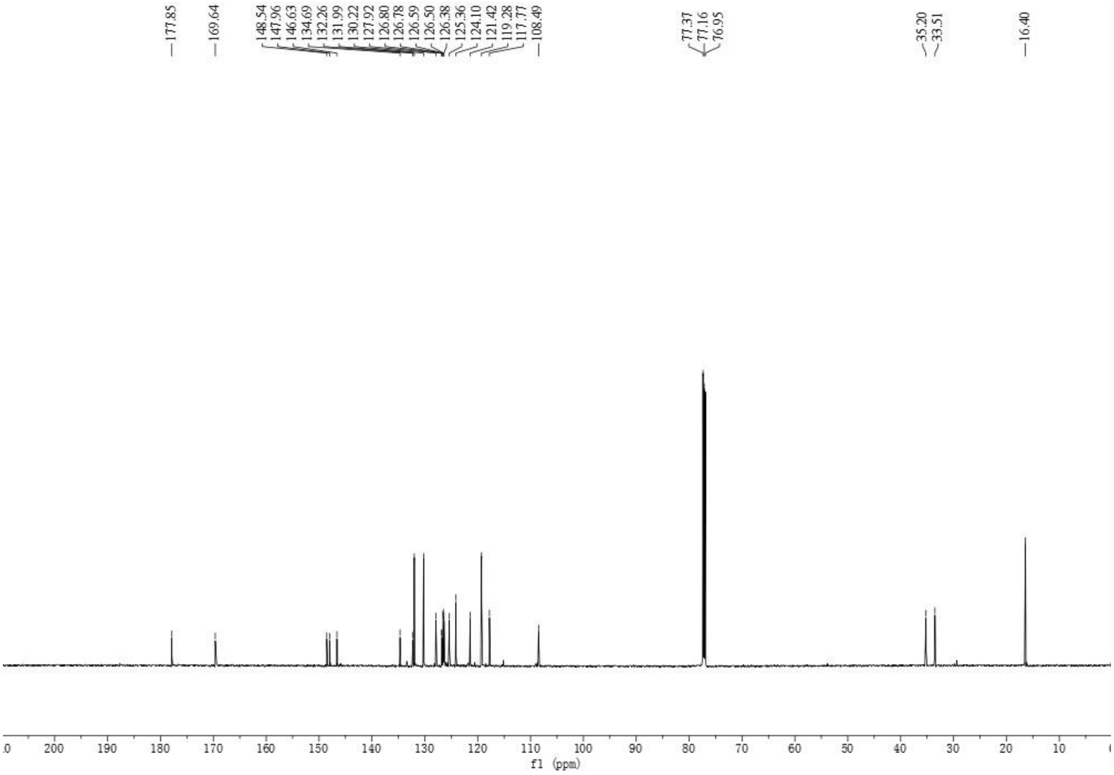

Supplementary Figure 41. ^{1}H NMR, and ^{13}C NMR spectra of compound **5d**

^{1}H NMR of 5e

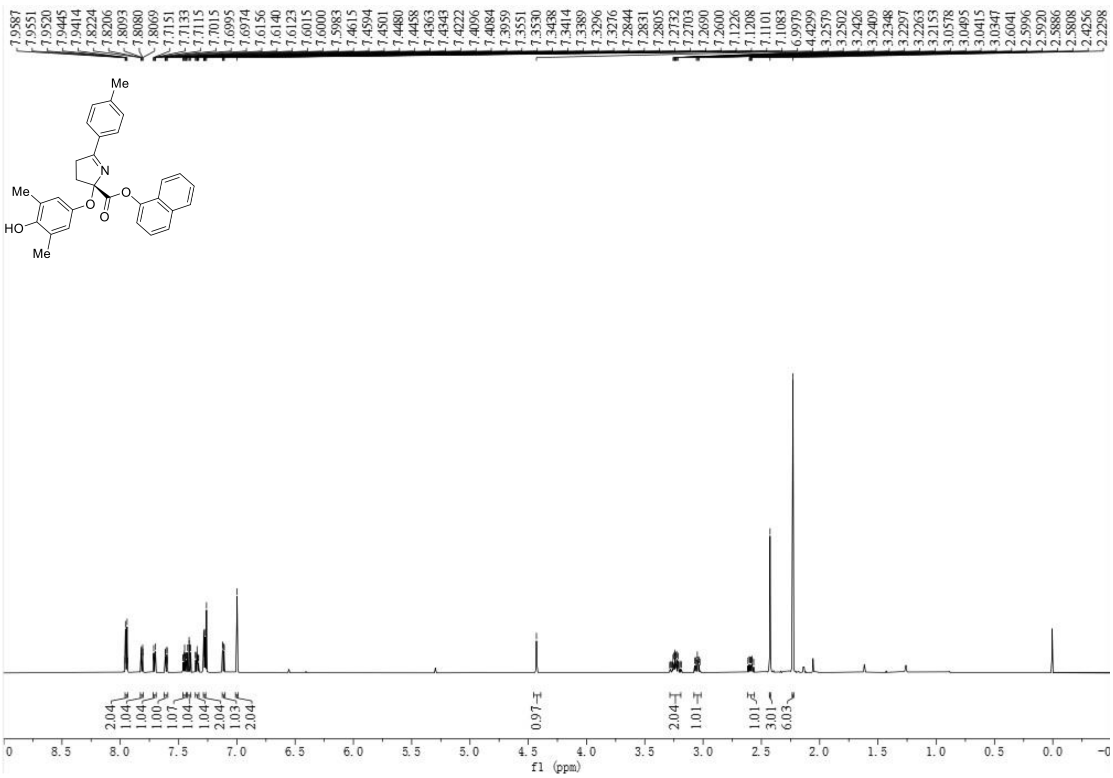

^{13}C NMR of 5e

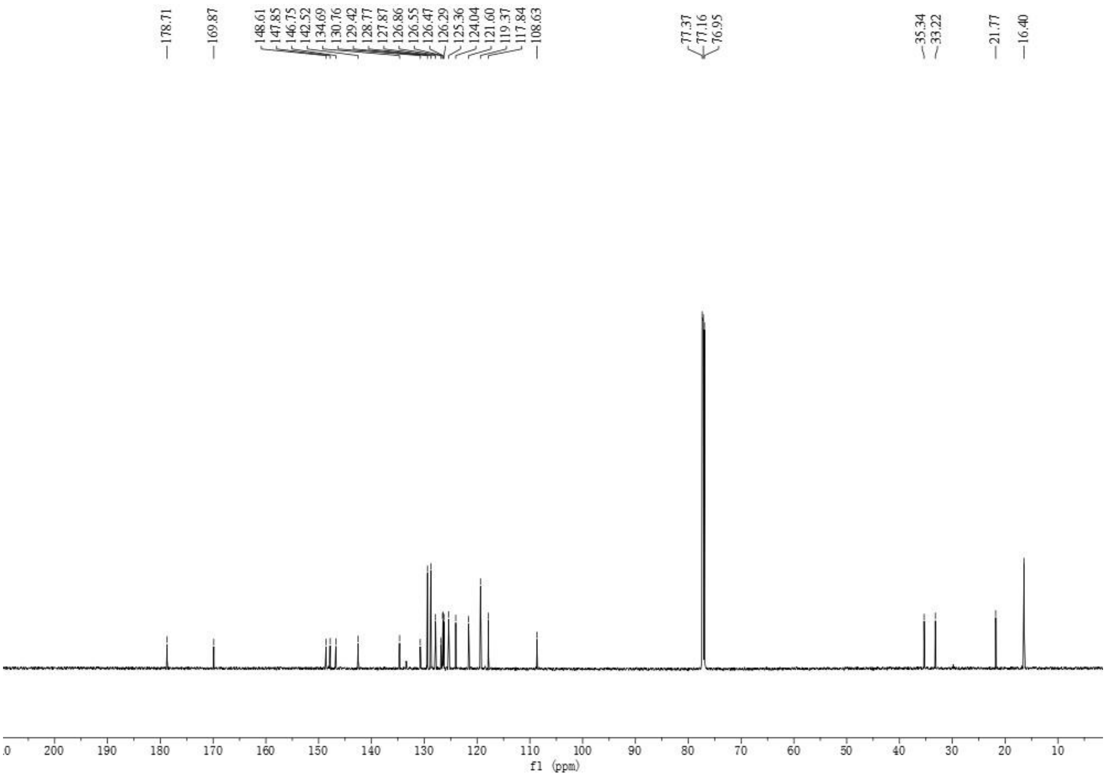

Supplementary Figure 42. ^{1}H NMR, and ^{13}C NMR spectra of compound **5e**

^{1}H NMR of 5f

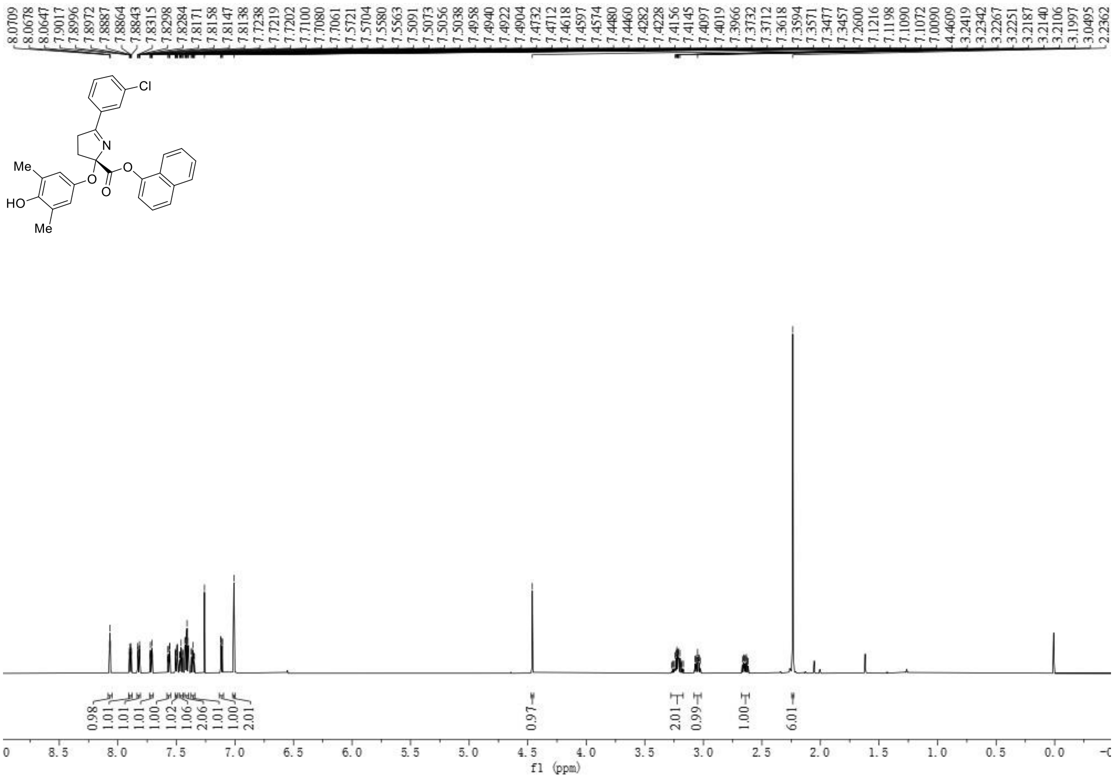

^{13}C NMR of 5f

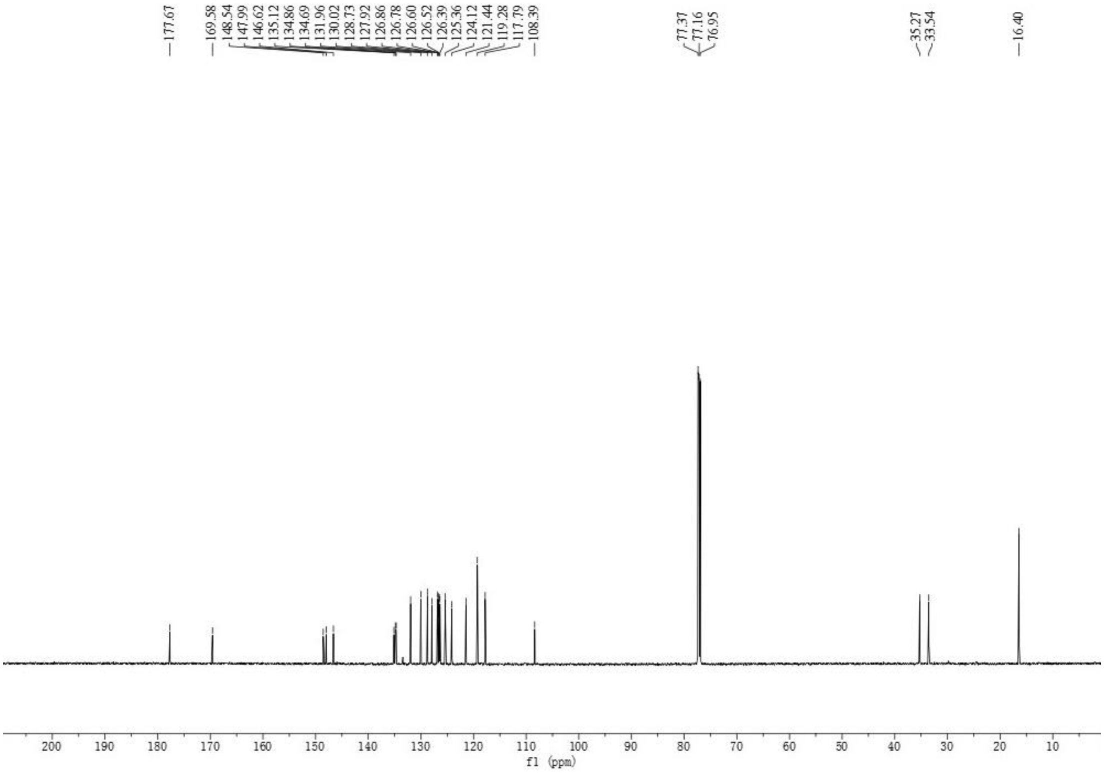

Supplementary Figure 43. ^{1}H NMR, and ^{13}C NMR spectra of compound **5f**

^{1}H NMR of 5g

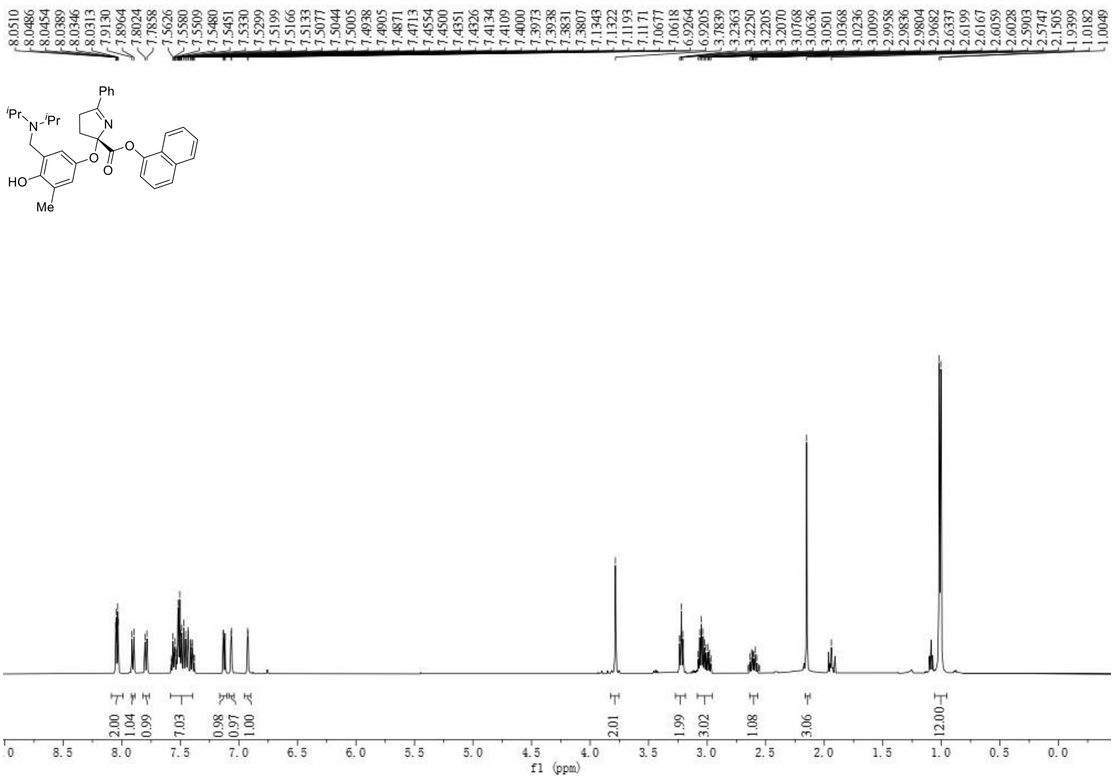

^{13}C NMR of 5g

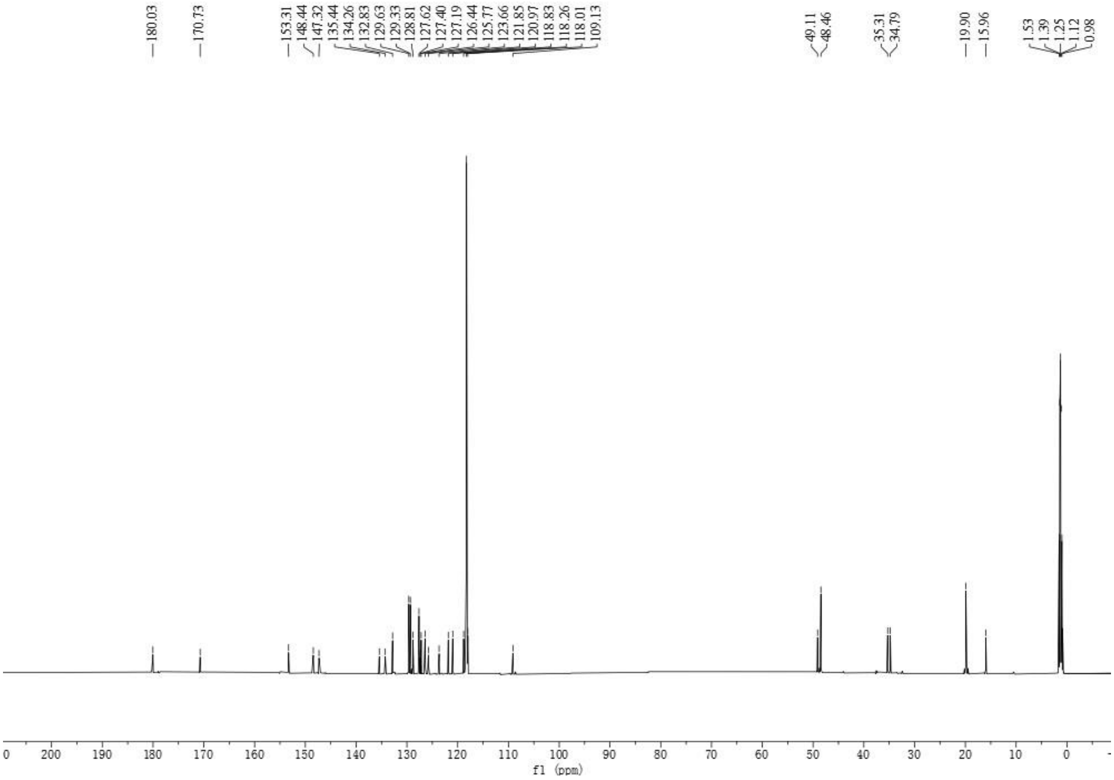

Supplementary Figure 44. ^{1}H NMR, and ^{13}C NMR spectra of compound **5g**

^{1}H NMR of 5h

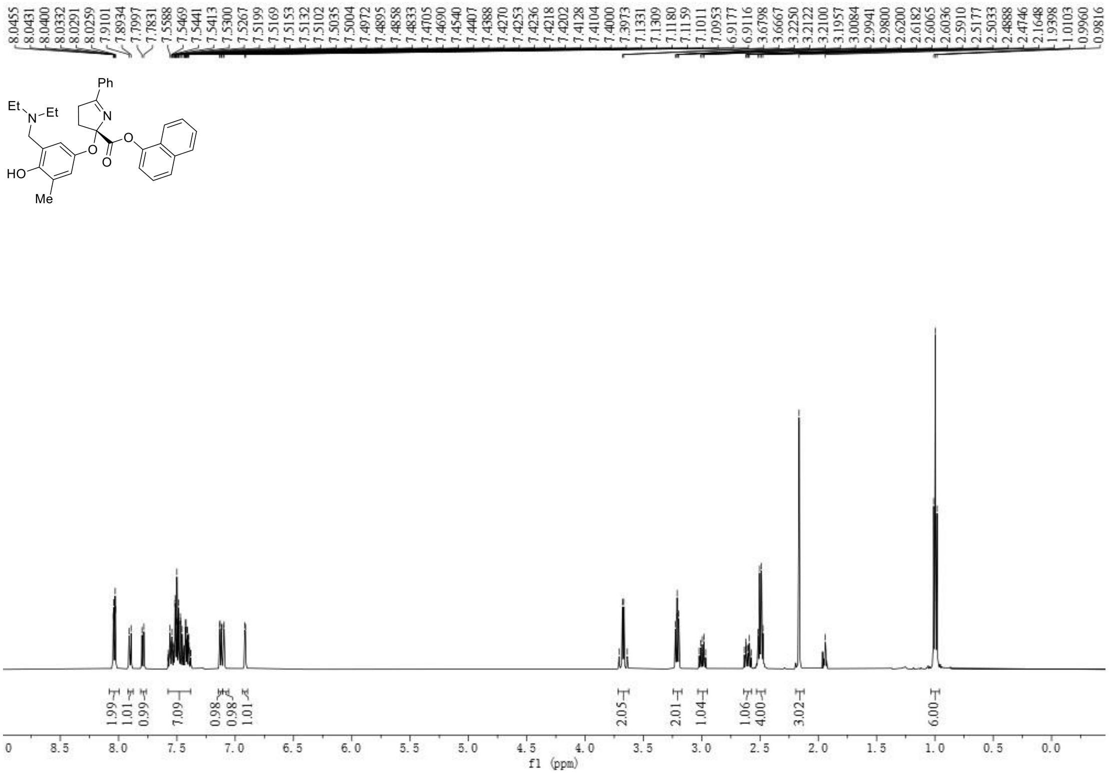

^{13}C NMR of 5h

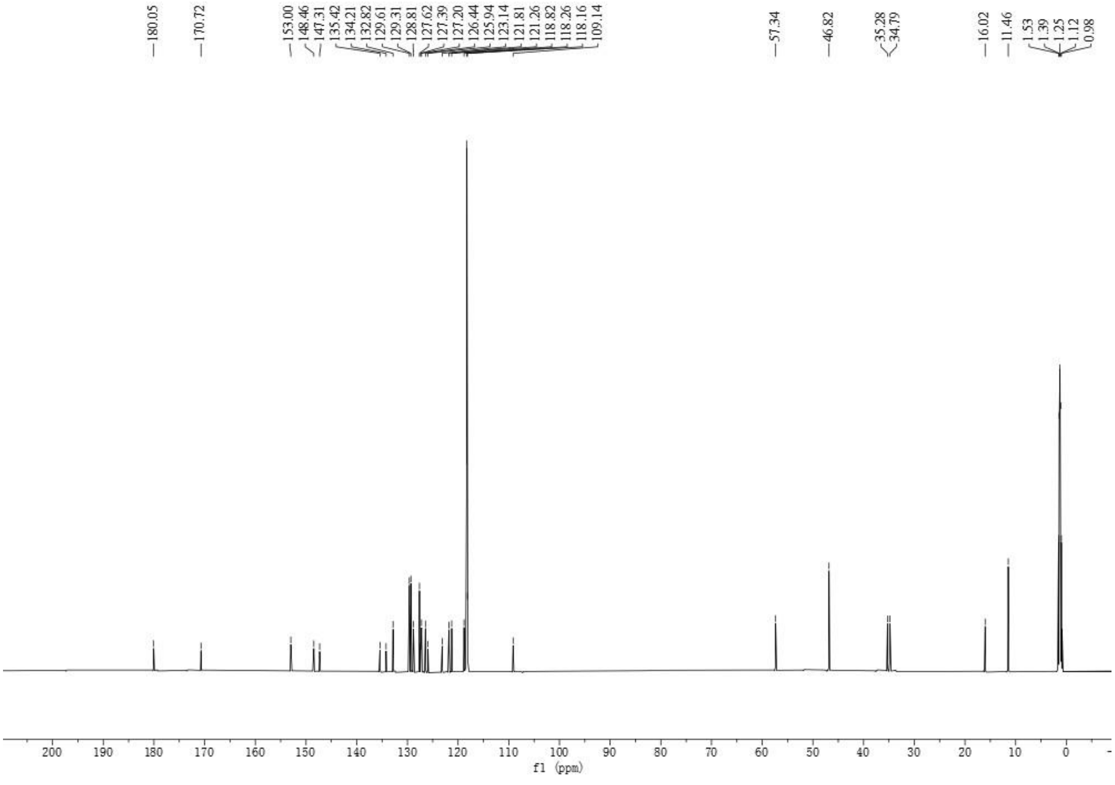

Supplementary Figure 45. ^{1}H NMR, and ^{13}C NMR spectra of compound **5h**

^{1}H NMR of 5i

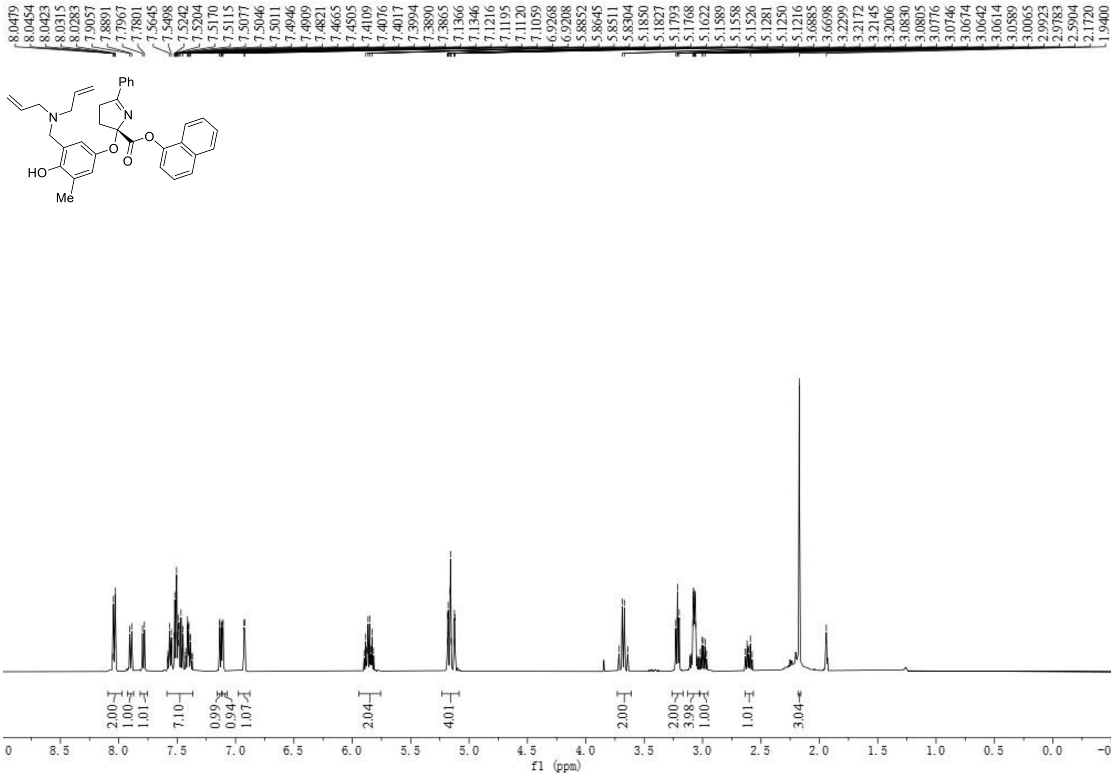

^{13}C NMR of 5i

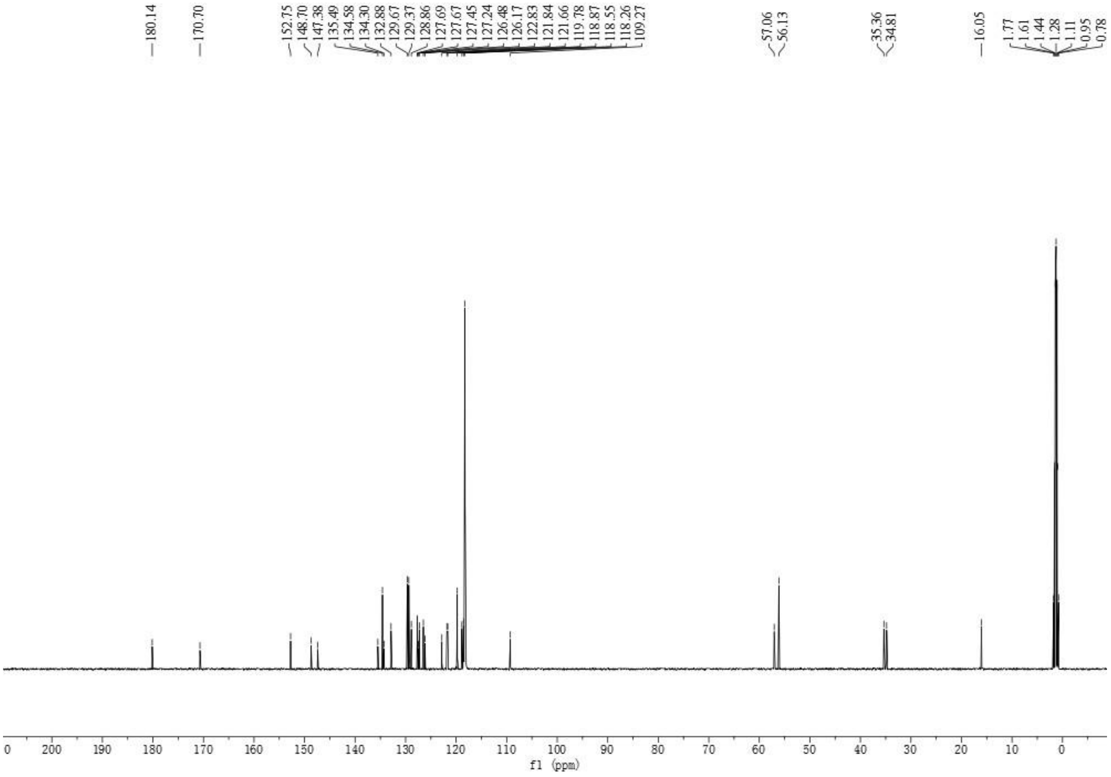

Supplementary Figure 46. ^{1}H NMR, and ^{13}C NMR spectra of compound **5i**

^{1}H NMR of 7a

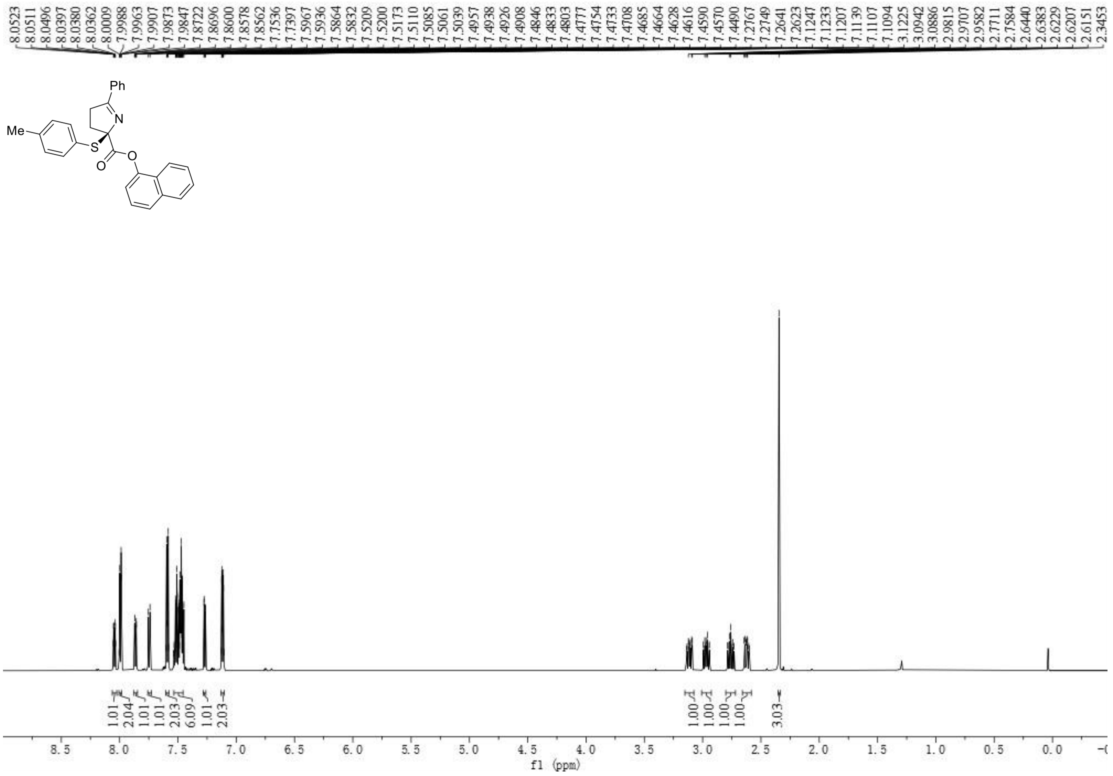

^{13}C NMR of 7a

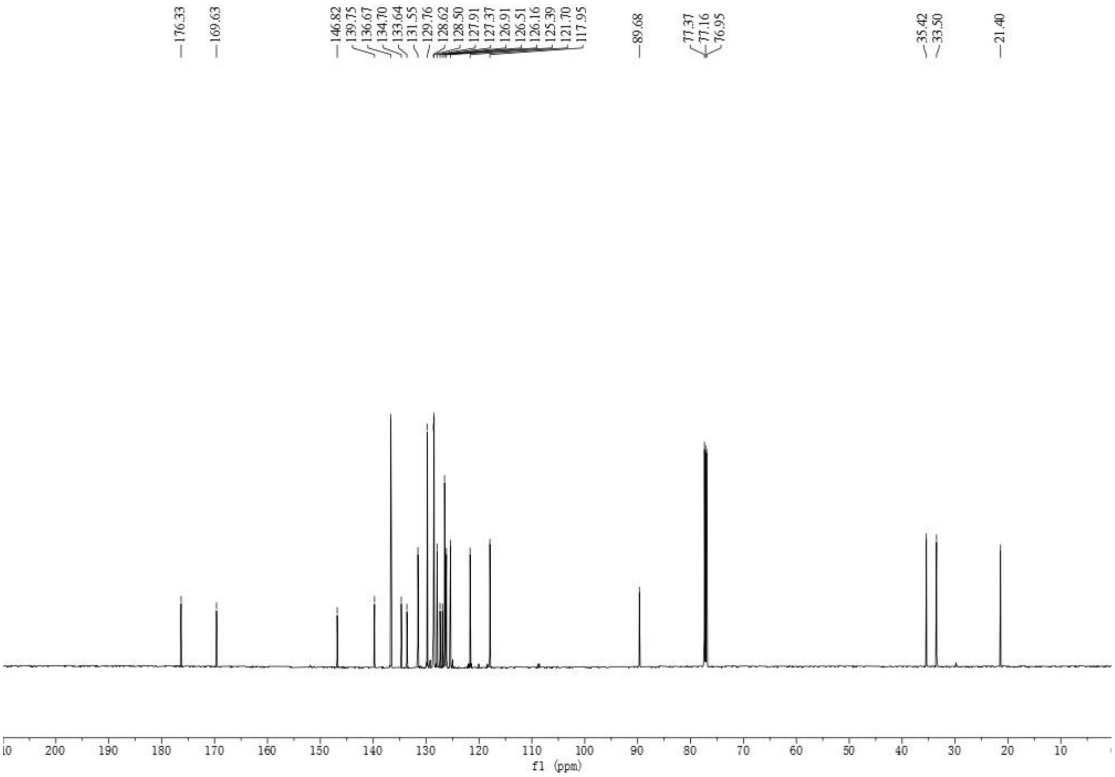

Supplementary Figure 47. ^{1}H NMR, and ^{13}C NMR spectra of compound **7a**

^{1}H NMR of 7b

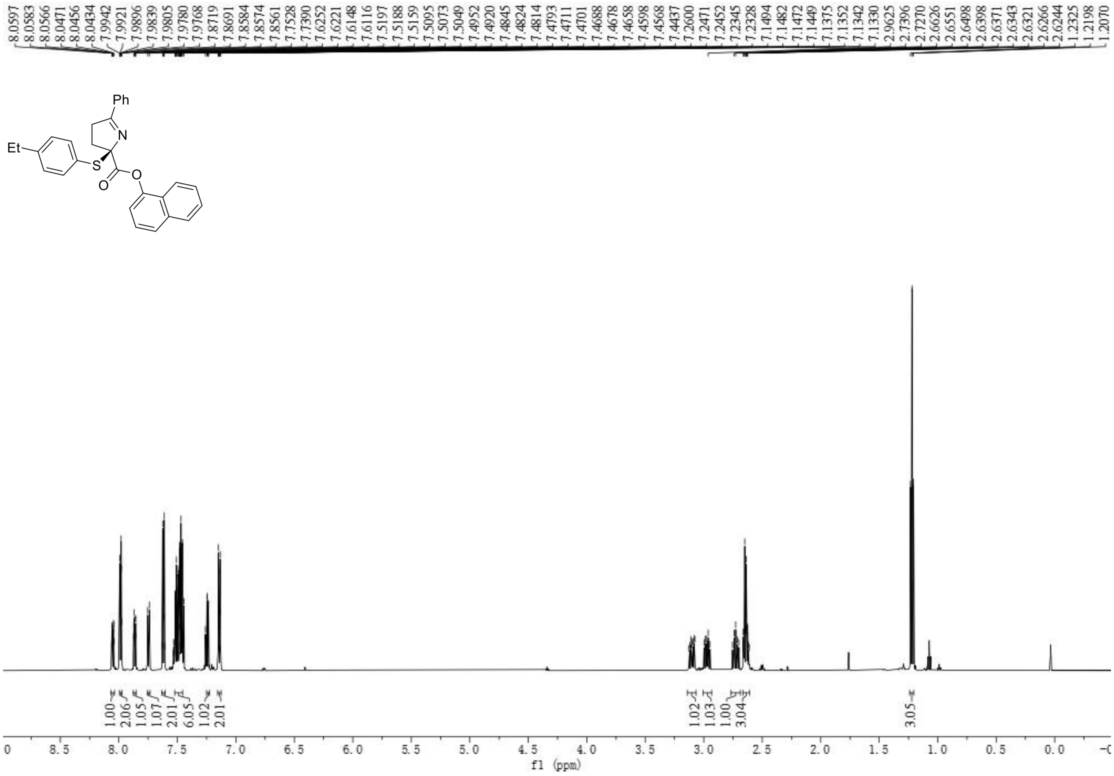

^{13}C NMR of 7b

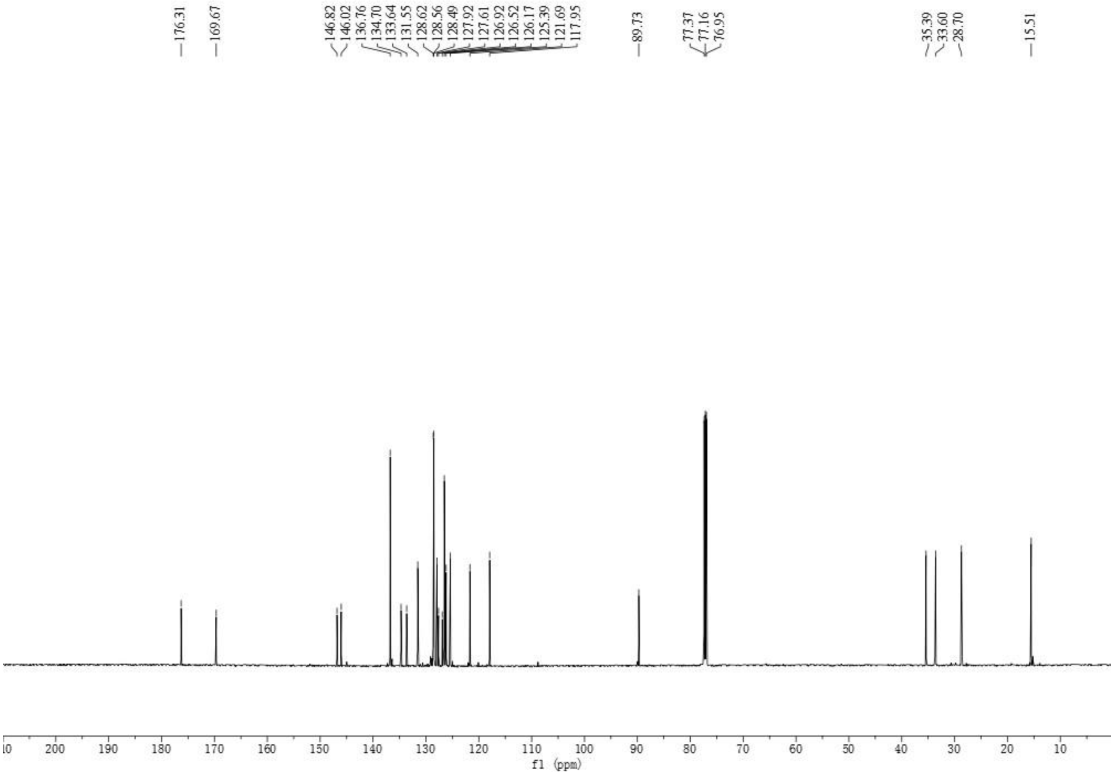

Supplementary Figure 48. ^{1}H NMR, and ^{13}C NMR spectra of compound **7b**

^{1}H NMR of 7c

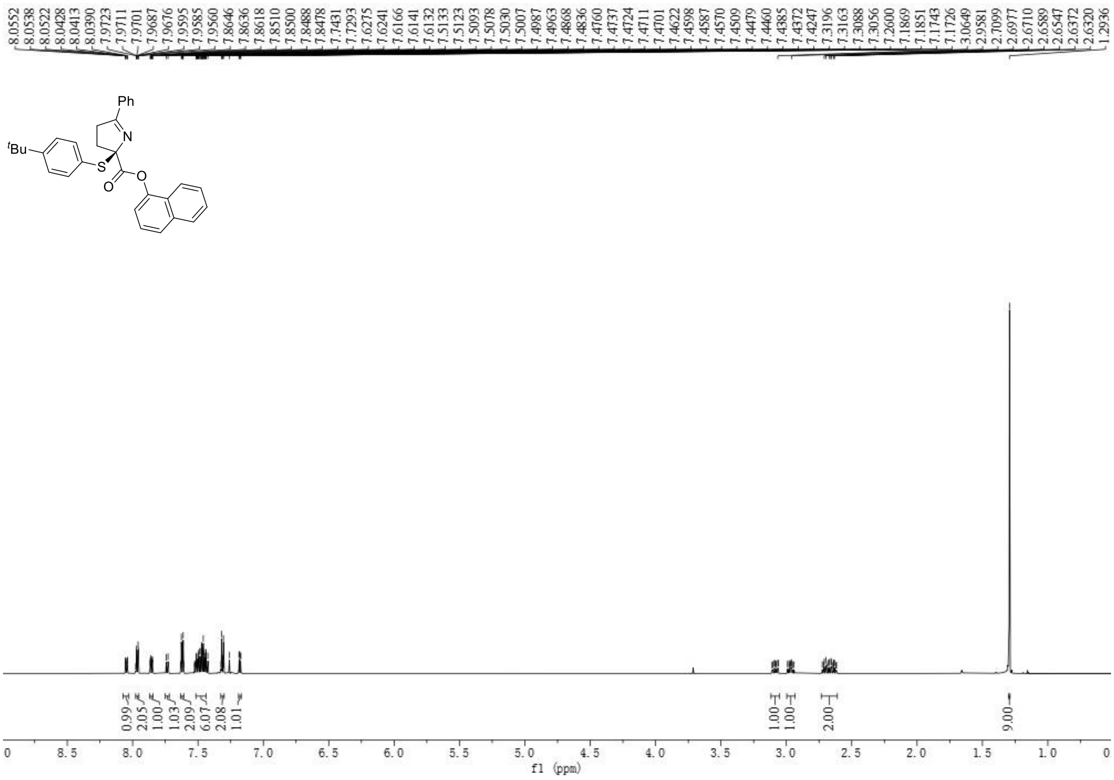

^{13}C NMR of 7c

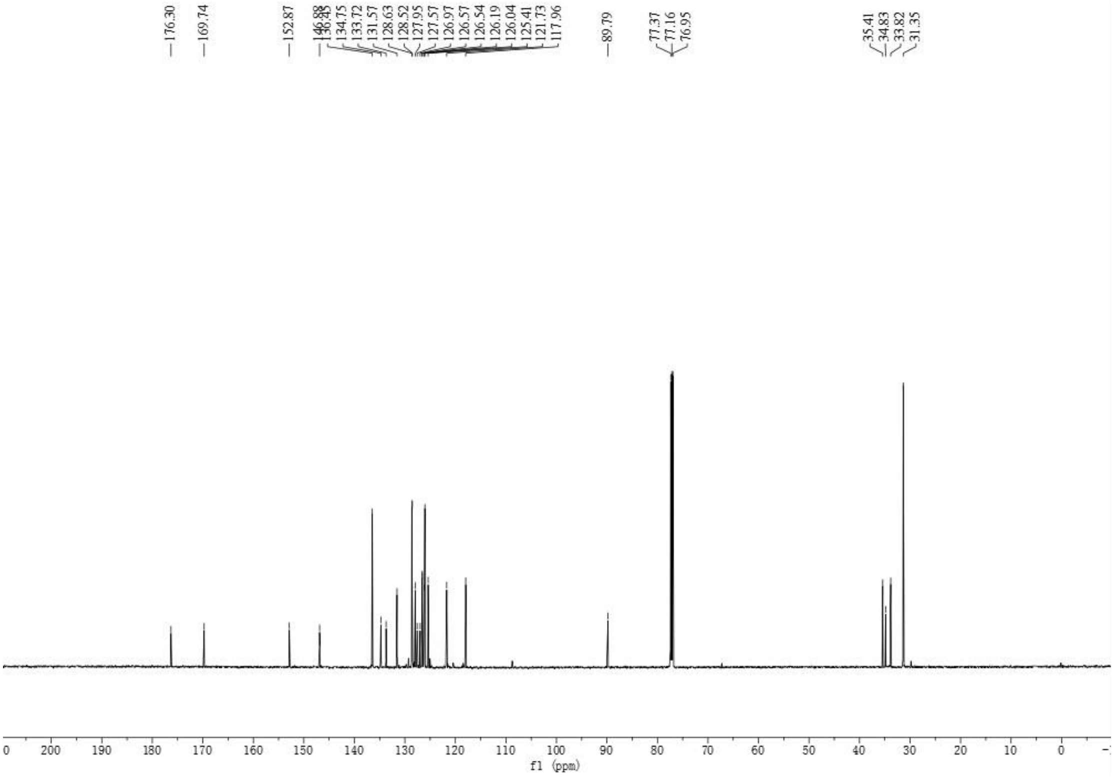

Supplementary Figure 49. ^{1}H NMR, and ^{13}C NMR spectra of compound **7c**

^{1}H NMR of 7d

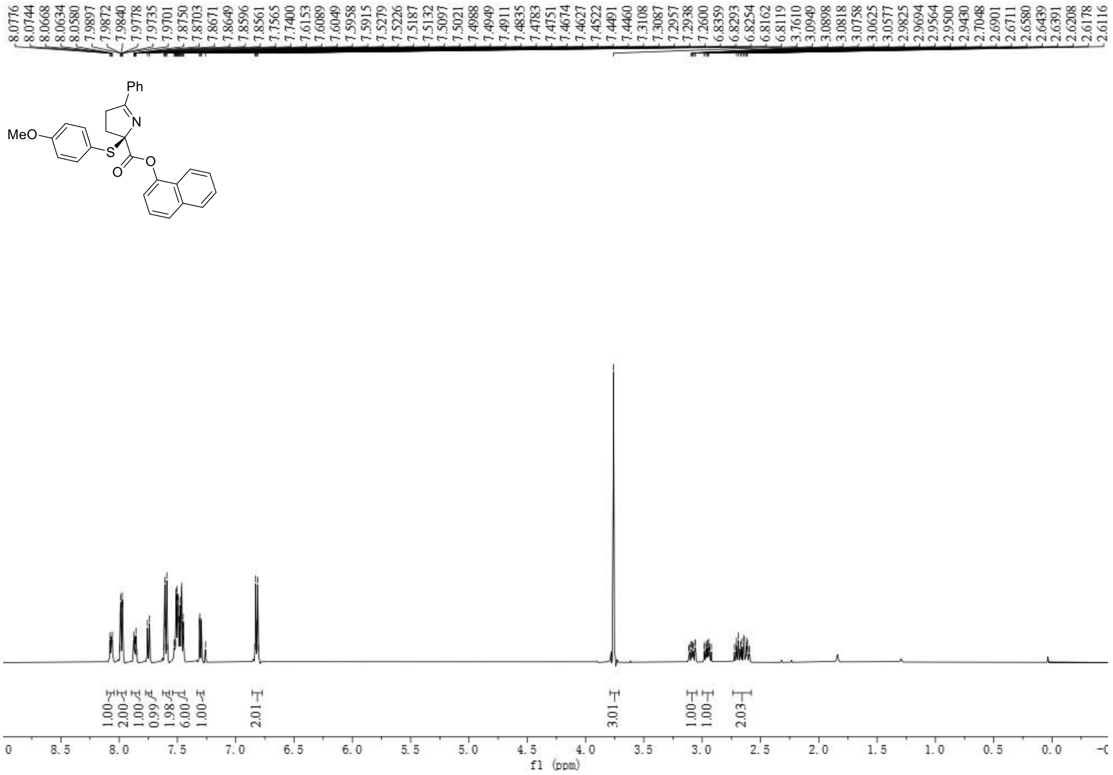

^{13}C NMR of 7d

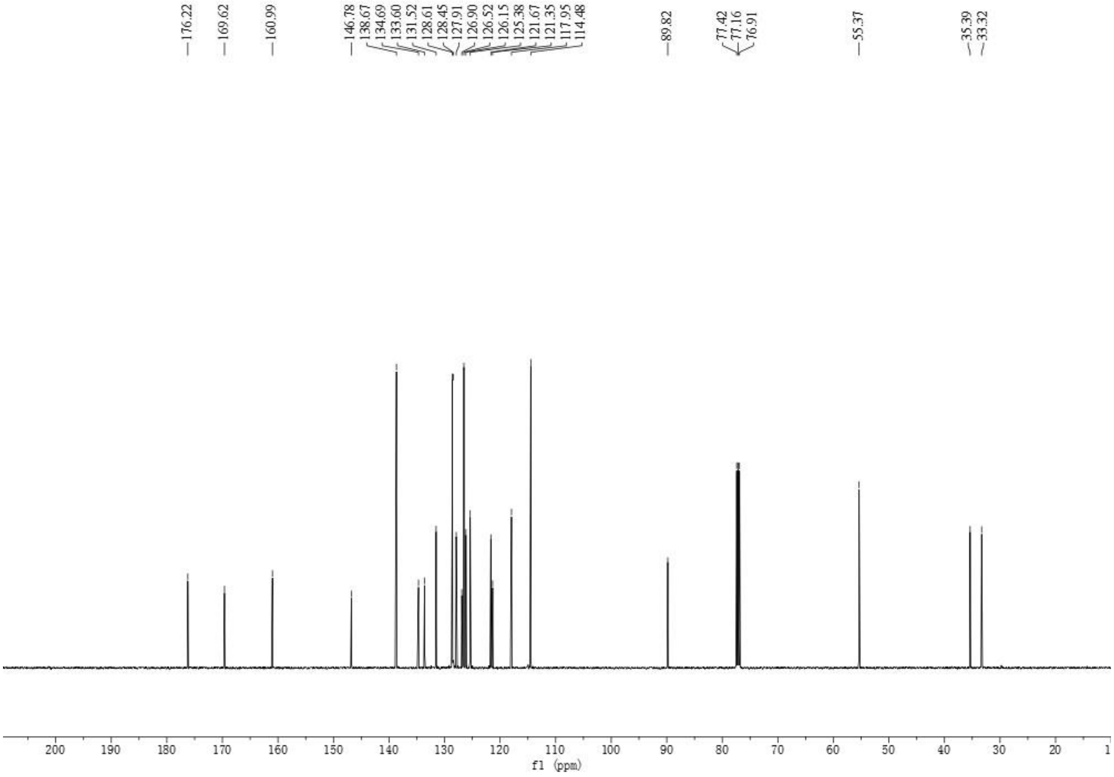

Supplementary Figure 50. ^{1}H NMR, and ^{13}C NMR spectra of compound **7d**

^{1}H NMR of 8

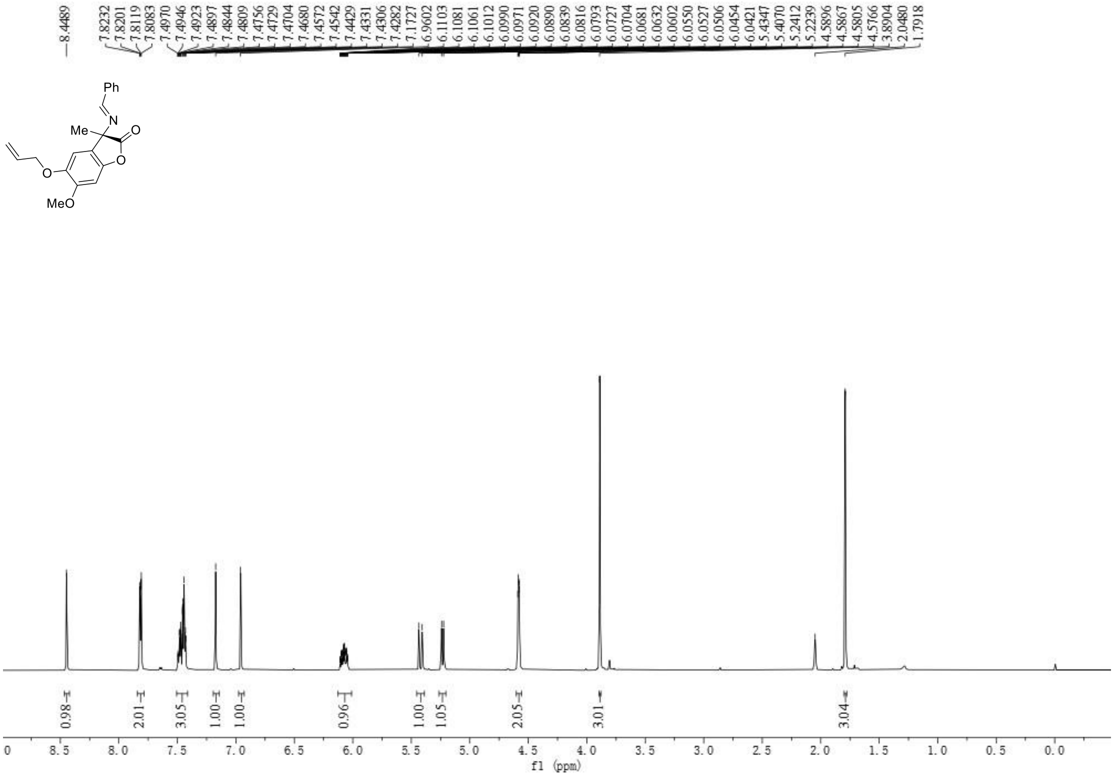

^{13}C NMR of 8

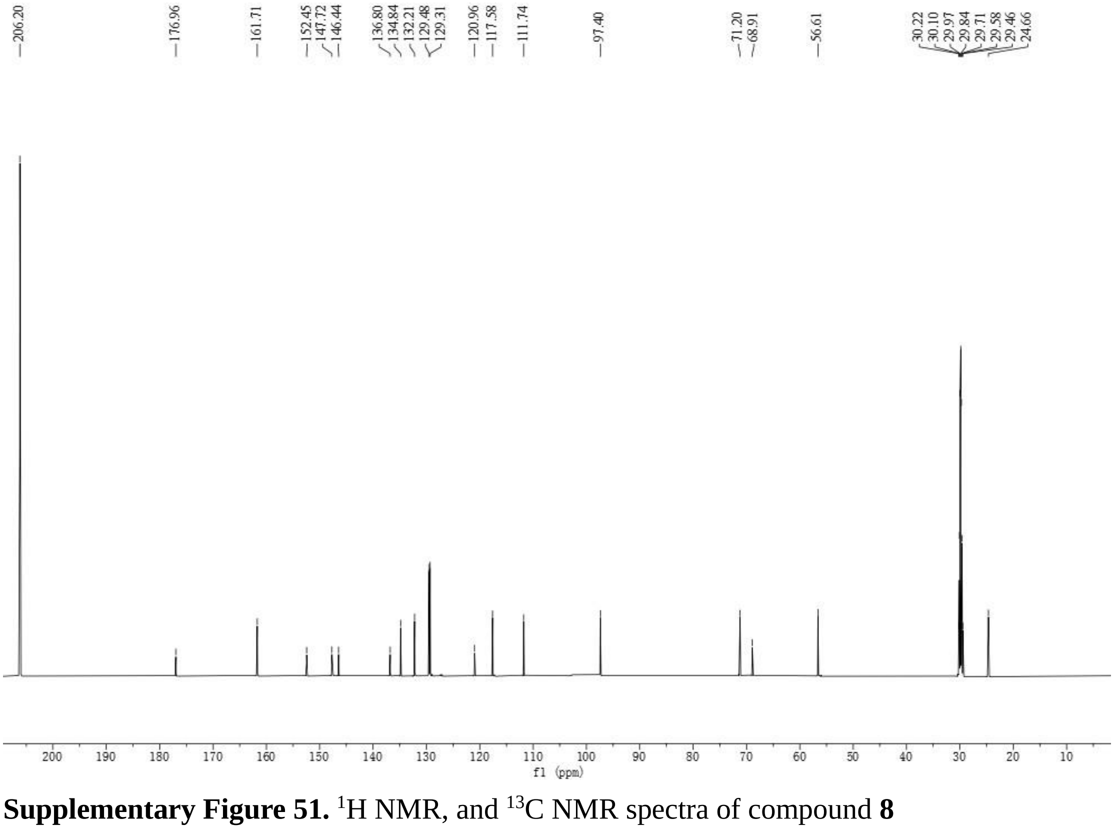

Supplementary Figure 51. ^{1}H NMR, and ^{13}C NMR spectra of compound **8**

^{1}H NMR of 9

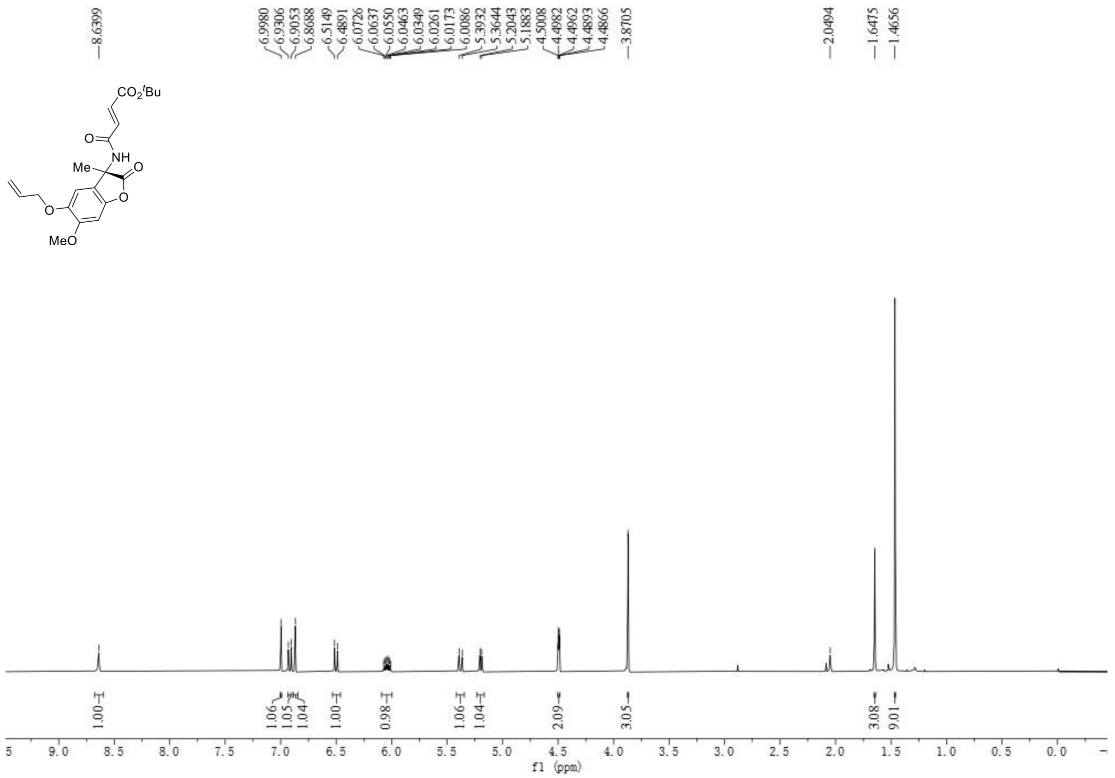

^{13}C NMR of 9

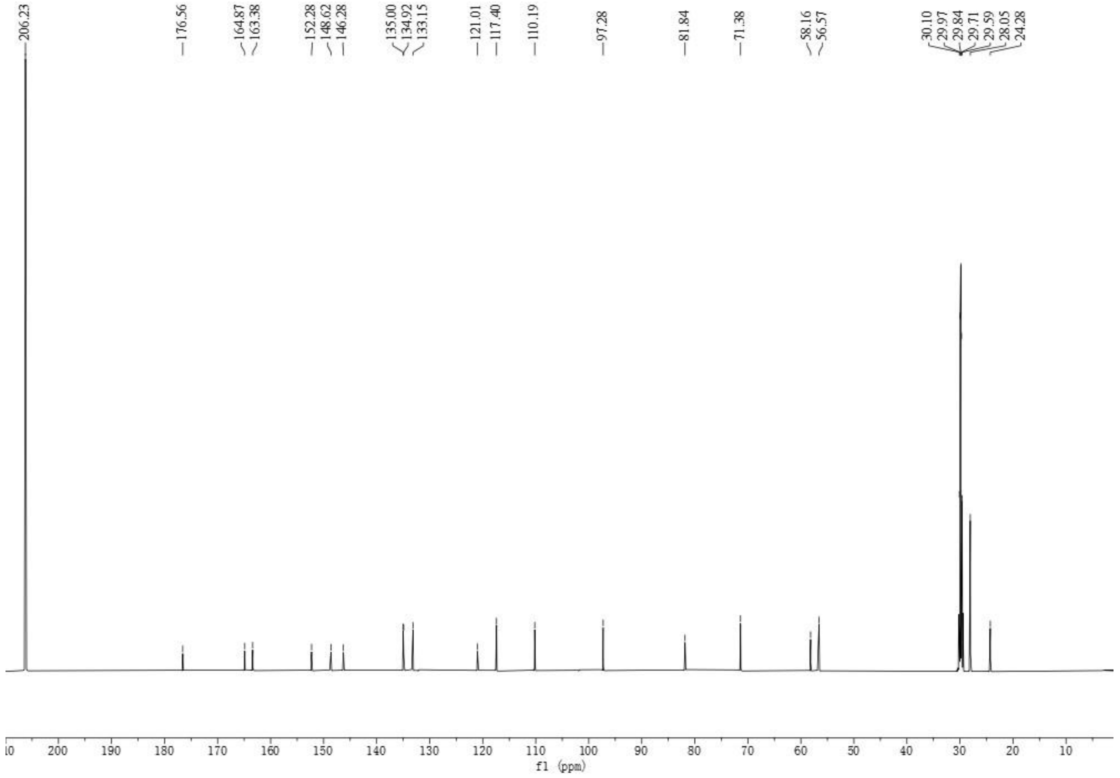

Supplementary Figure 52. ^{1}H NMR, and ^{13}C NMR spectra of compound **9**

^{1}H NMR of 11

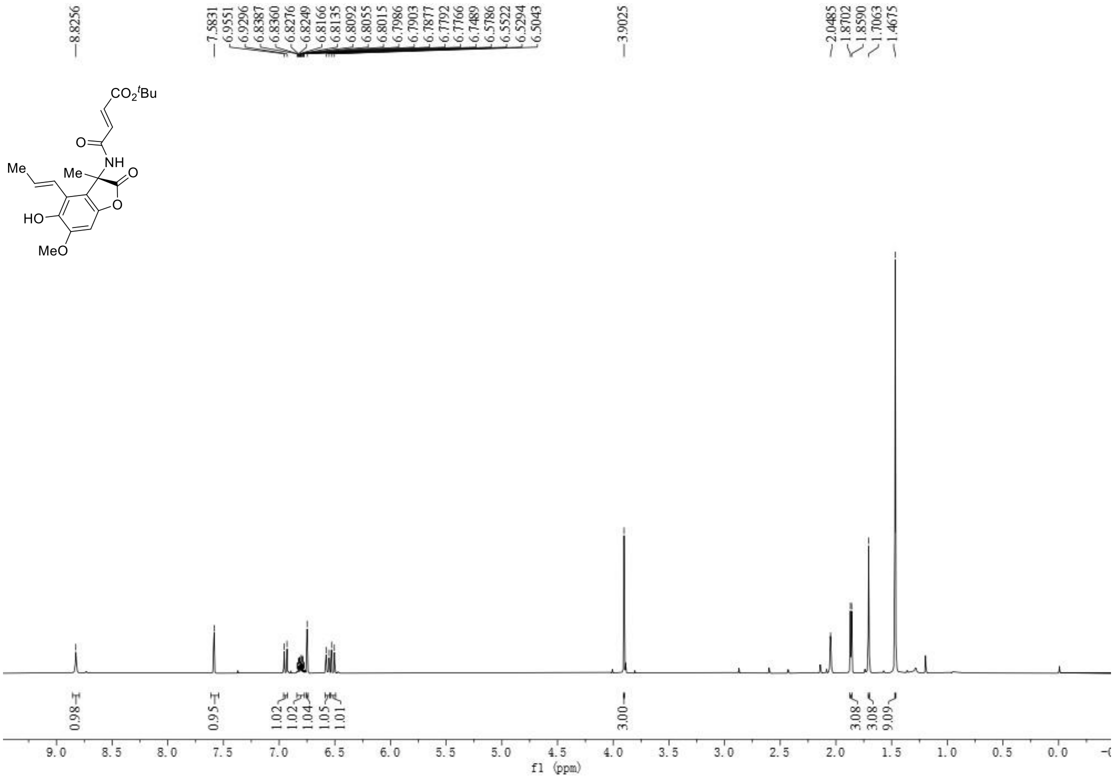

^{13}C NMR of 11

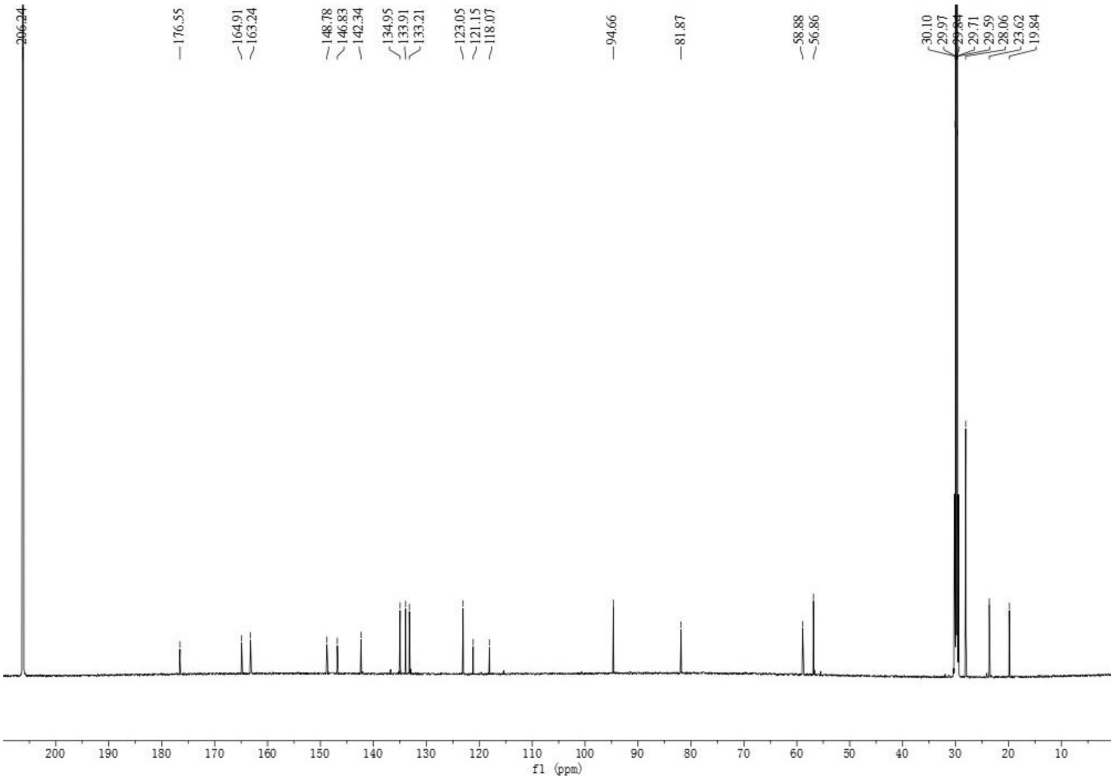

Supplementary Figure 53. ^{1}H NMR, and ^{13}C NMR spectra of compound **11**

^{1}H NMR of (−)-Fumimycin

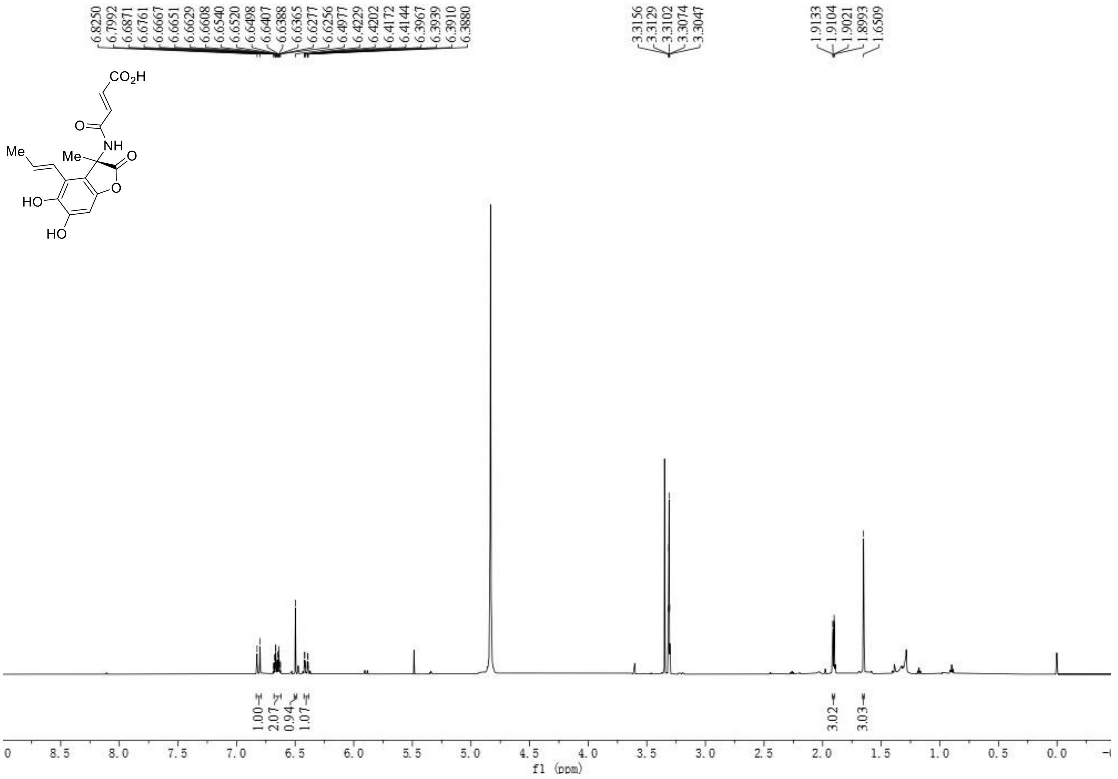

^{13}C NMR of (−)-Fumimycin

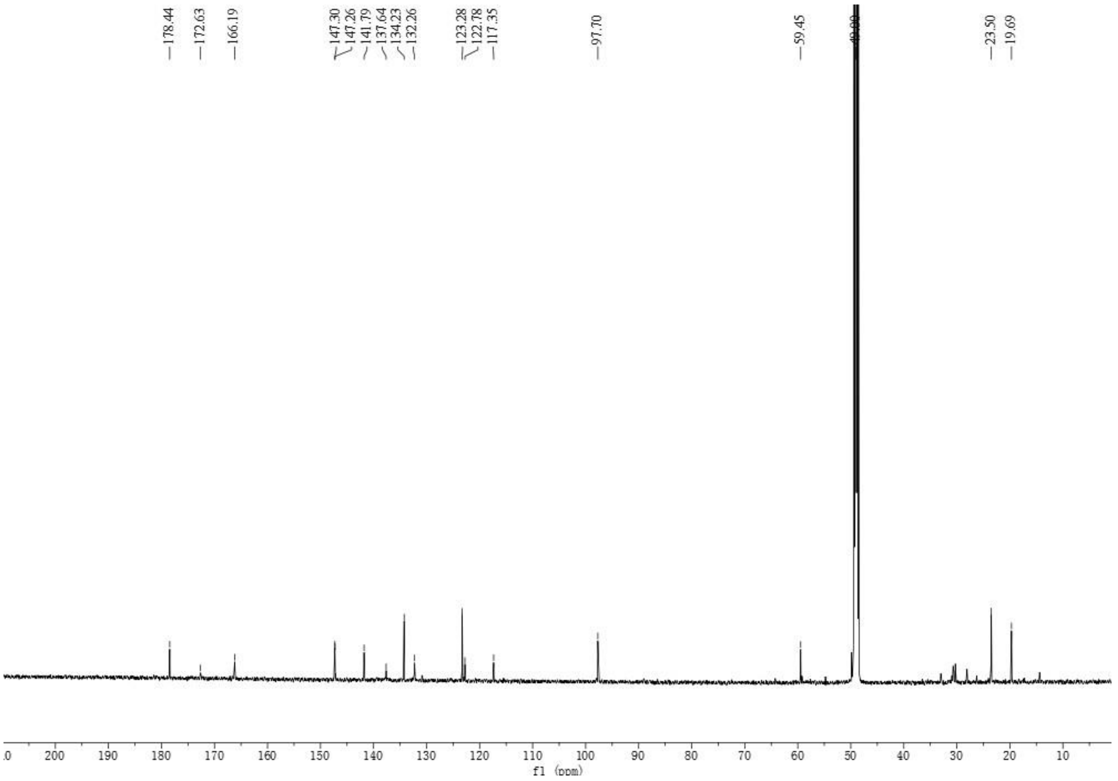

Supplementary Figure 54. ^{1}H NMR, and ^{13}C NMR spectra of compound (−)-**Fumimycin**

^{1}H NMR of 12

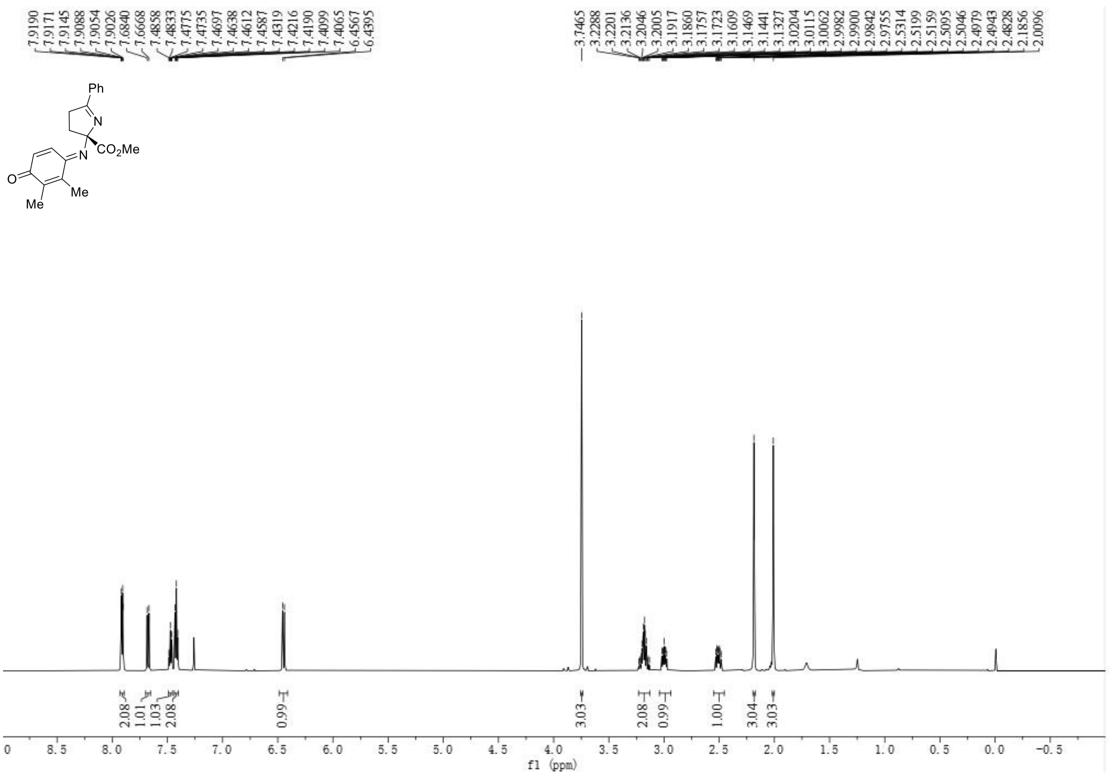

^{13}C NMR of 12

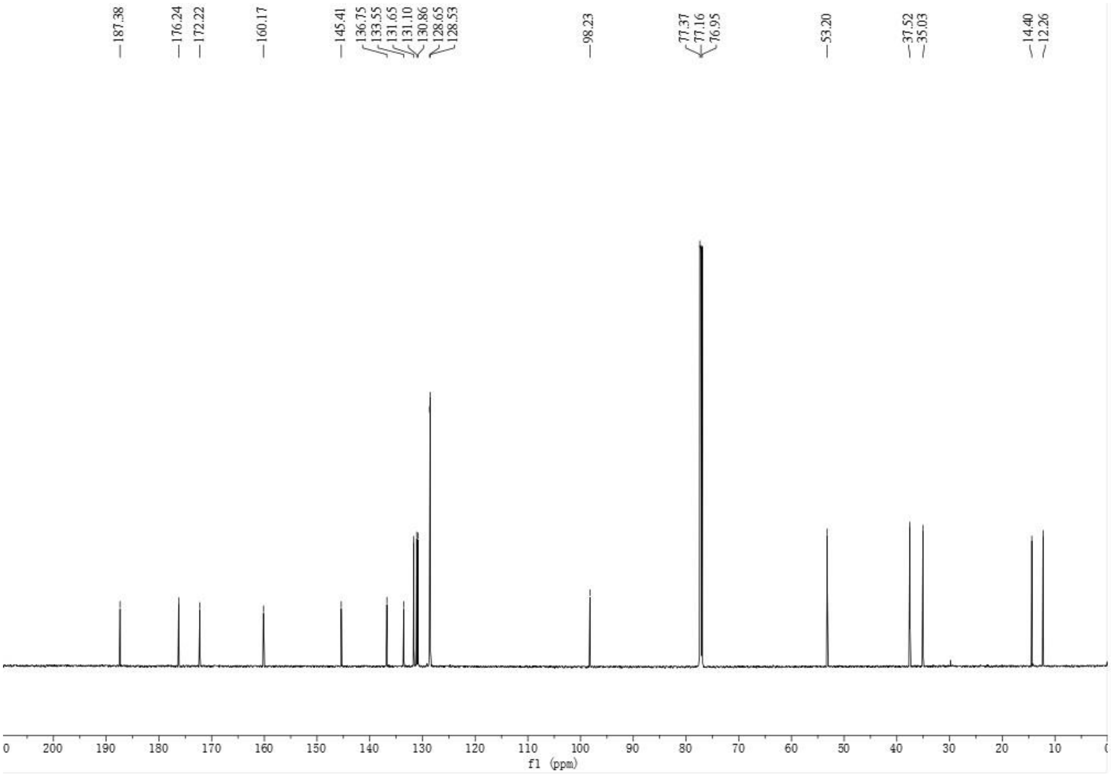

Supplementary Figure 55. ^{1}H NMR, and ^{13}C NMR spectra of compound **12**

3.2 HPLC traces

Rac-**3a**

SAMPLE INFORMATION

Sample Name:	xt-2-80-1-rac-20%-AD	Acquired By:	System
Sample Type:	Unknown	Sample Set Name	0731
Vial:	70	Acq. Method Set:	20% quanbo
Injection #:	1	Processing Method	XT 2 80 1 RAC
Injection Volume:	10.00 ul	Channel Name:	254.0nm
Run Time:	15.0 Minutes	Proc. Chnl. Descr.:	2998 PDA 254.0 nm (2998
Date Acquired:	7/31/2021 9:07:00 PM CST		
Date Processed:	7/31/2021 9:43:57 PM CST		

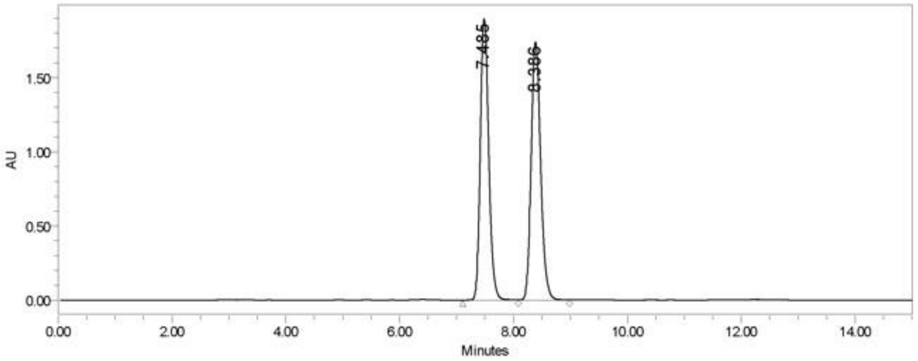

	RT	Area	% Area	Height
1	7.485	19523556	49.17	1894280
2	8.386	20183065	50.83	1740736

Asy-**3a** (DyKAT)

SAMPLE INFORMATION

Sample Name:	xt-2-80-3-20%-AD	Acquired By:	System
Sample Type:	Unknown	Sample Set Name	0731
Vial:	71	Acq. Method Set:	20% quanbo
Injection #:	1	Processing Method	XT 2 80 3
Injection Volume:	10.00 ul	Channel Name:	254.0nm
Run Time:	15.0 Minutes	Proc. Chnl. Descr.:	2998 PDA 254.0 nm (2998
Date Acquired:	7/31/2021 9:22:41 PM CST		
Date Processed:	7/31/2021 9:41:10 PM CST		

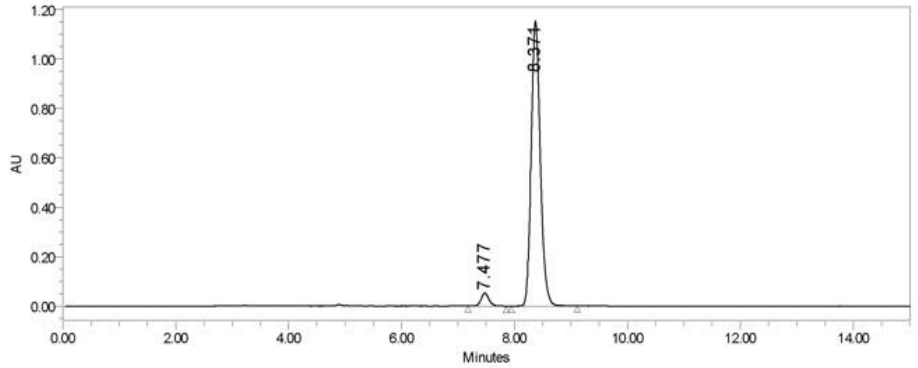

	RT	Area	% Area	Height
1	7.477	508138	3.79	52035
2	8.371	12914679	96.21	1152832

Asy-**3a** (KR)

SAMPLE INFORMATION

Sample Name:	xt-2-82-p-20%-AD	Acquired By:	System
Sample Type:	Unknown	Sample Set Name	0802
Vial:	119	Acq. Method Set:	20% quanbo
Injection #:	1	Processing Method	XT 2 82 P
Injection Volume:	10.00 ul	Channel Name:	254.0nm
Run Time:	15.0 Minutes	Proc. Chnl. Descr.:	2998 PDA 254.0 nm (2998
Date Acquired:	8/2/2021 8:07:04 PM CST		
Date Processed:	8/2/2021 8:24:34 PM CST		

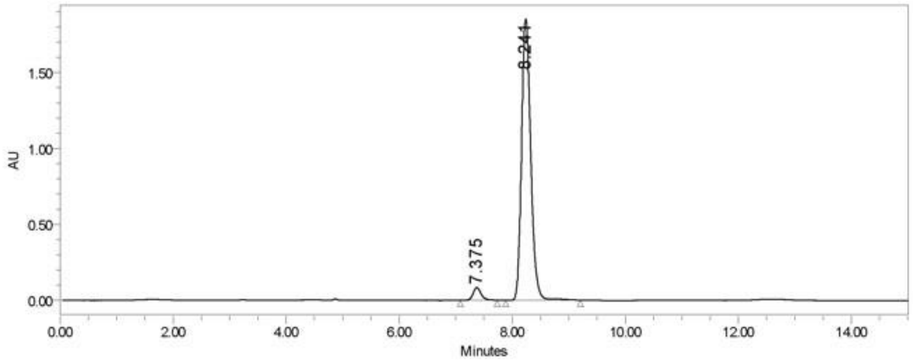

	RT	Area	% Area	Height
1	7.375	820077	3.81	83742
2	8.241	20685313	96.19	1850116

Supplementary Figure 56. HPLC spectra of compound **3a**

Rac-**3b**

SAMPLE INFORMATION

Sample Name:	xt-2-149-RAC-20%-AD	Acquired By:	System
Sample Type:	Unknown	Sample Set Name:	
Vial:	32	Acq. Method Set:	20%qb
Injection #:	1	Processing Method:	XT 2 149 RAC
Injection Volume:	10.00 ul	Channel Name:	254.0nm
Run Time:	12.0 Minutes	Proc. Chnl. Descr.:	2998 PDA 254.0 nm (2998
Date Acquired:	10/13/2021 6:22:14 PM CST		
Date Processed:	10/13/2021 6:36:31 PM CST		

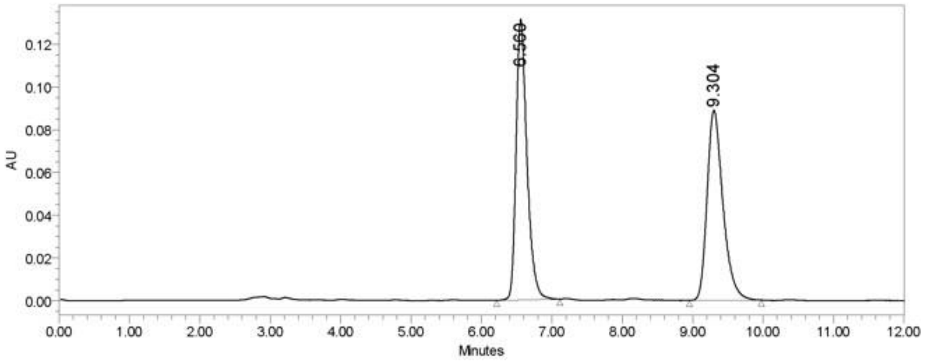

	RT	Area	% Area	Height
1	6.560	1410055	50.26	131259
2	9.304	1395491	49.74	88853

Asy-**3b** (DyKAT)

SAMPLE INFORMATION

Sample Name:	xt-2-150-2-20%-AD	Acquired By:	System
Sample Type:	Unknown	Sample Set Name:	1013
Vial:	67	Acq. Method Set:	20%qb
Injection #:	1	Processing Method:	XT 2 150 2
Injection Volume:	10.00 ul	Channel Name:	254.0nm
Run Time:	12.0 Minutes	Proc. Chnl. Descr.:	2998 PDA 254.0 nm (2998
Date Acquired:	10/13/2021 5:27:18 PM CST		
Date Processed:	10/13/2021 6:31:19 PM CST		

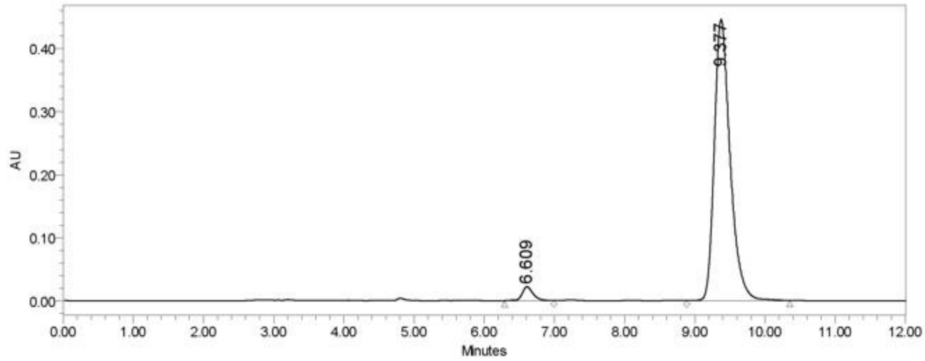

	RT	Area	% Area	Height
1	6.609	240034	3.32	22034
2	9.377	6992894	96.68	445932

Supplementary Figure 57. HPLC spectra of compound **3b**

Rac-**3c**

SAMPLE INFORMATION

Sample Name:	xt-2-143-rac-20%-AD	Acquired By:	System
Sample Type:	Unknown	Sample Set Name:	10132
Vial:	75	Acq. Method Set:	20%qb
Injection #:	1	Processing Method:	XT 2 143 RAC
Injection Volume:	10.00 ul	Channel Name:	254.0nm
Run Time:	12.5 Minutes	Proc. Chnl. Descr.:	2998 PDA 254.0 nm (2998
Date Acquired:	10/13/2021 8:39:26 PM CST		
Date Processed:	10/13/2021 9:03:49 PM CST		

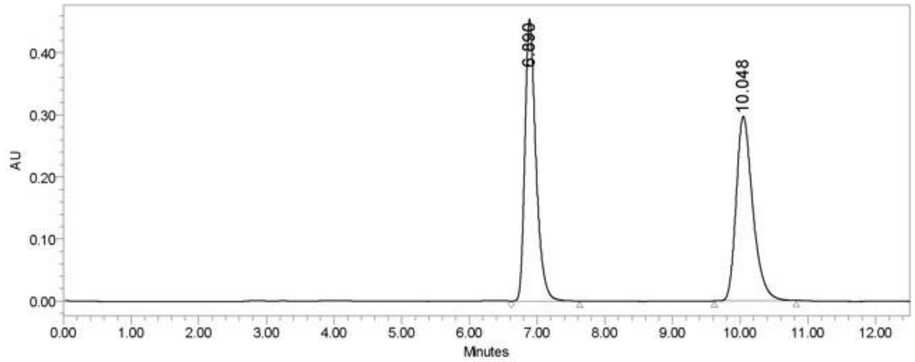

	RT	Area	% Area	Height
1	6.890	5133250	50.38	454441
2	10.048	5056183	49.62	296929

Asy-**3c** (DyKAT)

SAMPLE INFORMATION

Sample Name:	xt-2-147-1-20%-AD	Acquired By:	System
Sample Type:	Unknown	Sample Set Name:	10132
Vial:	74	Acq. Method Set:	20%qb
Injection #:	1	Processing Method:	xt 2 147 1
Injection Volume:	10.00 ul	Channel Name:	254.0nm
Run Time:	12.5 Minutes	Proc. Chnl. Descr.:	2998 PDA 254.0 nm (2998
Date Acquired:	10/13/2021 8:20:53 PM CST		
Date Processed:	10/13/2021 9:01:37 PM CST		

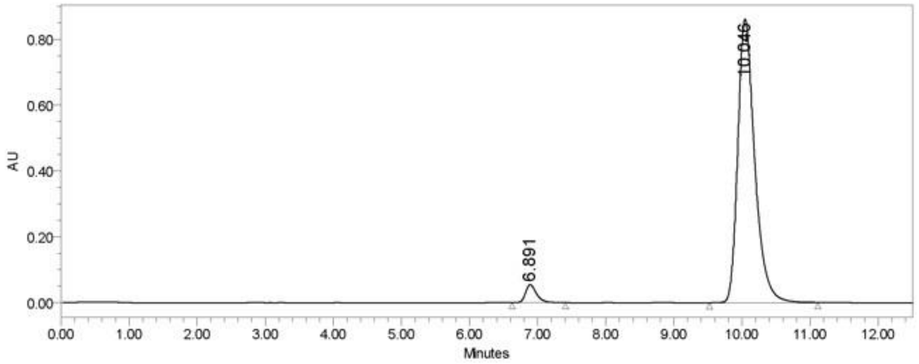

	RT	Area	% Area	Height
1	6.891	618696	4.00	54685
2	10.046	14830302	96.00	860879

Asy-**3c** (KR)

SAMPLE INFORMATION

Sample Name:	xt-2-147-3-20%-AD	Acquired By:	System
Sample Type:	Unknown	Sample Set Name:	
Vial:	54	Acq. Method Set:	20%qb
Injection #:	1	Processing Method:	XT 2 147 3
Injection Volume:	10.00 ul	Channel Name:	254.0nm
Run Time:	12.5 Minutes	Proc. Chnl. Descr.:	2998 PDA 254.0 nm (2998
Date Acquired:	10/13/2021 7:44:21 PM CST		
Date Processed:	10/13/2021 9:33:02 PM CST		

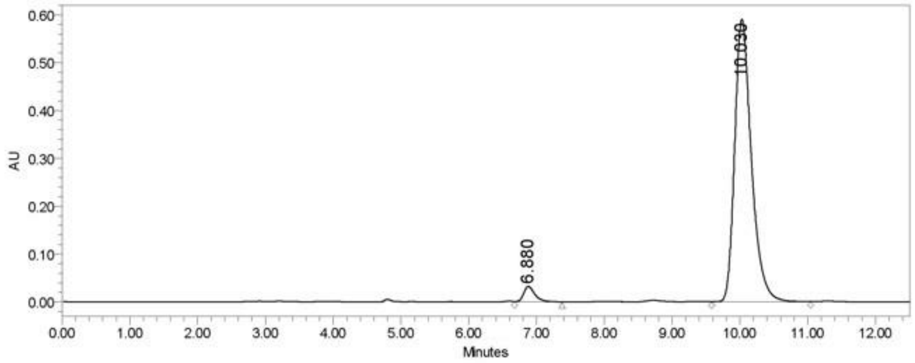

	RT	Area	% Area	Height
1	6.880	371434	3.54	32261
2	10.030	10108783	96.46	590286

Supplementary Figure 58. HPLC spectra of compound **3c**

Rac-**3d**

SAMPLE INFORMATION

Sample Name:	xt-2-116-rac-20%AD	Acquired By:	System
Sample Type:	Unknown	Sample Set Name:	xt10113
Vial:	80	Acq. Method Set:	20%qb
Injection #:	1	Processing Method:	xt 2 116 rac
Injection Volume:	10.00 ul	Channel Name:	254.0nm
Run Time:	13.0 Minutes	Proc. Chnl. Descr.:	2998 PDA 254.0 nm (2998
Date Acquired:	10/11/2021 10:15:33 PM CST		
Date Processed:	10/12/2021 5:09:38 PM CST		

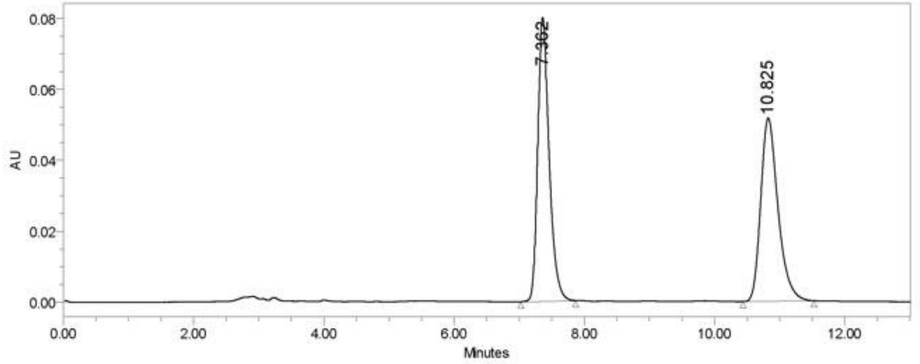

	RT	Area	% Area	Height
1	7.362	967798	50.28	79984
2	10.825	957002	49.72	51741

Asy-**3d** (DyKAT)

SAMPLE INFORMATION

Sample Name:	xt-2-117-4-20%-AD	Acquired By:	System
Sample Type:	Unknown	Sample Set Name:	
Vial:	35	Acq. Method Set:	20%qb
Injection #:	1	Processing Method:	xt 2 117 4
Injection Volume:	10.00 ul	Channel Name:	254.0nm
Run Time:	13.0 Minutes	Proc. Chnl. Descr.:	2998 PDA 254.0 nm (2998
Date Acquired:	10/13/2021 4:45:48 PM CST		
Date Processed:	10/13/2021 5:00:24 PM CST		

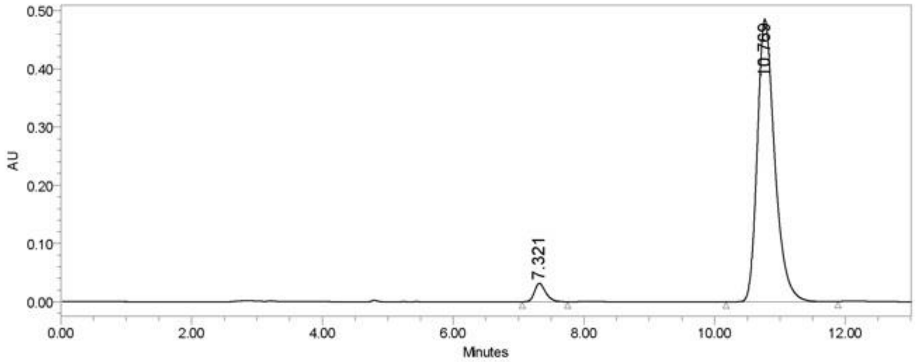

	RT	Area	% Area	Height
1	7.321	384416	4.09	31752
2	10.769	9007648	95.91	484970

Asy-**3d** (KR)

SAMPLE INFORMATION

Sample Name:	xt-2-117-3-20%AD	Acquired By:	System
Sample Type:	Unknown	Sample Set Name:	xt10113
Vial:	79	Acq. Method Set:	20%qb
Injection #:	1	Processing Method:	xt 2 117 3
Injection Volume:	10.00 ul	Channel Name:	254.0nm
Run Time:	13.0 Minutes	Proc. Chnl. Descr.:	2998 PDA 254.0 nm (2998
Date Acquired:	10/11/2021 9:56:45 PM CST		
Date Processed:	10/12/2021 5:08:08 PM CST		

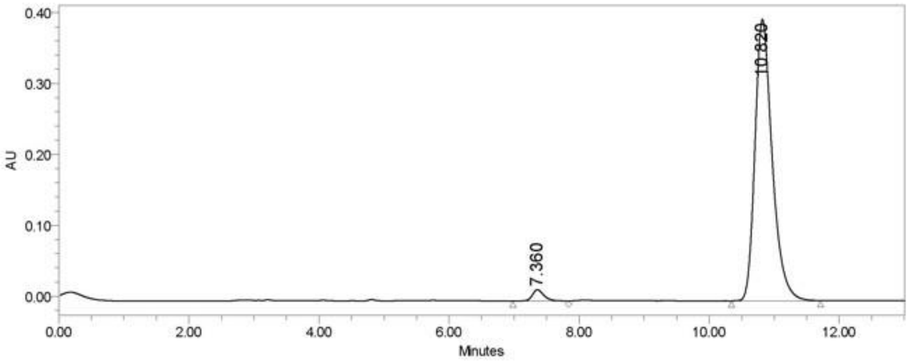

	RT	Area	% Area	Height
1	7.360	193280	2.56	15837
2	10.820	7343080	97.44	396731

Supplementary Figure 59. HPLC spectra of compound **3d**

Rac-**3e**

SAMPLE INFORMATION

Sample Name:	xt-2-108-rac-20%-AD	Acquired By:	System
Sample Type:	Unknown	Sample Set Name:	0831
Vial:	101	Acq. Method Set:	20%qb
Injection #:	1	Processing Method:	xt 2 108 rac
Injection Volume:	10.00 ul	Channel Name:	254.0nm
Run Time:	12.0 Minutes	Proc. Chnl. Descr.:	2998 PDA 254.0 nm (2998
Date Acquired:	8/30/2021 11:07:14 PM CST		
Date Processed:	8/31/2021 8:43:21 AM CST		

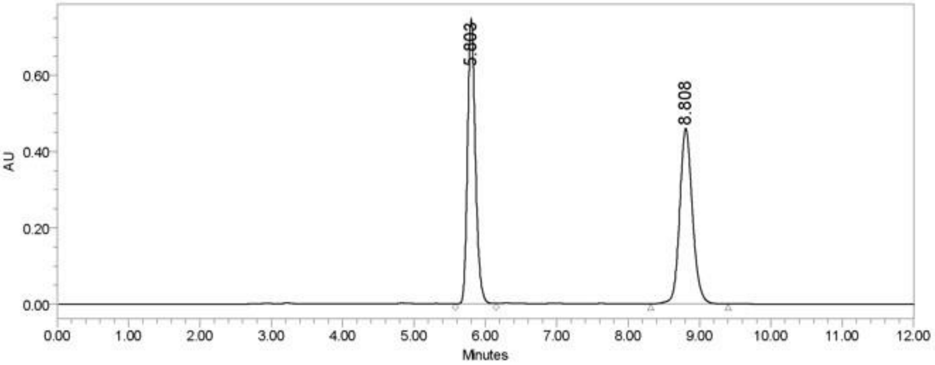

	RT	Area	% Area	Height
1	5.803	5669631	49.84	748228
2	8.808	5705109	50.16	460126

Asy-**3e** (DyKAT)

SAMPLE INFORMATION

Sample Name:	xt-2-109-1-20%-AD	Acquired By:	System
Sample Type:	Unknown	Sample Set Name:	0831
Vial:	102	Acq. Method Set:	20%qb
Injection #:	1	Processing Method:	xt 2 109 1
Injection Volume:	10.00 ul	Channel Name:	254.0nm
Run Time:	12.0 Minutes	Proc. Chnl. Descr.:	2998 PDA 254.0 nm (2998
Date Acquired:	8/30/2021 11:25:06 PM CST		
Date Processed:	8/31/2021 8:45:37 AM CST		

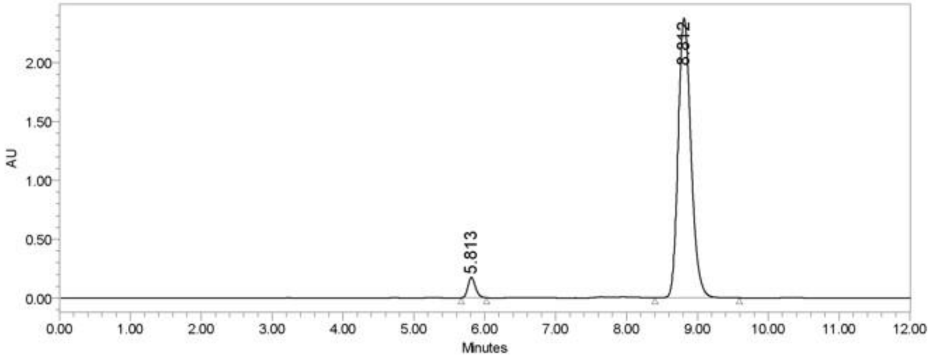

	RT	Area	% Area	Height
1	5.813	1287230	4.13	173133
2	8.812	29845166	95.87	2374603

Asy-**3e** (KR)

SAMPLE INFORMATION

Sample Name:	xt-2-109-4-20%-AD	Acquired By:	System
Sample Type:	Unknown	Sample Set Name:	08312
Vial:	103	Acq. Method Set:	20%qb
Injection #:	1	Processing Method:	xt 2 109 4
Injection Volume:	4.00 ul	Channel Name:	254.0nm
Run Time:	12.0 Minutes	Proc. Chnl. Descr.:	2998 PDA 254.0 nm (2998
Date Acquired:	8/31/2021 8:54:31 AM CST		
Date Processed:	8/31/2021 9:20:53 AM CST		

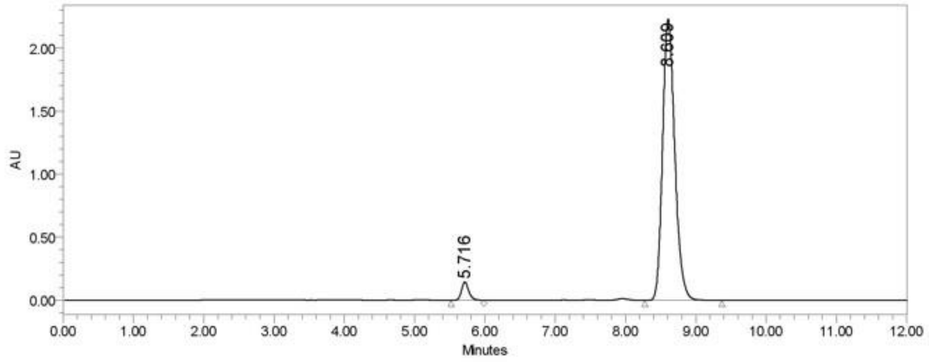

	RT	Area	% Area	Height
1	5.716	1029203	3.69	143929
2	8.609	26878910	96.31	2230543

Supplementary Figure 60. HPLC spectra of compound **3e**

Rac-**3f**

SAMPLE INFORMATION

Sample Name:	xt-2-151-rac-20%-AD	Acquired By:	System
Sample Type:	Unknown	Sample Set Name:	1021
Vial:	82	Acq. Method Set:	20%qb
Injection #:	1	Processing Method:	xt 2 151 rac
Injection Volume:	10.00 ul	Channel Name:	254.0nm
Run Time:	9.5 Minutes	Proc. Chnl. Descr.:	2998 PDA 254.0 nm (2998
Date Acquired:	10/21/2021 10:07:04 PM CST		
Date Processed:	10/21/2021 10:35:17 PM CST		

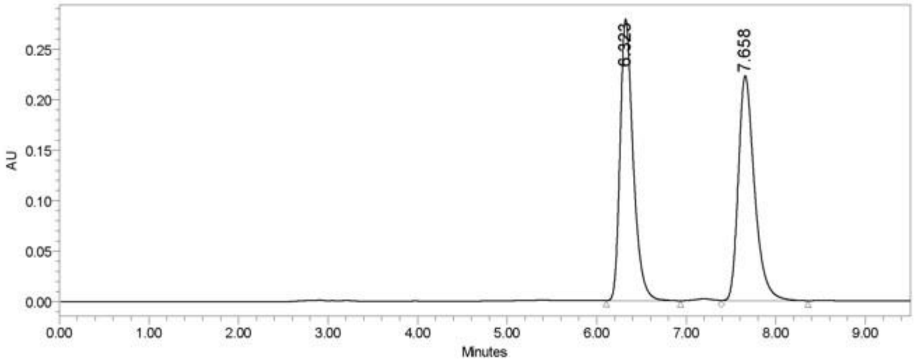

	RT	Area	% Area	Height
1	6.323	2836478	50.18	279083
2	7.658	2816358	49.82	223061

Asy-**3f** (DyKAT)

SAMPLE INFORMATION

Sample Name:	xt-2-154-1-20%-AD	Acquired By:	System
Sample Type:	Unknown	Sample Set Name:	1021
Vial:	83	Acq. Method Set:	20%qb
Injection #:	1	Processing Method:	xt 2 154 1
Injection Volume:	10.00 ul	Channel Name:	254.0nm
Run Time:	9.5 Minutes	Proc. Chnl. Descr.:	2998 PDA 254.0 nm (2998
Date Acquired:	10/21/2021 10:23:39 PM CST		
Date Processed:	10/21/2021 10:36:55 PM CST		

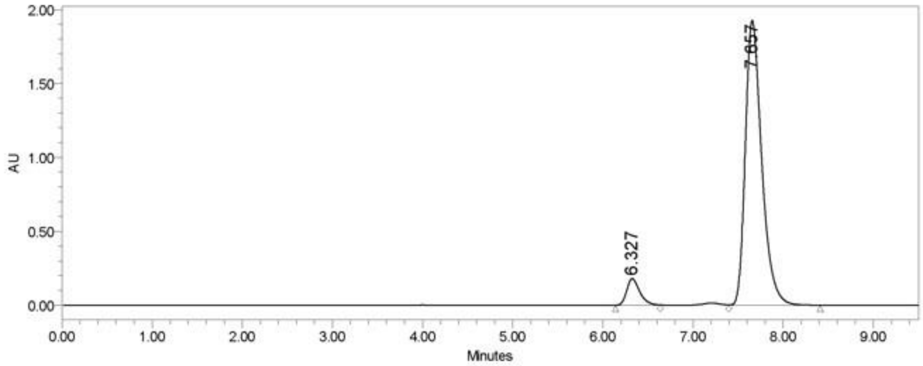

	RT	Area	% Area	Height
1	6.327	1812606	6.90	180309
2	7.657	24448450	93.10	1925920

Supplementary Figure 61. HPLC spectra of compound **3f**

Rac-**3g**

SAMPLE INFORMATION

Sample Name:	xt-2-112-RAC-20%-AD	Acquired By:	System
Sample Type:	Unknown	Sample Set Name:	09012
Vial:	43	Acq. Method Set:	20%qb
Injection #:	1	Processing Method:	XT 2 112 RAC
Injection Volume:	10.00 ul	Channel Name:	254.0nm
Run Time:	16.0 Minutes	Proc. Chnl. Descr.:	2998 PDA 254.0 nm (2998
Date Acquired:	9/1/2021 2:17:46 PM CST		
Date Processed:	9/1/2021 3:03:15 PM CST		

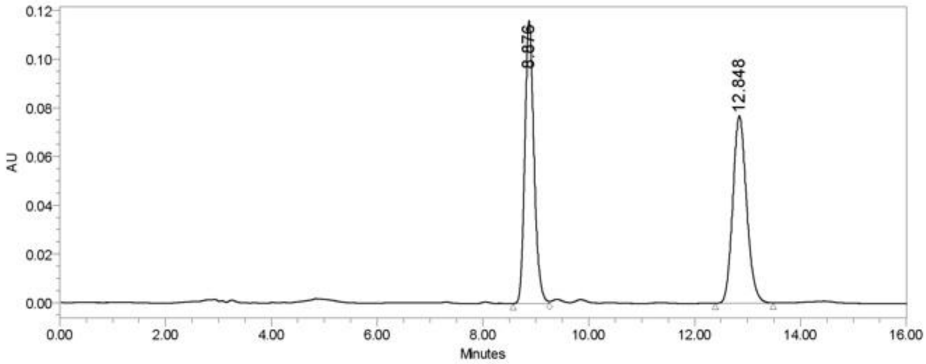

	RT	Area	% Area	Height
1	8.876	1394583	50.02	115997
2	12.848	1393522	49.98	76878

Asy-**3g** (DyKAT)

SAMPLE INFORMATION

Sample Name:	xt-2-113-3-20%-AD	Acquired By:	System
Sample Type:	Unknown	Sample Set Name:	09013
Vial:	72	Acq. Method Set:	20%qb
Injection #:	1	Processing Method:	XT 2 113 3
Injection Volume:	10.00 ul	Channel Name:	254.0nm
Run Time:	16.0 Minutes	Proc. Chnl. Descr.:	2998 PDA 254.0 nm (2998
Date Acquired:	9/1/2021 2:42:19 PM CST		
Date Processed:	9/1/2021 3:00:39 PM CST		

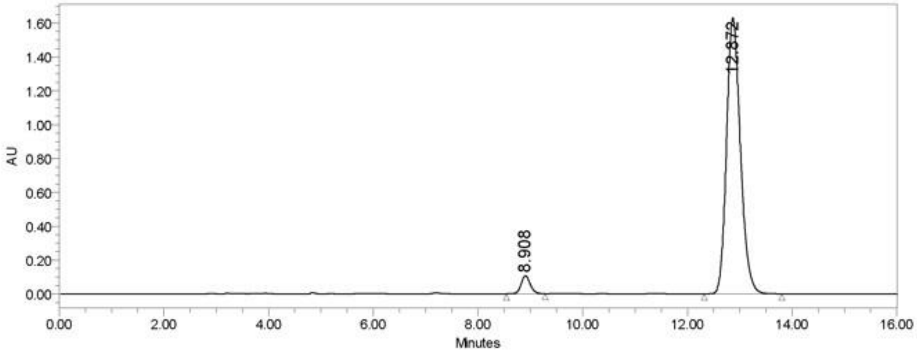

	RT	Area	% Area	Height
1	8.908	1299589	4.17	106995
2	12.872	29880473	95.83	1629167

Asy-**3g** (KR)

SAMPLE INFORMATION

Sample Name:	xt-2-113-1-20%-AD	Acquired By:	System
Sample Type:	Unknown	Sample Set Name:	09012
Vial:	42	Acq. Method Set:	20%qb
Injection #:	1	Processing Method:	XT 2 113 1
Injection Volume:	10.00 ul	Channel Name:	254.0nm
Run Time:	16.0 Minutes	Proc. Chnl. Descr.:	2998 PDA 254.0 nm (2998
Date Acquired:	9/1/2021 1:56:52 PM CST		
Date Processed:	9/1/2021 3:01:53 PM CST		

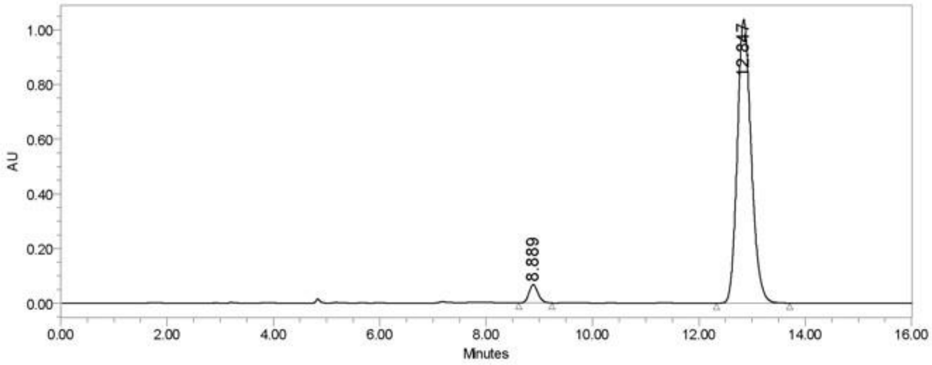

	RT	Area	% Area	Height
1	8.889	797483	4.05	66823
2	12.847	18884990	95.95	1037535

Supplementary Figure 62. HPLC spectra of compound **3g**

Rac-**3h**

SAMPLE INFORMATION

Sample Name:	xt-2-74-rac-20%-AD	Acquired By:	System
Sample Type:	Unknown	Sample Set Name:	
Vial:	50	Acq. Method Set:	20%qb
Injection #:	1	Processing Method:	XT 2 74 RAC
Injection Volume:	10.00 ul	Channel Name:	254.0nm
Run Time:	60.0 Minutes	Proc. Chnl. Descr.:	2998 PDA 254.0 nm (2998
Date Acquired:	1/21/2022 3:00:36 PM CST		
Date Processed:	1/21/2022 5:06:22 PM CST		

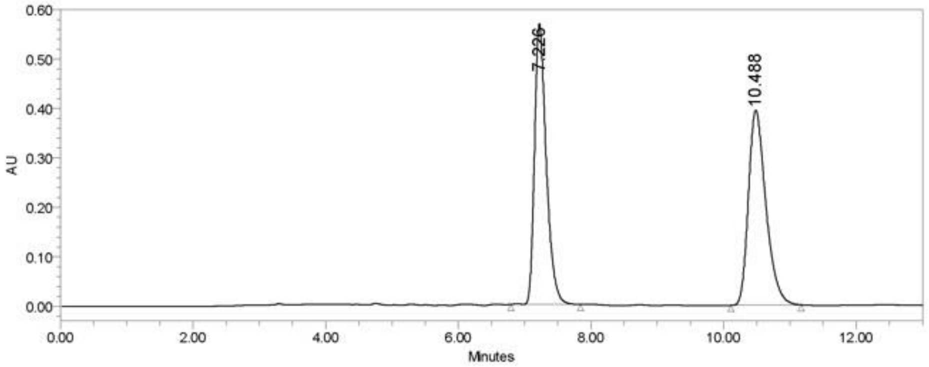

	RT	Area	% Area	Height
1	7.226	6946550	49.07	567927
2	10.488	7208479	50.93	394266

Asy-**3h** (DyKAT)

SAMPLE INFORMATION

Sample Name:	xt-3-75-1-20%-AD	Acquired By:	System
Sample Type:	Unknown	Sample Set Name:	0122
Vial:	5	Acq. Method Set:	20%qb
Injection #:	1	Processing Method:	xt 3 75 1
Injection Volume:	10.00 ul	Channel Name:	254.0nm
Run Time:	15.0 Minutes	Proc. Chnl. Descr.:	2998 PDA 254.0 nm (2998
Date Acquired:	1/22/2022 11:07:52 AM CST		
Date Processed:	1/22/2022 11:35:47 AM CST		

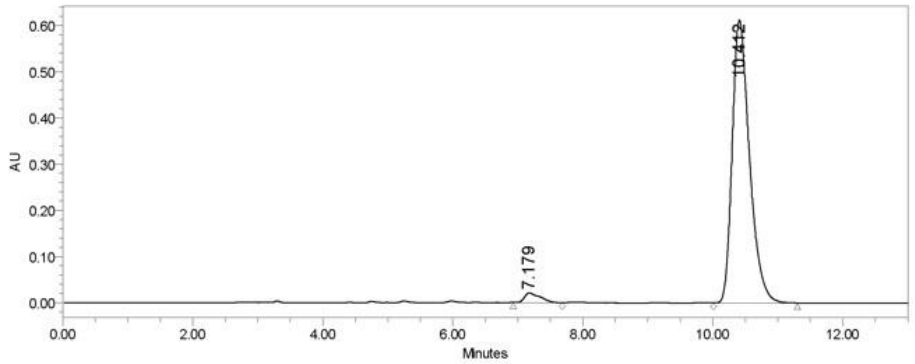

	RT	Area	% Area	Height
1	7.179	342664	2.94	20737
2	10.412	11318977	97.06	612113

Asy-**3h** (KR)

SAMPLE INFORMATION

Sample Name:	XT-3-75-2-20%-AD	Acquired By:	System
Sample Type:	Unknown	Sample Set Name:	0121
Vial:	85	Acq. Method Set:	20%qb
Injection #:	1	Processing Method:	XT 3 74 2
Injection Volume:	10.00 ul	Channel Name:	254.0nm
Run Time:	15.0 Minutes	Proc. Chnl. Descr.:	2998 PDA 254.0 nm (2998
Date Acquired:	1/21/2022 3:38:51 PM CST		
Date Processed:	1/21/2022 5:08:11 PM CST		

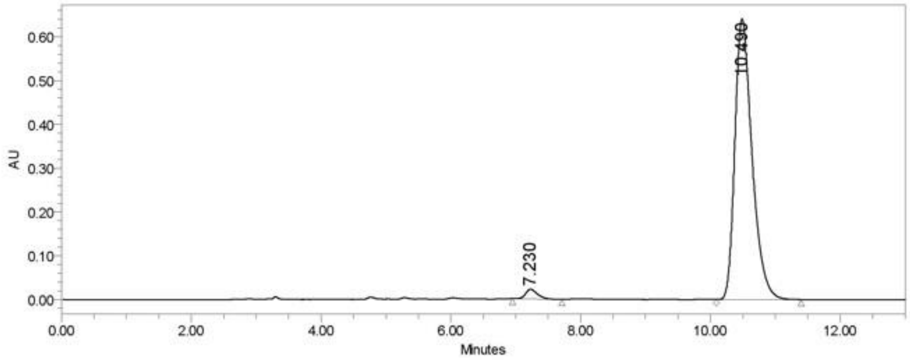

	RT	Area	% Area	Height
1	7.230	273683	2.27	21884
2	10.490	11780593	97.73	641077

Supplementary Figure 63. HPLC spectra of compound **3h**

Rac-**3i**

SAMPLE INFORMATION

Sample Name:	xt-2-71-RAC-20%-AD	Acquired By:	System
Sample Type:	Unknown	Sample Set Name:	
Vial:	4	Acq. Method Set:	20%qb
Injection #:	1	Processing Method:	XT 3 71 RAC
Injection Volume:	10.00 ul	Channel Name:	254.0nm
Run Time:	100.0 Minutes	Proc. Chnl. Descr.:	2998 PDA 254.0 nm (2998
Date Acquired:	1/18/2022 10:12:16 PM CST		
Date Processed:	1/18/2022 10:25:04 PM CST		

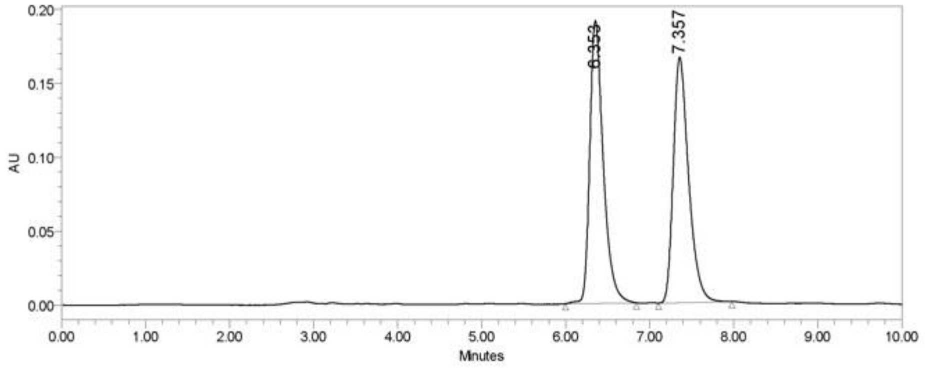

	RT	Area	% Area	Height
1	6.353	2118644	50.24	191398
2	7.357	2098140	49.76	166017

Asy-**3i** (DyKAT)

SAMPLE INFORMATION

Sample Name:	xt-2-72-3-20%-AD	Acquired By:	System
Sample Type:	Unknown	Sample Set Name:	
Vial:	106	Acq. Method Set:	20%qb
Injection #:	1	Processing Method:	XT 3 72 3
Injection Volume:	10.00 ul	Channel Name:	254.0nm
Run Time:	100.0 Minutes	Proc. Chnl. Descr.:	2998 PDA 254.0 nm (2998
Date Acquired:	1/18/2022 9:55:01 PM CST		
Date Processed:	1/18/2022 10:26:50 PM CST		

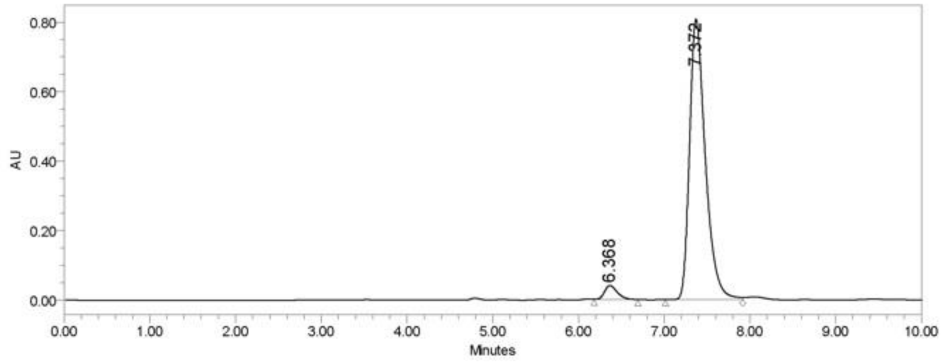

	RT	Area	% Area	Height
1	6.368	401223	3.74	39594
2	7.372	10321746	96.26	807319

Supplementary Figure 64. HPLC spectra of compound **3i**

Rac-**3j**

SAMPLE INFORMATION

Sample Name:	xt-2-110-rac-20%-AD	Acquired By:	System
Sample Type:	Unknown	Sample Set Name:	08312
Vial:	116	Acq. Method Set:	20%qb
Injection #:	1	Processing Method:	xt 2 110 rac
Injection Volume:	10.00 ul	Channel Name:	254.0nm
Run Time:	18.0 Minutes	Proc. Chnl. Descr.:	2998 PDA 254.0 nm (2998
Date Acquired:	8/31/2021 5:44:06 PM CST		
Date Processed:	8/31/2021 6:17:22 PM CST		

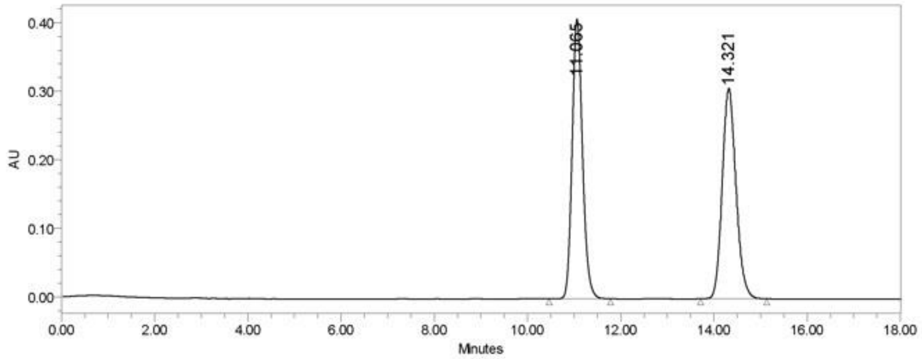

	RT	Area	% Area	Height
1	11.065	6334056	50.06	407969
2	14.321	6319222	49.94	306956

Asy-**3j** (DyKAT)

SAMPLE INFORMATION

Sample Name:	xt-2-111-3-20%-AD	Acquired By:	System
Sample Type:	Unknown	Sample Set Name:	08312
Vial:	118	Acq. Method Set:	20%qb
Injection #:	1	Processing Method:	xt 2 111 3
Injection Volume:	10.00 ul	Channel Name:	254.0nm
Run Time:	18.0 Minutes	Proc. Chnl. Descr.:	2998 PDA 254.0 nm (2998
Date Acquired:	8/31/2021 6:27:50 PM CST		
Date Processed:	8/31/2021 6:46:33 PM CST		

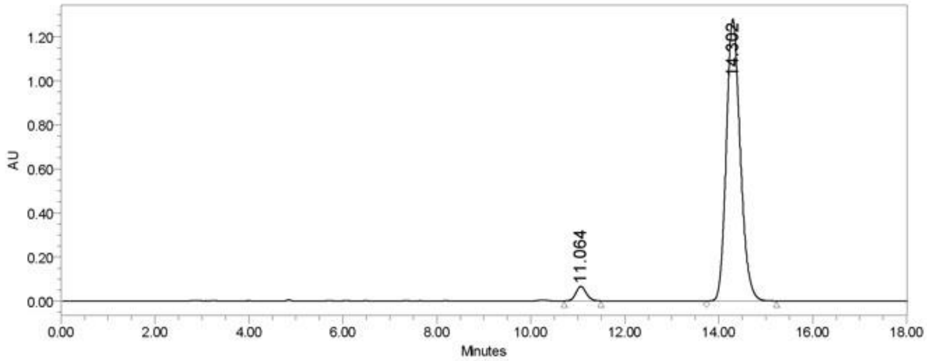

	RT	Area	% Area	Height
1	11.064	1008595	3.67	65683
2	14.302	26456111	96.33	1279745

Asy-**3j** (KR)

SAMPLE INFORMATION

Sample Name:	xt-2-111-1-20%-AD	Acquired By:	System
Sample Type:	Unknown	Sample Set Name:	08312
Vial:	117	Acq. Method Set:	20%qb
Injection #:	1	Processing Method:	xt 2 111 1
Injection Volume:	10.00 ul	Channel Name:	254.0nm
Run Time:	18.0 Minutes	Proc. Chnl. Descr.:	2998 PDA 254.0 nm (2998
Date Acquired:	8/31/2021 6:05:59 PM CST		
Date Processed:	8/31/2021 6:41:38 PM CST		

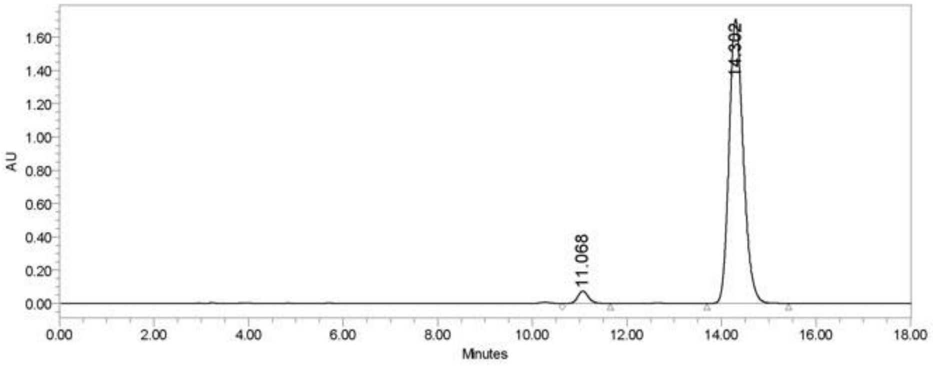

	RT	Area	% Area	Height
1	11.068	1148189	3.13	74006
2	14.302	35513291	96.87	1706581

Supplementary Figure 65. HPLC spectra of compound **3j**

Rac-**3k**

SAMPLE INFORMATION

Sample Name:	xt-2-101-RAC-20%-AD	Acquired By:	System
Sample Type:	Unknown	Sample Set Name	0825
Vial:	72	Acq. Method Set:	20% quanbo
Injection #:	1	Processing Method	XT 2 101 1 RAC
Injection Volume:	10.00 ul	Channel Name:	254.0nm
Run Time:	15.0 Minutes	Proc. Chnl. Descr.:	2998 PDA 254.0 nm (2998
Date Acquired:	8/25/2021 3:21:14 PM CST		
Date Processed:	10/23/2021 9:42:57 PM CST		

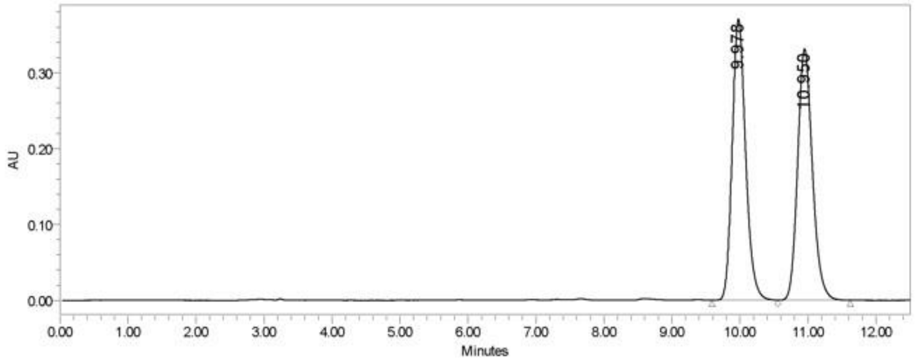

	RT	Area	% Area	Height
1	9.978	5063455	50.12	370316
2	10.950	5040192	49.88	331178

Asy-**3k** (DyKAT)

SAMPLE INFORMATION

Sample Name:	xt-2-106-1-20%-AD	Acquired By:	System
Sample Type:	Unknown	Sample Set Name	0825
Vial:	70	Acq. Method Set:	20% quanbo
Injection #:	1	Processing Method	xt 2 106 1
Injection Volume:	10.00 ul	Channel Name:	254.0nm
Run Time:	15.0 Minutes	Proc. Chnl. Descr.:	2998 PDA 254.0 nm (2998
Date Acquired:	8/25/2021 2:49:53 PM CST		
Date Processed:	10/23/2021 9:40:20 PM CST		

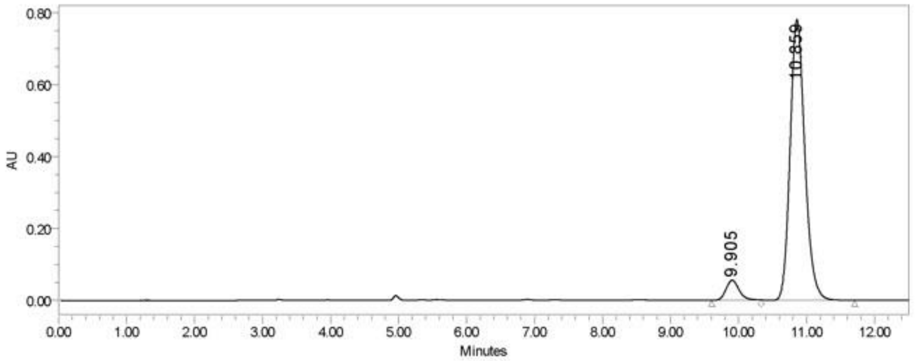

	RT	Area	% Area	Height
1	9.905	769411	6.12	56162
2	10.859	11808224	93.88	780853

Supplementary Figure 66. HPLC spectra of compound **3k**

Rac-**3l**

SAMPLE INFORMATION

Sample Name:	xt-2-137-rac-20%-AD	Acquired By:	System
Sample Type:	Unknown	Sample Set Name:	1022
Vial:	2	Acq. Method Set:	20%qb
Injection #:	1	Processing Method:	XT 2 137 RAC
Injection Volume:	10.00 ul	Channel Name:	254.0nm
Run Time:	11.0 Minutes	Proc. Chnl. Descr.:	2998 PDA 254.0 nm (2998
Date Acquired:	10/22/2021 9:28:11 PM CST		
Date Processed:	10/22/2021 10:02:41 PM CST		

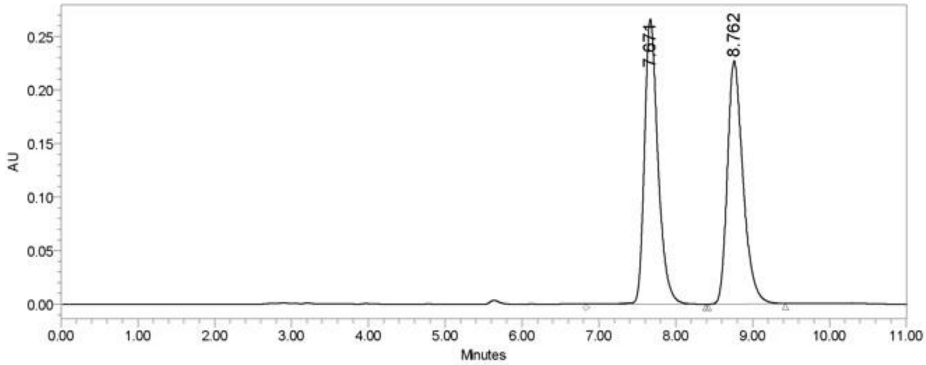

	RT	Area	% Area	Height
1	7.671	3264357	50.37	266248
2	8.762	3216136	49.63	227150

Asy-**3l** (DyKAT)

SAMPLE INFORMATION

Sample Name:	xt-2-139-4-20%-AD	Acquired By:	System
Sample Type:	Unknown	Sample Set Name:	1022
Vial:	3	Acq. Method Set:	20%qb
Injection #:	1	Processing Method:	XT 2 139 4
Injection Volume:	10.00 ul	Channel Name:	254.0nm
Run Time:	11.0 Minutes	Proc. Chnl. Descr.:	2998 PDA 254.0 nm (2998
Date Acquired:	10/22/2021 9:46:03 PM CST		
Date Processed:	10/22/2021 10:01:02 PM CST		

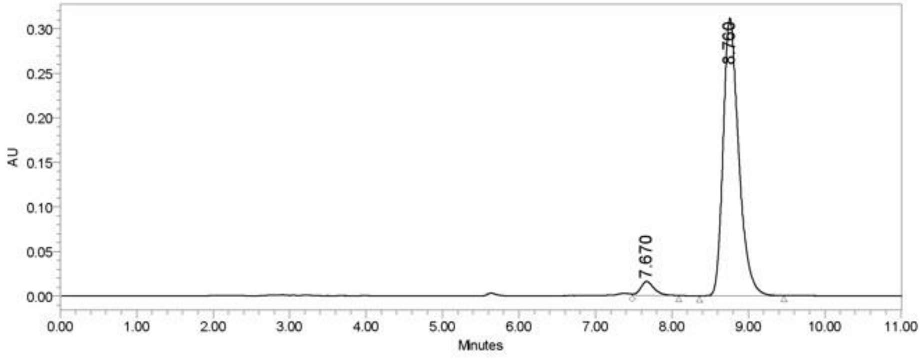

	RT	Area	% Area	Height
1	7.670	195286	4.23	15733
2	8.760	4417414	95.77	311844

Supplementary Figure 67. HPLC spectra of compound **3l**

Rac-**3m**

SAMPLE INFORMATION

Sample Name:	xt-3-164-RAC-10%-IA	Acquired By:	System
Sample Type:	Unknown	Sample Set Name:	
Vial:	68	Acq. Method Set:	10%qb
Injection #:	1	Processing Method:	xt 3 164 rac
Injection Volume:	10.00 ul	Channel Name:	254.0nm
Run Time:	60.0 Minutes	Proc. Chnl. Descr.:	2998 PDA 254.0 nm (2998
Date Acquired:	10/3/2022 8:26:56 PM CST		
Date Processed:	10/4/2022 4:26:49 PM CST		

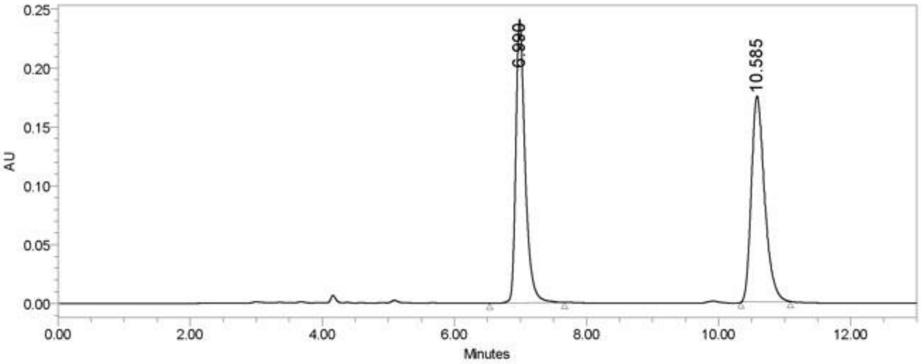

	RT	Area	% Area	Height
1	6.990	2379083	49.38	240906
2	10.585	2438685	50.62	174933

Asy-**3m** (DyKAT)

SAMPLE INFORMATION

Sample Name:	xt-3-164-asy-10%-IA	Acquired By:	System
Sample Type:	Unknown	Sample Set Name:	
Vial:	4	Acq. Method Set:	10%qb
Injection #:	1	Processing Method:	xt 3 164 asy
Injection Volume:	10.00 ul	Channel Name:	254.0nm
Run Time:	60.0 Minutes	Proc. Chnl. Descr.:	2998 PDA 254.0 nm (2998
Date Acquired:	10/4/2022 4:10:09 PM CST		
Date Processed:	10/4/2022 4:25:03 PM CST		

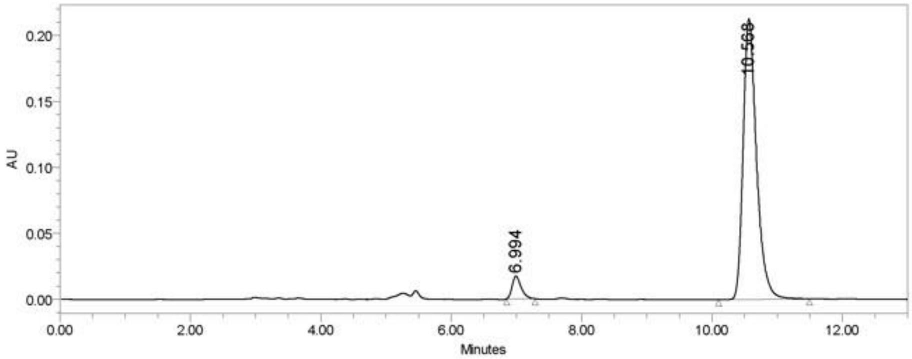

	RT	Area	% Area	Height
1	6.994	165436	5.20	17200
2	10.568	3016111	94.80	212573

Supplementary Figure 68. HPLC spectra of compound **3m**

Rac-**3n**

SAMPLE INFORMATION

Sample Name:	xt-3-78-RAC-20%-AD	Acquired By:	System
Sample Type:	Unknown	Sample Set Name:	
Vial:	24	Acq. Method Set:	20%qb
Injection #:	1	Processing Method:	XT 3 78 RAC
Injection Volume:	10.00 ul	Channel Name:	254.0nm
Run Time:	60.0 Minutes	Proc. Chnl. Descr.:	2998 PDA 254.0 nm (2998
Date Acquired:	1/26/2022 9:55:10 PM CST		
Date Processed:	5/5/2022 9:21:03 PM CST		

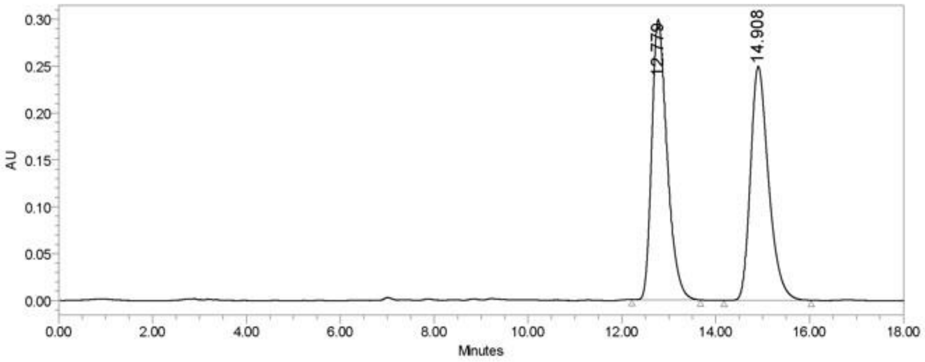

	RT	Area	% Area	Height
1	12.779	6779380	50.30	298984
2	14.908	6698344	49.70	249534

Asy-**3n** (DyKAT)

SAMPLE INFORMATION

Sample Name:	xt-3-78-RAC-20%-AD	Acquired By:	System
Sample Type:	Unknown	Sample Set Name:	
Vial:	42	Acq. Method Set:	20%qb
Injection #:	1	Processing Method:	xt 3 79 asy
Injection Volume:	10.00 ul	Channel Name:	254.0nm
Run Time:	60.0 Minutes	Proc. Chnl. Descr.:	2998 PDA 254.0 nm (2998
Date Acquired:	1/26/2022 10:17:00 PM CST		
Date Processed:	5/5/2022 9:19:19 PM CST		

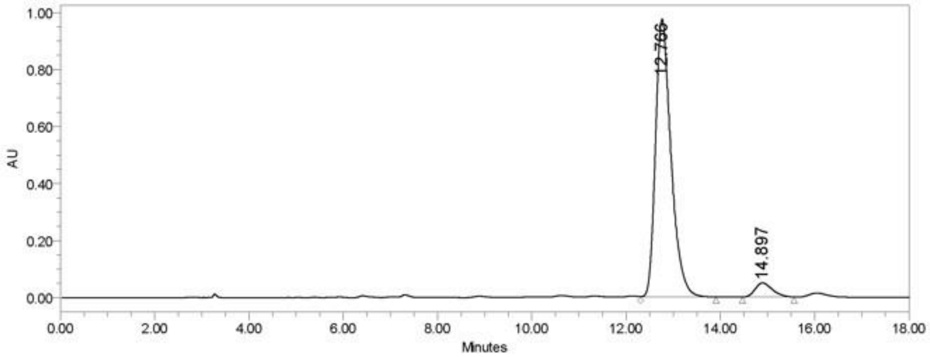

	RT	Area	% Area	Height
1	12.766	22203709	94.52	974707
2	14.897	1288013	5.48	50106

Supplementary Figure 69. HPLC spectra of compound **3n**

Rac-**3o**

SAMPLE INFORMATION

Sample Name:	xt-2-159-rac-20%-IG	Acquired By:	System
Sample Type:	Unknown	Sample Set Name	1026
Vial:	55	Acq. Method Set:	20% quanbo
Injection #:	1	Processing Method	XT 2159 RAC
Injection Volume:	10.00 ul	Channel Name:	254.0nm
Run Time:	25.0 Minutes	Proc. Chnl. Descr.:	2998 PDA 254.0 nm (2998
Date Acquired:	10/26/2021 4:46:12 PM CST		
Date Processed:	10/26/2021 5:16:22 PM CST		

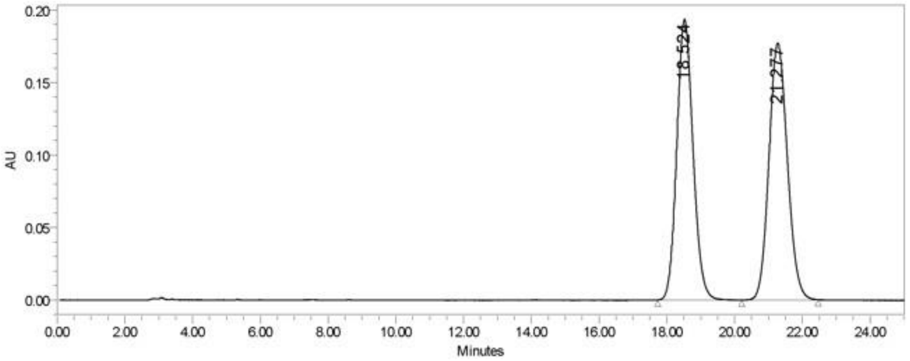

	RT	Area	% Area	Height
1	18.524	6476700	49.26	193897
2	21.277	6671291	50.74	177270

Asy-**3o** (DyKAT)

SAMPLE INFORMATION

Sample Name:	xt-2-161-2-20%-IG	Acquired By:	System
Sample Type:	Unknown	Sample Set Name	
Vial:	71	Acq. Method Set:	20% quanbo
Injection #:	1	Processing Method	XT 2 160 2
Injection Volume:	10.00 ul	Channel Name:	254.0nm
Run Time:	30.0 Minutes	Proc. Chnl. Descr.:	2998 PDA 254.0 nm (2998
Date Acquired:	10/25/2021 4:57:13 PM CST		
Date Processed:	10/26/2021 5:20:53 PM CST		

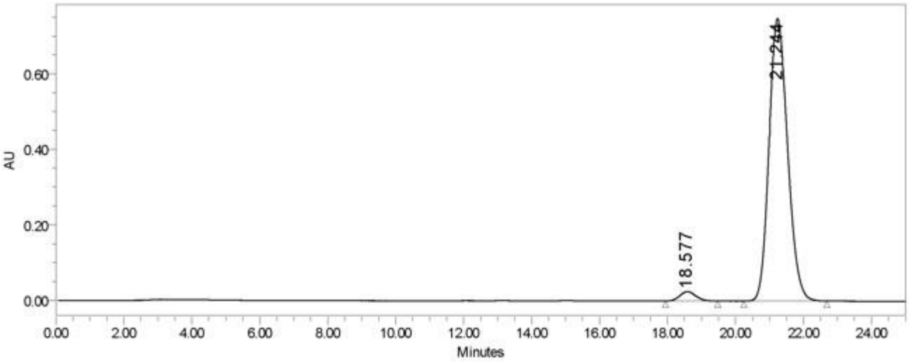

	RT	Area	% Area	Height
1	18.577	841503	2.90	25243
2	21.244	28203690	97.10	748004

Supplementary Figure 70. HPLC spectra of compound **3o**

Rac-**3p**

SAMPLE INFORMATION

Sample Name:	xt-2-114-rac-5%-IA	Acquired By:	System
Sample Type:	Unknown	Sample Set Name:	
Vial:	48	Acq. Method Set:	5%qb
Injection #:	1	Processing Method:	XT 2 114 RAC
Injection Volume:	10.00 ul	Channel Name:	254.0nm
Run Time:	60.0 Minutes	Proc. Chnl. Descr.:	2998 PDA 254.0 nm (2998
Date Acquired:	9/4/2021 10:21:16 PM CST		
Date Processed:	10/26/2021 10:15:11 PM CST		

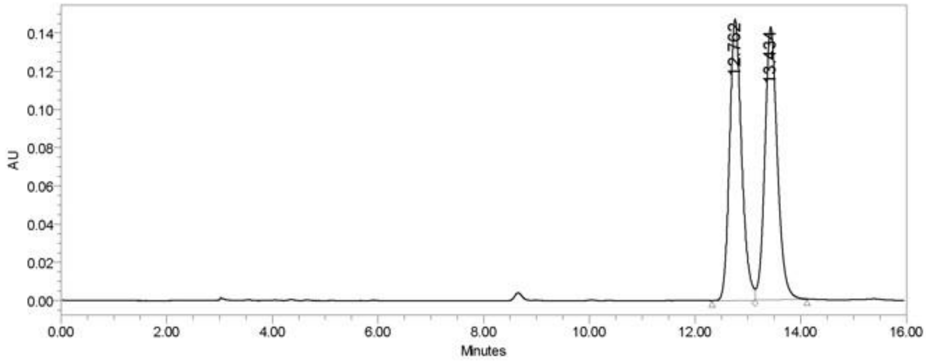

	RT	Area	% Area	Height
1	12.762	2329916	49.61	147157
2	13.434	2366227	50.39	142783

Asy-**3p** (KR)

SAMPLE INFORMATION

Sample Name:	xt-2-115-2-5%-IA	Acquired By:	System
Sample Type:	Unknown	Sample Set Name:	0904
Vial:	70	Acq. Method Set:	5%qb
Injection #:	1	Processing Method:	XT 2 115 2
Injection Volume:	10.00 ul	Channel Name:	254.0nm
Run Time:	18.0 Minutes	Proc. Chnl. Descr.:	2998 PDA 254.0 nm (2998
Date Acquired:	9/4/2021 10:42:54 PM CST		
Date Processed:	10/26/2021 10:13:31 PM CST		

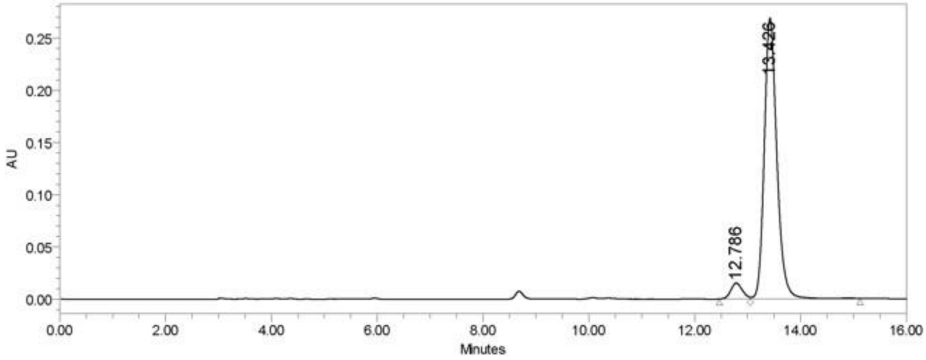

	RT	Area	% Area	Height
1	12.786	236368	5.04	15203
2	13.426	4451607	94.96	268885

Supplementary Figure 71. HPLC spectra of compound **3p**

Rac-**1a**

SAMPLE INFORMATION

Sample Name:	xt-2-82-s-RAC-20%-AD	Acquired By:	System
Sample Type:	Unknown	Sample Set Name	
Vial:	9	Acq. Method Set:	20% quanbo
Injection #:	1	Processing Method	XT 2 80 S RAC
Injection Volume:	10.00 ul	Channel Name:	254.0nm
Run Time:	10.0 Minutes	Proc. Chnl. Descr.:	2998 PDA 254.0 nm (2998
Date Acquired:	8/2/2021 8:37:12 PM CST		
Date Processed:	8/2/2021 8:46:21 PM CST		

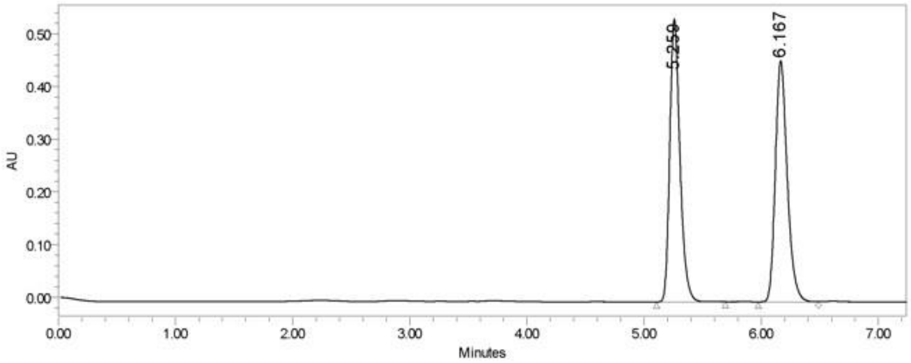

	RT	Area	% Area	Height
1	5.259	3230167	49.96	536377
2	6.167	3235025	50.04	457562

Asy-**1a**

SAMPLE INFORMATION

Sample Name:	xt-2-82-s-20%-AD	Acquired By:	System
Sample Type:	Unknown	Sample Set Name	08022
Vial:	120	Acq. Method Set:	20% quanbo
Injection #:	1	Processing Method	XT 2 82 S
Injection Volume:	10.00 ul	Channel Name:	254.0nm
Run Time:	7.2 Minutes	Proc. Chnl. Descr.:	2998 PDA 254.0 nm (2998
Date Acquired:	8/2/2021 8:51:41 PM CST		
Date Processed:	8/2/2021 8:59:31 PM CST		

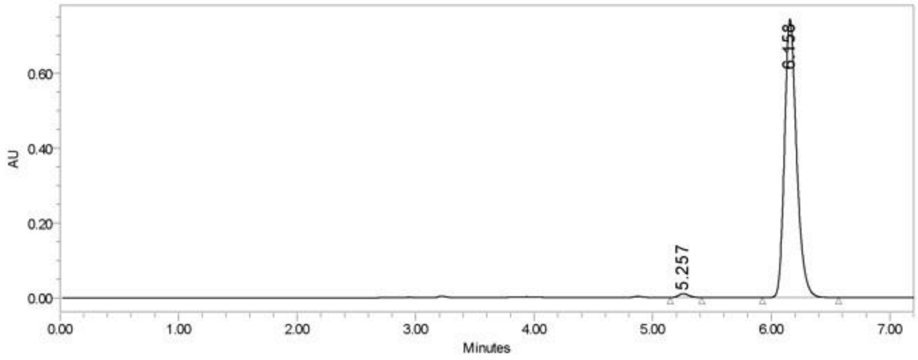

	RT	Area	% Area	Height
1	5.257	60022	1.12	10240
2	6.158	5282157	98.88	743161

Supplementary Figure 72. HPLC spectra of compound **1a**

Rac-**1ac**

SAMPLE INFORMATION

Sample Name:	xt-2-147-s-rac-20%-AD	Acquired By:	System
Sample Type:	Unknown	Sample Set Name:	10132
Vial:	77	Acq. Method Set:	20%qb
Injection #:	1	Processing Method:	XT 2 147 S RAC
Injection Volume:	10.00 ul	Channel Name:	254.0nm
Run Time:	7.9 Minutes	Proc. Chnl. Descr.:	2998 PDA 254.0 nm (2998
Date Acquired:	10/13/2021 9:13:28 PM CST		
Date Processed:	10/13/2021 9:37:53 PM CST		

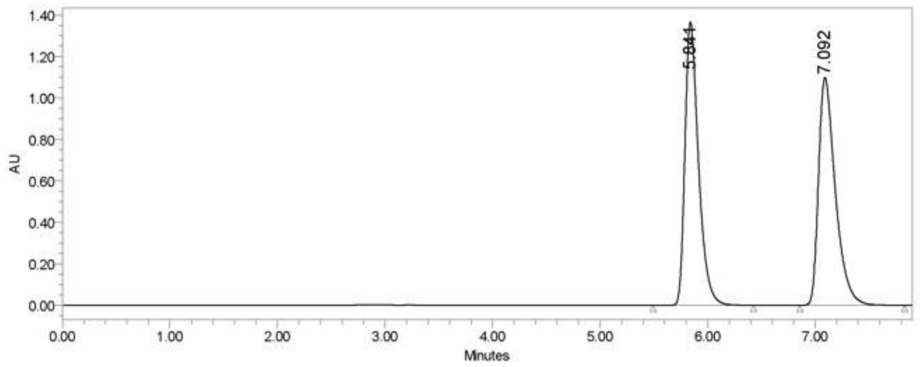

	RT	Area	% Area	Height
1	5.841	11852359	49.95	1366135
2	7.092	11875214	50.05	1098427

Asy-**1ac**

SAMPLE INFORMATION

Sample Name:	xt-2-147-3-s-20%-AD	Acquired By:	System
Sample Type:	Unknown	Sample Set Name:	10133
Vial:	75	Acq. Method Set:	20%qb
Injection #:	1	Processing Method:	XT 2 147 3 S
Injection Volume:	10.00 ul	Channel Name:	254.0nm
Run Time:	7.9 Minutes	Proc. Chnl. Descr.:	2998 PDA 254.0 nm (2998
Date Acquired:	10/13/2021 9:25:46 PM CST		
Date Processed:	10/13/2021 9:35:39 PM CST		

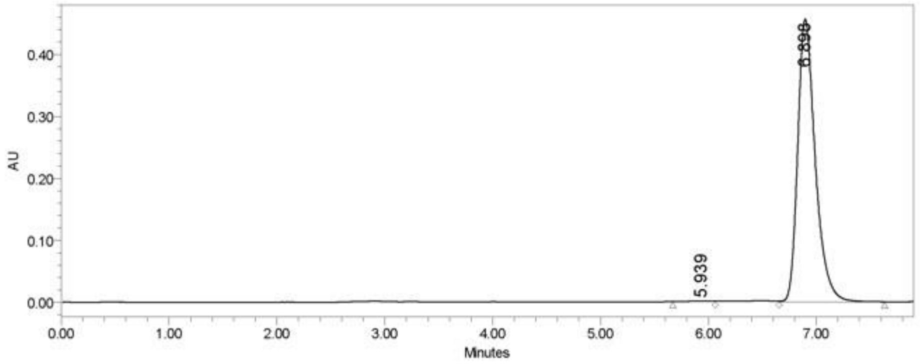

	RT	Area	% Area	Height
1	5.939	13483	0.26	860
2	6.898	5154257	99.74	455912

Supplementary Figure 73. HPLC spectra of compound **1ac**

Rac-**1ad**

SAMPLE INFORMATION

Sample Name:	xt-2-117-s-rac-20%AD	Acquired By:	System
Sample Type:	Unknown	Sample Set Name:	0
Vial:	34	Acq. Method Set:	20%qb
Injection #:	1	Processing Method:	xt 2 117 s rac
Injection Volume:	10.00 ul	Channel Name:	254.0nm
Run Time:	10.0 Minutes	Proc. Chnl. Descr.:	2998 PDA 254.0 nm (2998
Date Acquired:	10/11/2021 9:34:35 PM CST		
Date Processed:	10/12/2021 5:12:58 PM CST		

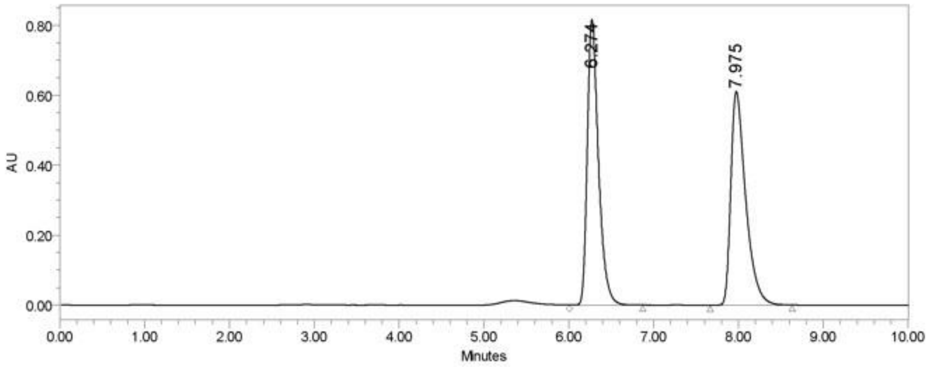

	RT	Area	% Area	Height
1	6.274	7466763	50.02	817354
2	7.975	7461290	49.98	611754

Asy-**1ad**

SAMPLE INFORMATION

Sample Name:	xt-2-117-2-s-20%AD	Acquired By:	System
Sample Type:	Unknown	Sample Set Name:	10112
Vial:	33	Acq. Method Set:	20%qb
Injection #:	1	Processing Method:	xt 2 117 3 s
Injection Volume:	10.00 ul	Channel Name:	254.0nm
Run Time:	10.0 Minutes	Proc. Chnl. Descr.:	2998 PDA 254.0 nm (2998
Date Acquired:	10/11/2021 9:06:29 PM CST		
Date Processed:	10/12/2021 5:11:28 PM CST		

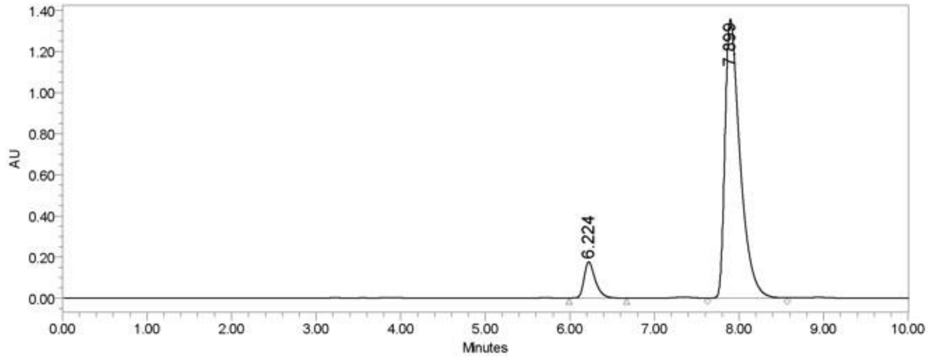

	RT	Area	% Area	Height
1	6.224	1623317	8.79	176177
2	7.899	16847352	91.21	1357061

Supplementary Figure 74. HPLC spectra of compound **1ad**

Rac-**1ae**

SAMPLE INFORMATION

Sample Name:	xt-2-108-s-rac-20%-AD	Acquired By:	System
Sample Type:	Unknown	Sample Set Name:	0831
Vial:	104	Acq. Method Set:	20%qb
Injection #:	1	Processing Method:	xt 2 108 s rac
Injection Volume:	10.00 ul	Channel Name:	254.0nm
Run Time:	8.0 Minutes	Proc. Chnl. Descr.:	2998 PDA 254.0 nm (2998
Date Acquired:	8/31/2021 12:00:48 AM CST		
Date Processed:	8/31/2021 8:49:26 AM CST		

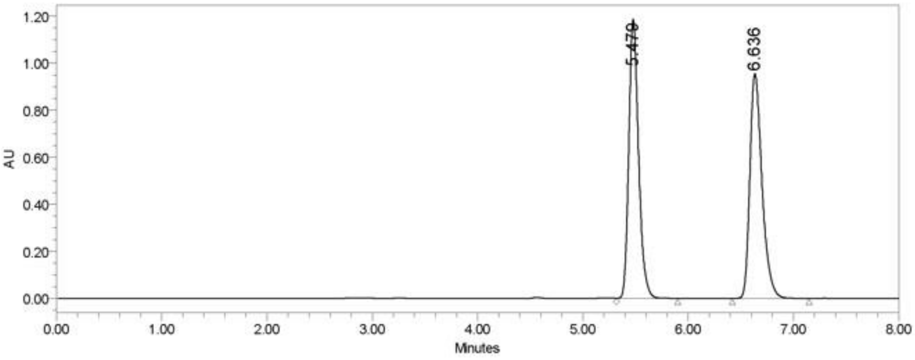

	RT	Area	% Area	Height
1	5.479	7649585	49.90	1186949
2	6.636	7678894	50.10	955320

Asy-**1ae**

SAMPLE INFORMATION

Sample Name:	xt-2-109-4-s-20%-AD	Acquired By:	System
Sample Type:	Unknown	Sample Set Name:	0831
Vial:	105	Acq. Method Set:	20%qb
Injection #:	1	Processing Method:	xt 2 109 4 s
Injection Volume:	10.00 ul	Channel Name:	254.0nm
Run Time:	8.0 Minutes	Proc. Chnl. Descr.:	2998 PDA 254.0 nm (2998
Date Acquired:	8/31/2021 12:14:41 AM CST		
Date Processed:	8/31/2021 8:51:22 AM CST		

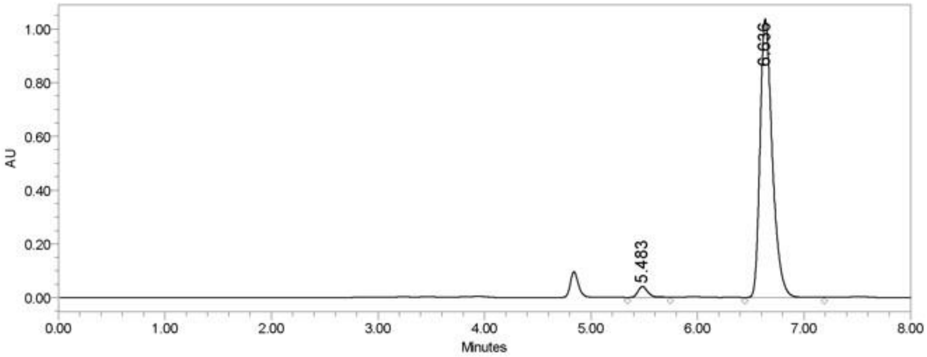

	RT	Area	% Area	Height
1	5.483	280651	3.26	41359
2	6.636	8338747	96.74	1037336

Supplementary Figure 75. HPLC spectra of compound **1ae**

Rac-**1ag**

SAMPLE INFORMATION

Sample Name:	xt-2-100-rac-20%-AD	Acquired By:	System
Sample Type:	Unknown	Sample Set Name:	0901
Vial:	31	Acq. Method Set:	20%qb
Injection #:	1	Processing Method:	XT 2 100 RAC
Injection Volume:	10.00 ul	Channel Name:	254.0nm
Run Time:	13.0 Minutes	Proc. Chnl. Descr.:	2998 PDA 254.0 nm (2998
Date Acquired:	8/31/2021 10:52:33 PM CST		
Date Processed:	9/1/2021 8:41:57 AM CST		

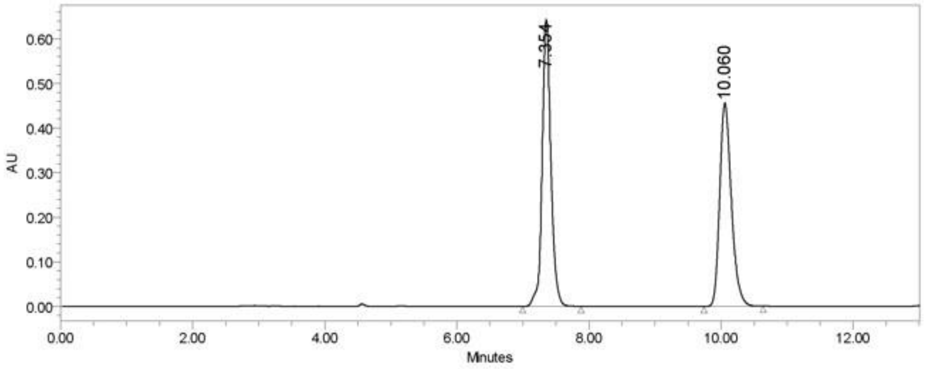

	RT	Area	% Area	Height
1	7.354	5889394	51.15	643621
2	10.060	5624535	48.85	455979

Asy-**1ag**

SAMPLE INFORMATION

Sample Name:	xt-2-113-1-S-20%-AD	Acquired By:	System
Sample Type:	Unknown	Sample Set Name:	0901
Vial:	32	Acq. Method Set:	20%qb
Injection #:	1	Processing Method:	XT 2 113 1 S
Injection Volume:	10.00 ul	Channel Name:	254.0nm
Run Time:	13.0 Minutes	Proc. Chnl. Descr.:	2998 PDA 254.0 nm (2998
Date Acquired:	8/31/2021 11:11:23 PM CST		
Date Processed:	9/1/2021 8:43:23 AM CST		

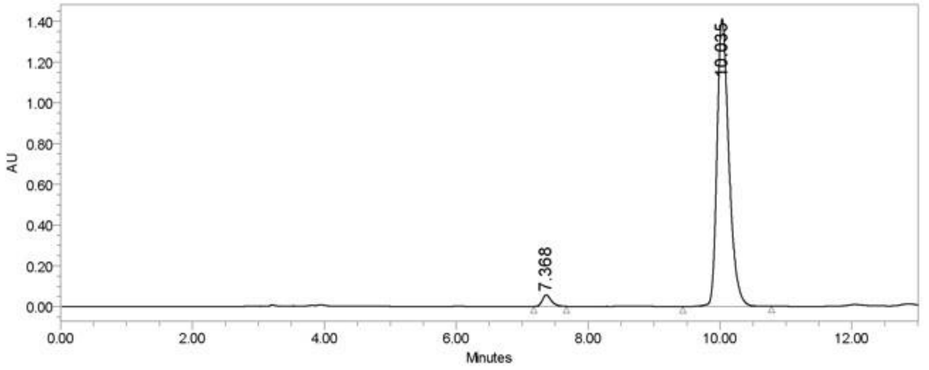

	RT	Area	% Area	Height
1	7.368	535608	2.93	56365
2	10.035	17759957	97.07	1411623

Supplementary Figure 76. HPLC spectra of compound **1ag**

Rac-**1ah**

SAMPLE INFORMATION

Sample Name:	xt-2-75-2-s-RAC-20%-AD	Acquired By:	System
Sample Type:	Unknown	Sample Set Name:	
Vial:	7	Acq. Method Set:	20%qb
Injection #:	1	Processing Method:	XT 2 75 2 S RAC
Injection Volume:	10.00 ul	Channel Name:	254.0nm
Run Time:	60.0 Minutes	Proc. Chnl. Descr.:	2998 PDA 254.0 nm (2998
Date Acquired:	1/21/2022 11:24:11 AM CST		
Date Processed:	1/21/2022 2:59:52 PM CST		

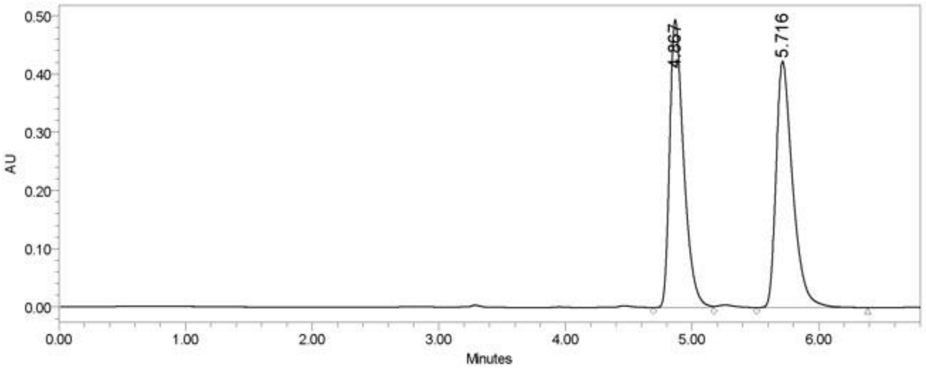

	RT	Area	% Area	Height
1	4.867	3820173	49.74	494249
2	5.716	3859991	50.26	423011

Asy-**1ah**

SAMPLE INFORMATION

Sample Name:	xt-2-75-2-s-20%-AD	Acquired By:	System
Sample Type:	Unknown	Sample Set Name:	
Vial:	34	Acq. Method Set:	20%qb
Injection #:	1	Processing Method:	xt 2 75 2 s
Injection Volume:	10.00 ul	Channel Name:	254.0nm
Run Time:	60.0 Minutes	Proc. Chnl. Descr.:	2998 PDA 254.0 nm (2998
Date Acquired:	1/21/2022 2:44:15 PM CST		
Date Processed:	1/21/2022 2:57:49 PM CST		

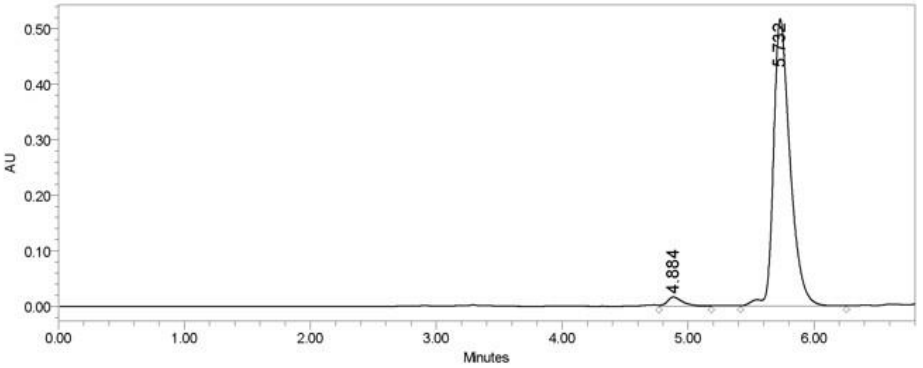

	RT	Area	% Area	Height
1	4.884	148173	3.02	16783
2	5.732	4751598	96.98	516764

Supplementary Figure 77. HPLC spectra of compound **1ah**

Rac-**1aj**

SAMPLE INFORMATION

Sample Name:	xt-2-111-1-s-rac-20%-AD	Acquired By:	System
Sample Type:	Unknown	Sample Set Name:	08281
Vial:	40	Acq. Method Set:	20%qb
Injection #:	1	Processing Method:	xt 2 110 1 s rac
Injection Volume:	10.00 ul	Channel Name:	254.0nm
Run Time:	15.0 Minutes	Proc. Chnl. Descr.:	2998 PDA 254.0 nm (2998
Date Acquired:	8/28/2021 9:59:25 PM CST		
Date Processed:	8/28/2021 10:29:42 PM CST		

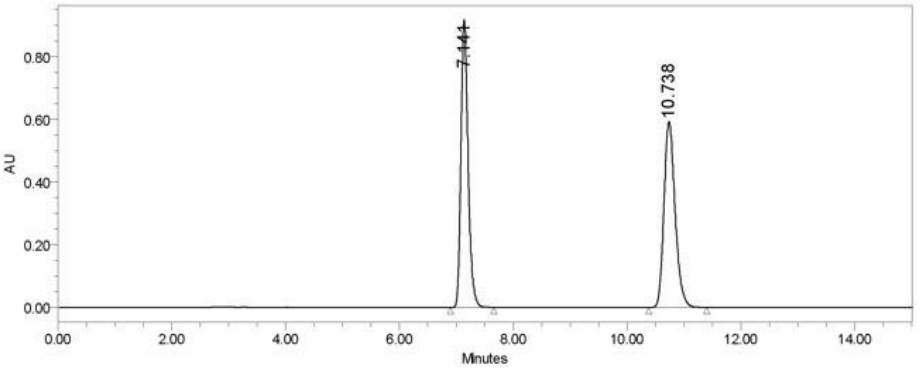

	RT	Area	% Area	Height
1	7.141	7985586	49.90	917612
2	10.738	8016575	50.10	593408

Asy-**1aj**

SAMPLE INFORMATION

Sample Name:	xt-2-111-1-s-20%-AD	Acquired By:	System
Sample Type:	Unknown	Sample Set Name:	08281
Vial:	41	Acq. Method Set:	20%qb
Injection #:	1	Processing Method:	xt 2 111 1 s
Injection Volume:	10.00 ul	Channel Name:	254.0nm
Run Time:	15.0 Minutes	Proc. Chnl. Descr.:	2998 PDA 254.0 nm (2998
Date Acquired:	8/28/2021 10:15:07 PM CST		
Date Processed:	8/28/2021 10:31:43 PM CST		

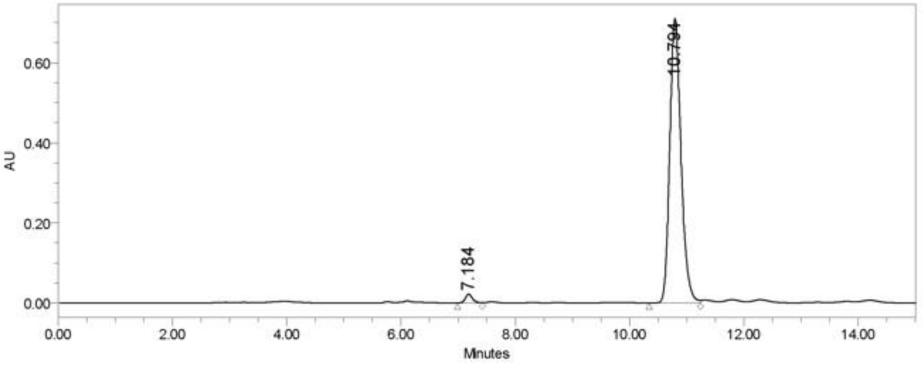

	RT	Area	% Area	Height
1	7.184	194141	1.97	22126
2	10.794	9663393	98.03	710636

Supplementary Figure 78. HPLC spectra of compound **1aj**

Rac-**1ap**

SAMPLE INFORMATION

Sample Name:	xt-2-114-S-rac-20%-AD	Acquired By:	System
Sample Type:	Unknown	Sample Set Name:	
Vial:	89	Acq. Method Set:	20%qb
Injection #:	1	Processing Method:	XT 2 114 S RAC
Injection Volume:	10.00 ul	Channel Name:	254.0nm
Run Time:	60.0 Minutes	Proc. Chnl. Descr.:	2998 PDA 254.0 nm (2998
Date Acquired:	9/2/2021 10:02:05 PM CST		
Date Processed:	10/26/2021 10:18:47 PM CST		

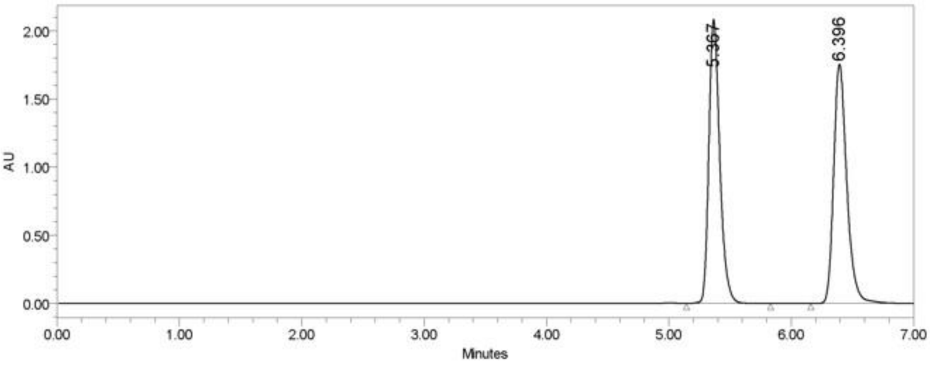

	RT	Area	% Area	Height
1	5.367	12944090	49.64	2082981
2	6.396	13131888	50.36	1755839

Asy-**1ap**

SAMPLE INFORMATION

Sample Name:	xt-2-115-2-s-20%-AD	Acquired By:	System
Sample Type:	Unknown	Sample Set Name:	
Vial:	20	Acq. Method Set:	20%qb
Injection #:	1	Processing Method:	XT 2 115 S RAC
Injection Volume:	10.00 ul	Channel Name:	254.0nm
Run Time:	20.0 Minutes	Proc. Chnl. Descr.:	2998 PDA 254.0 nm (2998
Date Acquired:	9/3/2021 7:24:40 PM CST		
Date Processed:	10/26/2021 10:17:04 PM CST		

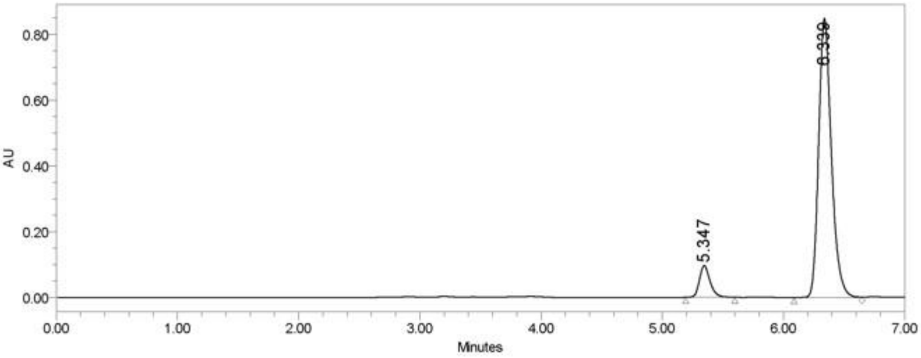

	RT	Area	% Area	Height
1	5.347	591677	8.71	96406
2	6.339	6199568	91.29	848444

Supplementary Figure 79. HPLC spectra of compound **1ap**

Rac-**4a**

SAMPLE INFORMATION

Sample Name:	xt-2-74-1-RAC-10%-IA	Acquired By:	System
Sample Type:	Unknown	Sample Set Name:	
Vial:	93	Acq. Method Set:	10%qb
Injection #:	1	Processing Method:	XT 2 74 1
Injection Volume:	10.00 ul	Channel Name:	254.0nm
Run Time:	60.0 Minutes	Proc. Chnl. Descr.:	2998 PDA 254.0 nm (2998
Date Acquired:	7/29/2021 8:48:30 PM CST		
Date Processed:	7/29/2021 9:48:37 PM CST		

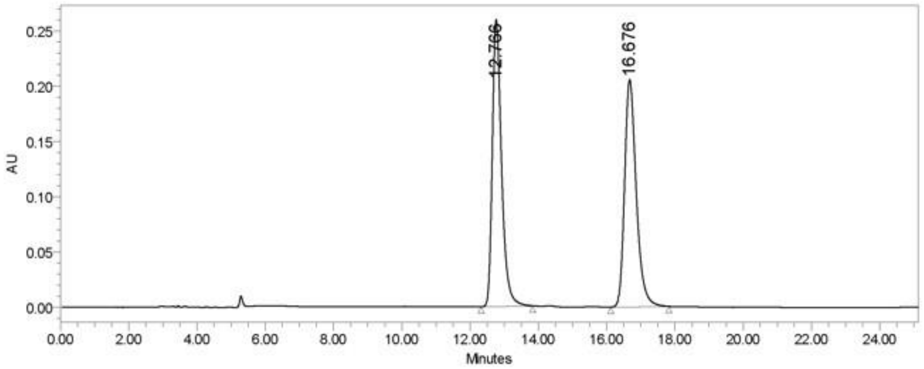

	RT	Area	% Area	Height
1	12.766	4887755	49.94	259567
2	16.676	4899185	50.06	205730

Asy-**4a**

SAMPLE INFORMATION

Sample Name:	xt-2-74-2-10%-IA	Acquired By:	System
Sample Type:	Unknown	Sample Set Name:	0729
Vial:	94	Acq. Method Set:	10%qb
Injection #:	1	Processing Method:	XT 2 74 2
Injection Volume:	10.00 ul	Channel Name:	254.0nm
Run Time:	25.0 Minutes	Proc. Chnl. Descr.:	2998 PDA 254.0 nm (2998
Date Acquired:	7/29/2021 9:14:50 PM CST		
Date Processed:	7/29/2021 9:46:10 PM CST		

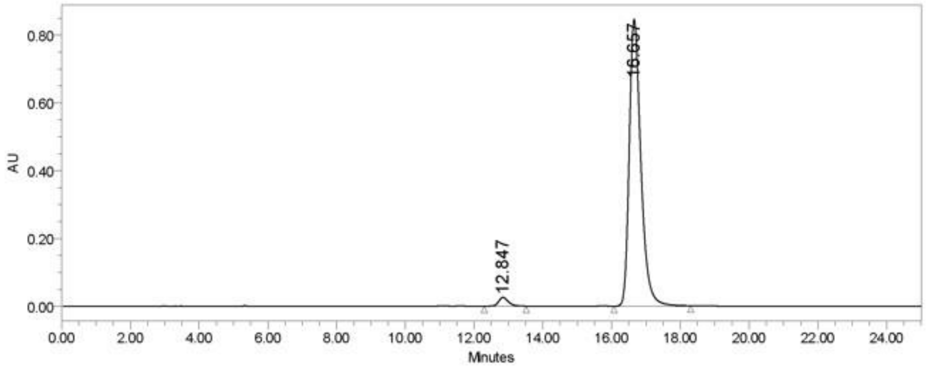

	RT	Area	% Area	Height
1	12.847	515250	2.52	26189
2	16.657	19951144	97.48	846223

Supplementary Figure 80. HPLC spectra of compound **4a**

Rac-**4b**

SAMPLE INFORMATION

Sample Name:	xt-2-163-rac-20%-IA	Acquired By:	System
Sample Type:	Unknown	Sample Set Name	
Vial:	9	Acq. Method Set:	20% quanbo
Injection #:	1	Processing Method	XT 2 163 RAC
Injection Volume:	10.00 ul	Channel Name:	254.0nm
Run Time:	60.0 Minutes	Proc. Chnl. Descr.:	2998 PDA 254.0 nm (2998
Date Acquired:	10/26/2021 9:50:41 PM CST		
Date Processed:	10/26/2021 10:19:54 PM CST		

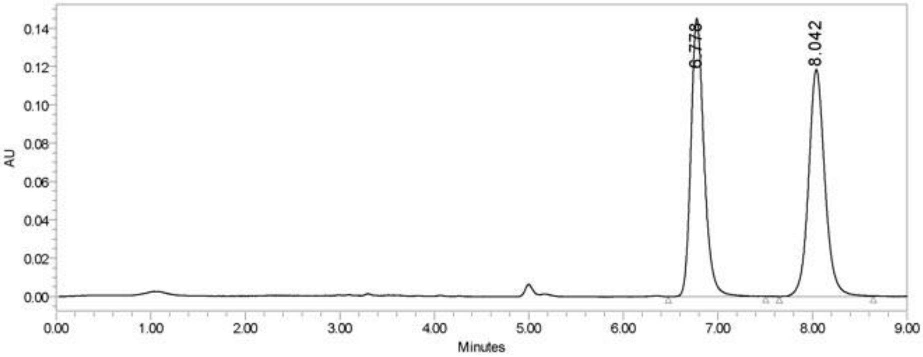

	RT	Area	% Area	Height
1	6.778	1409690	49.80	145242
2	8.042	1420756	50.20	118521

Asy-**4b**

SAMPLE INFORMATION

Sample Name:	xt-2-164-1-20%-IA	Acquired By:	System
Sample Type:	Unknown	Sample Set Name	
Vial:	29	Acq. Method Set:	20% quanbo
Injection #:	1	Processing Method	XT 2 164 1
Injection Volume:	10.00 ul	Channel Name:	254.0nm
Run Time:	60.0 Minutes	Proc. Chnl. Descr.:	2998 PDA 254.0 nm (2998
Date Acquired:	10/26/2021 10:08:36 PM CST		
Date Processed:	10/26/2021 10:22:25 PM CST		

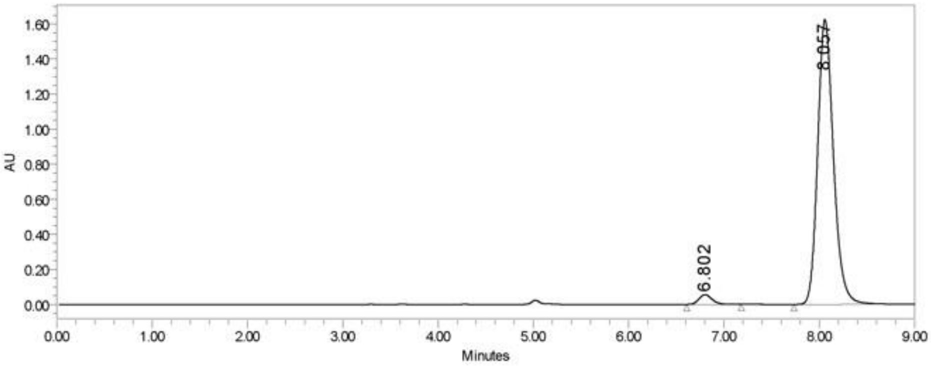

	RT	Area	% Area	Height
1	6.802	539205	2.79	56018
2	8.057	18780436	97.21	1625292

Supplementary Figure 81. HPLC spectra of compound **4b**

Rac-**4c**

SAMPLE INFORMATION

Sample Name:	xt-2-162-rac-20%-IA	Acquired By:	System
Sample Type:	Unknown	Sample Set Name:	
Vial:	6	Acq. Method Set:	20%qb
Injection #:	1	Processing Method:	XT 2 162 RAC
Injection Volume:	10.00 ul	Channel Name:	254.0nm
Run Time:	60.0 Minutes	Proc. Chnl. Descr.:	2998 PDA 254.0 nm (2998
Date Acquired:	10/27/2021 4:27:05 PM CST		
Date Processed:	10/28/2021 10:42:13 AM CST		

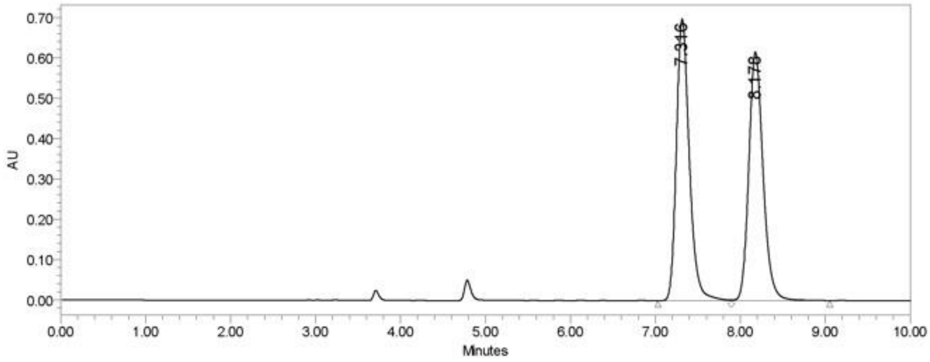

	RT	Area	% Area	Height
1	7.316	7376925	50.62	698200
2	8.178	7197318	49.38	616340

Asy-**4c**

SAMPLE INFORMATION

Sample Name:	xt-2-157-20%-IA	Acquired By:	System
Sample Type:	Unknown	Sample Set Name:	
Vial:	28	Acq. Method Set:	20%qb
Injection #:	1	Processing Method:	XT 2 157
Injection Volume:	10.00 ul	Channel Name:	254.0nm
Run Time:	15.0 Minutes	Proc. Chnl. Descr.:	2998 PDA 254.0 nm (2998
Date Acquired:	10/27/2021 5:03:34 PM CST		
Date Processed:	10/28/2021 10:43:59 AM CST		

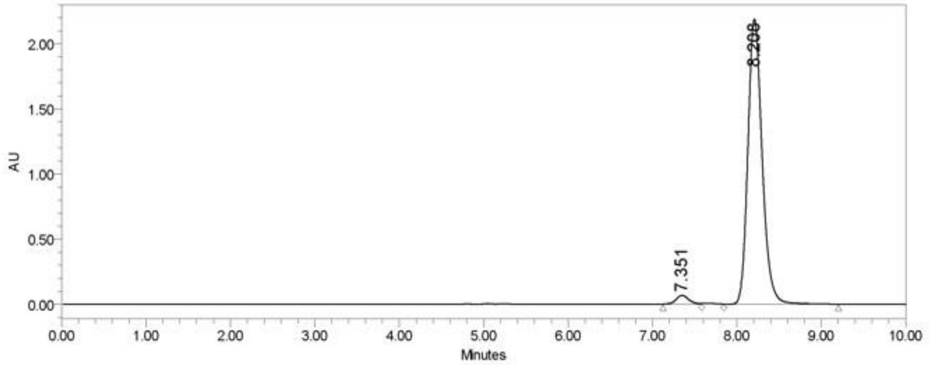

	RT	Area	% Area	Height
1	7.351	712143	2.70	67228
2	8.208	25682876	97.30	2187961

Supplementary Figure 82. HPLC spectra of compound **4c**

Rac-**4d**

SAMPLE INFORMATION

Sample Name:	xt-2-85-RAC-20%-AD	Acquired By:	System
Sample Type:	Unknown	Sample Set Name	
Vial:	31	Acq. Method Set:	20% quanbo
Injection #:	1	Processing Method	XT 2 85 RAC
Injection Volume:	10.00 ul	Channel Name:	254.0nm
Run Time:	30.0 Minutes	Proc. Chnl. Descr.:	2998 PDA 254.0 nm (2998
Date Acquired:	8/5/2021 7:04:44 PM CST		
Date Processed:	8/6/2021 10:23:50 AM CST		

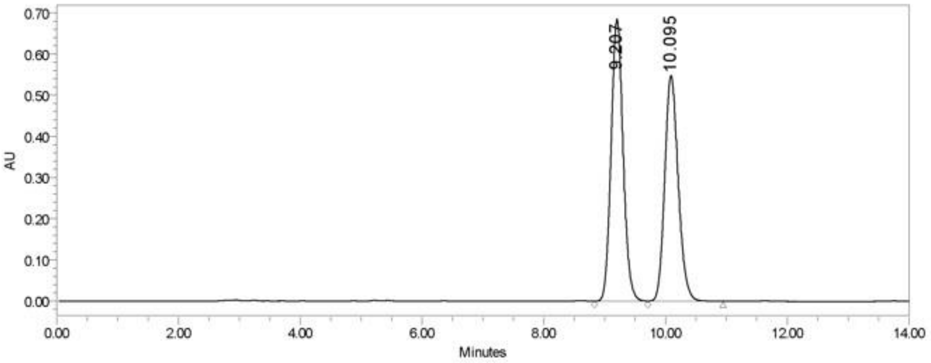

	RT	Area	% Area	Height
1	9.207	9368825	52.59	685041
2	10.095	8444421	47.41	548260

Asy-**4d**

SAMPLE INFORMATION

Sample Name:	xt-2-87'-20%-AD	Acquired By:	System
Sample Type:	Unknown	Sample Set Name	
Vial:	33	Acq. Method Set:	20% quanbo
Injection #:	1	Processing Method	XT 2 87
Injection Volume:	2.00 ul	Channel Name:	254.0nm
Run Time:	14.0 Minutes	Proc. Chnl. Descr.:	2998 PDA 254.0 nm (2998
Date Acquired:	8/6/2021 10:39:12 AM CST		
Date Processed:	8/6/2021 10:55:30 AM CST		

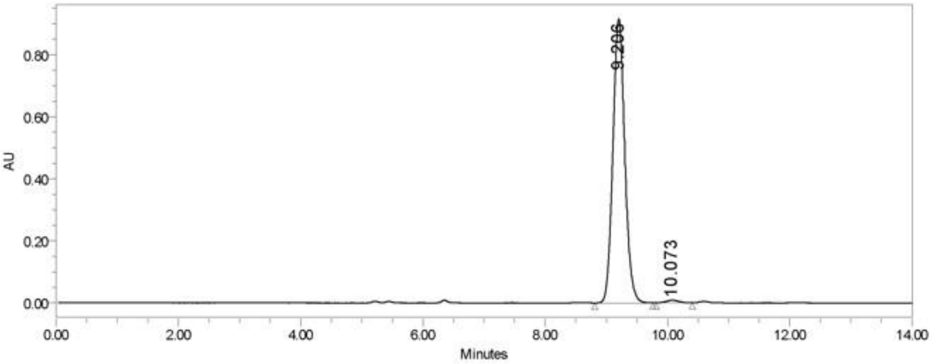

	RT	Area	% Area	Height
1	9.206	12316620	99.03	915624
2	10.073	120446	0.97	8473

Supplementary Figure 83. HPLC spectra of compound **4d**

Rac-**4e**

SAMPLE INFORMATION

Sample Name:	xt-2-165-rac-20%-IA	Acquired By:	System
Sample Type:	Unknown	Sample Set Name:	
Vial:	7	Acq. Method Set:	20%qb
Injection #:	1	Processing Method:	XT 2 165 RAC
Injection Volume:	10.00 ul	Channel Name:	254.0nm
Run Time:	60.0 Minutes	Proc. Chnl. Descr.:	2998 PDA 254.0 nm (2998
Date Acquired:	10/27/2021 4:44:51 PM CST		
Date Processed:	10/28/2021 10:38:02 AM CST		

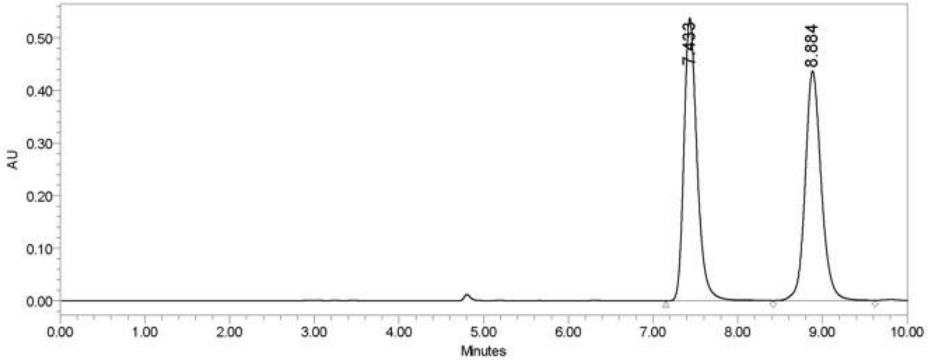

	RT	Area	% Area	Height
1	7.433	5790110	49.98	537731
2	8.884	5793880	50.02	437297

Asy-**4e**

SAMPLE INFORMATION

Sample Name:	xt-2-166-20%-IA	Acquired By:	System
Sample Type:	Unknown	Sample Set Name:	
Vial:	8	Acq. Method Set:	20%qb
Injection #:	1	Processing Method:	XT 2 166
Injection Volume:	10.00 ul	Channel Name:	254.0nm
Run Time:	20.0 Minutes	Proc. Chnl. Descr.:	2998 PDA 254.0 nm (2998
Date Acquired:	10/28/2021 10:22:25 AM CST		
Date Processed:	10/28/2021 10:40:12 AM CST		

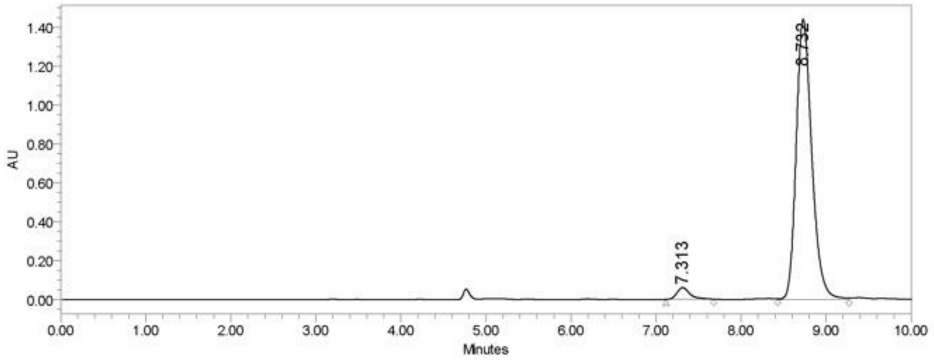

	RT	Area	% Area	Height
1	7.313	663477	3.50	60154
2	8.732	18299106	96.50	1440726

Supplementary Figure 84. HPLC spectra of compound **4e**

Rac-**4f**

SAMPLE INFORMATION

Sample Name:	xt-2-167-RAC-20%-IA	Acquired By:	System
Sample Type:	Unknown	Sample Set Name:	
Vial:	63	Acq. Method Set:	20%qb
Injection #:	1	Processing Method:	xt 2 167 rac
Injection Volume:	10.00 ul	Channel Name:	254.0nm
Run Time:	60.0 Minutes	Proc. Chnl. Descr.:	2998 PDA 254.0 nm (2998
Date Acquired:	10/27/2021 5:20:17 PM CST		
Date Processed:	10/28/2021 10:20:05 AM CST		

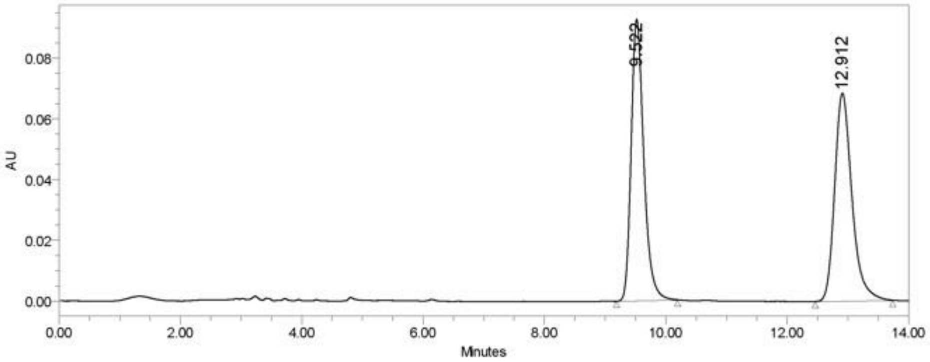

	RT	Area	% Area	Height
1	9.522	1347702	49.76	92783
2	12.912	1360972	50.24	68515

Asy-**4f**

SAMPLE INFORMATION

Sample Name:	xt-2-168-20%-IA	Acquired By:	System
Sample Type:	Unknown	Sample Set Name:	
Vial:	78	Acq. Method Set:	20%qb
Injection #:	1	Processing Method:	XT 2 168
Injection Volume:	10.00 ul	Channel Name:	254.0nm
Run Time:	60.0 Minutes	Proc. Chnl. Descr.:	2998 PDA 254.0 nm (2998
Date Acquired:	10/27/2021 9:36:47 PM CST		
Date Processed:	10/28/2021 10:22:01 AM CST		

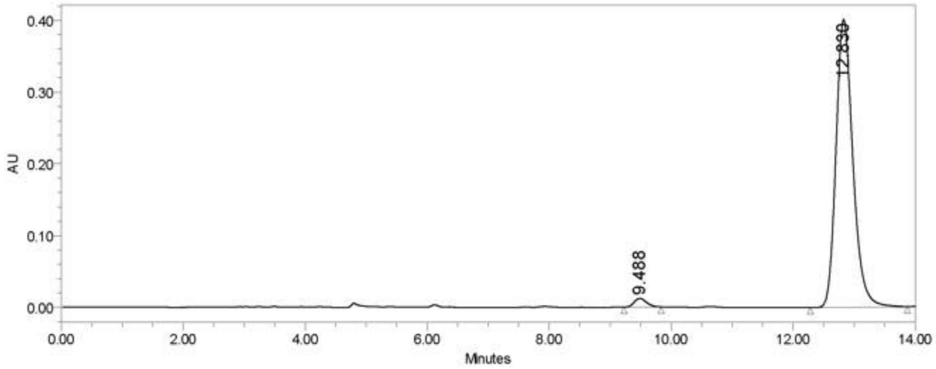

	RT	Area	% Area	Height
1	9.488	168525	2.13	12131
2	12.830	7742442	97.87	401480

Supplementary Figure 85. HPLC spectra of compound **4f**

Rac-**4g**

SAMPLE INFORMATION

Sample Name:	xt-2-177-rac-re-20%-IG	Acquired By:	System
Sample Type:	Unknown	Sample Set Name	
Vial:	92	Acq. Method Set:	20% quanbo
Injection #:	1	Processing Method	xt 2 177 rac
Injection Volume:	10.00 ul	Channel Name:	264.0nm
Run Time:	60.0 Minutes	Proc. Chnl. Descr.:	2998 PDA 264.0 nm (2998
Date Acquired:	11/3/2021 9:48:46 PM CST		
Date Processed:	11/3/2021 10:05:08 PM CST		

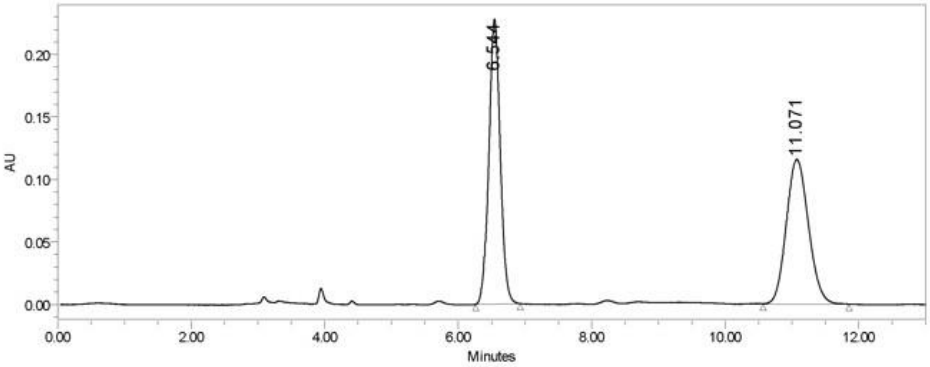

	RT	Area	% Area	Height
1	6.544	2702276	50.40	227890
2	11.071	2659861	49.60	115797

Asy-**4g**

SAMPLE INFORMATION

Sample Name:	xt-2-177-rac-20%-IG	Acquired By:	System
Sample Type:	Unknown	Sample Set Name	
Vial:	70	Acq. Method Set:	20% quanbo
Injection #:	1	Processing Method	XT 2 178
Injection Volume:	10.00 ul	Channel Name:	264.0nm
Run Time:	60.0 Minutes	Proc. Chnl. Descr.:	2998 PDA 264.0 nm (2998
Date Acquired:	11/3/2021 9:21:48 PM CST		
Date Processed:	11/3/2021 10:07:32 PM CST		

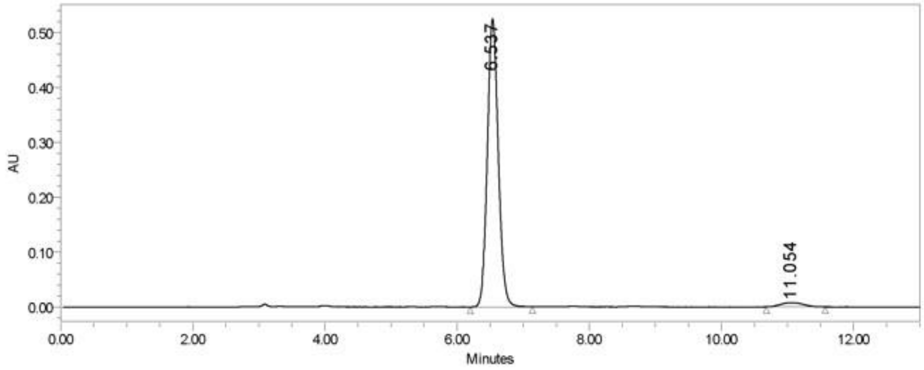

	RT	Area	% Area	Height
1	6.537	6036127	96.96	524631
2	11.054	189374	3.04	7855

Supplementary Figure 86. HPLC spectra of compound **4g**

Rac-**4h**

SAMPLE INFORMATION

Sample Name:	xt-3-16-rac-20%-IG	Acquired By:	System
Sample Type:	Unknown	Sample Set Name	1206
Vial:	56	Acq. Method Set:	20% quanbo
Injection #:	1	Processing Method	XT 3 16 RAC
Injection Volume:	10.00 ul	Channel Name:	254.0nm
Run Time:	15.0 Minutes	Proc. Chnl. Descr.:	2998 PDA 254.0 nm (2998
Date Acquired:	12/6/2021 3:26:42 PM CST		
Date Processed:	12/7/2021 2:54:44 PM CST		

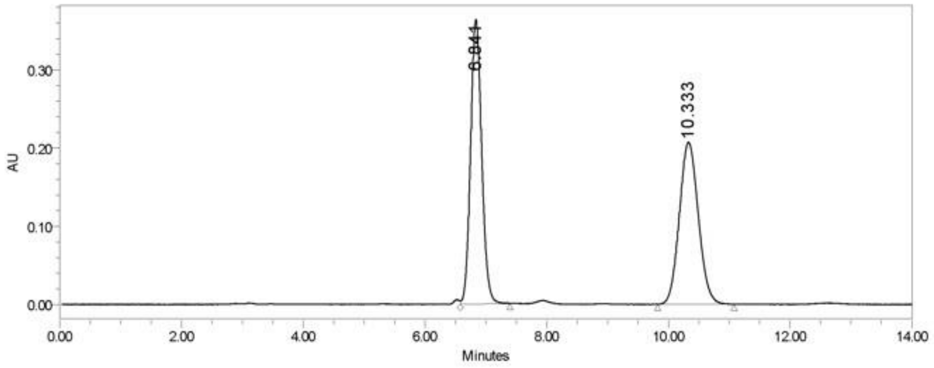

	RT	Area	% Area	Height
1	6.841	4557830	50.40	363358
2	10.333	4486296	49.60	207185

Asy-**4h**

SAMPLE INFORMATION

Sample Name:	xt-3-17-20%-IG	Acquired By:	System
Sample Type:	Unknown	Sample Set Name	
Vial:	17	Acq. Method Set:	20% quanbo
Injection #:	1	Processing Method	XT 3 17
Injection Volume:	10.00 ul	Channel Name:	254.0nm
Run Time:	60.0 Minutes	Proc. Chnl. Descr.:	2998 PDA 254.0 nm (2998
Date Acquired:	12/6/2021 4:10:03 PM CST		
Date Processed:	12/7/2021 2:53:19 PM CST		

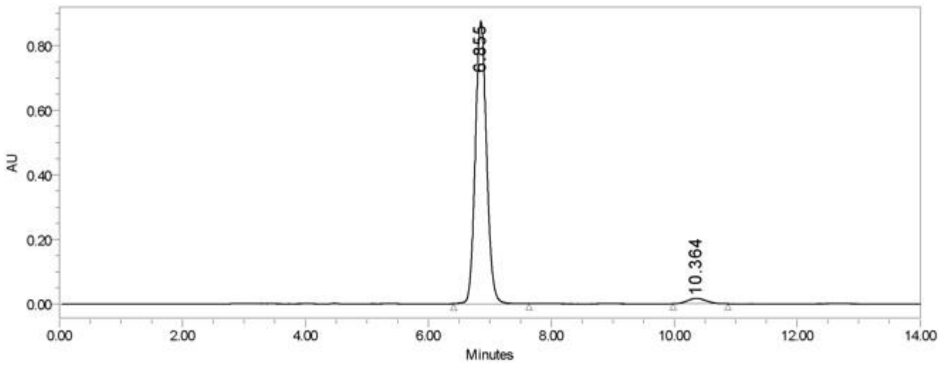

	RT	Area	% Area	Height
1	6.855	11037328	96.85	875580
2	10.364	359187	3.15	16842

Supplementary Figure 87. HPLC spectra of compound **4h**

Rac-**4i**

SAMPLE INFORMATION

Sample Name:	xt-2-172-RAC-20%-IG	Acquired By:	System
Sample Type:	Unknown	Sample Set Name	
Vial:	22	Acq. Method Set:	20% quanbo
Injection #:	1	Processing Method	xt 2 172 rac
Injection Volume:	10.00 ul	Channel Name:	254.0nm
Run Time:	60.0 Minutes	Proc. Chnl. Descr.:	2998 PDA 254.0 nm (2998
Date Acquired:	10/31/2021 2:35:52 PM CST		
Date Processed:	11/29/2021 3:41:06 PM CST		

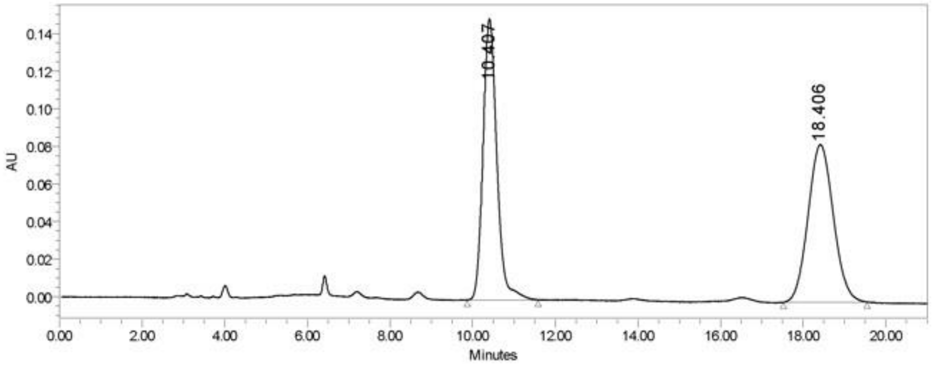

	RT	Area	% Area	Height
1	10.407	3451806	49.04	149455
2	18.406	3586285	50.96	83871

Asy-**4i**

SAMPLE INFORMATION

Sample Name:	xt-2-173-20%-IG	Acquired By:	System
Sample Type:	Unknown	Sample Set Name	
Vial:	23	Acq. Method Set:	20% quanbo
Injection #:	1	Processing Method	XT 2 173
Injection Volume:	10.00 ul	Channel Name:	254.0nm
Run Time:	60.0 Minutes	Proc. Chnl. Descr.:	2998 PDA 254.0 nm (2998
Date Acquired:	10/31/2021 2:01:32 PM CST		
Date Processed:	11/29/2021 3:43:08 PM CST		

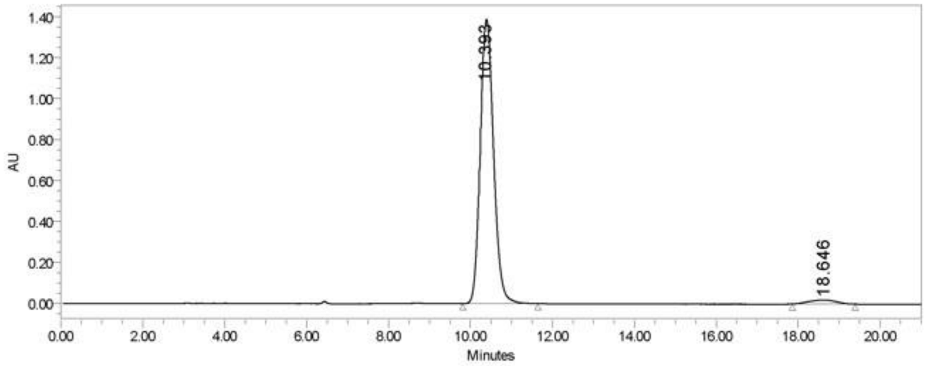

	RT	Area	% Area	Height
1	10.393	31126243	97.16	1387939
2	18.646	909667	2.84	19510

Supplementary Figure 88. HPLC spectra of compound **4i**

Rac-**4j**

SAMPLE INFORMATION

Sample Name:	xt-3-11-rac-20%-AD	Acquired By:	System
Sample Type:	Unknown	Sample Set Name	
Vial:	10	Acq. Method Set:	20% quanbo
Injection #:	1	Processing Method	XT 3 11 RAC
Injection Volume:	10.00 ul	Channel Name:	254.0nm
Run Time:	60.0 Minutes	Proc. Chnl. Descr.:	2998 PDA 254.0 nm (2998
Date Acquired:	12/2/2021 9:54:28 AM CST		
Date Processed:	12/2/2021 10:47:55 AM CST		

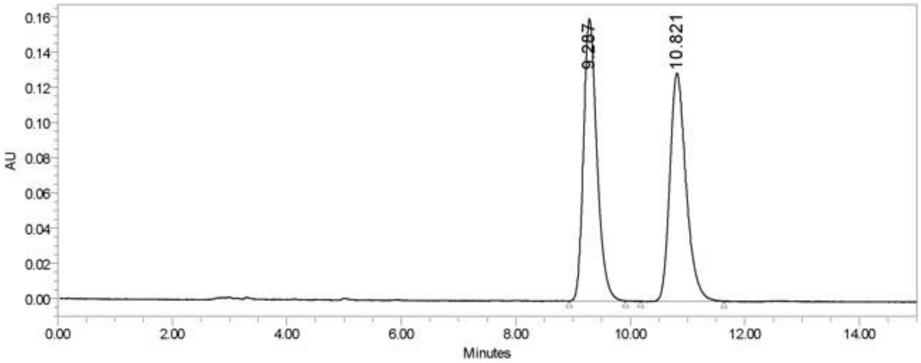

	RT	Area	% Area	Height
1	9.287	2601871	50.54	160310
2	10.821	2546684	49.46	129546

Asy-**4j**

SAMPLE INFORMATION

Sample Name:	xt-3-13-20%-AD	Acquired By:	System
Sample Type:	Unknown	Sample Set Name	
Vial:	42	Acq. Method Set:	20% quanbo
Injection #:	1	Processing Method	XT 3 13
Injection Volume:	10.00 ul	Channel Name:	254.0nm
Run Time:	60.0 Minutes	Proc. Chnl. Descr.:	2998 PDA 254.0 nm (2998
Date Acquired:	12/2/2021 10:13:14 AM CST		
Date Processed:	12/2/2021 10:49:18 AM CST		

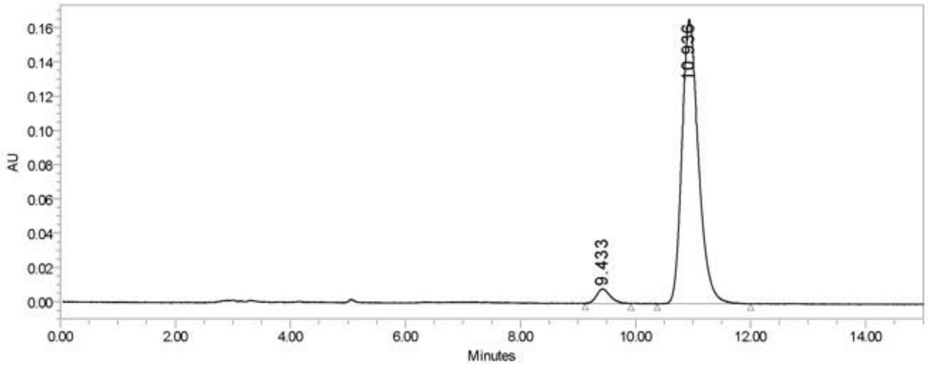

	RT	Area	% Area	Height
1	9.433	139566	4.06	8442
2	10.936	3300934	95.94	166038

Supplementary Figure 89. HPLC spectra of compound **4j**

Rac-**4k**

SAMPLE INFORMATION

Sample Name:	xt-2-174-RAC-20%-IG	Acquired By:	System
Sample Type:	Unknown	Sample Set Name	
Vial:	45	Acq. Method Set:	20% quanbo
Injection #:	1	Processing Method	XT 2 174 RAC
Injection Volume:	10.00 ul	Channel Name:	254.0nm
Run Time:	60.0 Minutes	Proc. Chnl. Descr.:	2998 PDA 254.0 nm (2998
Date Acquired:	10/31/2021 3:04:35 PM CST		
Date Processed:	4/22/2022 10:58:02 PM CST		

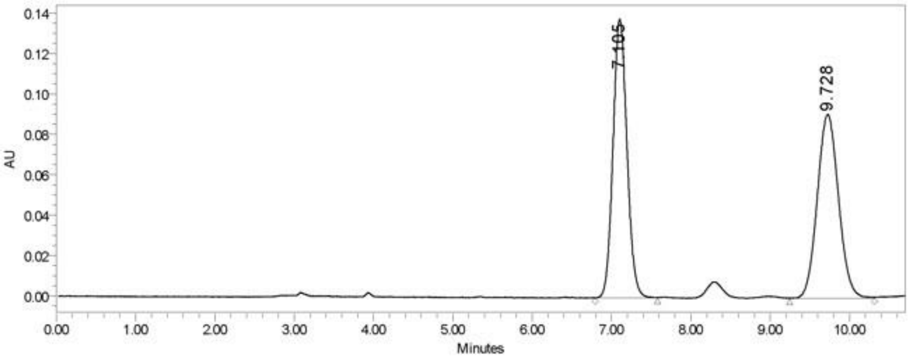

	RT	Area	% Area	Height
1	7.105	1682765	49.90	137953
2	9.728	1689325	50.10	91127

Asy-**4k**

SAMPLE INFORMATION

Sample Name:	xt-2-175-20%-IG	Acquired By:	System
Sample Type:	Unknown	Sample Set Name	
Vial:	55	Acq. Method Set:	20% quanbo
Injection #:	1	Processing Method	XT 2 175 ASY
Injection Volume:	10.00 ul	Channel Name:	254.0nm
Run Time:	60.0 Minutes	Proc. Chnl. Descr.:	2998 PDA 254.0 nm (2998
Date Acquired:	10/31/2021 3:25:54 PM CST		
Date Processed:	4/22/2022 11:01:38 PM CST		

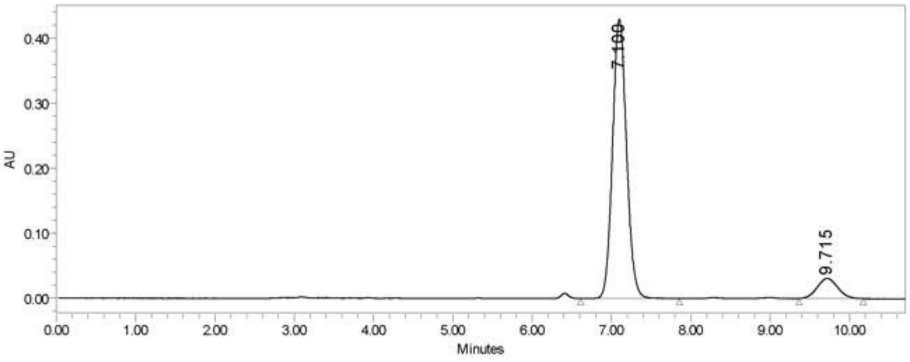

	RT	Area	% Area	Height
1	7.100	5234480	90.27	429416
2	9.715	564523	9.73	31002

Supplementary Figure 90. HPLC spectra of compound **4k**

Rac-**4l**

SAMPLE INFORMATION

Sample Name:	xt-3-22-rac-20%-AD	Acquired By:	System
Sample Type:	Unknown	Sample Set Name	
Vial:	116	Acq. Method Set:	20% quanbo
Injection #:	1	Processing Method	XT 3 22 RAC
Injection Volume:	10.00 ul	Channel Name:	254.0nm
Run Time:	60.0 Minutes	Proc. Chnl. Descr.:	2998 PDA 254.0 nm (2998
Date Acquired:	12/20/2021 3:45:43 PM CST		
Date Processed:	5/5/2022 9:50:10 PM CST		

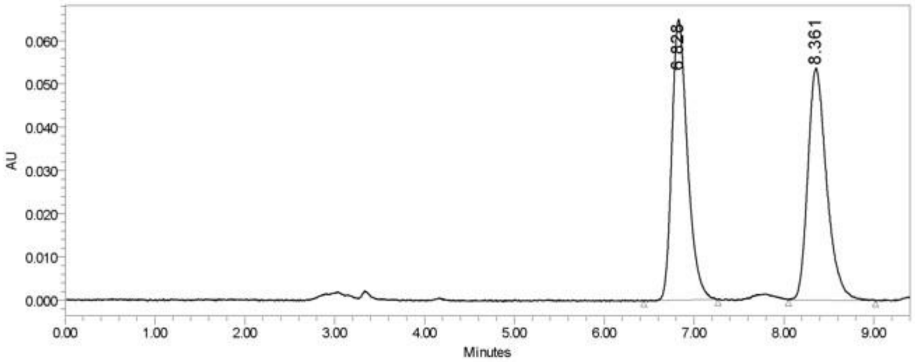

	RT	Area	% Area	Height
1	6.828	784361	50.11	64854
2	8.361	780859	49.89	53615

Asy-**4l**

SAMPLE INFORMATION

Sample Name:	xt-3-23-20%-AD	Acquired By:	System
Sample Type:	Unknown	Sample Set Name	
Vial:	17	Acq. Method Set:	20% quanbo
Injection #:	1	Processing Method	XT 3 23 RAC
Injection Volume:	10.00 ul	Channel Name:	254.0nm
Run Time:	60.0 Minutes	Proc. Chnl. Descr.:	2998 PDA 254.0 nm (2998
Date Acquired:	12/20/2021 4:07:59 PM CST		
Date Processed:	5/5/2022 9:51:40 PM CST		

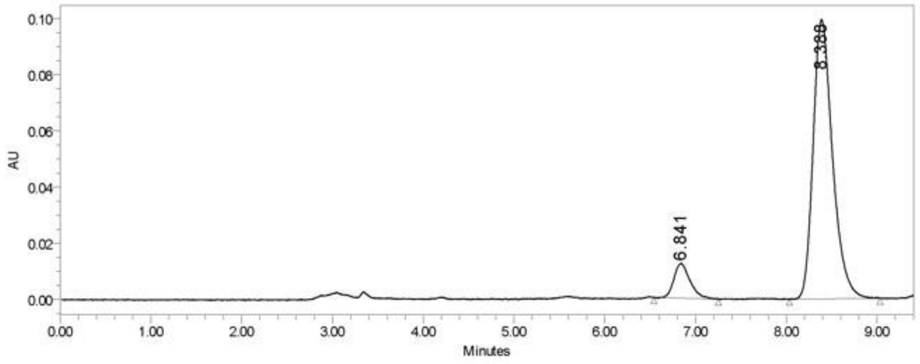

	RT	Area	% Area	Height
1	6.841	150698	9.31	12300
2	8.388	1467592	90.69	99377

Supplementary Figure 91. HPLC spectra of compound **4l**

Rac-**4m**

SAMPLE INFORMATION

Sample Name:	xt-3-55-rac-20%-IG	Acquired By:	System
Sample Type:	Unknown	Sample Set Name:	
Vial:	41	Acq. Method Set:	20%qb
Injection #:	1	Processing Method:	XT 3 55 RAC
Injection Volume:	10.00 ul	Channel Name:	254.0nm
Run Time:	60.0 Minutes	Proc. Chnl. Descr.:	2998 PDA 254.0 nm (2998
Date Acquired:	1/5/2022 4:42:37 PM CST		
Date Processed:	1/5/2022 7:17:57 PM CST		

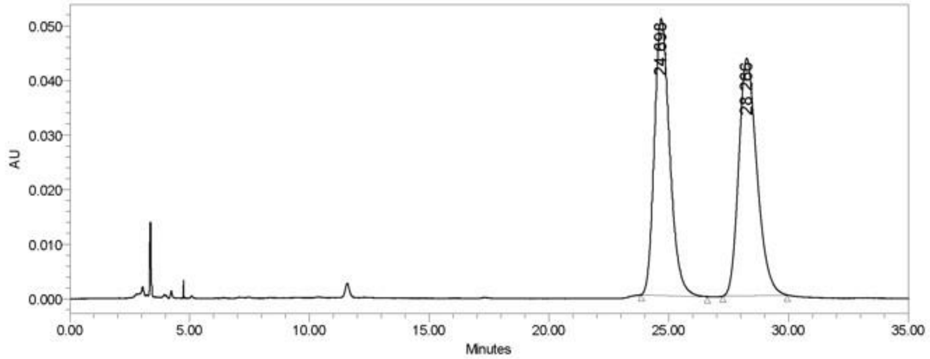

	RT	Area	% Area	Height
1	24.698	2276984	49.71	50669
2	28.266	2303228	50.29	43504

Asy-**4m**

SAMPLE INFORMATION

Sample Name:	XT-3-56-20%-IG	Acquired By:	System
Sample Type:	Unknown	Sample Set Name:	0105
Vial:	39	Acq. Method Set:	20%qb
Injection #:	1	Processing Method:	XT 3 56
Injection Volume:	10.00 ul	Channel Name:	254.0nm
Run Time:	35.0 Minutes	Proc. Chnl. Descr.:	2998 PDA 254.0 nm (2998
Date Acquired:	1/5/2022 5:21:07 PM CST		
Date Processed:	1/5/2022 7:21:21 PM CST		

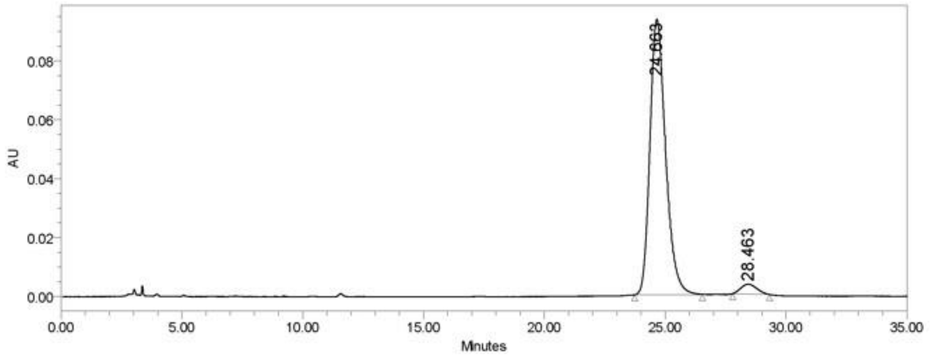

	RT	Area	% Area	Height
1	24.663	4216832	96.48	93527
2	28.463	153674	3.52	3340

Supplementary Figure 92. HPLC spectra of compound **4m**

Rac-**4n**

SAMPLE INFORMATION

Sample Name:	xt-3-118-RAC-20%-AD	Acquired By:	System
Sample Type:	Unknown	Sample Set Name	
Vial:	102	Acq. Method Set:	20% quanbo
Injection #:	1	Processing Method	XT 3 67 RAC
Injection Volume:	10.00 ul	Channel Name:	254.0nm
Run Time:	60.0 Minutes	Proc. Chnl. Descr.:	2998 PDA 254.0 nm (2998
Date Acquired:	5/16/2022 10:03:01 PM CST		
Date Processed:	5/16/2022 10:36:18 PM CST		

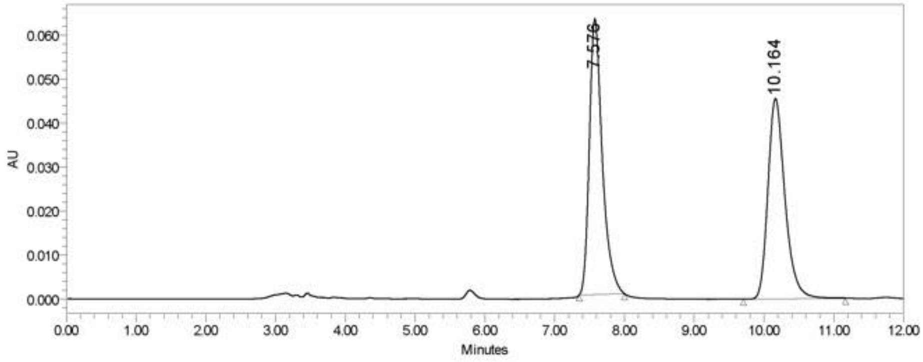

	RT	Area	% Area	Height
1	7.576	806014	51.02	62677
2	10.164	773847	48.98	45558

Asy-**4n**

SAMPLE INFORMATION

Sample Name:	xt-3-118-asy-20%-AD	Acquired By:	System
Sample Type:	Unknown	Sample Set Name	
Vial:	94	Acq. Method Set:	20% quanbo
Injection #:	1	Processing Method	XT 3 67 ASY
Injection Volume:	10.00 ul	Channel Name:	254.0nm
Run Time:	60.0 Minutes	Proc. Chnl. Descr.:	2998 PDA 254.0 nm (2998
Date Acquired:	5/16/2022 9:43:32 PM CST		
Date Processed:	5/16/2022 10:30:00 PM CST		

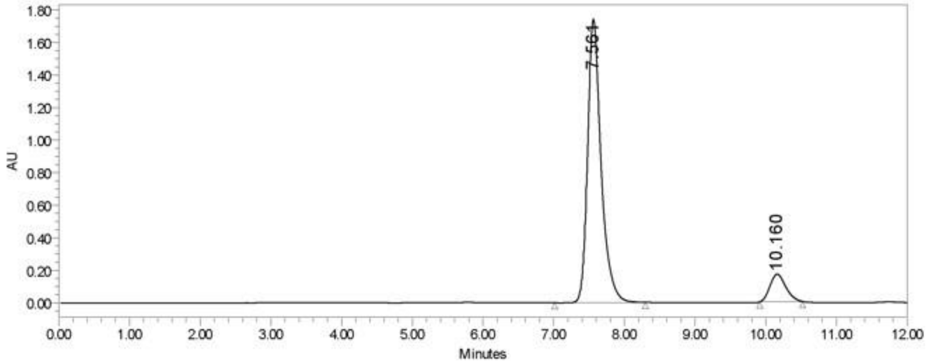

	RT	Area	% Area	Height
1	7.561	23033819	89.75	1742933
2	10.160	2631074	10.25	168831

Supplementary Figure 93. HPLC spectra of compound **4n**

Rac-**4o**

SAMPLE INFORMATION

Sample Name:	xt-3-160-1-rac-10%-AD	Acquired By:	System
Sample Type:	Unknown	Sample Set Name	
Vial:	88	Acq. Method Set:	10% quanbo
Injection #:	1	Processing Method	XT 3 160 1 RAC
Injection Volume:	10.00 ul	Channel Name:	254.0nm
Run Time:	60.0 Minutes	Proc. Chnl. Descr.:	2998 PDA 254.0 nm (2998
Date Acquired:	8/23/2022 3:35:53 PM CST		
Date Processed:	10/20/2022 11:31:16 AM CST		

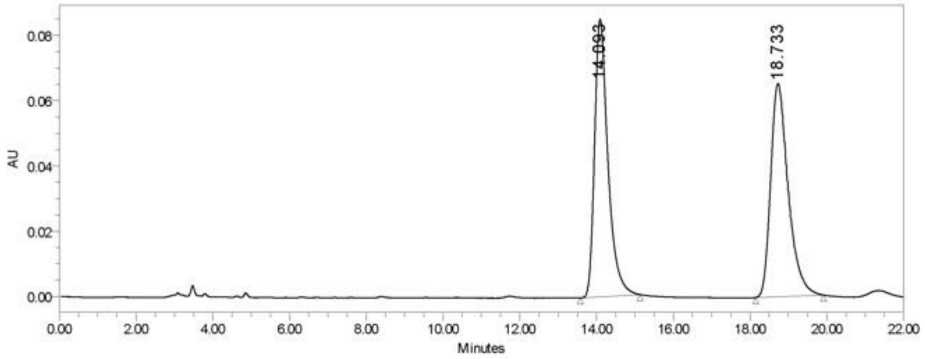

	RT	Area	% Area	Height
1	14.093	2067758	49.98	85083
2	18.733	2069812	50.02	65377

Asy-**4o**

SAMPLE INFORMATION

Sample Name:	xt-3-160-1-asy-10%-AD	Acquired By:	System
Sample Type:	Unknown	Sample Set Name	
Vial:	14	Acq. Method Set:	10% quanbo
Injection #:	1	Processing Method	xt 3 160 1 asy
Injection Volume:	10.00 ul	Channel Name:	254.0nm
Run Time:	60.0 Minutes	Proc. Chnl. Descr.:	2998 PDA 254.0 nm (2998
Date Acquired:	8/24/2022 11:08:41 AM CST		
Date Processed:	10/20/2022 11:29:15 AM CST		

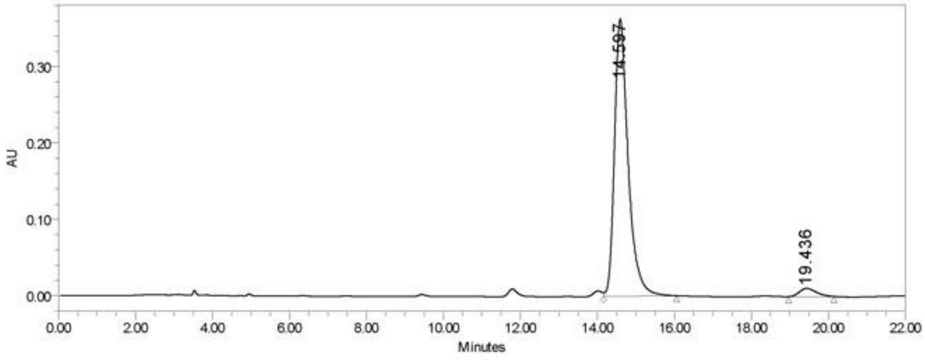

	RT	Area	% Area	Height
1	14.597	8978001	96.41	363494
2	19.436	334362	3.59	10883

Supplementary Figure 94. HPLC spectra of compound **4o**

Rac-**5a**

SAMPLE INFORMATION

Sample Name:	xt-3a-rac-30%-AD	Acquired By:	System
Sample Type:	Unknown	Sample Set Name:	
Vial:	36	Acq. Method Set:	30%qb
Injection #:	1	Processing Method:	xt 3a rac
Injection Volume:	10.00 ul	Channel Name:	254.0nm
Run Time:	60.0 Minutes	Proc. Chnl. Descr.:	2998 PDA 254.0 nm (2998
Date Acquired:	10/9/2022 8:04:48 PM CST		
Date Processed:	10/20/2022 11:19:43 AM CST		

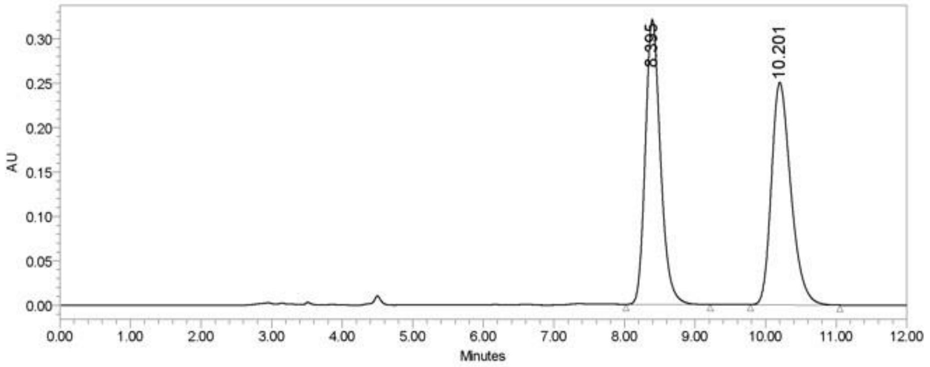

	RT	Area	% Area	Height
1	8.395	4814708	50.03	320735
2	10.201	4809608	49.97	250259

Asy-**5a**

SAMPLE INFORMATION

Sample Name:	xt-3a-asy-30%-AD	Acquired By:	System
Sample Type:	Unknown	Sample Set Name:	
Vial:	73	Acq. Method Set:	30%qb
Injection #:	1	Processing Method:	XT 3A ASY
Injection Volume:	10.00 ul	Channel Name:	254.0nm
Run Time:	60.0 Minutes	Proc. Chnl. Descr.:	2998 PDA 254.0 nm (2998
Date Acquired:	10/9/2022 11:29:43 AM CST		
Date Processed:	10/20/2022 11:18:24 AM CST		

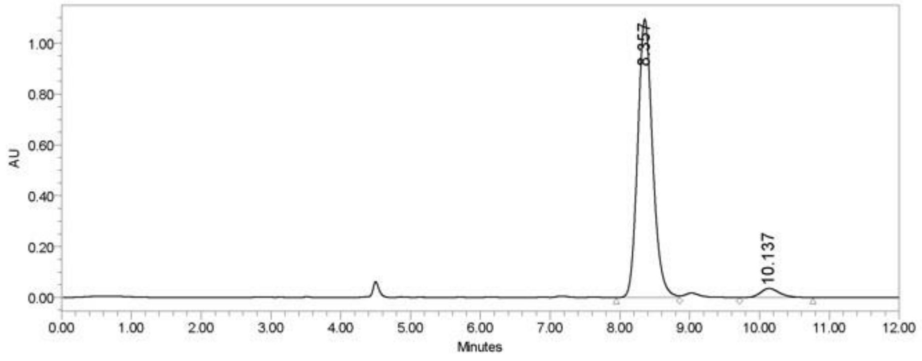

	RT	Area	% Area	Height
1	8.357	16304381	96.04	1094434
2	10.137	672650	3.96	35667

Supplementary Figure 95. HPLC spectra of compound **5a**

Rac-**5b**

SAMPLE INFORMATION

Sample Name:	XT-2-188-RAC-30%-AD	Acquired By:	System
Sample Type:	Unknown	Sample Set Name	1119
Vial:	87	Acq. Method Set:	30%quanbo
Injection #:	1	Processing Method	xt 2 188 rac
Injection Volume:	10.00 ul	Channel Name:	254.0nm
Run Time:	15.0 Minutes	Proc. Chnl. Descr.:	2998 PDA 254.0 nm (2998
Date Acquired:	11/19/2021 3:11:54 PM CST		
Date Processed:	11/19/2021 3:30:05 PM CST		

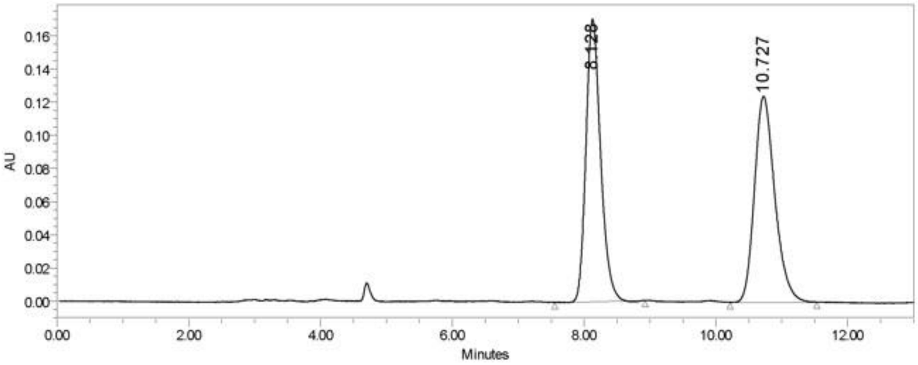

	RT	Area	% Area	Height
1	8.128	2641884	49.60	170382
2	10.727	2684770	50.40	124272

Asy-**5b**

SAMPLE INFORMATION

Sample Name:	xt-2-195-30%-AD	Acquired By:	System
Sample Type:	Unknown	Sample Set Name	1119
Vial:	37	Acq. Method Set:	30%quanbo
Injection #:	1	Processing Method	XT 2 195
Injection Volume:	10.00 ul	Channel Name:	254.0nm
Run Time:	15.0 Minutes	Proc. Chnl. Descr.:	2998 PDA 254.0 nm (2998
Date Acquired:	11/19/2021 11:40:50 AM CST		
Date Processed:	11/19/2021 3:35:55 PM CST		

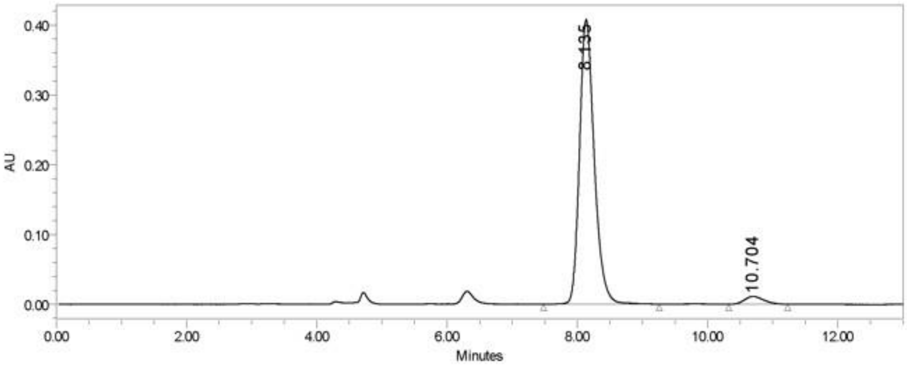

	RT	Area	% Area	Height
1	8.135	6411142	96.55	407574
2	10.704	229289	3.45	11098

Supplementary Figure 96. HPLC spectra of compound **5b**

Rac-**5c**

SAMPLE INFORMATION

Sample Name:	xt-2-187-rac-30%-AD	Acquired By:	System
Sample Type:	Unknown	Sample Set Name	1
Vial:	31	Acq. Method Set:	30%quanbo
Injection #:	1	Processing Method	XT 2 187 RAC
Injection Volume:	10.00 ul	Channel Name:	254.0nm
Run Time:	15.0 Minutes	Proc. Chnl. Descr.:	2998 PDA 254.0 nm (2998
Date Acquired:	11/24/2021 5:09:05 PM CST		
Date Processed:	11/24/2021 6:31:45 PM CST		

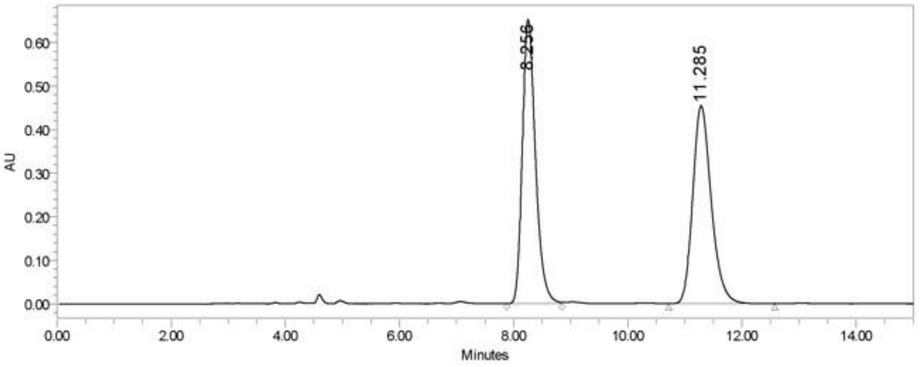

	RT	Area	% Area	Height
1	8.256	10354501	50.05	652372
2	11.285	10333279	49.95	454685

Asy-**5c**

SAMPLE INFORMATION

Sample Name:	xt-3-4-30%-AD	Acquired By:	System
Sample Type:	Unknown	Sample Set Name	
Vial:	89	Acq. Method Set:	30%quanbo
Injection #:	1	Processing Method	xt 3 4
Injection Volume:	10.00 ul	Channel Name:	254.0nm
Run Time:	30.0 Minutes	Proc. Chnl. Descr.:	2998 PDA 254.0 nm (2998
Date Acquired:	11/24/2021 2:31:14 PM CST		
Date Processed:	11/24/2021 5:21:10 PM CST		

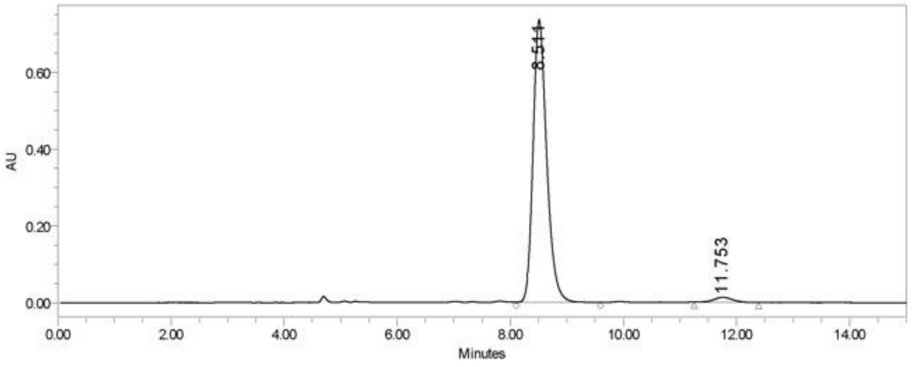

	RT	Area	% Area	Height
1	8.511	12457912	97.62	736625
2	11.753	303624	2.38	13085

Supplementary Figure 97. HPLC spectra of compound **5c**

Rac-**5d**

SAMPLE INFORMATION

Sample Name:	xt-2-185-20%-AD	Acquired By:	System
Sample Type:	Unknown	Sample Set Name	
Vial:	92	Acq. Method Set:	20% quanbo
Injection #:	1	Processing Method	XT 2 185 RAC
Injection Volume:	10.00 ul	Channel Name:	254.0nm
Run Time:	60.0 Minutes	Proc. Chnl. Descr.:	2998 PDA 254.0 nm (2998
Date Acquired:	12/6/2021 9:46:53 PM CST		
Date Processed:	12/7/2021 2:37:36 PM CST		

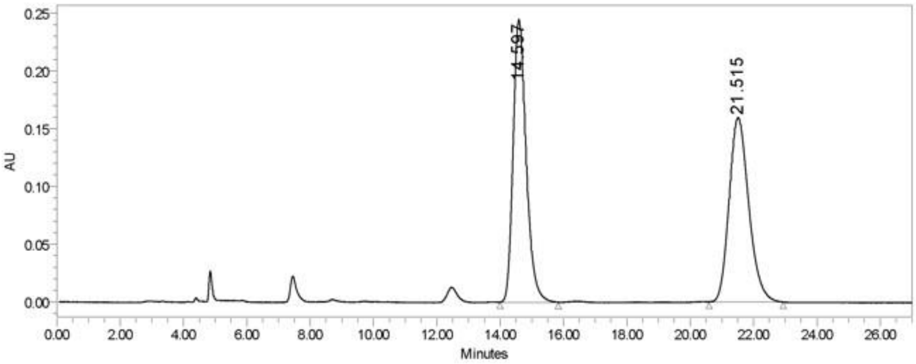

	RT	Area	% Area	Height
1	14.597	6958240	50.34	244832
2	21.515	6863471	49.66	159736

Asy-**5d**

SAMPLE INFORMATION

Sample Name:	xt-3-19-20%-AD	Acquired By:	System
Sample Type:	Unknown	Sample Set Name	1207
Vial:	10	Acq. Method Set:	20% quanbo
Injection #:	1	Processing Method	XT 3 19
Injection Volume:	10.00 ul	Channel Name:	254.0nm
Run Time:	30.0 Minutes	Proc. Chnl. Descr.:	2998 PDA 254.0 nm (2998
Date Acquired:	12/7/2021 11:45:48 AM CST		
Date Processed:	12/7/2021 2:39:32 PM CST		

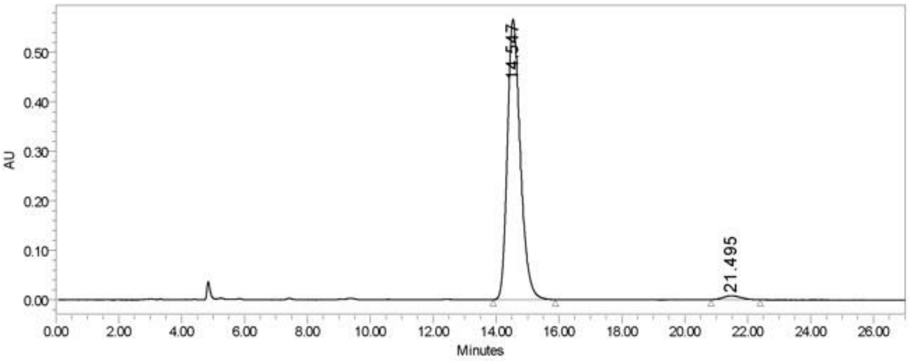

	RT	Area	% Area	Height
1	14.547	16135648	98.01	566359
2	21.495	326836	1.99	8127

Supplementary Figure 98. HPLC spectra of compound **5d**

Rac-**5e**

SAMPLE INFORMATION

Sample Name:	XT-3-10-20%-AD	Acquired By:	System
Sample Type:	Unknown	Sample Set Name:	1207
Vial:	70	Acq. Method Set:	20%qb
Injection #:	1	Processing Method:	XT 3 10 RAC
Injection Volume:	10.00 ul	Channel Name:	254.0nm
Run Time:	16.0 Minutes	Proc. Chnl. Descr.:	2998 PDA 254.0 nm (2998
Date Acquired:	12/7/2021 11:20:24 AM CST		
Date Processed:	12/7/2021 2:43:31 PM CST		

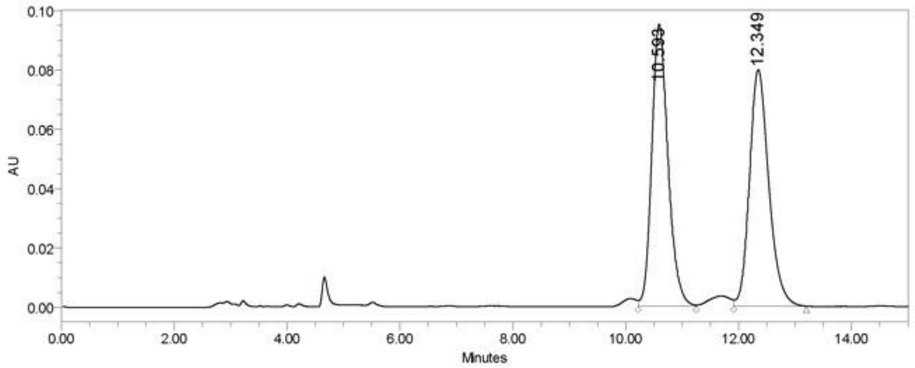

	RT	Area	% Area	Height
1	10.593	1898237	50.05	95082
2	12.349	1894801	49.95	79754

Asy-**5e**

SAMPLE INFORMATION

Sample Name:	xt-3-18-20%-AD	Acquired By:	System
Sample Type:	Unknown	Sample Set Name:	
Vial:	35	Acq. Method Set:	20%qb
Injection #:	1	Processing Method:	XT 3 18
Injection Volume:	10.00 ul	Channel Name:	254.0nm
Run Time:	60.0 Minutes	Proc. Chnl. Descr.:	2998 PDA 254.0 nm (2998
Date Acquired:	12/7/2021 10:58:19 AM CST		
Date Processed:	12/7/2021 2:45:28 PM CST		

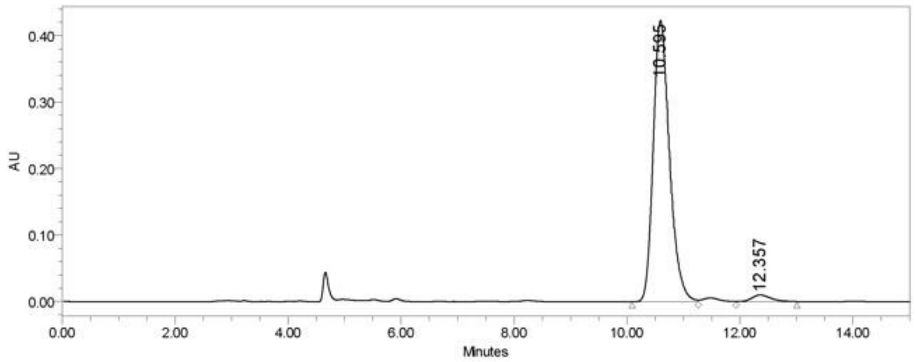

	RT	Area	% Area	Height
1	10.595	8458518	97.29	422855
2	12.357	235784	2.71	10067

Supplementary Figure 99. HPLC spectra of compound **5e**

Rac-**5f**

SAMPLE INFORMATION

Sample Name:	xt-3-105-RAC-30%-AD	Acquired By:	System
Sample Type:	Unknown	Sample Set Name:	
Vial:	43	Acq. Method Set:	30%qb
Injection #:	1	Processing Method:	xt 3 105 rac
Injection Volume:	10.00 ul	Channel Name:	254.0nm
Run Time:	60.0 Minutes	Proc. Chnl. Descr.:	2998 PDA 254.0 nm (2998
Date Acquired:	4/18/2022 9:28:03 PM CST		
Date Processed:	4/19/2022 10:38:33 PM CST		

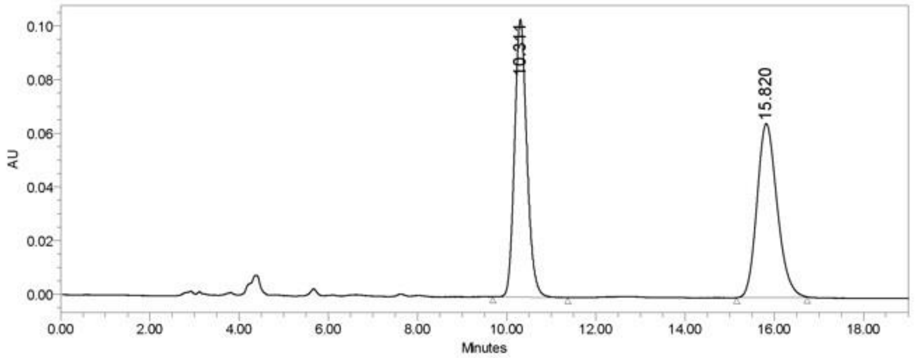

	RT	Area	% Area	Height
1	10.311	1987468	49.32	103496
2	15.820	2042588	50.68	64825

Asy-**5f**

SAMPLE INFORMATION

Sample Name:	xt-3-105-ASY-30%-AD	Acquired By:	System
Sample Type:	Unknown	Sample Set Name:	
Vial:	78	Acq. Method Set:	30%qb
Injection #:	1	Processing Method:	XT 3 105 ASY
Injection Volume:	10.00 ul	Channel Name:	254.0nm
Run Time:	60.0 Minutes	Proc. Chnl. Descr.:	2998 PDA 254.0 nm (2998
Date Acquired:	4/18/2022 10:48:23 PM CST		
Date Processed:	4/19/2022 10:40:14 PM CST		

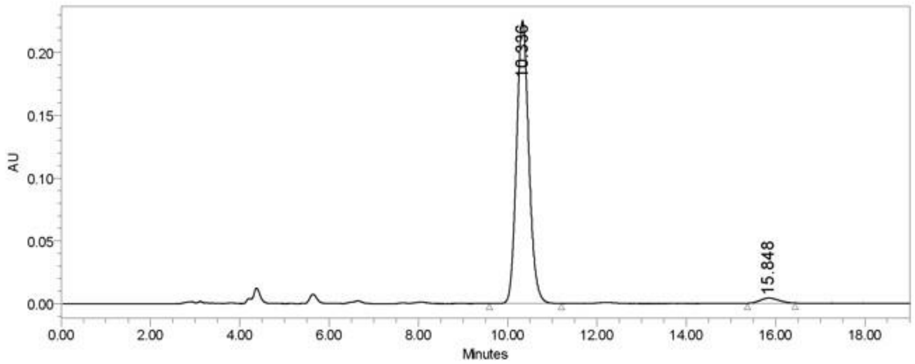

	RT	Area	% Area	Height
1	10.336	4316485	97.28	225431
2	15.848	120527	2.72	4136

Supplementary Figure 100. HPLC spectra of compound **5f**

Rac-**5g**

SAMPLE INFORMATION

Sample Name:	xt-3-91-rac-10%-AD	Acquired By:	System
Sample Type:	Unknown	Sample Set Name:	
Vial:	72	Acq. Method Set:	10%qb
Injection #:	1	Processing Method:	xt 3 91 rac
Injection Volume:	10.00 ul	Channel Name:	272.0nm
Run Time:	60.0 Minutes	Proc. Chnl. Descr.:	2998 PDA 272.0 nm (2998
Date Acquired:	4/20/2022 11:37:19 AM CST		
Date Processed:	4/20/2022 3:38:19 PM CST		

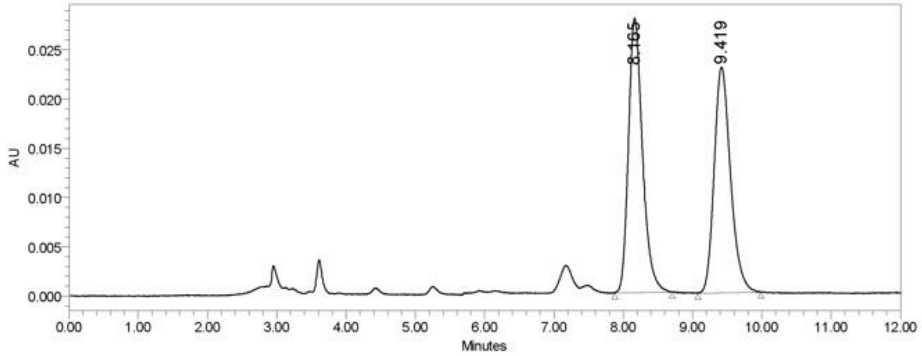

	RT	Area	% Area	Height
1	8.165	393054	51.13	27851
2	9.419	375660	48.87	22906

Asy-**5g**

SAMPLE INFORMATION

Sample Name:	xt-3-91-ASY-10%-AD	Acquired By:	System
Sample Type:	Unknown	Sample Set Name:	
Vial:	26	Acq. Method Set:	10%qb
Injection #:	1	Processing Method:	XT 3 91 ASY
Injection Volume:	10.00 ul	Channel Name:	272.0nm
Run Time:	60.0 Minutes	Proc. Chnl. Descr.:	2998 PDA 272.0 nm (2998
Date Acquired:	10/12/2022 4:10:26 PM CST		
Date Processed:	10/20/2022 11:16:51 AM CST		

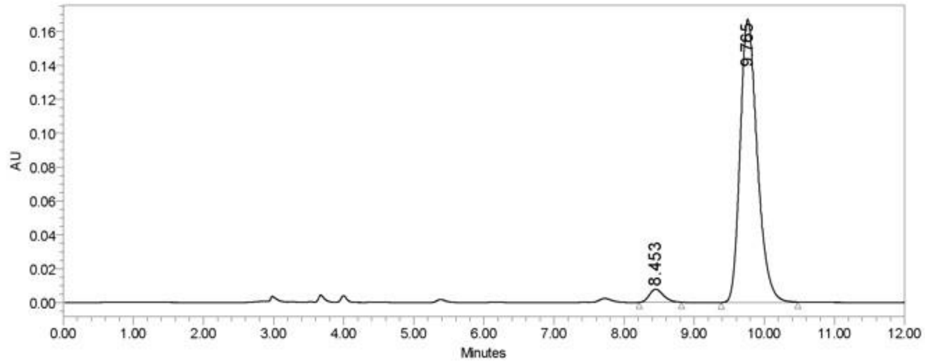

	RT	Area	% Area	Height
1	8.453	108750	3.69	7734
2	9.765	2836021	96.31	167037

Supplementary Figure 101. HPLC spectra of compound **5g**

Rac-**5h**

SAMPLE INFORMATION

Sample Name:	xt-3-99-rac-20%-IG	Acquired By:	System
Sample Type:	Unknown	Sample Set Name:	
Vial:	8	Acq. Method Set:	20%qb
Injection #:	1	Processing Method:	XT 3 99 RAC
Injection Volume:	10.00 ul	Channel Name:	255.0nm
Run Time:	60.0 Minutes	Proc. Chnl. Descr.:	2998 PDA 255.0 nm (2998
Date Acquired:	3/31/2022 9:50:13 PM CST		
Date Processed:	4/1/2022 3:06:19 PM CST		

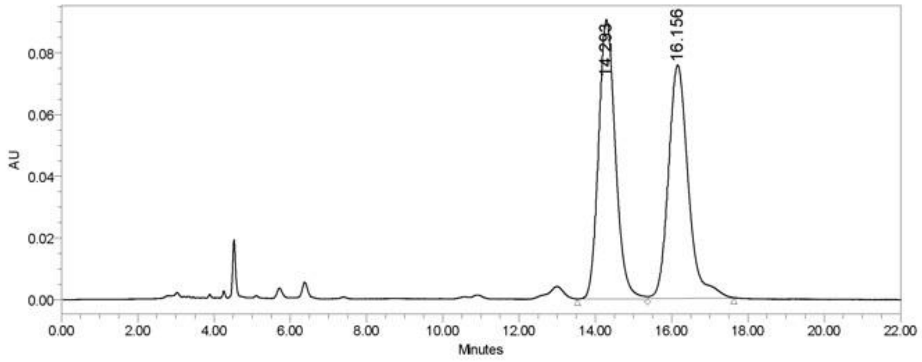

	RT	Area	% Area	Height
1	14.293	2766376	50.40	90523
2	16.156	2722433	49.60	75633

Asy-**5h**

SAMPLE INFORMATION

Sample Name:	xt-3-99-asy-20%-IG	Acquired By:	System
Sample Type:	Unknown	Sample Set Name:	
Vial:	33	Acq. Method Set:	20%qb
Injection #:	1	Processing Method:	XT 3 99 ASY
Injection Volume:	10.00 ul	Channel Name:	255.0nm
Run Time:	60.0 Minutes	Proc. Chnl. Descr.:	2998 PDA 255.0 nm (2998
Date Acquired:	3/31/2022 10:15:34 PM CST		
Date Processed:	4/1/2022 3:08:20 PM CST		

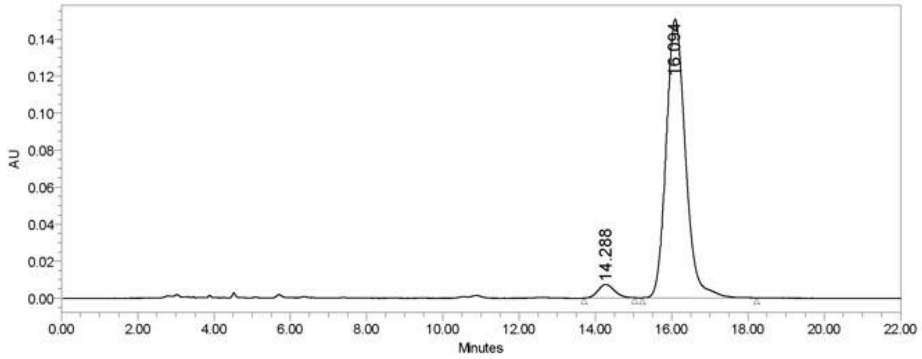

	RT	Area	% Area	Height
1	14.288	219435	4.01	7296
2	16.094	5258346	95.99	150478

Supplementary Figure 102. HPLC spectra of compound **5h**

Rac-**5i**

SAMPLE INFORMATION

Sample Name:	xt-3-103-rac-20%-IG	Acquired By:	System
Sample Type:	Unknown	Sample Set Name	
Vial:	20	Acq. Method Set:	20% quanbo
Injection #:	1	Processing Method	XT 3 103 RAC
Injection Volume:	10.00 ul	Channel Name:	254.0nm
Run Time:	60.0 Minutes	Proc. Chnl. Descr.:	2998 PDA 254.0 nm (2998
Date Acquired:	4/9/2022 9:18:51 AM CST		
Date Processed:	4/10/2022 4:38:43 PM CST		

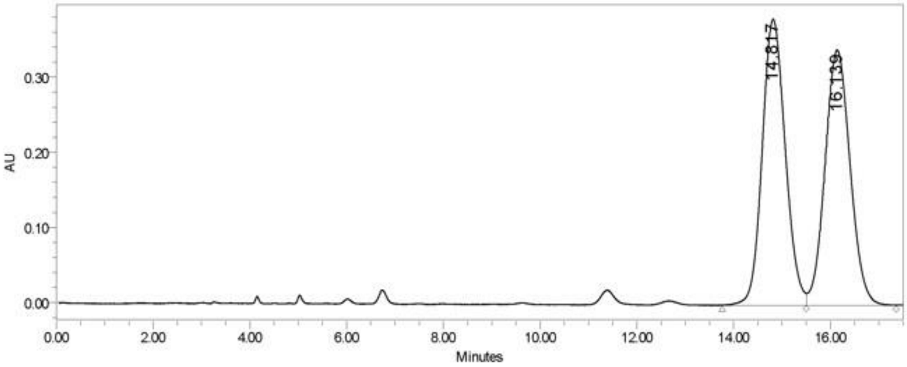

	RT	Area	% Area	Height
1	14.817	12554513	50.59	380660
2	16.139	12261048	49.41	339875

Asy-**5i**

SAMPLE INFORMATION

Sample Name:	xt-3-103-asy-20%-IG	Acquired By:	System
Sample Type:	Unknown	Sample Set Name	
Vial:	32	Acq. Method Set:	20% quanbo
Injection #:	1	Processing Method	XT 3 103 ASY
Injection Volume:	10.00 ul	Channel Name:	254.0nm
Run Time:	60.0 Minutes	Proc. Chnl. Descr.:	2998 PDA 254.0 nm (2998
Date Acquired:	4/9/2022 9:45:35 AM CST		
Date Processed:	4/10/2022 4:36:29 PM CST		

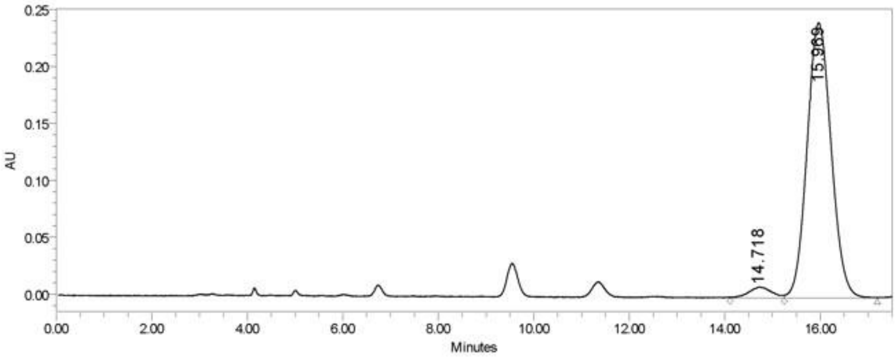

	RT	Area	% Area	Height
1	14.718	315019	3.61	9543
2	15.969	8418233	96.39	241341

Supplementary Figure 103. HPLC spectra of compound **5i**

Rac-**7a**

SAMPLE INFORMATION

Sample Name:	xt-3-153-3-rac-20%-IA	Acquired By:	System
Sample Type:	Unknown	Sample Set Name	
Vial:	20	Acq. Method Set:	20% quanbo
Injection #:	1	Processing Method	XT 3 153 3 RAC
Injection Volume:	10.00 ul	Channel Name:	254.0nm
Run Time:	60.0 Minutes	Proc. Chnl. Descr.:	2998 PDA 254.0 nm (2998
Date Acquired:	7/28/2022 3:45:37 PM CST		
Date Processed:	9/24/2022 10:20:57 PM CST		

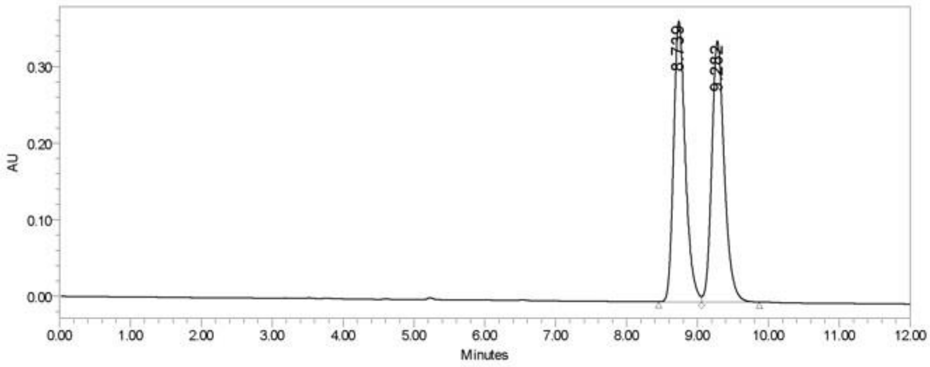

	RT	Area	% Area	Height
1	8.739	4198081	49.86	366819
2	9.282	4221651	50.14	341014

Asy-**7a**

SAMPLE INFORMATION

Sample Name:	xt-3-153-3-ASY-20%-IA	Acquired By:	System
Sample Type:	Unknown	Sample Set Name	
Vial:	50	Acq. Method Set:	20% quanbo
Injection #:	1	Processing Method	XT 3 153 3
Injection Volume:	10.00 ul	Channel Name:	254.0nm
Run Time:	60.0 Minutes	Proc. Chnl. Descr.:	2998 PDA 254.0 nm (2998
Date Acquired:	7/28/2022 4:05:15 PM CST		
Date Processed:	9/24/2022 10:19:09 PM CST		

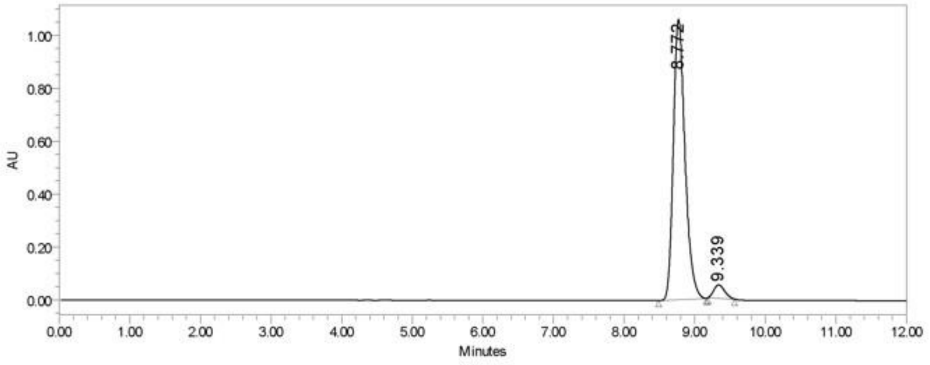

	RT	Area	% Area	Height
1	8.772	12089275	95.75	1059478
2	9.339	535956	4.25	50405

Supplementary Figure 104. HPLC spectra of compound **7a**

Rac-**7b**

SAMPLE INFORMATION

Sample Name:	xt-3-153-4-RAC-20%-IF	Acquired By:	System
Sample Type:	Unknown	Sample Set Name:	
Vial:	104	Acq. Method Set:	20%qb
Injection #:	1	Processing Method:	XT 3 154 4 RAC
Injection Volume:	10.00 ul	Channel Name:	254.0nm
Run Time:	60.0 Minutes	Proc. Chnl. Descr.:	2998 PDA 254.0 nm (2998
Date Acquired:	7/29/2022 4:14:43 PM CST		
Date Processed:	7/29/2022 4:52:39 PM CST		

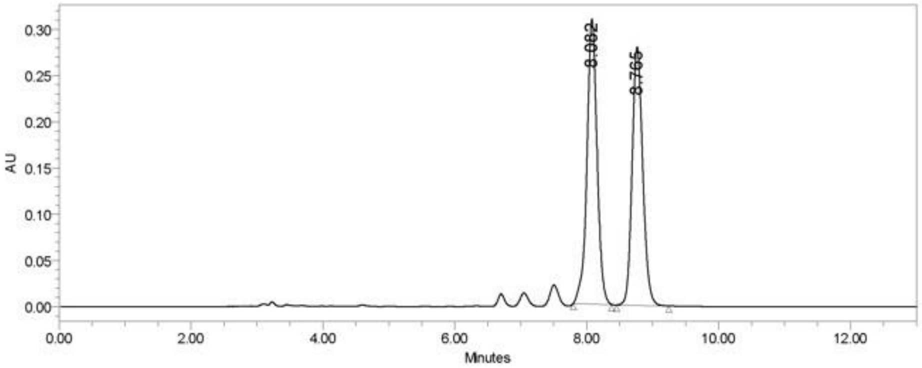

	RT	Area	% Area	Height
1	8.082	3296466	51.23	307849
2	8.765	3137834	48.77	279656

Asy-**7b**

SAMPLE INFORMATION

Sample Name:	xt-3-153-4-ASY-20%-IF	Acquired By:	System
Sample Type:	Unknown	Sample Set Name:	
Vial:	22	Acq. Method Set:	20%qb
Injection #:	1	Processing Method:	XT 3 153 4 ASY
Injection Volume:	10.00 ul	Channel Name:	254.0nm
Run Time:	60.0 Minutes	Proc. Chnl. Descr.:	2998 PDA 254.0 nm (2998
Date Acquired:	7/29/2022 4:33:55 PM CST		
Date Processed:	7/29/2022 4:50:46 PM CST		

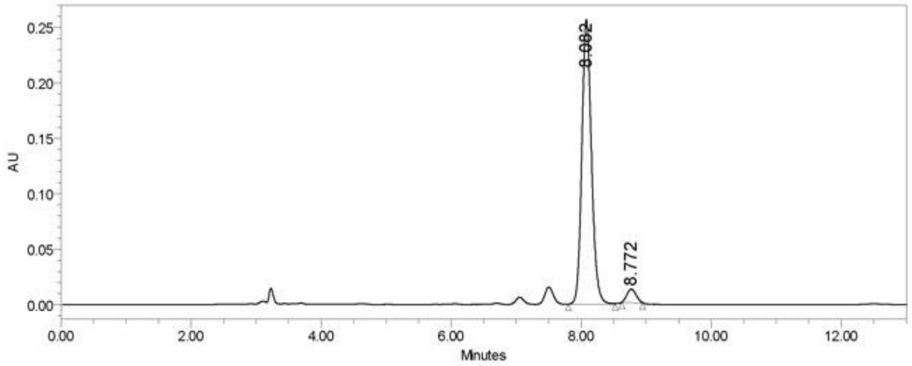

	RT	Area	% Area	Height
1	8.082	2634525	95.80	256544
2	8.772	115364	4.20	11976

Supplementary Figure 105. HPLC spectra of compound **7b**

Rac-**7c**

SAMPLE INFORMATION

Sample Name:	xt-3-153-1-rac-20%-IF	Acquired By:	System
Sample Type:	Unknown	Sample Set Name	
Vial:	106	Acq. Method Set:	20% quanbo
Injection #:	1	Processing Method	XT 3 153 1 RAC
Injection Volume:	10.00 ul	Channel Name:	256.0nm
Run Time:	60.0 Minutes	Proc. Chnl. Descr.:	2998 PDA 256.0 nm (2998
Date Acquired:	7/26/2022 8:09:53 PM CST		
Date Processed:	7/26/2022 8:40:30 PM CST		

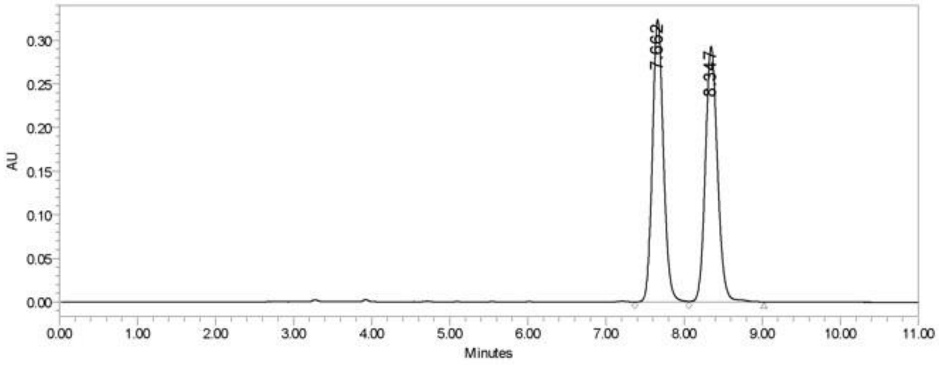

	RT	Area	% Area	Height
1	7.662	3176039	49.94	323927
2	8.347	3184283	50.06	293145

Asy-**7c**

SAMPLE INFORMATION

Sample Name:	xt-3-153-1-ASY-20%-IF	Acquired By:	System
Sample Type:	Unknown	Sample Set Name	
Vial:	8	Acq. Method Set:	20% quanbo
Injection #:	1	Processing Method	XT 3 153 1 ASY
Injection Volume:	10.00 ul	Channel Name:	256.0nm
Run Time:	60.0 Minutes	Proc. Chnl. Descr.:	2998 PDA 256.0 nm (2998
Date Acquired:	7/26/2022 8:25:54 PM CST		
Date Processed:	7/26/2022 8:43:15 PM CST		

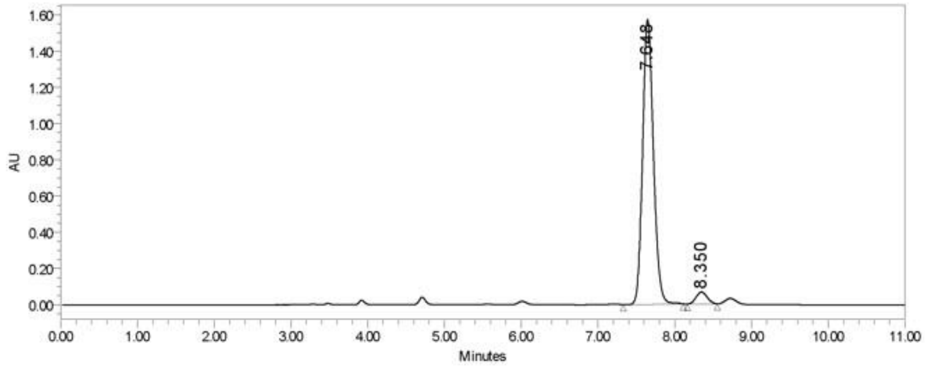

	RT	Area	% Area	Height
1	7.648	15375436	95.85	1571984
2	8.350	665052	4.15	66029

Supplementary Figure 106. HPLC spectra of compound **7c**

Rac-**7d**

SAMPLE INFORMATION

Sample Name:	xt-3-153-2-RAC-20%-AD	Acquired By:	System
Sample Type:	Unknown	Sample Set Name:	
Vial:	37	Acq. Method Set:	20%qb
Injection #:	1	Processing Method:	xt 3 153 2 rac
Injection Volume:	10.00 ul	Channel Name:	266.0nm
Run Time:	60.0 Minutes	Proc. Chnl. Descr.:	2998 PDA 266.0 nm (2998
Date Acquired:	7/27/2022 8:06:23 PM CST		
Date Processed:	7/27/2022 10:50:52 PM CST		

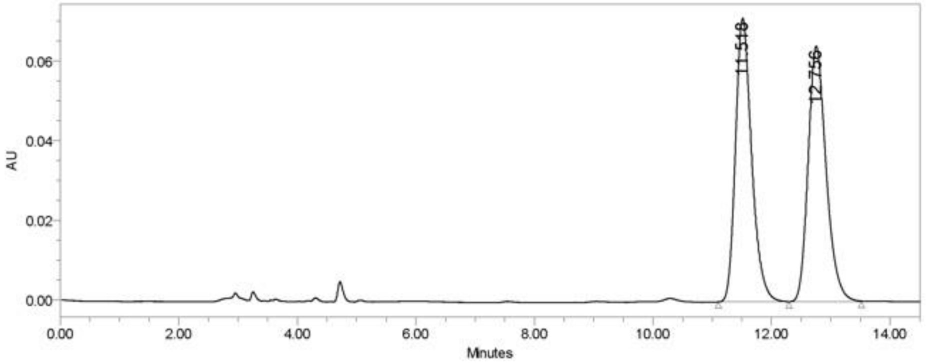

	RT	Area	% Area	Height
1	11.518	1350388	50.08	71219
2	12.756	1346258	49.92	64075

Asy-**7d**

SAMPLE INFORMATION

Sample Name:	xt-3-153-2-asy-20%-AD	Acquired By:	System
Sample Type:	Unknown	Sample Set Name:	
Vial:	93	Acq. Method Set:	20%qb
Injection #:	1	Processing Method:	xt 3 153 2 asy
Injection Volume:	10.00 ul	Channel Name:	266.0nm
Run Time:	60.0 Minutes	Proc. Chnl. Descr.:	2998 PDA 266.0 nm (2998
Date Acquired:	7/27/2022 10:32:31 PM CST		
Date Processed:	7/27/2022 10:49:18 PM CST		

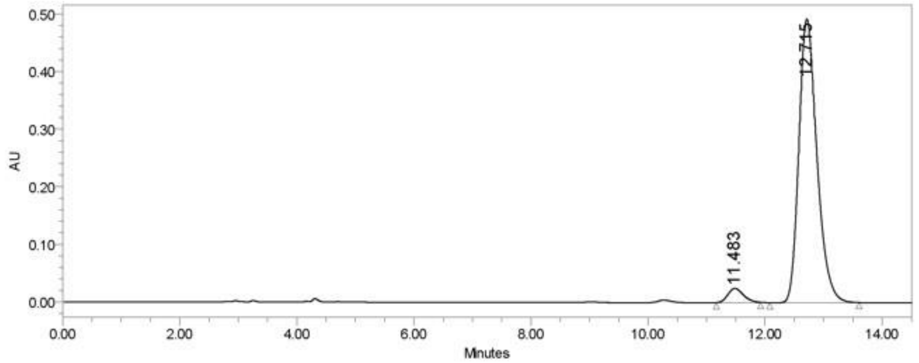

	RT	Area	% Area	Height
1	11.483	452637	4.13	24649
2	12.715	10499150	95.87	492455

Supplementary Figure 107. HPLC spectra of compound **7d**

Rac-**8**

SAMPLE INFORMATION

Sample Name:	xt-3-160-2-rac-10%-AD	Acquired By:	System
Sample Type:	Unknown	Sample Set Name	
Vial:	89	Acq. Method Set:	10% quanbo
Injection #:	1	Processing Method	XT 3 160 2 RAC
Injection Volume:	10.00 ul	Channel Name:	254.0nm
Run Time:	60.0 Minutes	Proc. Chnl. Descr.:	2998 PDA 254.0 nm (2998
Date Acquired:	8/23/2022 10:44:15 PM CST		
Date Processed:	10/20/2022 11:35:17 AM CST		

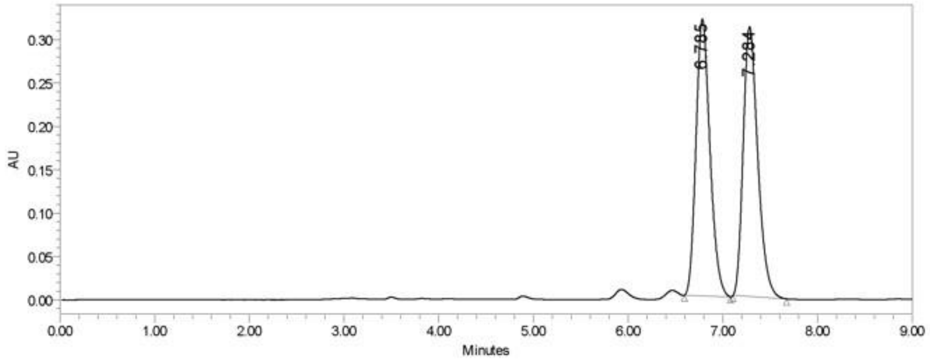

	RT	Area	% Area	Height
1	6.785	3284108	49.85	318831
2	7.284	3303752	50.15	310775

Asy-**8**

SAMPLE INFORMATION

Sample Name:	xt-3-160-2-asy-10%-AD	Acquired By:	System
Sample Type:	Unknown	Sample Set Name	
Vial:	91	Acq. Method Set:	10% quanbo
Injection #:	1	Processing Method	XT 3 160 2 ASY
Injection Volume:	10.00 ul	Channel Name:	254.0nm
Run Time:	60.0 Minutes	Proc. Chnl. Descr.:	2998 PDA 254.0 nm (2998
Date Acquired:	8/24/2022 7:12:16 PM CST		
Date Processed:	10/20/2022 11:34:59 AM CST		

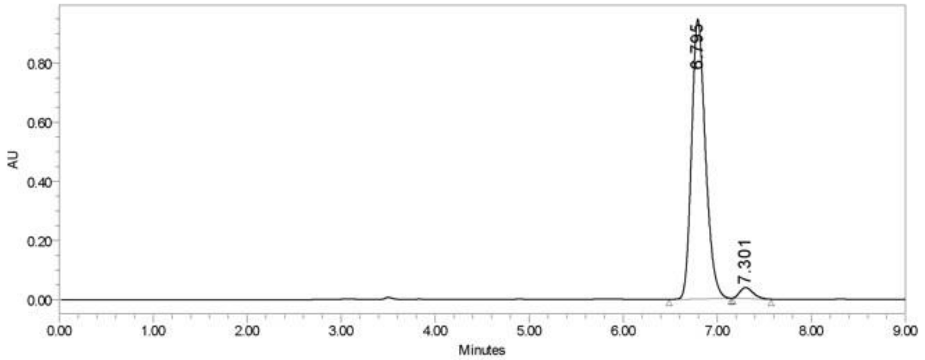

	RT	Area	% Area	Height
1	6.795	9772044	96.41	945989
2	7.301	363851	3.59	36810

Supplementary Figure 108. HPLC spectra of compound **8**

Rac-**9**

SAMPLE INFORMATION

Sample Name:	xt-3-160-3-RAC-30%-AD	Acquired By:	System
Sample Type:	Unknown	Sample Set Name:	
Vial:	95	Acq. Method Set:	30%qb
Injection #:	1	Processing Method:	XT 3 160 3 RAC
Injection Volume:	10.00 ul	Channel Name:	254.0nm
Run Time:	60.0 Minutes	Proc. Chnl. Descr.:	2998 PDA 254.0 nm (2998
Date Acquired:	8/26/2022 3:51:21 PM CST		
Date Processed:	10/20/2022 11:45:54 AM CST		

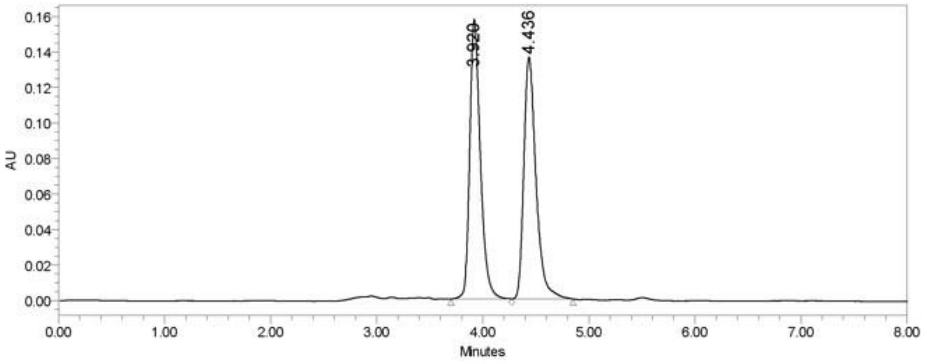

	RT	Area	% Area	Height
1	3.920	1096447	50.13	157422
2	4.436	1090647	49.87	136174

Asy-**9**

SAMPLE INFORMATION

Sample Name:	xt-3-160-3-ASY-30%-AD	Acquired By:	System
Sample Type:	Unknown	Sample Set Name:	
Vial:	110	Acq. Method Set:	30%qb
Injection #:	1	Processing Method:	xt 3 160 3 asy
Injection Volume:	10.00 ul	Channel Name:	254.0nm
Run Time:	60.0 Minutes	Proc. Chnl. Descr.:	2998 PDA 254.0 nm (2998
Date Acquired:	8/26/2022 4:05:16 PM CST		
Date Processed:	10/20/2022 11:43:51 AM CST		

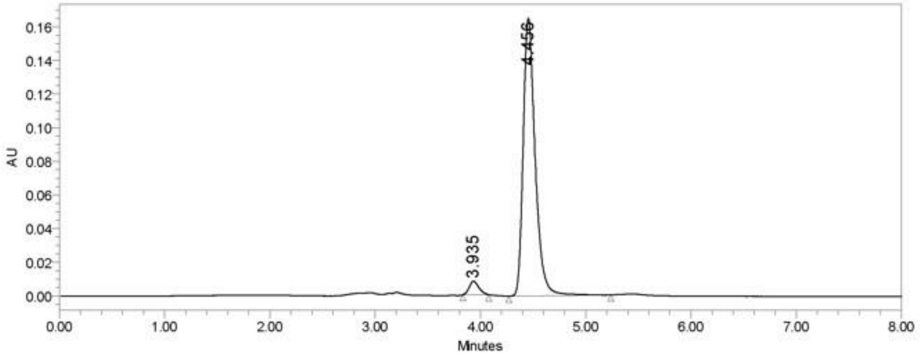

	RT	Area	% Area	Height
1	3.935	50667	3.74	7944
2	4.456	1303480	96.26	165077

Supplementary Figure 109. HPLC spectra of compound **9**

Rac-**11**

SAMPLE INFORMATION

Sample Name:	xt-3-160-5-rac-30%-IA	Acquired By:	System
Sample Type:	Unknown	Sample Set Name	1003
Vial:	39	Acq. Method Set:	30%quanbo
Injection #:	1	Processing Method	XT 3 160 5 RAC
Injection Volume:	10.00 ul	Channel Name:	316.0nm
Run Time:	10.0 Minutes	Proc. Chnl. Descr.:	2998 PDA 316.0 nm (2998
Date Acquired:	10/3/2022 12:08:15 PM CST		
Date Processed:	10/3/2022 2:47:06 PM CST		

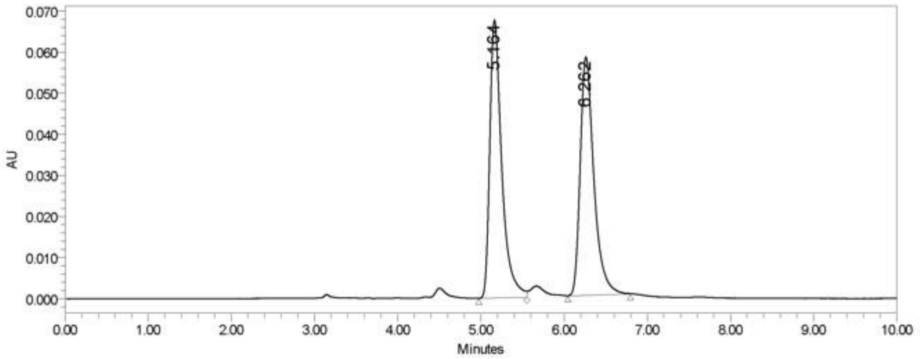

	RT	Area	% Area	Height
1	5.164	672467	50.50	67595
2	6.262	659281	49.50	57970

Asy-**11**

SAMPLE INFORMATION

Sample Name:	xt-3-160-5-asy-30%-IA	Acquired By:	System
Sample Type:	Unknown	Sample Set Name	
Vial:	15	Acq. Method Set:	30%quanbo
Injection #:	1	Processing Method	xt 3 160 5
Injection Volume:	10.00 ul	Channel Name:	316.0nm
Run Time:	60.0 Minutes	Proc. Chnl. Descr.:	2998 PDA 316.0 nm (2998
Date Acquired:	10/3/2022 9:42:39 AM CST		
Date Processed:	10/3/2022 11:49:46 AM CST		

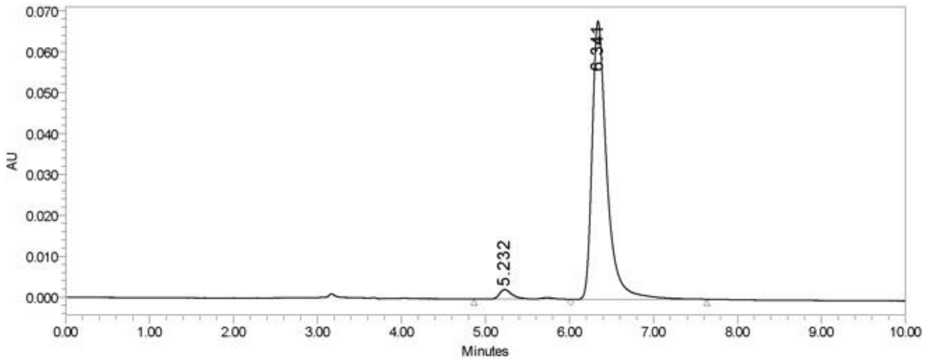

	RT	Area	% Area	Height
1	5.232	28184	3.29	2342
2	6.341	828885	96.71	67982

Supplementary Figure 110. HPLC spectra of compound **11**

Rac-**12**

SAMPLE INFORMATION

Sample Name:	xt-5-109-rac-30%-AD	Acquired By:	System
Sample Type:	Unknown	Sample Set Name:	
Vial:	69	Acq. Method Set:	30%qb
Injection #:	1	Processing Method:	xt 5 109 rac
Injection Volume:	20.00 ul	Channel Name:	268.0nm
Run Time:	60.0 Minutes	Proc. Chnl. Descr.:	2998 PDA 268.0 nm (2998
Date Acquired:	8/9/2023 10:17:03 AM CST		
Date Processed:	8/9/2023 10:32:24 AM CST		

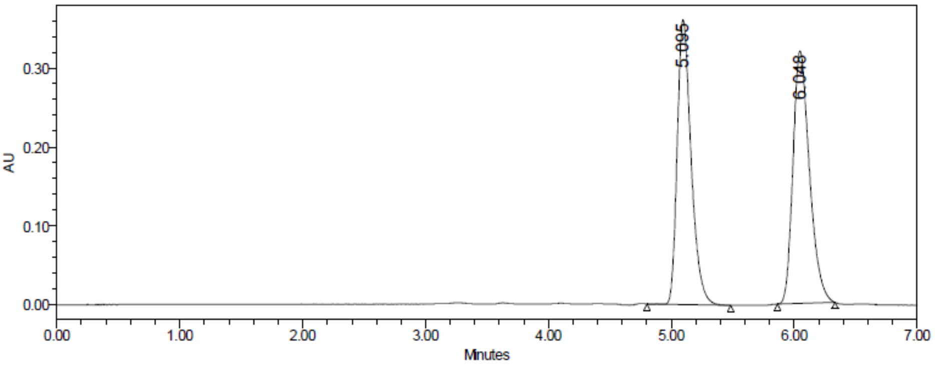

	RT	Area	% Area	Height
1	5.095	2837833	48.70	361083
2	6.048	2989318	51.30	319848

Asy-**12**

SAMPLE INFORMATION

Sample Name:	xt-5-109-2-30%-AD	Acquired By:	System
Sample Type:	Unknown	Sample Set Name:	
Vial:	37	Acq. Method Set:	30%qb
Injection #:	1	Processing Method:	xt 5 109 2
Injection Volume:	20.00 ul	Channel Name:	268.0nm
Run Time:	60.0 Minutes	Proc. Chnl. Descr.:	2998 PDA 268.0 nm (2998
Date Acquired:	8/9/2023 10:03:26 AM CST		
Date Processed:	8/9/2023 10:27:54 AM CST		

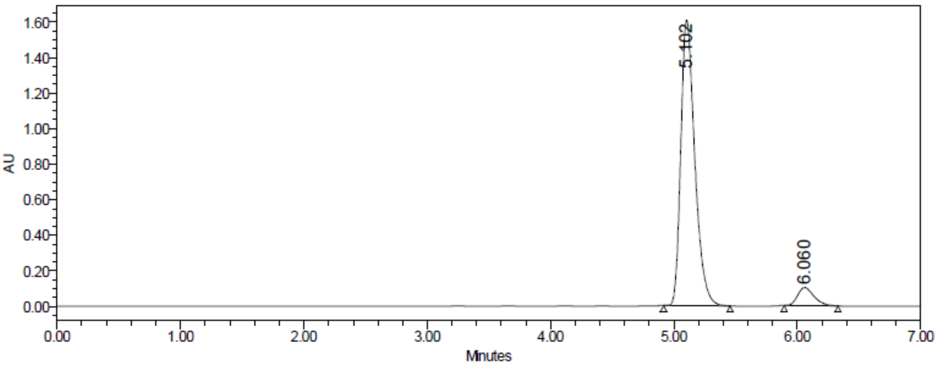

	RT	Area	% Area	Height
1	5.102	12689574	93.07	1608187
2	6.060	944169	6.93	102337

Supplementary Figure 111. HPLC spectra of compound **12**

4. Supplementary References

1. Liu, P., Huo, X., Li, B., He, R., Zhang, J., Wang, T., Xie, F. & Zhang, W. Stereoselective allylic alkylation of 1-pyrroline-5-carboxylic esters via a Pd/Cu dual catalysis. *Org. Lett.* **20**, 6564−6568 (**2018**).
2. Xiao, Q., He, Q., Li, J. & Wang, J. 1,4-Diazabicyclo[2.2.2]octane-promoted aminotrifluoromethylthiolation of α,β-unsaturated carbonyl compounds: *N*-trifluoromethylthio-4-nitrophthalimide acts as both the nitrogen and SCF_3 sources. *Org. Lett.* **17**, 6090−6093 (2015).
3. Li, L.-B., Dan, W.-J., Tan, F.-F., Cui, L.-H., Yuan, Z.-P., Wu, W.-J. & Zhang, J.-W. Synthesis and antibacterial activities of yanglingmycin analogues. *Chem. Pharm. Bull*. **63**, 33−37 (2015).
4. Maltsev, O. V., Walter, V., Brandl, M. J.& Hintermann, L. Medium buffer effects on the condensation of l-cysteine and aryl nitriles to (*R*)-2-aryl-4,5-dihydrothiazole-4-carboxylic acids. *Synthesis*. **45**, 2763−2767 (2013).
5. Huo, X., He, R., Fu, J., Zhang, J., Yang, G. & Zhang, W. Stereoselective and site-specific allylic alkylation of amino acids and small peptides via a Pd/Cu dual catalysis. *J. Am. Chem. Soc*. **139**, 9819−9822 (2017).
6. Mori-Quiroz, L. M. & Clift, M. D. Exploiting alkylquinone tautomerization: amine benzylation. *Org. Lett.* **18**, 3446-3449 (2016).
7. Retini, M., Bartolucci, S., Bartoccini, F., Mari, M. & Piersanti, G. Concise and convergent enantioselective total syntheses of (+)- and (−)-fumimycin. *J. Org. Chem*. **84**, 12221−12227 (2019).
